# Population genomics of wild Chinese rhesus macaques reveals a dynamic demographic history and local adaptation, with implications for biomedical research

**DOI:** 10.1093/gigascience/giy106

**Published:** 2018-08-27

**Authors:** Zhijin Liu, Xinxin Tan, Pablo Orozco-terWengel, Xuming Zhou, Liye Zhang, Shilin Tian, Zhongze Yan, Huailiang Xu, Baoping Ren, Peng Zhang, Zuofu Xiang, Binghua Sun, Christian Roos, Michael W Bruford, Ming Li

**Affiliations:** 1 Chinese Academy of Sciences Key Laboratory of Animal Ecology and Conservation Biology, Institute of Zoology, Beichen West Road, Chaoyang District, Beijing, 100101, China; 2 University of Chinese Academy of Sciences, Yuquan Road, Shijingshan District, Beijing, 100049, China; 3 School of Biosciences, Cardiff University, Sir Martin Evans Building, Museum Avenue, Cardiff CF10 3AX, United Kingdom; 4 Division of Genetics, Department of Medicine, Brigham and Women's Hospital, Harvard Medical School, Boston, MA 02115, USA; 5 Novogene Bioinformatics Institute, Jiuxianqiao North Road, Chaoyang District, Beijing, 100083, China; 6 Institute of Physical Science and Information Technology, Anhui University, Jiulong Road, Hefei, 230601, China; 7 College of Life Science, Sichuan Agricultural University, Xinkang Road, Yucheng District, Ya'an, 625014, China; 8 School of Sociology and Anthropology, Sun Yat-sen University, Xingang Xi Road, Guang Zhou, 510275, China; 9 College of Life Science and Technology, Central South University of Forestry and Technology, Shaoshan South Road, Changsha, 410004, China; 10 School of Life Sciences, Anhui University, Jiulong Road, Hefei, 230601, China; 11 Gene Bank of Primates and Primate Genetics Laboratory, German Primate Center, Leibniz Institute for Primate Research, Kellnerweg 4, Göttingen, 37077, Germany; 12 Center for Excellence in Animal Evolution and Genetics, Chinese Academy of Sciences, Kunming, 650223, China

**Keywords:** *Macaca mulatta*, population genomics, adaptive selection, biomedical model

## Abstract

**Background:**

The rhesus macaque (RM, *Macaca mulatta*) is the most important nonhuman primate model in biomedical research. We present the first genomic survey of wild RMs, sequencing 81 geo-referenced individuals of five subspecies from 17 locations in China, a large fraction of the species’ natural distribution.

**Results:**

Populations were structured into five genetic lineages on the mainland and Hainan Island, recapitulating current subspecies designations. These subspecies are estimated to have diverged 125.8 to 51.3 thousand years ago, but feature recent gene flow. Consistent with the expectation of a larger body size in colder climates and smaller body size in warmer climates (Bergman's rule), the northernmost RM lineage (*M. m. tcheliensis*), possessing the largest body size of all Chinese RMs, and the southernmost lineage (*M. m. brevicaudus*), with the smallest body size of all Chinese RMs, feature positively selected genes responsible for skeletal development. Further, two candidate selected genes (*Fbp1, Fbp2*) found in *M. m. tcheliensis* are involved in gluconeogenesis, potentially maintaining stable blood glucose levels during starvation when food resources are scarce in winter. The tropical subspecies *M. m. brevicaudus* showed positively selected genes related to cardiovascular function and response to temperature stimuli, potentially involved in tropical adaptation. We found 118 single-nucleotide polymorphisms matching human disease-causing variants with 82 being subspecies specific.

**Conclusions:**

These data provide a resource for selection of RMs in biomedical experiments. The demographic history of Chinese RMs and their history of local adaption offer new insights into their evolution and provide valuable baseline information for biomedical investigation.

## Introduction

Understanding how species evolve and adapt to their environments is an essential question in evolutionary biology. Rhesus macaques (RMs, *Macaca mulatta*) are, after humans, the world's most widely distributed primates [1–5], occupying a vast geographic distribution spanning from Afghanistan to the Chinese shore of the Pacific Ocean and south into Myanmar, Thailand, Laos, and Vietnam [5]. As the most widely distributed nonhuman primate species, RMs occupy diverse ecological landscapes and habitats, making them an interesting model to address questions about how species evolve and adapt to local environmental variation, including characterizing the genomic architecture of adaptation to habitat, climate, and other biotic and abiotic factors. Yet, despite much work on primate comparative genomics, very few population genomic studies have been carried out on wild RMs [6, 7]. Importantly, as RMs are widely used as a primate model in physiological, psychological, and cognitive studies [8–10], knowledge about their genomic architecture could improve and refine biomedical research [10] as the genomic composition of experimental animals can have a considerable influence on the outcome of experiments [11, 12]. Therefore, information on the genomic diversity of captive and wild RMs that could become a genomic resource for future utilization in medical research is essential.

In biomedical research, two main RM populations (Indian and Chinese) are recognized [6, 13]. They diverged from each other ∼162 thousand years ago (kya) and are characterized by extensive differences in morphology, behavior, ecology, physiology, reproduction, and disease progression [6, 13–19]. In 1978 India banned all RM exports to breeding centers across the world, thus curtailing the availability of wild Indian RMs and subsequently increasing the demand for Chinese RMs in biomedical research, thereby making a detailed characterization of genetic variants from Chinese RMs crucial for biomedical usage of this species.

Until recently, the genomes of 133 captive RMs from eight colonies have been sequenced; however, 124 of them are of Indian origin and only 9 individuals were presumed to be of Chinese origin [6]. In addition, Zhong et al. [7] reported genomic variation in 26 Chinese captive RMs identifying ∼46 million (M) single-nucleotide polymorphisms (SNPs). Nevertheless, most of the RM genetic variation known to date is limited to captive populations that may contain composite genotypes due of admixture among animals of different and unclear origin [20]. Here, we present the first attempt to survey the geo-referenced genomic diversity in wild Chinese RM populations, which is the largest extant population of the species. The current effective population size of Chinese and Indian RMs was estimated to be approximately 240,000 and 17,000, respectively, indicating that the Chinese RMs are likely to harbor substantially more genomic diversity compared to their Indian conspecifics [13]. Therefore, this population genomic survey of 81 RMs originating from 17 wild locations across China, including phylogenetic and demographic analyses as well as genome-wide selection scans, corresponds to the most comprehensive characterization of RM genetic diversity to date. The aim in this survey is to characterize the processes that lead to the extant patterns of variability as well as to identify the potential implications for the use of these populations in biomedical research.

## Results and Discussion

### Genetic diversity, phylogeny, and population structure

**Figure 1:**
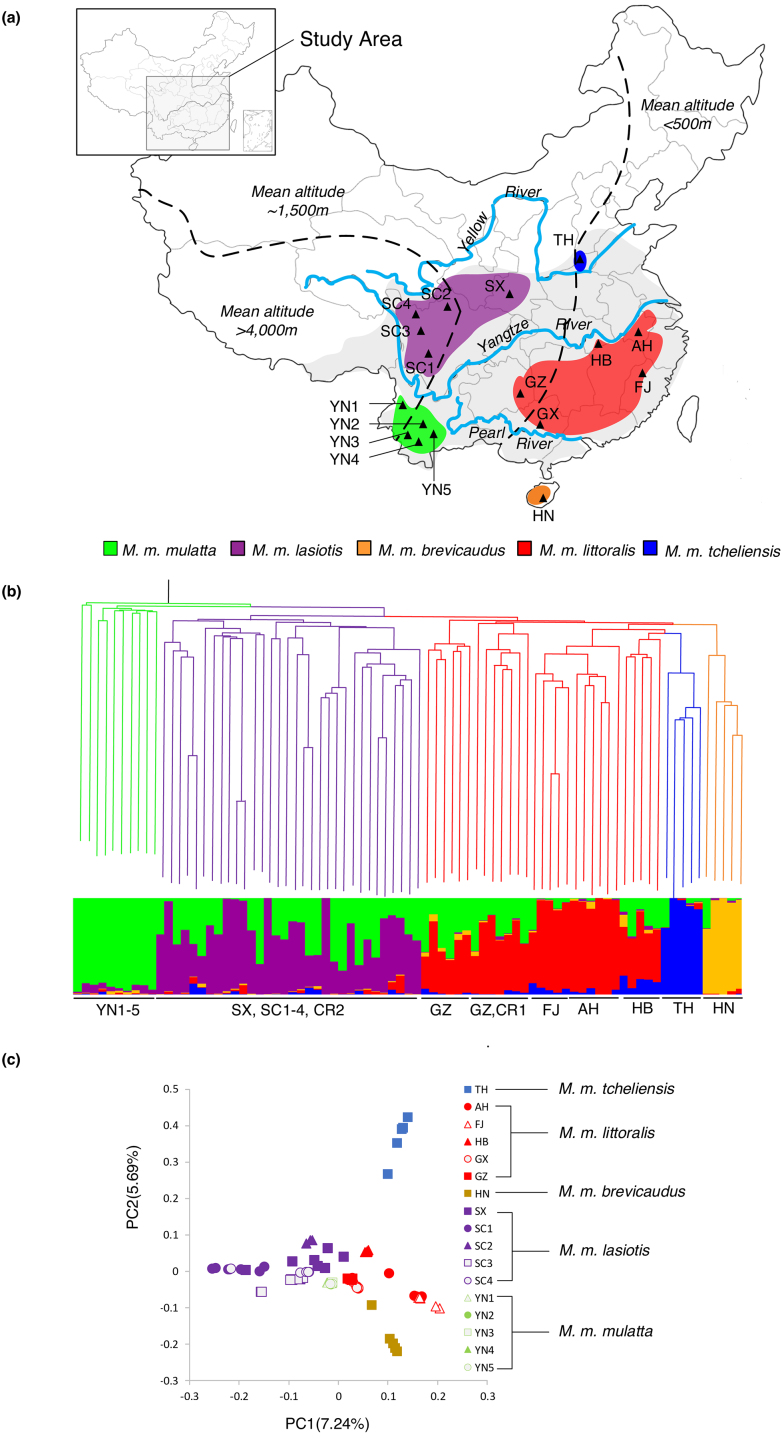
Phylogeny and population genetic structure of 81 wild Chinese RMs. **(a)** Geographic distribution of RMs in China (gray shadow) and the 17 sampling sites along with their subspecies assignment. **(b)** Neighbor-joining (NJ) tree and clustering solution inferred using STRUCTURE and displaying five populations (inferred with Evanno's ∆*K* method; Supplementary Fig. S5). **(c)** Principal component analysis plots depicting the first two components (variance explained by PC1 = 7.24% and PC2 = 5.69%).

Blood and tissue samples from 79 wild-born RMs, representing five subspecies [21, 22], were collected at 17 sites in China (*M. m. tcheliensis*: TH; *M. m. littoralis*: AH, FJ, HB, GX, GZ; *M. m. brevicaudus*: HN; *M. m. lasiotis*: SX, SC1, SC2, SC3, SC4; *M. m. mulatta*: YN1, YN2, YN3, YN4, YN5; Fig. 1a and Supplementary Table S1). Genome sequences of two additional Chinese RMs (CR1 and CR2) were retrieved from the National Center for Biotechnology Information (NCBI) [9, 23, 24]. Resequencing was at a high average depth of 28.06 ± 5.08 × for 10 individuals and a moderate average depth of 9.98 ± 1.05 × for the remainder (n = 71), with an overall average genome coverage of 93.77% of the RM reference (Mmul_8.0.1; Supplementary Table S1). A total of 52,534,348 autosome SNPs were identified in these 81 wild Chinese RMs (Supplementary Table S2), and the nucleotide diversity measured by segregating sites (Watterson's θ, θ_W_) and mean pairwise differences (θπ) were 0.00375 and 0.00247, respectively (Table 1). The number of SNPs (all positions with differences to the genome reference) per individual ranged from 7.0 to 9.2 M (mean of 8.50 M; Supplementary Fig. S1 and Supplementary Table S3). Among all detected SNPs, 8,171,139 were shared among all subspecies and 22,768,395 were shared by at least two subspecies, with the remaining SNPs confined to a single subspecies (Supplementary Fig. S2a). For each subspecies, the subspecies-specific SNPs (ssSNPs) ranged from 702,099 to 7,736,924 and the nonsynonymous ssSNPs ranged from 3,056 to 25,960 (Supplementary Fig. S2a, S2b). Among Chinese RM subspecies, *M. m. mulatta* had the highest heterozygosity (2.29 × 10^−3^ ± 3.24 × 10^−5^), followed by *M. m. lasiotis* (2.04 × 10^−3^± 1.40 × 10^−4^) and *M. m. littoralis* (2.00 × 10^−3^ ± 1.18 × 10^−4^). The lowest heterozygosity rates were found in *M. m. brevicaudus* (1.82 × 10^−3^ ± 1.28 × 10^−4^) and *M. m. tcheliensis* (1.46 × 10^−3^ ± 2.65 × 10^−4^) (Supplementary Fig. S3).

**Table 1:** Genetic diversity (θ) and effective population size (*N*_e_) in Chinese rhesus macaques based on segregating sites (θ_W_) and nucleotide diversity (θπ)

			θ_W_		θπ	
		Sample size (n)	θ	*N*_e_	θ	*N*_e_
Chinese rhesus macaques (all samples)		81	0.00375	93 750	0.00247	61 750
Subspecies	*M. m. littoralis*	29	0.00313	78 250	0.00240	60 000
	*M. m. tcheliensis*	5	0.00215	53 750	0.00230	57 500
	*M. m. brevicaudus*	5	0.00203	50 750	0.00207	51 750
	*M. m. lasiotis*	32	0.00298	74 500	0.00239	59 750
	*M. m. mulatta*	10	0.00303	75 750	0.00245	61 250

We reconstructed a neighbor-joining (NJ) tree for Chinese RMs based on autosomal SNPs, using Indian RMs and *M. sylvanus* as outgroups (Fig. 1b and Supplementary Fig. S4). Individuals from *M. m. lasiotis, M. m. brevicaudus*, and *M. m. tcheliensis* form monophyletic lineages, respectively, while *M. m. mulatta* and *M. m. littoralis* are paraphyletic. Next, we performed a population structure analysis using STRUCTURE (version 2.3.4) [25], which estimates individual ancestry and admixture proportions assuming *K* ancestral populations. Plots of *ΔK* generated from STRUCTURE results indicated five genetic clusters present in the full dataset (Fig. 1b and Supplementary Fig. S5). A principal component analysis (PCA) corroborated the division of Chinese RMs into five groups. The first eigenvector separated *M. m. mulatta* and *M. m. lasiotis* from *M. m. tcheliensis, M. m. littoralis*, and *M. m. brevicaudus* (variance explained = 7.24%, Tracy-Widom *P* = 4.78 × 10^−44^), and the second eigenvector further separated *M. m. tcheliensis, M. m. littoralis*, and *M. m. brevicaudus* (variance explained = 5.69%, Tracy-Widom *P* = 4.21 × 10^−27^) (Fig. 1c, Supplementary Table S4). The division of Chinese RMs into five geographic lineages supports the former taxonomic division of Chinese RMs into five subspecies [21, 22]. *M. m. mulatta* (YN1–5) and *M. m. lasiotis* (SC1–4, SX) form the pan-western populations of Chinese RMs, with both subspecies inhabiting the montane Tibetan Plateau regions with an altitude ≥1500 meters above sea level in western China and separated from each other by the Yangtze River. *M. m. littoralis* (AH, FJ, HB, GX, GZ), *M. m. tcheliensis* (TH), and *M. m. brevicaudus* (HN) occur in the eastern coastal lowland of China and form the pan-eastern population. *M. m. tcheliensis* from the Taihang Mountains area is the northernmost (34°54’–35°16’ N; 112°02’–112°52’ E), while *M. m. brevicaudus*, restricted to Hainan Island, is the most southern Chinese RM subspecies.

### Demographic and phylogeographic history

**Figure 2:**
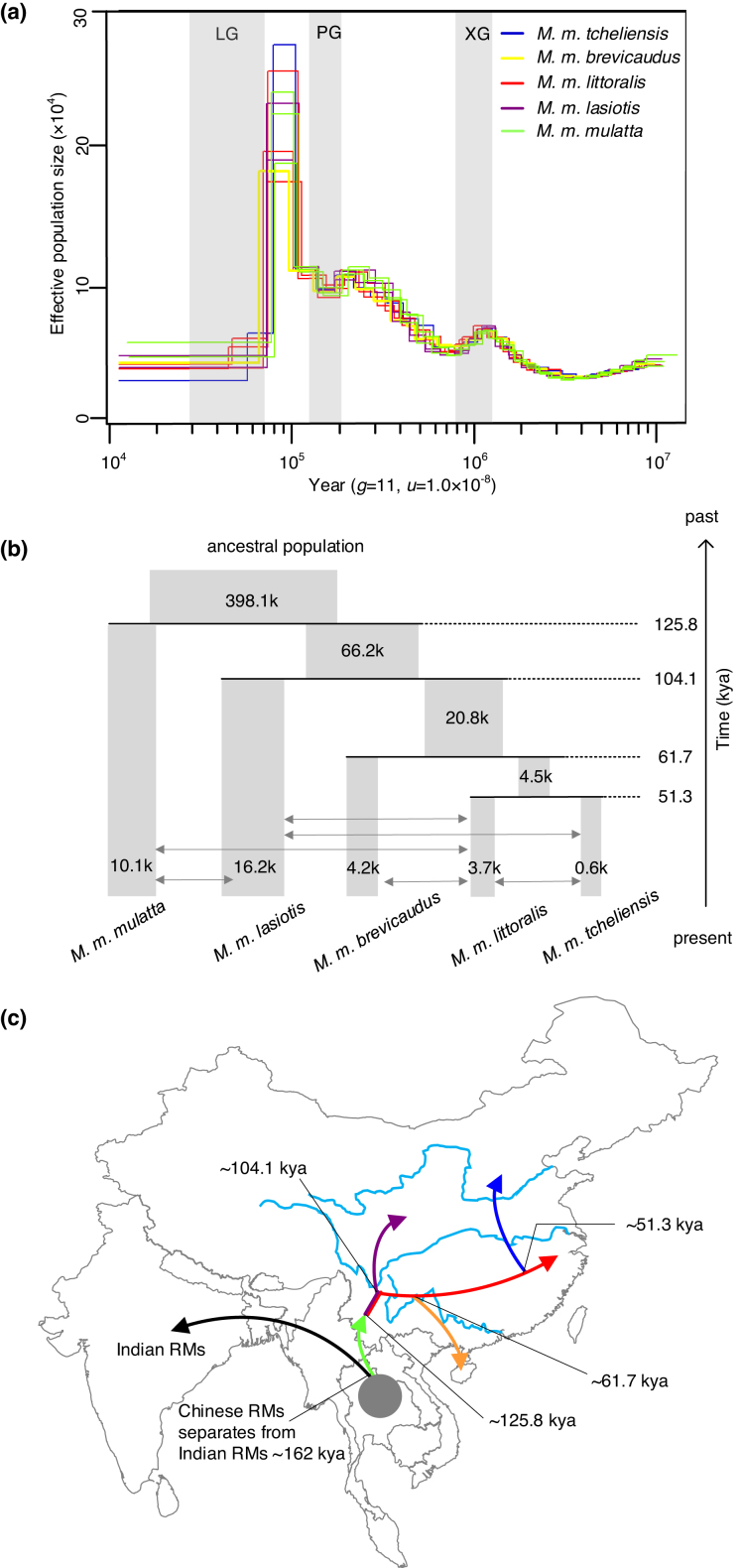
Demographic history and differentiation scenarios of Chinese RMs. **(a)** Historical changes in effective population size reconstructed using the PSMC applied on individual whole genomes for each of the five subspecies. The generation length (*g*) and the neutral mutation rate per generation (*μ*) were assumed to be 11 years and 1.08 × 10^–8^, respectively. The Xixiabangma Glaciation (1200–800 kya), Penultimate Glaciation (200–130 kya), and Last Glaciation (70–10 kya) are shaded in gray. **(b)** Demographic history inferred by *fastsimcoal2*. The width of the gray bars and numbers on them indicate the estimated effective population size (all effective population sizes were converted to individuals). The arrows indicate migration rate between different subspecies. The detailed migration rates are listed in Supplementary Table S5. Numbers at the right show the divergence times between subspecies (all times were converted to years assuming a generation time of 11 years). **(c)** Biogeographic scenario for RMs. Chinese RMs separated from Indian RMs ∼162 kya [13], followed by further migration into China by the different RM subspecies indicated with arrows colored following the color key in Fig. 1a.

The estimated effective population sizes, based on θ_W_ and θπ, are approximately 93,750 and 61,750 for Chinese RMs (Table 1). In order to infer the ancient demographic history of Chinese RMs, we applied a pairwise sequential Markovian coalescent (PSMC) [26] analysis using 10 RM individuals with an average sequencing coverage depth higher than 20 × (one individual of *M. m. tcheliensis* and one of *M. m. brevicaudus*, two of *M. m. lasiotis*, three of *M. m. littoralis*, as well as three individuals of *M. m. mulatta*). The inferred PSMC trajectories were very similar for all analyzed individuals throughout most of the species’ history, reflecting the species’ cohesiveness (Fig. 2a). The ancient demographic history of RMs is marked by population fluctuations following the glacial periods during the Pleistocene [27]. Approximately 1200–800 kya all Chinese RMs experienced a population reduction at the time of the Xixiabangma Glaciation, followed by an expansion during the Mid-Pleistocene inter-glaciation (800–200 kya). This expansion was then interrupted by the Penultimate Glaciation (200–130 kya) when suitable habitat might have been lost leading to a population decline [27]. PSMC analyses also suggested that all the Chinese RMs had a population expansion during the last interglacial (around 100 kya) and a subsequent bottleneck during the Last Glaciation (LG; 70–10 kya) (Fig. 2a). Interestingly, the demographic inference by Xue et al. [6], derived from genomic data of a single Chinese RM (CH_37 945) from AH (*M. m. littoralis*), qualitatively resembled the demographic trajectory of *M. m. littoralis* presented herein.

To further describe the divergence process among the five Chinese RM subspecies, we also employed the SVDquartes approach [28–31] that takes incomplete lineage sorting into account. The obtained phylogenetic tree suggests a “step-by-step” divergence of the five subspecies. Accordingly, the *M. m. mulatta* lineage diverged from that of the remaining Chinese RMs first, and then the *M. m. lasiotis* diverged from the ancestral lineage of pan-eastern RMs (*M. m. tcheliensis, M. m. littoralis*, and *M. m. brevicaudus*). Subsequently, *M. m. brevicaudus* diverged from the ancestor of *M. m. tcheliensis* and *M. m. littoralis*, the divergence of which occurred last (Supplementary Fig. S6). Under this step-by-step divergence scenario, we performed the joint site frequency spectrum (SFS)-based approach implemented in *fastsimcoal2* [32] to model demographic fluctuations, respective divergence times, and gene flow events among the five RM subspecies. Following the divergence between the ancestral lineages of Indian and Chinese RMs (∼162 kya), the ancestor of *M. m. mulatta* diverged from the remaining Chinese RMs ∼125.8 kya (95% confidence interval [CI]: 92.0–162.1 kya) (Fig. 2b) [6, 13]. Subsequently, *M. m. lasiotis* diverged from the ancestral lineage of pan-eastern RMs ∼104.1 kya (95% CI: 50.2–154.5 kya) near the end of the last interglacial. The divergence time between *M. m. brevicaudus* and the ancestor of *M. m. tcheliensis* and *M. m. littoralis* was estimated at ∼61.7 kya (95% CI: 43.6–115.1 kya), while the divergence between the latter two occurred ∼51.3 kya (95% CI: 7.2–55.4 kya) during the last glacial maximum [33, 34]. Interestingly, the coalescence analysis revealed a large ancestral population size of the Chinese RMs 125.8 kya (95% CI: 92.0–162.1 kya) and a subsequent population decline and divergence among the five subspecies (Fig. 2b), which coincided with the population expansion during the last interglacial (around 100 kya) and the subsequent bottleneck of Chinese RMs during the LG (70–10 kya) revealed by PSMC analyses. Our results indicate that substantial gene flow occurred between all five extant lineages of Chinese RMs (Fig. 2b, Supplementary Table S5, and Supplementary Fig. S7).

A previous study of mitochondrial DNA identified two major haplogroups dividing Chinese RMs into a western and an eastern clade. Modern Chinese RMs were thought to have undergone a northward expansion while entering China via two possible routes: the first into the western mountains and the second following the eastern coast [35]. Our evolutionary model, however, suggests a step-by-step colonization process of RMs in China (Fig 2c). After the divergence from the Indian population (∼162 kya) [6, 13], the ancestor of Chinese RMs colonized the Tibetan Plateau from southwestern China and then experienced a range expansion north and eastward. The pan-western population (*M. m. mulatta* and *M. m. lasiotis*) inhabited the western montane region in China, while the pan-eastern population (*M. m. tcheliensis, M. m. littoralis*, and *M. m. brevicaudus*) entered the eastern coastal region. These five subspecies further diverged from each other during the bottleneck caused by the LG. Additionally, barriers such as the Yellow, Yangtze, and Pearl rivers and open sea (Fig. 1a) led to further differentiation by limiting gene flow among them. Water bodies and mountains could therefore be described as driving the formation of a habitat “lattice,” with the different subspecies of RMs occupying different grids in the lattice.

### Signatures of selection and local adaptation

**Figure 3:**
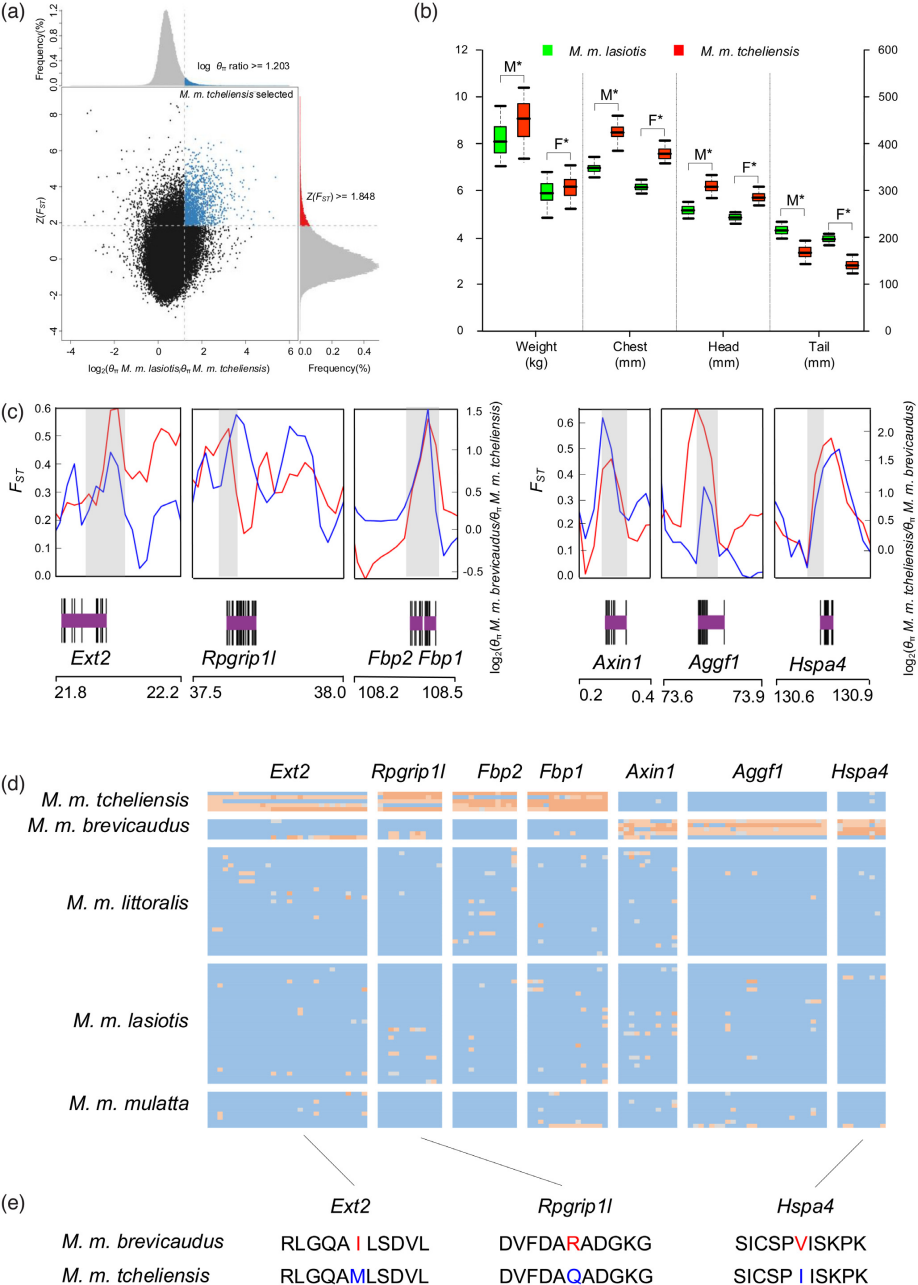
Genomic regions with selection-sweep signals in RM. **(a)** Distribution of log_2_ (*θ*_π_*M. m. lasiotis*_/_*θ*_π_*M. m. tcheliensis*) and Z (*F_ST_*) of 50-kb windows with 25-kb steps. Blue dots located in the selected regions requirement (corresponding to Z test *P*< 0.05, where Z (*F_ST_*) ≥ 1.848 and *θ*_π_ log-ratio ≥ 1.203) represent selected windows for *M. m. tcheliensis*. **(b)** Morphological comparison between *M. m. tcheliensis* and *M. m. lasiotis*. M and F represent males and females. **(c)** Example of genes with selection sweep signals. *Ext2, Rpgrip1l, Fbp2*, and *Fbp1* in *M. m. tcheliensis* and *Axin1, Aggf1*, and *Hspa4* in *M. m. brevicaudus. F_ST_* and *θ*_π_ log-ratios between the two subspecies are represented in red and blue, respectively. All values in Fig. 3c are plotted using 50-kb windows with half steps. Genome annotations are show at the bottom (black bar, coding sequences (purple bar, genes). **(d)** SNP genotypes in putative selective sweeps containing *Ext2, Rpgrip1l, Fbp2, Fbp1, Axin1, Aggf1*, and *Hspa4*. **(e)** Nonsynonymous variants in gene *Ext2, Rpgrip1l* and *Hspa4*.

The wide distribution of Chinese RMs and their respective contrasting habitat types, as well as their wide use in biomedical studies, makes them an important case study for the analysis of signatures of local adaptation to divergent selective pressures [36–38]. We identified putative targets of selection by carrying out pairwise comparisons between RM subspecies inhabiting the most different environments to increase the chance of finding selection signatures, i.e., *M. m. tcheliensis* that occurs in the northernmost range of the species under cold conditions and *M. m. brevicaudus* that inhabits the southernmost range of the species, a tropical island. For each analysis, we compared the target subspecies (i.e. *M. m. tcheliensis* or *M. m. brevicaudus*) to the each of the other subspecies using the fixation index (*F*_ST_) and genetic diversity (*θ*_π_), calculated on 50-kb-long sliding windows (Fig. 3 and Supplementary Figs. S8–S13). The top 5% of the windows with the largest *F*_ST_ and *θ*_π_ ratios (*θ*_π_2/θ_π_1) in each pairwise comparison were considered to be potentially under positive selection. For each target subspecies, we identified potential selective-sweep regions as the intersection between the top 5% outliers in all pairwise comparisons (four pairwise comparisons in each case) (Supplementary Fig. S8). We used these consistent selective-sweep regions for further analyses, as they represent robust putative positively selected regions. The sizes of candidate selective-sweep regions ranged from 0.100 Mb to 11.075 Mb, and the number of genes located in these regions, which are expected to represent targets of selection for each subspecies, varied from 6 to 176 in different subspecies (Supplementary Table S6).

*M. m. tcheliensis* from the Taihang Mountains area is the northernmost population of the species. The Taihang Mountains are characterized by a continental monsoon climate, and conditions for RMs are harsh during winter and early spring, with extreme cold temperatures of –14°C [39]. Food resources are limited and consist mainly of barks, twigs, roots of crops, and withered grass, thus, all sources are high in fiber but low in energy and nutritional value [40, 41]. Therefore, *M. m. tcheliensis* suffers from starvation due to food shortages during winter and early spring. In starvation, blood glucose levels are maintained by gluconeogenesis through which glucose is converted from other molecules, such as amino acids and lactic acid [42]. For *M. m. tcheliensis*, the positive-selection genes are enriched in the gene ontology (GO) term “fructose 1, 6-bisphosphate 1-phosphatase activity” with two genes (*Fbp1, Fbp2*; modified Fisher exact *P* = 1.90E-02; Fig. 3c, 3d; Supplementary Table S7). These two genes encode for fructose-1, 6-bisphosphatase 1 and fructose-1, 6-bisphosphatase isozyme 2, which catalyze the hydrolysis of fructose 1, 6-bisphosphate and play a rate-limiting role in gluconeogenesis. Furthermore, in starved zebrafish it was shown that the expression of *Fbp1* was significantly upregulated in brain and liver tissues [43]. The positive-selection genes are also enriched in other terms and pathways related to gluconeogenesis, including the Kyoto Encyclopedia of Genes and Genomes (KEGG) pathway “fructose and mannose metabolism” (modified Fisher exact *P* = 4.35E-02) and GO terms “hexose biosynthetic process,” “monosaccharide biosynthetic process,” and “cellular carbohydrate biosynthetic process” (modified Fisher exact *P* = 3.36E-02, *P* = 4.64E-02, and *P* = 2.65E-02, respectively; Supplementary Table S7). Our findings suggest that the regulation of gluconeogenesis might be a mechanism of *M. m. tcheliensis* to adapt to food shortages in winter.

According to Bergman's rule, animals living in cold climates tend to have larger body sizes compared to their relatives in warm climates (i.e., they have a lower surface area to volume ratio), so they radiate less body heat per unit of mass [44]. Consistent with this expectation, among all RM subspecies, *M. m. tcheliensis* exhibits the largest body size and mass and the largest head and chest circumference (Fig. 3b and Supplementary Table S8) [40, 45]. Among the consistent signatures of positive selection identified in *M. m. tcheliensis* (176 genes), we found signatures of selective sweeps in eight genes linked to limb morphogenesis or skeletal system development (Supplementary Table S6). Among these genes, *Fto* and *Rpgripl1* play an essential role in post-natal growth of mammals [46]. Mice lacking *Fto* completely display immediate post-natal growth retardation, with shorter body length, lower body weight, and lower bone mineral density, than control animals [47]. Furthermore, *Sox5* and *Sox6* (Fig. 3c, 3d) play an essential role in synovial joint morphogenesis via promoting both growth plate and articular chondrocyte differentiation [48]. Mutations in *Atp6v0a4* could cause developmental delay and delayed closure of the anterior fontanelle in human [49], while expression of *Ext2* enhances the bone formation in mice [50]. These genes, which are involved in the growth and development of the skeletal system and appendages, are likely contributors to the larger body size of *M. m. tcheliensis* and represent an undescribed adaptive pathway for primates living in colder climates.

In contrast, *M. m. brevicaudus* inhabits the tropical island of Hainan where it copes with a mean annual temperature of 24°C. *M. m. brevicaudus* has the smallest body size and the smallest body mass among RM subspecies [45]. As described above, they radiate more body heat per unit of mass (Bergman's rule) [44]. We found 127 putatively selected genes in *M. m. brevicaudus* (Supplementary Table S6), four of which were found to be enriched in GO term “bone morphogenetic protein (BMP) signaling pathway” (modified Fisher exact *P* = 4.65E-02; Supplementary Table S9), and two genes were found to be enriched in GO term “I-SMAD (inhibitory small mothers against decapentaplegic) binding" (modified Fisher exact *P* = 4.65E-02; Supplementary Table S9). BMP and I-SMAD signaling pathways are involved in the development of bones and the skeleton [51, 52]. Mutations in *Axin1*, a gene of the I-SMAD pathway, cause kinked tails in mice [53]. In *M. m. brevicaudus*, we found two nonsynonymous mutations in this gene (A674G, T656I) (Supplementary Fig. S14 and Supplementary Tables S10 and S11).

Additionally, putatively selected genes in *M. m. brevicaudus* (Fig. 3c, 3d and Supplementary Table S6) were also involved in GO terms related to cardiovascular system and blood circulation, e.g., *Aggf1* related to GO term “blood vessel morphogenesis” and *Ctnna3* related to GO term “regulation of heart rate by cardiac conduction.” The upregulated *Aggf1* expression is capable of increasing blood flow in mouse hindlimb [54]. In addition, *Hspa4*, heat shock 70kDa protein 4, is directly involved in GO term “response to temperature stimulus.” We thus hypothesize that the cardiovascular system of *M. m. brevicaudus* might play an important role in stabilizing body temperature, assisted by blood flow through different body parts requiring good fluidity and vascular permeability to transfer heat out of the body [55]. Testing these hypotheses needs further functional assays; however, these genes, together with the positively selected genes identified in *M. m. tcheliensis*, are known to be relevant to human physical function and thus are likely of importance in the adaptation of Chinese RMs to different climate conditions.

Both coding and noncoding changes could contribute to local adaptations of organisms [56]. To further investigate the adaptive mechanism of *M. m. tcheliensis* and *M. m. brevicaudus* to the opposite climates (cold vs hot), we focused on SNPs in the gene regions of above-described candidate genes. A total of 5,817 SNPs were found with significant differences at the 5% level in the distributions of genotypes between these two subspecies, and 10 SNPs were nonsynonymous variants (Supplementary Tables S10 and S11). In *M. m. tcheliensis*, nonsynonymous mutations were found in the coding regions of *Atp6v0a4* (R667Q), *Ext2* (I363M), *Fto* (N10S), and *Rpgrip1l* (R1281Q) (Supplementary Table S11 and Supplementary Fig. S14), implying that selection might have acted on protein sequence changes. No nonsynonymous changes were detected in *Fbp1, Fbp2, Sox5*, and *Sox6*. However, SNPs are located in the 1 kb up/downstream, 5’ and 3’ UTR, and intronic regions of these genes (Supplementary Table S10), indicating selection on noncoding regulatory variants. In addition, nonsynonymous mutations in *Aggf1* (H343Y), *Axin1* (A674G, T656I), *Hspa4* (I782V), and *Ctnna3* (V551I, T577M) were revealed for *M. m. brevicaudus* (Supplementary Table S11 and Supplementary Fig. S14).

In addition to the genes related to the adaptation to various climate conditions, we also found signatures of positive selection in genes related to the nervous system. In *M. m. tcheliensis*, the 176 identified candidate genes are enriched in GO term “synapse” (modified Fisher exact *P* = 4.28E-02; Supplementary Table S7) with eight genes; two of these gene, *Gabra2* and *Chrm2*, are associated with alcohol dependence [57]. For *M. m. brevicaudus*, 18 putatively selected genes related to nervous system development were found. For example, *Dcc* is reported to be required for long-term potentiation and memory [58]. *Auts2*, one of the eight putatively selected genes in *M. m. lasiotis*, has been shown to regulate neuronal migration, and mutations in this gene cause mental dysfunction in human [59] (Supplementary Table S6). Our findings suggest that RM subspecies have experienced different adaptive processes in the nervous system. Respective genomic differences should be taken into account when animals are selected for neurobiological research.

### Disease-causing variants and implication for biomedical research

**Figure 4:**
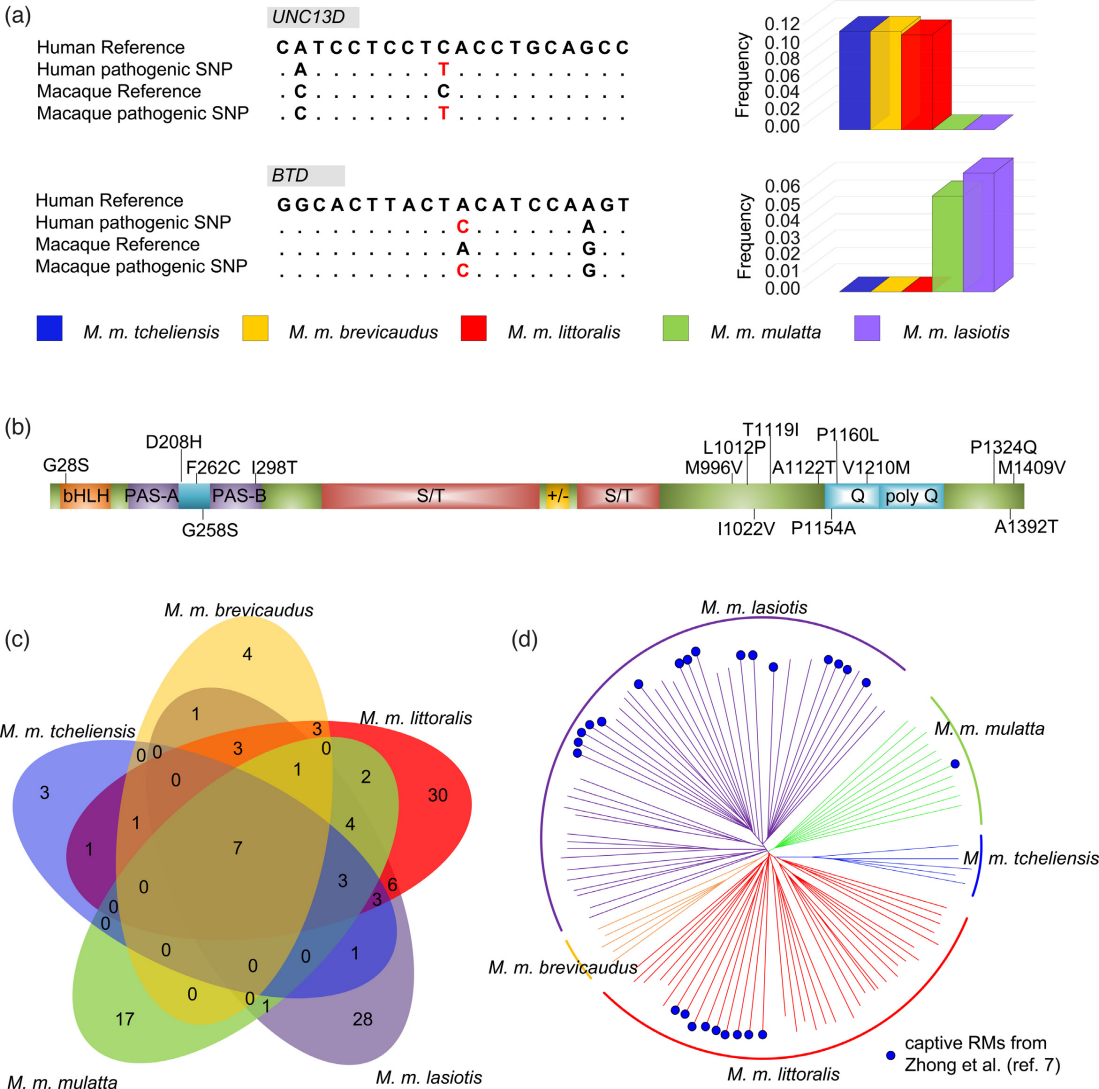
Population study of putative pathogenic SNPs in Chinese RM subspecies. **(a)** The site and frequency of pathogenic SNPs located in *Unc13d* and *Btd* genes. **(b)** Scheme of the *Ncoa3* gene in RM. The positions of nonsynonymous polymorphisms (black) and three amino acid deletions (in red) are marked. **(c)** Private and shared pathogenic SNPs in Chinese RM subspecies (blue: *M. m. tcheliensis;* orange: *M. m. brevicaudus;* red: *M. m. littoralis;* green: *M. m. mulatta;* purple: *M. m. lasiotis*). The sizes of the areas are not proportional to the magnitude of the numbers. **(d)** NJ tree including the 81 Chinese RMs derived from this study. The 26 captive Chinese RMs from Zhong et al. [7] are indicated by blue dot.

Given the large evolutionary similarity between macaques and humans, human diseases are better modeled in RMs than in many other animals. Thus, variants in RMs that match to orthologous human variants annotated as “pathogenic” are of particular interest. We examined presumed homologous Chinese RM SNPs in the human genome. A total of 34,850,330 RM SNPs analyzed in this study were successfully identified in the human genome (hg19). Among these SNPs, 118 [60] variants matched human variants with the accordant reference alleles, and alternative alleles were annotated as “disease causing” in HGMD or pathogenic in ClinVar. These 118 RM SNPs affect genes that cause specific human diseases including acromesomelic dysplasia maroteaux type, anonychia, atransferrinemia, blau syndrome, carcinoma of the colon, Charcot-Marie-Tooth disease, deafness, early infantile epileptic encephalopathy 7, glycogen storage disease, and others (Supplementary Table S12). Among these 118 SNPs, only 7 pathogenic SNPs are shared by all five subspecies, while 82 are subspecies specific (Fig. 4c, Supplementary Table S12). For example, the SNP rs116229331 in the gene *Unc13d* (human Chr17: 73836585C>T), known to cause juvenile idiopathic arthritis in humans [60], has an RM homologue (RM Chr16: 69559126 C>T, Fig. 4a) that is present in *M. m. tcheliensis, M. m. brevicaudus*, and *M. m. littoralis* but absent in *M. m. lasiotis* and *M. m. mulatta*. Another pathogenic variant (rs397514345, human Chr3: 15686724 A>C) in the *Btd* gene is involved in biotinidase deficiency [61]. Its homologous RM variant (RM Chr2: 172277927 A>C, Fig. 4a) is found only in *M. m. lasiotis* and *M. m. mulatta*. In addition, we also identified 16 nonsynonymous SNPs in the *Noca3* gene, which encodes a protein that modulates the replication and transcriptional reactivation of human immunodeficiency virus type 1 (HIV-1) during virus latency [62] (Fig. 4b). Ten of these 16 nonsynonymous SNPs are private to one subspecies (Supplementary Table S13). The effects of these variants on HIV-1 replication and reactivation are unknown and need further investigation, but the high number of mutations suggests a complex response of the host to the virus.

Overall, these findings suggest that the genomic architecture of Chinese RMs used in biomedical research and their geographic origin could strongly influence the outcome of biomedical experiments and should be taken into account when using Chinese RMs in clinical and neurobiological research. Unfortunately, genome-wide screening of RMs used in biomedical research is, to date, only rarely conducted, and uncharacterized animals are most often used. Importantly, individuals from all five Chinese RM subspecies are used in biomedical research [63, 64]. Combined with our data, 9 of the 26 captive Chinese RMs reported by Zhong et al. [7] were found to cluster with *M. m. littoralis*, 16 with *M. m. lasiotis*, and 1 with *M. m. mulatta* (Fig. 4d). Thus, the data and results presented here provide the basis to trace the origin of captive RMs and to allow for the selection of appropriate animal models when testing for particular diseases and are thus a significant contribution to the “3 Rs” principle, which aims to reduce, refine, and replace experimental animals [65].

## Conclusion

We present the first description of the evolutionary history and genomic variation of geo-referenced wild RMs throughout China, including scenarios on potential functions of this variation in adaptation to local environments. This genomic resource represents a valuable contribution to the understanding of the biology and evolution of a highly successful and important biomedical research species. In particular, it is important to note that due to the difference in evolutionary history of the subspecies identified here, it can be expected that animals originating from different regions may react differently to experimental tests, and thus their background needs to be assessed beforehand [10]. Our results highlight the importance that genome typing can play in biomedical research where animal origins are uncertain, and the resources generated here provide a baseline for genomic assessment of biomedical research populations and genetic resource conservation and for refined usage of RMs in future research.

## Materials and Methods

### Ethics statement

The methods were carried out in accordance with the approved guidelines of the Good Experimental Practices adopted by the Institute of Zoology, Chinese Academy of Sciences (CAS). All experimental procedures and animal collection were conducted under the supervision of the Committee for Animal Experiments of the Institute of Zoology (CAS).

### Sample collection and sequencing

Samples from 79 individuals with information about geographic origin were collected from 17 local wildlife rescue centers, which covered most of the species’ range in China. Muscle samples were collected from deceased individuals, and the blood samples were taken during routine physical examinations. Total genomic DNA was extracted from blood or tissue samples using standard phenol/chloroform methods. For each individual, ∼3 μg DNA was sheared into fragments of 500 bp with the Covaris system. DNA fragments were then processed and sequenced using the Illumina HiSeq 2000 and 2500 platforms. Furthermore, published genomic data for two individuals were downloaded from NCBI [9, 23] and filtered using the same conditions. Raw reads were first filtered with the following criteria: reads with unidentified nucleotides (N) exceeded 10% were discarded and reads with the proportion of low-quality base (phred quality ≤ 5) larger than 50% were discarded. After quality control, 3,095.6 Gb of high-quality sequences with 22.53 billion pair-end reads (100 or 125 bp) were generated.

### Sequence data preprocessing and variant calling

High-quality sequence reads were mapped to the macaque reference genome, Mmul_8.0.1 [66], using the Burrows–Wheeler Aligner 0.7.10-r789 (BWA, RRID:SCR_010910) [67]. Sequence Alignment/Map (SAM) format files were imported to SAMtools v0.1.19 (SAMtools, RRID:SCR_002105) [68] for sorting and then imported to Picard, version 1.118 (Picard, RRID:SCR_006525) [69] for removing duplicated reads. To improve the quality of sites reported, we performed SNP calling following GATK's best practice, version 3.3–0 (GATK, RRID:SCR_001876) on autosomal sites only [70]. We got the GVCF file for each individual using the “HaplotypeCaller” method in GATK and then using the GenotypeGVCFs-based method with the “-includeNonVariantSites” flag to get the population VCF file, including all the confident sites. After that, we applied the “SelectVariants” to exclude the Indel and split the variant and nonvariant sites. Then, we applied the hard filter command “VariantFiltration” to exclude potential false-positive variant calls with the following criteria: “-filterExpression QD < 5.0 ‖ FS > 60.0 ‖ MQ < 40.0 ‖ ReadPosRankSum< -8.0 || MQRankSum < -12.5” and “–genotypeFilterExpression DP < 4.0”. Additionally, the sites are filtered out if there is an “N” in the reference sequence or the site is fixed difference to the reference genome or the site including more than 20% missing genotypes. For nonvariant sites, we filtered the sites if there is an “N” in the reference sequence or if the site is including more than 20% missing genotypes. All the SNPs were annotated by ANNOVAR v2013–06-21 (ANNOVAR, RRID:SCR_012821) [71] (Supplementary Table S2). For each individual, the heterozygosity was calculated as the heterozygous SNP rate across the whole genome based on the whole number of sites that are callable (Supplementary Table S3).

### Genetic diversity and structure analysis

An NJ tree was constructed for the 81 individuals based on the autosomal genome data using the software TreeBeST. The bootstrap was set to 1,000 times to assess branch support, with the genome information of Indian RMs and *M. sylvanus* as outgroups. FigTree ([72], v1.4.0) was used to visualize the phylogenetic tree (Fig. 1b and Supplementary Fig. S4). Population structure analysis was performed using the software STRUCTURE 2.3.4 [25], which estimates individual ancestry and admixture proportions assuming *K* ancestral populations. We ran STRUCTURE five times to assess convergence and tested the number of genetic clusters (*K*) from 2–9 (Supplementary Fig. S5). We also carried out a PCA using the smartPCA program from the Eigensoft package, v5.0 (Eigensoft, RRID:SCR_004965) [73]. To determine the significance level of principal components, a Tracy-Widom test was done after the PCA (Supplementary Table S4). Decay of linkage disequilibrium against physical distance for the different populations was calculated using the Haploview software [74], with the maxdistance set as 500 kb (Supplementary Fig. S15).

### Demographic and divergence inference using PSMC and fastsimcoal2

We called the consensus sequences using Samtools mpileup [68] by applying: “samtools mpileup -q 1 -C 50 -S -D -m 2 -F 0.002 -u -f *.fa(genome) *.bam | bcftools view -c—| vcfutils.pl vcf2fq -d 10 -D 100 -Q 20 - > *.psmc.fq” and “fq2psmcfa -q10 -s 100 *.psmc.fq >*.psmc.fa.” To ensure the quality of consensus sequences, we used data of 10 individuals with an average coverage >20 × (22.20–34.32 ×). The PSMC model [26] was used to estimate the population histories from the individual genomes (sex chromosomes excluded) with the following parameters: –N30 –t15 –r5 –p “4+25 × 2+4+6.” We chose a generation length of 11 years and a mutation rate per generation (*μ*) of 1.0 × 10^−8^ (for the rationale to use these values, see [6, 75]).

We used PAUP* 4.0a142 (PAUP, RRID:SCR_014931) [30] to run SVDquartets to estimate the branching pattern among the five subspecies with the following command: SVDQuartets SpeciesTree = yes bootstrap evalQuartets = all seed = 0 nthreads = 40. The joint SFS approach implemented in *fastsimcoal2* [32] was performed to model more recent demographic fluctuations and respective divergence times based on the species tree estimation by SVDquartets. A VCF file containing callable variant sites was converted to fastsimcoal style folded SFS. To mitigate the effect of linkage disequilibrium, we filtered out the SNPs located within 10 kb from genes and then we took one SNP every 10 kb randomly. The multidimensional folded SFS for all five subspecies was generated with easySFS [76]. During the likelihood calculation, a conditional maximization algorithm (ECM) is used to maximize the likelihood of each parameter while keeping the others stabilized. This ECM procedure runs through 40 cycles where each composite likelihood was calculated using 100,000 coalescent simulations. Additionally, in order to avoid likelihood estimates that oversample parameter values at local maxima across the composite likelihood surface, we ran 50 replicates with each starting from different initial conditions. We chose the replicate with the highest estimated maximum likelihood score to estimate confidence intervals using parametric bootstrapping. The SFS used in the bootstrap was simulated with the parameter values from the highest likelihood model and then new parameter values were re-estimated from the simulated SFS. We ran 100 parametric bootstraps (Supplementary Fig. S7).

### Positive selection

To identify genomic regions that may have been subject to selection for each subspecies in different habitats, we scanned the genome using one-to-one pairwise comparisons between all five subspecies. We calculated the genome-wide distribution of *F*_ST_ values [77] and *θ*_π_ ratios for each pairwise comparison among five RM subspecies. We calculated *θ*_π_ for each population and the *F*_ST_ between the two populations in each comparison using VCFtools (VCFtools, RRID:SCR_001235) [78] with a genome-wide sliding window strategy (50 kb in length with 25-kb step). The *F*_ST_ values were Z-transformed, and the log value of *θ*_π_ ratio (*θ*_π_2/θ_π_1) was estimated. Candidate regions under positive selection were extracted based on the top 5% of log-odds ratios for both Z (*F*_ST_) and log (*θ*_π_ –ratio). Finally, for each subspecies, we used the intersection of putatively selected regions generated by all the pairwise comparisons with other subspecies as the candidate regions under positive selection (i.e., consistent signatures of selective sweeps). Genes located in these regions are expected to represent targets of selection. Functional classification and enrichment analysis of GO categories and KEGG pathways for these candidate genes were performed using DAVID v6.8 (DAVID, RRID:SCR_001881) [79]. The modified Fisher exact *P* value cutoff was 0.05. Chi-square and *P* values for the allele frequencies in *M. m. tcheliensis* vs *M. m. brevicaudus* for the resequenced SNPs from the candidate genes were assessed with the Haploview program [74].

### Genomic divergence and implication for biomedical research

A total of 118 of 52,534,348 RM SNPs analyzed in this study were successfully mapped to human reference sequence version hg19 (GRCh37) using liftOver [80] and were annotated as “disease causing” in HGMD (version 2015.1) or pathogenic in ClinVar (downloaded 25/02/2018) (Supplementary Table S12).

## Availability of supporting data

All data generated from this study have been submitted to the NCBI Sequence Read Archive under BioProject PRJNA345528. The datasets supporting the results of this article are available in the *GigaScience* GigaDB repository [81].

## Additional files

**Supplementary Fig. S1**. Variant number for the 81 individuals in this study.

**Supplementary Fig. S2**. Private and shared SNPs per Chinese RM subspecies.

**Supplementary Fig. S3**. Heterozygosity per base-pair for five subspecies of Chinese RMs.

**Supplementary Fig. S4**. Neighbor-joining tree derived from 1000 bootstrap replicates.

**Supplementary Fig. S5**. Plots of *ΔK* generated from STRUCTURE results.

**Supplementary Fig. S6**. The species tree based on SVDquartets+PAUP*.

**Supplementary Fig. S7**. Confidence Intervals from 100 parametric bootstraps for inferred demographic parameters.

**Supplementary Fig. S8**. Positive selection flow chart for each subspecies.

**Supplementary Fig. S9**. Distribution of Z(*F_ST_*) and *θ*_π_ log ratio of 50kb windows with 25kb sliding window for *M. m. tcheliensis*.

**Supplementary Fig. S10**. Distribution of Z(*F_ST_*) and *θ*_π_ log ratio of 50kb windows with 25kb sliding window for *M. m. brevicaudus*.

**Supplementary Fig. S11**. Distribution of Z(*F_ST_*) and *θ*_π_ log ratio of 50kb windows with 25kb sliding window for *M. m. littoralis*.

**Supplementary Fig. S12**. Distribution of Z(*F_ST_*) and *θ*_π_ log ratio of 50kb windows with 25kb sliding window for *M. m. lasiotis*.

**Supplementary Fig. S13**. Distribution of Z(*F_ST_*) and *θ*_π_ log ratio of 50kb windows with 25kb sliding window for *M. m. mulatta*.

**Supplementary Fig. S14**. Non-synonymous variants in putatively selected genes.

**Supplementary Fig. S15**. Linkage disequilibrium pattern of the five Chinese RM subspecies.

**Supplementary Table S1**. Overview of sample information and sequencing statistics.

**Supplementary Table S2**. Distribution of autosomal SNPs within various genomic regions of RM.

**Supplementary Table S3**. Identified SNPs and heterozygosity for 81 individuals from 17 sampling locations.

**Supplementary Table S4**. Tracy-Widom (*TW*) statistics and *P* values for the ten first eigenvalues in PCA.

**Supplementary Table S5**. Inferred demographic parameters with 95% confidence intervals for fastsimcoal2 model.

**Supplementary Table S6**. List of positively selected genes in the five Chinese RM subspecies.

**Supplementary Table S7**. Enrichment of genes under selective sweep in *M. m. tcheliensis*.

**Supplementary Table S8**. Morphological differences between the five investigated Chinese RM subspecies.

**Supplementary Table S9**. Enrichment of the genes under selective sweep in *M. m. brevicaudus*.

**Supplementary Table S10**. Distribution of SNPs in the selected genes described in the part of “Signatures of selection and local adaptation”.

**Supplementary Table S11**. Non-synonymous SNPs with significant differences at the 5% level in the distributions of genotypes between *M. m. tcheliensis* and *M. m. brevicaudus*.

**Supplementary Table S12**. List of RM variants scored by HGMD and ClinVar as “disease causing” or “pathogenic”.

**Supplementary Table S13**. Population study for *Ncoa3* reveals multiple genotypes.

## Abbreviations

BMP: bone morphogenetic protein CI: confidence interval GO: Gene Ontology HIV-1: human immunodeficiency virus type 1 I-SMAD: inhibitory small mothers against decapentaplegic KEGG: Kyoto Encyclopedia of Genes and Genomes kya: thousand years ago LG: Last Glaciation M: million NCBI: National Center for Biotechnology Information NJ: neighbor-joining PCA: principal component analysis PSMC: pairwise sequential Markovian coalescent RM: rhesus macaque SAM: Sequence Alignment/Map SFS: site frequency spectrum SNP: single-nucleotide polymorphism ssSNP: subspecies-specific single-nucleotide polymorphism

## Funding

This project was sponsored by grants to the following: M.L. (National Natural Science Foundation of China, 31530068 and 31821001; Strategic Priority Research Program of the Chinese Academy of Sciences, XDB31000000 and XDA19050202; National Key R&D Program of China, 2016YFC0503200; and Science & Technology Department of Sichuan Province, 2018JZ0008); Z.L. (Natural Science Foundation of China, 31471989).

## Competing interests

The authors declare that they have no competing interests.

## Author contributions

M.L., Z.L., and M.B. conceived the study and designed the project. Z.L., X.T., P.O., X.Z., L.Z., and S.T. managed the project, performed the analyses, and wrote the manuscript. Z.L., B.S., and H.X. prepared samples. Z.L., X.T., and P.O. performed genetic analyses. Z.L., X.T., P.O., B.R., L.Z., G.L., Z.Y., Z.P., Z.X., C.R., M.B., and M.L. discussed the data. Z.L. and X.T. wrote the manuscript with contributions from P.O., B.W., H.X., W.Z., C.R., M.B., and M.L.. All authors contributed to data interpretation.

## Supplementary Material

GIGA-D-17-00291_Original_Submission.pdf

GIGA-D-17-00291_Revision_1.pdf

GIGA-D-17-00291_Revision_2.pdf

Response_to_Reviewer_Comments_Original_Submission.pdf

Response_to_Reviewer_Comments_Revision_1.pdf

Reviewer_1_Report_(Original_Submission) -- Jeffrey Rogers 12/27/2017 Reviewed

Reviewer_1_Report_(Revision_1) -- Jeffrey Rogers 04/19/2018 Reviewed

Reviewer_2_Report_(Original_Submission) -- Alexander Nater 01/04/2018 Reviewed

Reviewer_2_Report_(Revision_1) -- Alexander Nater 04/15/2018 Reviewed

Reviewer_2_Report_(Revision_2) -- Alexander Nater 07/12/2018 Reviewed

Supplement Files
