# Supplementary material for: Population genomics of wild Chinese rhesus macaques reveals a dynamic demographic history and local adaptation, with implications for biomedical research
Source: Gigascience. 2018 Aug 27;7(9):giy106. doi: 10.1093/gigascience/giy106 (PMC6143732; doi:10.1093/gigascience/giy106)
Supplement: GIGA-D-17-00291_Original_Submission.pdf [file giy106_giga-d-17-00291_original_submission.pdf]

GigaScience

Population genomics of wild Chinese rhesus macaques reveals a dynamic demographic history and local adaptation, with implications for biomedical research

--Manuscript Draft--

**Manuscript Number:**	GIGA-D-17-00291
**Full Title:**	Population genomics of wild Chinese rhesus macaques reveals a dynamic demographic history and local adaptation, with implications for biomedical research
**Article Type:**	Research
**Funding Information:**	Strategic Priority Research Program of the Chinese Academy of Sciences (XDPB0202) — Dr Ming Li<br>NSFC (31530068) — Dr Ming Li<br>NSFC (31471989) — PhD Zhijin Liu<br>National Key R&D Program of China (2016YFC0503200) — Dr Ming Li
**Abstract:**	The rhesus macaque (RM, Macaca mulatta) is the most important nonhuman primate model in evolutionary biology and biomedical research. We present the first population genomics survey of wild RMs, comprising 81 geo-referenced individuals representing five subspecies from 17 locations in China, covering a large fraction of the species' natural distribution. Genetic diversity, measured with a total of 55.4 M autosomal single nucleotide polymorphisms (SNPs), is higher in wild RMs than captive populations. We find a hierarchical population structure with four distinct genetic lineages found on the mainland and one on Hainan Island recapitulating current subspecies designations. The five subspecies are estimated to have diverged between 140 and 72 thousand years ago, but with recent gene flow among some groups. Consistent with the expectation of a larger body size in colder climates (Bergman's rule), the northernmost RM lineage (subspecies, M. m. tcheliensis) exhibits the largest body size of all Chinese RMs and was featured with positively selected genes responsible for skeletal development. The tropical subspecies M. m. brevicaudus was characterized by positively selected genes related to cardiovascular function and response to temperature stimuli, which are potentially involved in adaptation to tropical climates. We further delineated 111 RM SNPs matching human disease-causing variants with 74 being subspecies-specific. The data presented herein provides a reference resource for the choice of sub-group of RMs when carrying out biomedical experiments. The unexpected demographic history of Chinese RMs, coupled with their history of local adaption offers new insights into the evolution of RMs and provides valuable baseline information for biomedical research.
**Corresponding Author:**	Ming Li<br><br>CHINA
**Corresponding Author Secondary Information:**	
**Corresponding Author's Institution:**	
**Corresponding Author's Secondary Institution:**	
**First Author:**	Zhijin Liu
**First Author Secondary Information:**	
**Order of Authors:**	Zhijin Liu<br>Xinxin Tan<br>Pablo Orozco-terWengel<br>Xuming Zhou

Powered by Editorial Manager® and ProduXion Manager® from Aries Systems Corporation

	Shilin Tian
	Liye Zhang
	Guangjian Liu
	Zhongze Yan
	Huailiang Xu
	Boshi Wang
	Baoping Ren
	Peng Zhang
	Zuofu Xiang
	Binghua Sun
	Christian Roos
	Michael W. Bruford
	Ming Li
**Order of Authors Secondary Information:**	
**Opposed Reviewers:**	
**Additional Information:**	
**Question**	**Response**
Are you submitting this manuscript to a special series or article collection?	No
**Experimental design and statistics**<br><br>Full details of the experimental design and statistical methods used should be given in the Methods section, as detailed in our Minimum Standards Reporting Checklist. Information essential to interpreting the data presented should be made available in the figure legends.<br><br>Have you included all the information requested in your manuscript?	Yes
**Resources**<br><br>A description of all resources used, including antibodies, cell lines, animals and software tools, with enough information to allow them to be uniquely identified, should be included in the Methods section. Authors are strongly encouraged to cite Research Resource Identifiers (RRIDs) for antibodies, model organisms and tools, where possible.<br><br>Have you included the information requested as detailed in our Minimum Standards Reporting Checklist?	Yes

Powered by Editorial Manager® and ProduXion Manager® from Aries Systems Corporation

**Availability of data and materials**<br><br>All datasets and code on which the conclusions of the paper rely must be either included in your submission or deposited in publicly available repositories (where available and ethically appropriate), referencing such data using a unique identifier in the references and in the “Availability of Data and Materials” section of your manuscript.<br><br>Have you have met the above requirement as detailed in our Minimum Standards Reporting Checklist?	Yes

Manuscript Click here to download Manuscript renamed_7afc7.docx

Title: Population genomics of wild Chinese rhesus macaques reveals a dynamic demographic history and local adaptation, with implications for biomedical research

Running Title: Population genomics of wild rhesus macaques

Zhijin Liu[1, †], Xinxin Tan[1, 2, 3, †], Pablo Orozco-terWengel[4, †], Xuming Zhou[5, †], Shilin Tian[6, 7, †], Liye Zhang[1, 2], Guangjian Liu[6], Zhongze Yan[1, 3], Huailiang Xu[7], Boshi Wang[1], Baoping Ren[1], Peng Zhang[8], Zuofu Xiang[9], Binghua Sun[10], Christian Roos[11, *], Michael W. Bruford[4, *], Ming Li[1, *]

[1] Key Laboratory of Animal Ecology and Conservation Biology, Institute of Zoology, Chinese Academy of Sciences, Beijing, China.

[2] University of Chinese Academy of Sciences, Beijing 100039, China.

[3] Institute of Health Sciences, Anhui University, Hefei, 230601, China.

[4] School of Biosciences, Cardiff University, Sir Martin Evans Building, Museum Avenue, Cardiff CF10 3AX, United Kingdom.

[5] Division of Genetics, Department of Medicine, Brigham and Women's Hospital, Harvard Medical School, Boston, MA 02115, USA.

[6] Novogene Bioinformatics Institute, Beijing 100083, China.

[7] College of Life Science, Sichuan Agricultural University, Ya'an 625014, China.

[8] School of Sociology and Anthropology, Sun Yat-sen University, Guang Zhou, China.

[9] College of Life Science and Technology, Central South University of Forestry and Technology, Changsha 410004, Hunan, China.

[10] School of Life Sciences, Anhui University, Hefei, 230601, China.

[11] Gene Bank of Primates and Primate Genetics Laboratory, German Primate Center, Leibniz Institute for Primate Research, Kellnerweg 4, 37077 Göttingen, Germany.

† Contributed equally

***** Correspondence: Ming Li, lim@ioz.ac.cn; Michael W. Bruford, BrufordMW@cardiff.ac.uk; Christian Roos, CRoos@dpz.eu

Abstract

The rhesus macaque (RM, *Macaca mulatta*) is the most important nonhuman primate model in evolutionary biology and biomedical research. We present the first population genomics survey of wild RMs, comprising 81 geo-referenced individuals representing five subspecies from 17 locations in China, covering a large fraction of the species' natural distribution. Genetic diversity, measured with a total of 55.4 M autosomal single nucleotide polymorphisms (SNPs), is higher in wild RMs than captive populations. We find a hierarchical population structure with four distinct genetic lineages found on the mainland and one on Hainan Island recapitulating current subspecies designations. The five subspecies are estimated to have diverged between 140 and 72 thousand years ago, but with recent gene flow among some groups. Consistent with the expectation of a larger body size in colder climates (Bergman's rule), the northernmost RM lineage (subspecies, *M. m. tcheliensis*) exhibits the largest body size of all Chinese RMs and was featured with positively selected genes responsible for skeletal development. The tropical subspecies *M. m. brevicaudus* was characterized by positively selected genes related to cardiovascular function and response to temperature stimuli, which are potentially involved in adaptation to tropical climates. We further delineated 111 RM SNPs matching human disease-causing variants with 74 being subspecies-specific. The data presented herein provides a reference resource for the choice of sub-group of RMs when carrying out biomedical experiments. The unexpected demographic history of Chinese RMs, coupled with their history of local adaption offers new insights into the evolution of RMs and provides valuable baseline information for biomedical research.

Keywords: *Macaca mulatta*, population genomics, adaptive selection, biomedical model

Introduction

Understanding how species evolve and adapt to their environments is an essential question in evolutionary biology. Rhesus macaques (RMs, *Macaca mulatta*) are, after humans, the world's most successful primates [1-5], occupying a vast geographic distribution spanning from Afghanistan to the Chinese shore of the Pacific Ocean and south into Myanmar, Thailand, Laos and Vietnam [5]. As the most widely distributed nonhuman primate species, RMs occupy diverse ecological landscapes and habitats, making them an interesting model to address questions about how species evolve and adapt to local environmental variation, including characterizing the genomic architecture of adaptation to habitat, climate and other biotic and abiotic factors. Yet, despite much work on primate comparative genomics, very few population genomic studies have been carried out on wild RMs [6, 7]. Importantly, as RMs are widely used as a primate model in physiological, psychological and cognitive studies [8-10], knowledge about their genomic architecture could improve and refine biomedical research [10] as the genomic composition of experimental animals can have a considerable influence on the outcome of experiments [11, 12]. Therefore, information on the genomic diversity not only of captive, but also of wild RMs, that could become a genomic resource for future utilization in medical research, is essential.

In biomedical research, two main populations (Indian and Chinese) are recognized [6, 13]. They diverged from each other at ~162 thousand years ago (kya) and are characterized by extensive differences in morphology, behavior, ecology, physiology, reproduction, and disease progression [6, 13-19]. In 1978 India banned all RM exports to breeding centers across the world, thus curtailing the availability of wild Indian RMs and subsequently increasing the demand for Chinese RMs in biomedical research, thereby making a detailed characterization of genetic variants from Chinese RMs crucial for biomedical usage of this species.

To date, the genomes of 133 captive RMs from eight colonies have been sequenced, however, 124 of them are of Indian-origin and only nine individuals were presumed to be of Chinese origin [6]. Recently, Zhong *et al.* [7] reported genomic variation in 26 Chinese captive RMs identifying ~46 M (million) single nucleotide polymorphisms (SNPs). Nevertheless, most of these RM genetic variation is limited to captive populations which may contain composite genotypes due of admixture among animals of different and unclear origin [20]. Here we present the first attempt to survey the

geo-referenced genomic diversity in wild Chinese RM populations, which is the largest extant population of the species. The current effective population size of Chinese and Indian RM was estimated to be approximate 240,000 and 17,000 individuals, respectively, indicating that the Chinese RMs are likely to harbor substantially more genomic diversity compared to their Indian conspecifics [13]. Therefore, this population genomic survey of 81 RMs originating from 17 wild locations across China including phylogenetic, demographic and genome-wide selection scans, corresponds to the most comprehensive characterization of RM genetic diversity to date and aimed at characterizing the processes leading to the extant patterns of variability, as well as identifying the potential implications for the use of these populations in biomedical research.

Results and Discussion

Genetic diversity, phylogeny and population structure

Blood and tissue samples from 79 wild-born RMs, representing five subspecies [21, 22], were collected at 17 sites in China (*M. m. tcheliensis*: TH; *M. m. littoralis*: AH, FJ, HB, GX, GZ; *M. m. brevicaudus*: HN; *M. m. lasiotis*: SX, SC1, SC2, SC3, SC4; *M. m. mulatta*: YN1, YN2, YN3, YN4, YN5; Fig. 1a). Genome sequences of two additional Chinese RMs (CR1 and CR2) were retrieved from NCBI [9, 23, 24]. Re-sequencing was at a high average depth of 28.26±4.75× for ten individuals and a moderate average depth of 10.64±1.16× for the remainder (n=71), with an overall average genome coverage of 95.43% of the RM reference (rheMac2, Supplementary Table 1). A total of 55,404,179 SNPs were identified and used for downstream analyses (Supplementary Table 2), with wild RMs carrying on average over 20% more genomic variation than captive individuals (43.7 M SNPs detected in 31 Indian and Chinese captive RMs and 46.1 M SNPs found in 133 Indian and Chinese captive RMs)[6,7]. The number of SNPs per individual ranged from 7.3 to 11.6 M (mean of 9.5 M; Supplementary Fig. 1 and Supplementary Table 3). Among Chinese RM subspecies, *M. m. mulatta* had the highest heterozygosity (0.202%$\pm1.09\times10^{-4}$), followed by *M. m. littoralis* (0.180%$\pm1.55\times10^{-4}$) and *M. m. lasiotis* (0.178%$\pm1.47\times10^{-4}$). The lowest heterozygosity rates were found in *M. m. brevicaudus* (0.157%$\pm1.17\times10^{-4}$) and *M. m. tcheliensis* (0.135%$\pm3.07\times10^{-4}$) (Supplementary Fig. 2). Among all detected SNPs, 7,575,099 were shared among all subspecies and 23,676,191 were shared by at least two subspecies, with the remaining SNPs confined to a single subspecies (Supplementary Fig. 3a). For each subspecies, the subspecies-specific SNPs (ssSNPs) ranged from 834,655 to 8,507,232 and the non-synonymous ssSNPs varied from 3,723 to 27,537 (Supplementary Fig. 3a, b).

We reconstructed a neighbor-joining (NJ) tree for Chinese RMs based on autosomal SNPs, using Indian RMs and *M. sylvanus* as outgroups (Fig. 1b and Supplementary Fig. 4). Individuals from *M. m. lasiotis*, *M. m. brevicaudus* and *M. m. tcheliensis* form monophyletic lineages respectively, while *M. m. mulatta* and *M. m. littoralis* are paraphyletic. The divergence among Chinese RMs started with the successive splitting of the *M. m. mulatta* lineages, followed by *M. m. lasiotis*, before the eastern subspecies differentiated. Among the latter, *M. m. brevicaudus* and *M. m. tcheliensis* diverged from *M. m. littoralis*, respectively. Next, we performed a population

structure analysis using STRUCTURE (version 2.3.4) [25], which estimates individual ancestry and admixture structure analysis proportions assuming *K* ancestral populations. Plots of *ΔK* generated from STRUCTURE results indicated five genetic clusters present in the full data set (Fig. 1b and Supplementary Fig. 5). A principal component analysis (PCA) corroborated the division of Chinese RMs into five groups. The first eigenvector separated *M. m. mulatta* and *M. m. lasiotis* from *M. m. tcheliensis*, *M. m. littoralis* and *M. m. brevicaudus* (variance explained = 6.40%, Tracy-Widom $P = 4.29\times10^{-42}$), and the second eigenvector further separated *M. m. tcheliensis*, *M. m. littoralis* and *M. m. brevicaudus* (variance explained = 5.07%, Tracy-Widom $P = 5.29\times10^{-22}$) (Fig. 1c, Supplementary Table 4). The division of Chinese RMs into five geographic lineages supports the former taxonomic division of Chinese RMs into five subspecies [21, 22]. *M. m. mulatta* (YN1-5) and *M. m. lasiotis* (SC1-4, SX) form the pan-western populations of Chinese RMs, with both subspecies inhabiting the montane Tibetan Plateau regions with an altitude ≥1500 meters above sea level in western China and separated from each other by the Yangtze River. *M. m. littoralis* (AH, FJ, HB, GX, GZ), *M. m. tcheliensis* (TH) and *M. m. brevicaudus* (HN) occur in the eastern coastal lowland of China and form the pan-eastern population. *M. m. tcheliensis* from the Taihang Mountains area (TH) is the northernmost (34°54'–35°16' N; 112°02'–112°52' E), while *M. m. brevicaudus*, restricted to Hainan Island, is the most southern Chinese RM subspecies.

Demographic and phylogeographic history

We applied the pairwise sequential Markovian coalescent (PSMC) [26] using ten RM individuals with an average sequencing coverage depth higher than 20× (one individual of *M. m. tcheliensis* and one of *M. m. brevicaudus*, two of *M. m. lasiotis*, three of *M. m. littoralis* as well as three individuals of *M. m. mulatta*) to infer the ancient demographic history of Chinese RMs. The inferred PSMC trajectories were very similar for all analyzed individuals throughout most of the species' history until ~110 kya reflecting the species' cohesiveness (Fig. 2a). The ancient demographic history of RMs is marked by population fluctuations following the glacial periods during the Pleistocene [27]. Approximately 800-500 kya all Chinese RMs experienced a population reduction at the time of the Naynayxungla Glaciation (NG, 780-500 kya), followed by an expansion during the Mid-Pleistocene inter-glaciation (500-200 kya). This expansion was then interrupted by the Penultimate Glaciation (PG, 200-130 kya) when suitable habitat might have been lost leading to a population decline [27].

PSMC analyses also suggested that while *M. m. mulatta* and *M. m. lasiotis* stabilized with effective population sizes somewhere around 110 kya, *M. m. tcheliensis*, *M. m. littoralis* and *M. m. brevicaudus* went through a dramatic population increase and a subsequent bottleneck reaching a stable effective population size somewhere around 50-60 kya (Fig. 2a).

Due to the observed divergence of the five Chinese RM subspecies, we further employed the joint site frequency spectrum (SFS) approach to model scenarios that could explain the observed population structure, as well as respective divergence times between the five RM lineages. These analyses, carried out using *fastsimcoal2* [28] produced a significantly better fit of a step by step divergence scenario than alternative ones (Supplementary Tables 5 and 6, Supplementary Fig. 6), and support the demographic changes observed with the PSMC analyses. Under this model, following the divergence between the ancestral lineages of the Indian and Chinese RM (~162 kya), the ancestor of the *M. m. mulatta* lineage diverged form that of the remaining Chinese RMs ~140.2 kya near the end of the last interglacial (Fig. 2b) [6, 13]. This divergence was associated with a mild decrease in effective population size (*Ne*) of *M. m. mulatta* with respect to its ancestor. Subsequently, *M. m. lasiotis* diverged from the ancestral lineage of pan-eastern RM ~107.1 kya, undergoing a similar reduction in *Ne* as *M. m. mullata*. Contrastingly, this divergence was followed by an almost doubling of the ancestral effective population size (N_{A2} =232.5k) of the ancestral pan-eastern RM lineage (Fig 2b). The divergence time among *M. m. tcheliensis*, *M. m. littoralis* and *M. m. brevicaudus* was estimated to occurred ~71.7 kya, at the start of the period leading to the last glacial maximum (fig 2b) [29, 30], and coinciding with a drastic decrease in *Ne* in these three lineages. Gene flow after the divergence of subspecies occurred among almost all five lineages.

A previous study of mitochondrial DNA identified two major haplogroups dividing Chinese RMs in a western and an eastern clade, and with modern Chinese RMs thought to have undergone a northward expansion while entering China via two possible routes: the first into the western mountains and the second following the eastern coast [31]. Our evolutionary model, however, suggests a “step-by-step” colonization process of RMs into China (Fig 2c). After the divergence from the Indian population (~162 kya) [6, 13], the ancestor of Chinese RMs colonized the Tibetan Plateau from southwestern China, and then experienced a range expansion north and eastwards. The pan-western population (*M. m. mulatta* and *M. m. lasiotis*) inhabited the western montane region in China, while the pan-eastern population (*M. m. tcheliensis*, *M. m. littoralis* and *M. m. brevicaudus*)

entered the eastern coastal region. Barriers such as the Yellow, Yangtze and Pearl rivers and open sea (Fig. 1a) led to further differentiation, limiting gene flow among them. Water bodies and mountains could therefore be described as driving the formation of a habitat 'lattice' with the different subspecies of RMs occupying different grids in the lattice.

Signatures of selection and local adaptation

The wide distribution of Chinese RMs and their respective contrasting habitat types, as well as their wide use in biomedical studies, makes them an important case study for the analysis of signatures of local adaptation to divergent selective pressures [32-34]. We identified putative targets of selection by carrying out pair-wise comparisons between RM subspecies inhabiting the most different environments to increase the chance of finding selection signatures, i.e., *M. m. tcheliensis* that lives in the northernmost range of the species in the coldest environments, and *M. m. brevicaudus* that inhabits the southernmost range of the species in a tropical environment. For each analysis, we compared the five subspecies using the fixation index (F_{ST}) and genetic diversity (θ_π), calculated on 50kb long sliding windows (Fig. 3 and Supplementary Figs. 7-12). The top 5% of the windows with the largest F_{ST} and θ_π ratios ($\theta_\pi 2$ /$\theta_\pi 1$) in each pair-wise comparison were considered to be potentially under positive selection. For each subspecies, we identified the intersection of potential selective-sweep regions generated by all the pair-wise comparisons between a subspecies and each of the other subspecies (four pairwise comparisons in each case) (Supplementary Fig. 7). We used these consistent selective-sweep regions for further analyses, as they represent robust putative positively selected regions. The sizes of candidate selective-sweep regions ranged from 0.275 Mb to 8.575 Mb and the number of genes located in these regions, which are expected to represent targets of selection for each subspecies, varied from 8 to 141 in different subspecies (Supplementary Table 7).

M. m. tcheliensis from the Taihang (TH) Mountains area is the northernmost population of the species. The TH mountain are characterized by a continental monsoon climate, and conditions for RMs are harsh during winter and early spring with average temperatures of –20°C. According to Bergman's rule, animals living in cold climates tend to have larger body sizes compared to their relatives in warm climates (i.e. they have a lower surface area to volume ratio), so they radiate less body heat per unit of mass [35]. Consistent with this expectation, of all RM subspecies, *M. m.*

tcheliensis exhibits the largest body size and mass, the shortest tail length, the longest forearm length and the largest head and chest circumference (Fig. 3b and Supplementary Table 8) [36,37]. Among the consistent signatures of positive selection identified in *M. m. tcheliensis* (128 genes), we found signatures of selective sweeps in 14 genes linked to limb morphogenesis and bone development (Supplementary Table 7), which present two highly enriched functional categories, "embryonic hind-limb morphogenesis" (three genes, modified Fisher Exact P=1.59×10^{-2}) and i.e. "bone development" (three genes, modified Fisher Exact P=3.40×10^{-2}) (Supplementary Table 9). Among these genes, *Papss2* is known to affect the development of the skeletal system in mouse and human and *Papss2* mutations could cause brachyolmia [38,39], while *Sox5* (Fig. 3c, d) plays an essential role in synovial joint morphogenesis via promoting both growth plate and articular chondrocyte differentiation [40]. *Bcl2* has been shown to regulate chondrocyte maturation during skeletal development and could influence long bone length [41]. These genes involved in the growth and development of the skeletal system and appendages are likely contributors to the larger body size of *M. m. tcheliensis,* and represent an undescribed adaptive pathway for primates living in colder climates.

In contrast, *M. m. brevicaudus* inhabits the tropical island of Hainan (HN) where it copes with a mean annual temperature of 24°C. We found 141 putatively selected genes in *M. m. brevicaudus* (Fig. 3c, d, Supplementary Table 7), seven of which were found in gene ontology (GO) terms related to cardiovascular system and blood circulation. For example, *Ppp3cb* related to GO term "heart development" and *Ctnna3* related to GO term "regulation of heart rate by cardiac conduction" [42]. In addition, *Camk2g* and *Ero1a* are directly involved in the GO terms "regulation of cellular response to heat" and "response to temperature stimulus". We thus hypothesize that the cardiovascular system of *M. m. brevicaudus* might play an important role in stabilizing body temperature, assisted by blood flow through different body parts requiring good fluidity and vascular permeability to transfer heat out of the body [43]. Test of these hypothesis needs further functional assays, however, these genes, together with the positively selected genes identified in *M. m. tcheliensis*, are known to be relevant to human physical function, and thus are likely of importance in the adaptation of Chinese RMs to different climates.

Besides the genes related to the adaptation to various climate conditions, we also found positive selection in genes related to the nervous system. In *M. m. littoralis* three of the 104 identified

candidate genes are enriched in GO term “regulation of synaptic plasticity” (modified Fisher Exact *P*=1.38E-02; Supplementary Table 10) and four genes are enriched in the KEGG pathway “serotonergic synapse” (modified Fisher Exact *P*=2.11E-02; Supplementary Table 10). In *M. m. tcheliensis* twelve putatively selected genes (Supplementary Table 7) are involved in the process of neuron morphogenesis and synaptic transmission, and one of these gene, *Clstn2* is the synaptic protein and reported to play an important role in learning and memory [44]. For *M. m. brevicaudus,* seven putatively selected genes related to nervous system development were found. For example, *Dcc* is reported to be required for long-term potentiation and memory [45]. *Auts2,* one of the eight putatively selected genes in *M. m. mulatta,* has been shown to regulate neuronal migration, and mutations in this gene cause mental dysfunction in human [46] (Supplementary Table 7). Our findings suggest that RM subspecies have experienced different adaptive processes in the nervous system and respective genomic differences should be taken into account when animals are selected for neurobiological research.

Disease–causing variants and implication for biomedical research

Given the large evolutionary similarity between macaques and humans, human diseases are better modeled in RMs than in many other animals. Thus, variants in RMs that match to orthologous human variants annotated as ‘pathogenic’ are of particular interest. We examined presumed homologous Chinese RM SNPs in the human genome and a total of 32,845,501 RM SNPs analyzed in this study were successfully identified in the human genome (hg19). Among these SNPs, 111 variants matched human variants with the accordant reference alleles and alternative alleles were annotated as ‘disease causing’ in HGMD or pathogenic in ClinVar. Those 111 RM SNPs affect genes that cause specific human diseases including acromesomelic dysplasia maroteaux type, anonychia, atransferrinemia, blau syndrome, Carcinoma of colon, Charcot-Marie-Tooth disease, deafness, early infantile epileptic encephalopathy 7, glycogen storage disease and others (Supplementary Table 11). Thirty-nine out of these 111 SNPs were identified in previous studies [6], while the remaining 72 SNPs are newly described here. Only seven pathogenic SNPs are shared by all five subspecies, while 74 are subspecies-specific (Fig. 4c, Supplementary Table 11). For example, the SNP rs116229331 in the *Unc13d* gene (human Chr17: 73836585C>T), known to cause juvenile idiopathic arthritis in humans [47], has a RM homologue (RM Chr16: 71160253C>T, Fig. 4a) that

is present in *M. m. tcheliensis, M. m. brevicaudus* and *M. m. littoralis*, but absent in *M. m. lasiotis* and *M. m. mulatta*. Another pathogenic variant (rs397514345, human Chr3: 15686724 A>C) in the *Btd* gene is involved in biotinidase deficiency [48]. Its homologous RM variant (RM Chr2: 157981062 A>C, Fig. 4a) is found only in *M. m. lasiotis* and *M. m. mulatta.* In addition, we also identified 16 non-synonymous SNPs in the *Noca3* gene, which encodes a protein that modulates the replication and transcriptional reactivation of HIV-1 during virus latency [49] (Fig. 4b). Ten of these 16 non-synonymous SNPs are private to one subspecies (Supplementary Table 12). The effects of these variants on HIV-1 replication and reactivation are unknown and need further investigation, but the high number of mutations suggests a complex response of the host to the virus.

Overall, these findings suggest that the genomic architecture of Chinese RMs used in biomedical research and their geographic origin could strongly influence the outcome of biomedical experiments and should be taken into account when using Chinese RMs in clinical and neurobiological research. Unfortunately, genome wide screening of RMs used in biomedical research is so far only rarely conducted and uncharacterized animals are most often used. Importantly, individuals from all five Chinese RM subspecies are used in biomedical research [50, 51]. Combined with our data, nine of the 26 captive Chinese RMs reported by Zhong *et al.* [7] were found to cluster with *M. m. littoralis*, 16 with *M. m. lasiotis* and one with *M. m. mulatta* (Fig. 4d). Thus, the data and results presented here provide the base date for tracing the origin of captive RMs and the basis for the selection of appropriate animal models when testing for particular diseases, and are thus a significant contribution to the “3Rs” principle, which aim to reduce, refine, and replace experimental animals.

Conclusion

We present the first description of the evolutionary history and genomic variation of geo-referenced wild RMs throughout China, including scenarios on potential functions of this variation in adaptation to local environments. This genomic resource represents a valuable contribution to the understanding of the biology and evolution of a highly successful and important biomedical research species. In particular, it is important to note that due to the difference in evolutionary history of the subspecies identified here, it can be expected that animals originating from different regions may react differently to experimental tests, and thus their background needs to be assessed beforehand [10]. Our results highlight the importance that genome typing can play in biomedical research where animal origins are uncertain, and the resources generated here provide a baseline for genomic assessment of biomedical research populations, genetic resource conservation and for refined usage of RMs in future research.

Materials and Methods

Ethics statement

The methods were carried out in accordance with the approved guidelines of the Good Experimental Practices adopted by the Institute of Zoology, Chinese Academy of Sciences (CAS). All experimental procedures and animal collection were conducted under the supervision of the Committee for Animal Experiments of the Institute of Zoology, Chinese Academy of Sciences.

Sample Collection and Sequencing

Samples from 79 individuals with information about geographic origin were collected from 17 local wildlife rescue center, which covered most of the species' range in China. Muscle samples were collected from deceased individuals and the blood samples were taken during routine physical examinations. Total genomic DNA was extracted from blood or tissue samples using standard phenol/chloroform methods. For each individual, ~3 μg DNA was sheared into fragments of 500 bp with the Covaris system. DNA fragments were then processed and sequenced using the Illumina HiSeq 2000 and 2500 platform. Raw reads were first filtered with the following criteria: (1) reads with unidentified nucleotides (N) exceeded 10% were discarded, (2) reads with the proportion of low quality base (phred quality <=5) larger than 50% were discarded. After the quality control, a total of 2,736.91 Gb of high quality sequences with 22.53 billion pair-end reads (100 or 125 bp) were generated. Furthermore, published genomic data for two individuals were download form NCBI [9,23] and filtered using the same conditions.

Sequence Data Pre–processing and Variant Calling

High-quality sequence reads were mapped to the macaque reference genome, rheMac2 [52], using the Burrows–Wheeler Aligner (BWA) (0.7.10-r789) [53]. Sequence Alignment/Map (SAM) format files were imported to SAMtools (v0.1.19) [54] for sorting and removing duplicated reads. Following mapping, we performed SNP calling using SAMTools on autosomal sites only. To obtain high-quality SNPs, we applied the calling protocol used in Chen et al [55]. The variants were filtered unless the minimum root-mean-square (RMS) mapping quality was 20. And then variants were removed if their average Phred scaled base quality was lower than 20 or the distance between the SNP was less than 5bp. Furthermore, only the variants supported by at least four reads were

presented for the subsequent analysis. Using SAMTools we discovered 55,404,179 SNPs on the autosomes of 81 Chinese RMs. Finally, all the SNPs were annotated by ANNOVAR (v2013-06-21) [56] (Supplementary Table 2). For each individual the heterozygosity was calculated as heterozygous SNP rate across the whole genome (Supplementary Table 3).

Genetic Diversity and Structure Analysis

A neighbor–jointing (NJ) tree was constructed for the 81 individuals based on the autosomal genome data using the software TreeBeST. The bootstrap was set to 1,000 times to assess branch reliability, with the genome information of Indian RMs and *M. sylvanus* as outgroups. FigTree (http://tree.bio.ed.ac.uk/software/figtree/, v1.4.0) was used to visualize the phylogenetic tree (Fig. 1b). Population structure analysis was performed using the software STRUCTURE 2.3.4 [27], which estimates individual ancestry and admixture proportions assuming *K* ancestral populations. We ran STRUCTURE five times to assess convergence and tested the number of genetic clusters (*K*) from 2-9 (Supplementary Fig. 5). We also carried out a principle component analysis (PCA) using the smartPCA program from the Eigensoft package (v5.0) [57]. To determine the significance level of principal components, a Tracy-Widom test was done after the PCA (Supplementary Table 4). Linkage disequilibrium for the different populations was calculated using the haploview software [58] with the maxdistance set as 500kb (Supplementary Fig. 13).

Demographic and Divergence Inference Using PSMC and Fastsimcoal2

The PSMC model [24] was used to estimate the population histories from the individual genomes (sex chromosomes excluded) with the following parameters: –N25 –t15 –r5 –p '4+25×2+4+6'. We assumed a generation length of 11 years and a mutation rate per generation (μ) of 1.0×10^{-8} [6]. To ensure the quality of consensus sequences, we used data of ten individuals with an average coverage >20× (22.20-34.32×).

Due to the limitation of PSMC inference for recent dating, we performed the joint site frequency spectrum (SFS) approach implemented in *fastsimcoal2* [25] to simulate more recent demographic fluctuations and respective divergence times. For the five identified RM subspecies, eight alternative divergence scenarios describing the evolutionary relationships of these subspecies were tested against each other to identify the one that best supports the observed data (Supplementary Fig. 6). The parameters used in fastsimcoal2 were: -N 100000 (max. number of

simulations), -L 40 (max. number of EM cycles), - M 0.001 (min. relative difference in parameter values for the stopping criterion). Multiple replicates with each model starting from different initial conditions were run to ensure convergence. The best model was addressed through the maximum value of the likelihoods and the Akaike information criterion [25]. Among all the scenarios tested, the highest lnL and lowest AIC value was generated under the scenario 2 (Supplementary Table 5).

Based on the best model (model 2) identified in the previous step, a more detailed scenario was tested including additional population parameters of interest such as effective population sizes, migration rates. Migration rates were ignored between subspecies which have no direct connection. The outputs of this scenario were processed with arlsumstat to obtain distributions of various summary statistics (Supplementary Table 6).

Positive Selection

To identify genomic regions that may have been subject to selection for each subspecies inhabited in different habitats, we scanned the genome using one-to-one pair-wise comparisons between all five subspecies. For each pairwise comparison, the differences in genetic diversity between two subspecies were reflected by pairwise nucleotide diversity (θ_π) and the divergence in allele frequency in two subspecies was quantified by pairwise F_{ST}. We calculated θ_π for each population and the F_{ST} between the two populations in each comparison using VCFtools [59] with a genome-wide sliding window strategy (50-kb in length with 25-kb step). The F_{ST} values were Z-transformed and the log value of θ_π ratio ($\theta_\pi 2$ /$\theta_\pi 1$) was estimated. Putative selection targets were extracted based on the top 5% of log-odds ratios for both Z (F_{ST}) and log (θ_π–ratio). Finally for each subspecies we used the intersection of putative selected regions generated by all the pair-wise comparisons with other subspecies as the candidate regions with selective pressure (i.e. consistent signatures of selective sweeps). Genes located in these regions are expected to represent targets of selection. Functional classification and enrichment analysis of GO categories and KEGG pathways for these candidate genes were performed using DAVID (v6.8) [60]. The modified Fisher Exact *P*-value cut off was 0.05.

Genomic divergence and implication for biomedical research

A total of 111 out of 55,404,179 RM SNPs analyzed in this study were successfully mapped to human reference sequence version hg19 (GRCh37) using liftOver (https://genome.ucsc.edu/cgi-bin/hgLiftOver) and were annotated as 'disease causing' in HGMD (version 2015.1) or pathogenic

in ClinVar (downloaded 8/01/2017) (Supplementary Table 11).

For more details of methods please see supplementary notes in Supplementary Material.

Data Access

All data generated from this study have been submitted to the NCBI Sequence Read Archive (SRA) under BioProject PRJNA345528.

Competing interests

The authors declare that they have no competing interests.

Acknowledgments

The project was supported by the Strategic Priority Research Program of the Chinese Academy of Sciences (Grant No. XDPB0202), NSFC (31530068 and 31471989), and National Key R&D Program of China (2016YFC0503200). The authors thank Baoguo Li, Meng Yao, Songtao Guo, Jiqi Lu, Zhenlong Wang, Xuelong Jiang, Tao Meng and Qihai Zhou for their help in sampling; Daniel Pitt, Quan Kang, Qi Wu and Qi Pan for their assistance in data analysis.

Author contributions

Z. L., M. B. and M. L. conceived the study and designed the project. Z. L., X. T., P. O., X. Z. and S. T. managed the project, performed the analyses and wrote the manuscript. Z. L., B. S. and H. X. prepared samples. Z. L., X. T. and P. O. performed genetic analyses. Z. L., X. T., P. O., B. R., L. Z., G. L., Z. Y., Z. P., Z. X., C. R., M. B. and M. L. discussed the data. Z. L. and X. T. wrote the manuscript with contributions from P. O., B. W., H. X., W. Z., C. R., M. B. and M. L.; all authors contributed to data interpretation.

Supplementary Material

Supplementary information, figures S1-S13, tables S1-S12, and notes are available on line.

References

1. Moreno-Estrada A, Gignoux CR, Fernández-López JC et al. Human genetics. The genetics of Mexico recapitulates Native American substructure and affects biomedical traits. Science 2013; **344**:1280-1285.
2. Allentoft ME, Sikora M, Sjögren KG et al. Population genomics of Bronze Age Eurasia. Nature 2015; **522**:167-172.
3. Sudmant PH, Rausch T, Gardner EJ et al. An integrated map of structural variation in 2,504 human genomes. Nature 2015; **526**:75-81.
4. Maestripieri D. Macachiavellian intelligence: How rhesus macaques and humans have conquered the world. 2007. The University of Chicago Press, Chicago.
5. Zinner D, Fickenscher GH, Roos C. Family Cercopithecidae (Old World Monkeys). Handbook of the Mammals of the World. 2013; Pp. 550-753 in: Mittermeier RA, Rylands AB, Wilson DE. eds. Vol. 3. Primates. Lynx Edicions, Barcelona.
6. Xue C, Raveendran M, Harris RA et al. The population genomics of rhesus macaques (*Macaca mulatta*) based on whole genome sequences. Genome Res 2016; **26**:1651-1662.
7. Zhong X, Peng J, Shen QS et al. RhesusBase PopGateway: Genome-Wide Population Genetics Atlas in Rhesus Macaque. Mol Biol Evol 2016; **33**:1370-1375.
8. Fawcett GL, Raveendran M, Deiros DR et al. Characterization of single-nucleotide variation in Indian-origin rhesus macaques (*Macaca mulatta*). BMC Genomics 2011; **12**:311.
9. Yan G, Zhang G, Fang X et al. Genome sequencing and comparison of two nonhuman primate animal models, the cynomolgus and Chinese rhesus macaques. Nature Biot 2011; **29**:1019-1023.
10. Haus T, Ferguson B, Rogers J et al. Genome typing of nonhuman primate models: implications for biomedical research. Trends Genet 2014; **30**:482–487.
11. Flynn S, Satkoski J, Lerche N et al. Genetic variation at the TNF-alpha promotor and malaria susceptibility in rhesus (Macaca mulatta) and long-tailed (Macaca fascicularis) macaques. Infect Genet Evol 2009; **9**:769–777.
12. de Groot NG, Heijmans CMC, Koopman G et al. TRIM5 allelic polymorphism in macaque species/populations of different geographic origins: its impact on SIV vaccine studies. Tissue Antigens. 2011; **78**:256–62.

13. Hernandez RD, Hubisz MJ, Wheeler DA et al. Demographic histories and patterns of linkage disequilibrium in Chinese and Indian rhesus macaques. Science 2007; **316**:240-243.

14. Champoux M, Higley JD, Suomi SJ. Behavioral and physiological characteristics of Indian and Chinese-Indian hybrid rhesus macaque infants. Dev Psychobiol 1997; **31**:49–63.

15. Trichel AM, Rajakumar PA, Murphey-Corb M. Species-specific variation in SIV disease progression between Chinese and Indian subspecies of rhesus macaque. J Med Primatol 2002; **31**:171-178.

16. Tosi AJ, Morales JC, Melnick DJ. Paternal, maternal, and biparental molecular markers provide unique windows onto the evolutionary history of macaque monkeys. Evolution 2003; **57**:1419-1435.

17. Smith DG. Genetic characterization of Indian-origin and Chinese-origin rhesus macaques (*Macaca mulatta*). Comp Med 2005; **55**:227-230.

18. Ferguson B, Street SL, Wright H et al. Single nucleotide polymorphisms (SNPs) distinguish Indian-origin and Chinese-origin rhesus macaques (Macaca mulatta). BMC Genomics 2007; **8**:43.

19. Kubisch HM, Falkenstein KP, Deroche CB et al. Reproductive efficiency of captive Chinese- and Indian-origin rhesus macaque (*Macaca mulatta*) females. Am J Primatol 2012; **74**:174-184.

20. Kanthaswamy S, Johnson Z, Trask JS et al. Development and validation of a SNP-based assay for inferring the genetic ancestry of rhesus macaques (Macaca mulatta). Am J Primatol 2014; **76**:1105-1113.

21. Fooden J. Systematic review of the rhesus macaque, *Macaca mulatta* (Zimmermann, 1780). Field Zool 2000; **96**:1–180.

22. Jiang X, Wang Y, Ma S. Taxonomic revision and distribution of subspecies of rhesus monkey (*Macaca mulatta*) in China. Zool Res 1991; **12**:241-247.

23. Fang X, Zhang Y, Zhang R et al. Genome sequence and global sequence variation map with 5.5 million SNPs in Chinese rhesus macaque. Genome Biol 2011; **12**:R63.

24. Prado-Martinez J, Sudmant PH, Kidd JM et al. Great ape genetic diversity and population history. Nature 2013; **499**:471-475.

25. Evanno G, Regnaut S, Goudet J. Detecting the number of clusters of individuals using the software STRUCTURE: a simulation study. Mol Ecol 2005; **14**:2611-2620.

26. Li H, Durbin R. Inference of human population history from individual whole-genome sequences. Nature 2011; **475**:493-496.
27. Zheng B, Xu Q, Shen Y. The relationship between climate change and Quaternary glacial cycles on the Qinghai–Tibetan Plateau: review and speculation. Quatern Int 2002; **97**:93-101.
28. Excoffier, L. Dupanloup I, Huerta-Sánchez E et al Robust demographic inference from genomic and SNP data. PLoS Genet 2013; **9**:e1003905.
29. Owen LA, Finkel RC, Caffee MW. A note on the extent of glaciation throughout the Himalaya during the global Last Glacial Maximum. Quaternary Sci Rev 2002; **21**:147-157.
30. Owen LA. Latest Pleistocene and Holoene glacier fluctuations in the Himalaya and Tibet. Quaternary Sci Rev 2009; **28**:2150-2164.
31. Wu S, Luo J, Li Q et al. Ecological genetics of Chinese rhesus macaque in response to mountain building: all things are not equal. PLoS ONE 2013; **8**:e55315.
32. Yi X, Liang Y, Huerta-Sanchez E et al. Sequencing of 50 human exomes reveals adaptation to high altitude. Science 2010; **329**:75-78.
33. Bhatia G, Patterson N, Pasaniuc B et al. Genome-wide comparison of African-ancestry populations from CARe and other cohorts reveals signals of natural selection. Am J Hum Genet 2011; **89**:368–381.
34. Zhao SC, Zheng PP, Dong SS et al. Whole-genome sequencing of giant pandas provides insights into demographic history and local adaptation. Nat Genet 2013; **45**:67-71.
35. Bergmann C. Über die Verhältnisse der Wärmeökonomie der Thiere zu ihrer Grösse. Göttinger Studien 1847; **3**:595–708.
36. Zhang P, Lyu MY, Wu CF et al. Variation in body mass and morphological characters in Macaca mulatta brevicaudus from Hainan, China. Am J Primatol 2016; **78**:679-698.
37. Zhao X, Zhang H, Lv X et al. Survey and research of morphological characters of monkeys (*Macaca mulatta*) in the Taihang Mountains. J Henan Nor Uni 1989; **62**:120-125.
38. Kurima K, Warman ML, Krishnan S et al. A member of a family of sulfate-activating enzymes causes murine brachymorphism. Proc Natl Acad Sci USA 1998; **95**:8681-8685.
39. Faiyaz ul Haque M, King LM, Krakow D et al. Mutations in orthologous genes in human spondyloepimetaphyseal dysplasia and the brachymorphic mouse. Nat Genet 1998; **20**:157-162.
40. Dy P, Smits P, Silvester A et al. Synovial joint morphogenesis requires the chondrogenic action

of Sox5 and Sox6 in growth plate and articular cartilage. Dev Biol 2010; **341**:346-359.

41. Amling M, Neff L, Tanaka S et al. Bcl-2 lies downstream of parathyroid hormone–related in a signaling pathway that regulates chondrocyte maturation during skeletal development. J Cell Biol 1997; **136**:205-213.

42. Van Hengel J, Calore M, Bauce B et al. Mutations in the area composite protein αT-catenin are associated with arrhythmogenic rightventricular cardiomyopathy. Eur Heart J 2013; **34**:201-210.

43. González-Alonso J. Human thermoregulation and the cardiovascular system. Exp Physiol 2012; **97**:340-346.

44. Ranneva SV, Pavlov KS, Gromova AV et al. Features of emotional and social behavioral phenotypes of calsyntenin2 knockout mice. Behav Brain Res 2017; **332**:343-354.

45. Horn KE, Glasgow SD, Gobert D et al. DCC expression by neurons regulates synaptic plasticity in the adult brain. Cell Rep 2010; **31**:173-185.

46. Hori K, Hoshino M. Neuronal Migration and AUTS2 Syndrome. Brain Sci 2017; **7**:e54.

47. Hazen MM, Woodward AL, Hofmann I et al. Mutations of the hemophagocytic lymphohistiocytosis-associated gene UNC13D in a patient with systemic juvenile idiopathic arthritis. Arthritis Rheum 2008; **58**:567-570.

48. Procter M, Wolf B and Mao R. Forty-eight novel mutations causing biotinidase deficiency. Mol Genet Metab 2016; **117**:369-372.

49. Munier S, Delcroix-Genete D, Carthagena L et al. Characterization of two candidate genes, NCoA3 and IRF8, potentially involved in the control of HIV-1 latency. Retrovirology 2005; **2**:73.

50. Fan ZY, Song YL. Chinese Primate Status and Primate Captive Breeding for Biomedical Research in China. In: Institute for Laboratory Animal Research, National Research Council. International Perspectives: The Future of Nonhuman Primate Resources. Washington DC: National Academy Press. 2003.

51. Hao Xin. Monkey Research in China: Developing a Natural Resource. Cell 2007; **129**: 1033-1036.

52. Gibbs RA, Rogers J, Katze MG et al. Evolutionary and biomedical insights from the rhesus macaque genome. Science 2007; **316**:222-234.

53. Li H, Durbin R. Fast and accurate short read alignment with Burrows-Wheeler transform.

Bioinformatics 2009; **25**:1754-1760.

54. Li H, Handsaker B, Wysoker A et al. The sequence alignment/map format and SAMtools. Bioinformatics 2009; **25**:2078–2079.

55. Chen C, Liu Z, Pan Q et al. Genomic analyses reveal demographic history and temperate adaptation of the newly discovered honey bee subspecies *Apis mellifera sinisxinyuan* n. ssp. Mol Biol Evol 2016; **33**:1337–1348.

56. Wang K, Li M, Hakonarson H. ANNOVAR: functional annotation of genetic variants from high-throughput sequencing data. Nucleic Acids Res 2010; **38**:e164.

57. Patterson N, Price AL, Reich D. Population structure and eigenanalysis. PLoS Genet 2006; **2**:e190.

58. Barrett JC, Fry B, Maller J et al. Haploview: analysis and visualization of LD and haplotype maps. Bioinformatics 2005; **21**:263-265.

59. Danecek P, Auton A, Abecasis G et al. The variant call format and VCFtools. Bioinformatics 2011; **27**:2156-2158.

60. Huang da W, Sherman BT, Lempicki RA. Systematic and integrative analysis of large gene lists using DAVID Bioinformatics Resources. Nat Protoc 2009; **4**:44-57.

Figure Legends

Figure 1. Phylogeny and population genetic structure of 81 wild Chinese RMs. (a) Geographic distribution of RMs in China (gray shadow) and the 17 sampling sites along with their subspecies assignment. (b) Neighbor-joining (NJ) tree and clustering solution inferred using STRUCTURE and displaying five populations (inferred with Evanno's ΔK method; Supplementary Fig. 5). (c) Principal component analysis plots depicting the first two components (variance explained by PC1 = 6.40% and PC2 = 5.07%).

Figure 2. Demographic history and differentiation scenarios of Chinese RMs. (a) Historical changes in effective population size reconstructed using the pairwise sequential Markovian coalescent (PSMC) applied on individual whole genomes for each of the five subspecies. The generation length (g) and the neutral mutation rate per generation (μ) were assumed to be 11 years and 1.08×10^{-8}, respectively. The Naynayxungla Glaciation (NG, 780-500 kya), Penultimate Glaciation (PG, 200-130 kya) and Last Glaciation (LG, 70-10 kya) are shaded in gray. (b) Demographic history inferred by *fastsimcoal2*. The width of the gray bars and numbers on them indicate the estimated effective population size. The arrows indicate migration patterns with the numbers above arrows indicating the average number of migrants per generation between different subspecies. Numbers at the right show the divergence times between subspecies. (c) Biogeographic scenario for RMs. Chinese RMs separates from Indian RMs ~ 162 kya [13], followed by further migration into China by the different RM subspecies indicated with arrows colored following the color key in Fig. 1a.

Figure 3. Genomic regions with selection sweep signals in RM. (a) Distribution of $\log_2$ (θ_π *M. m. lasiotis*/θ_π *M. m. tcheliensis*) and Z (F_{ST}) of 50-kb windows with 25-kb steps. Blue dots located in the selected regions requirement (corresponding to Z test P<0.05, where Z (F_{ST}) >= 1.848 and θ_π log-ratio >= 1.203) represent selected windows for *M. m. tcheliensis*. (b) Morphological comparison between *M. m. tcheliensis* and *M. m. lasiotis*. M and F represent males and females. (c) Example of genes with selection sweep signals. *Sox5, Bcl2* and *Papss2* in *M. m. tcheliensis* and *Camk2g* and *Ppp3cb* in *M. m. brevicaudus.* F_{ST} and θ_π log-ratio between the two subspecies are represented in red and blue, respectively. All values in figure 3c are plotted using 50-kb windows with half steps. Genome annotations are show at the bottom (black bar, coding sequences (CDS); purple and orange bar, genes). (d) SNP genotypes in putative selective sweeps containing *Sox5, Bcl2, Papss2, Camk2g* and *Ppp3cb*.

Figure 4. Population study of putative pathogenic SNPs in Chinese RM subspecies. (a) The site and frequency of pathogenic SNPs located in *Unc13d* and *Btd* genes. (b) Scheme of the *Ncoa3* gene in RM. The positions of nonsynonymous polymorphisms (black) and three amino-acid deletions (in red) are marked. (c) Private and shared pathogenic SNPs in Chinese RM subspecies (blue: *M. m. tcheliensis;* orange: *M. m. brevicaudus;* red: *M. m. littoralis;* green: *M. m. mulatta;* purple: *M. m. lasiotis*). The sizes of the areas are not proportional to the magnitude of the numbers. (d) NJ tree including the 81 Chinese RMs derived from this study, the 26 captive Chinese RMs from Zhong et al. [7] are indicated by blue dot.

Click here to download Figure Figure.1.pdf

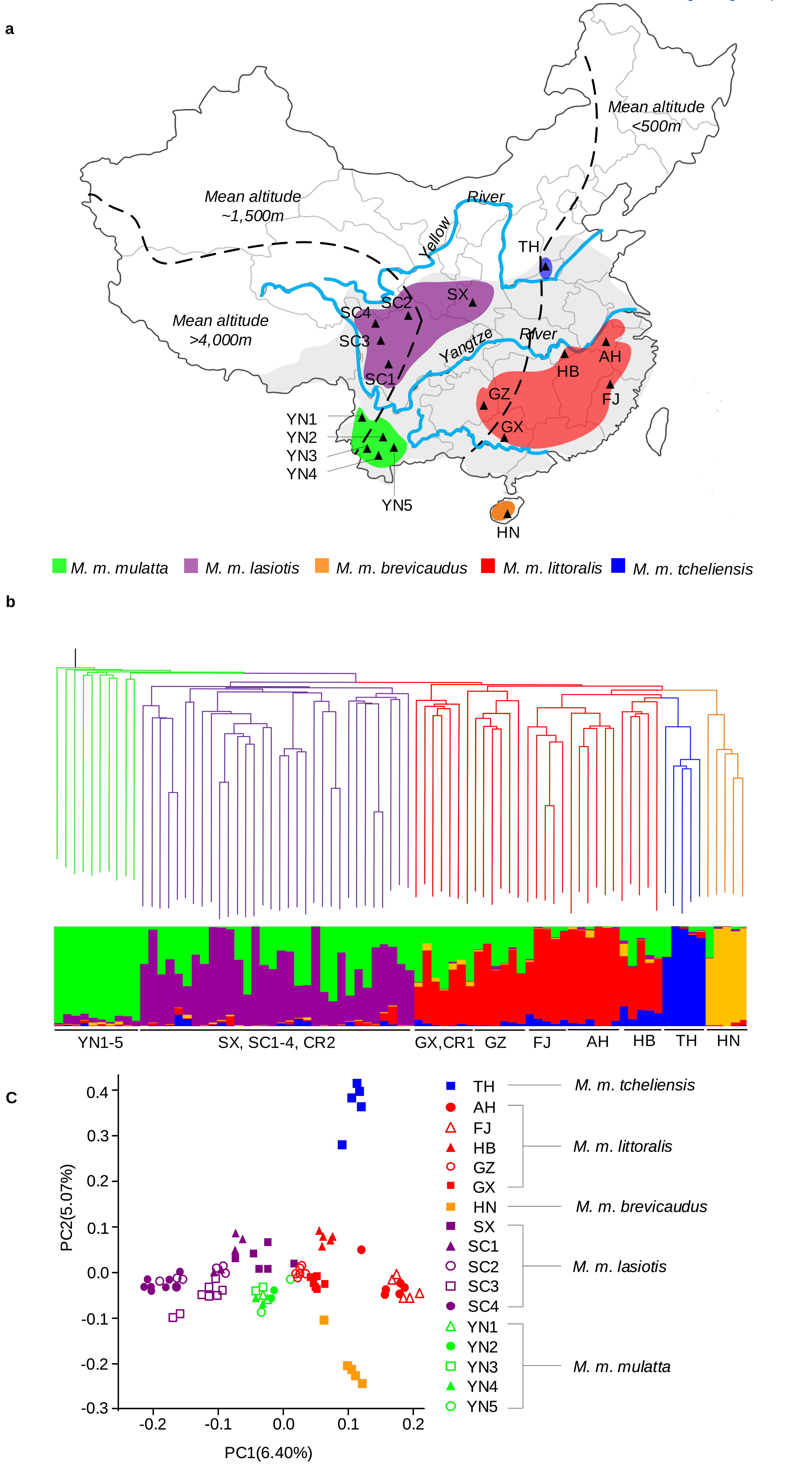

Figure

Click here to download Figure Figure.2.pdf

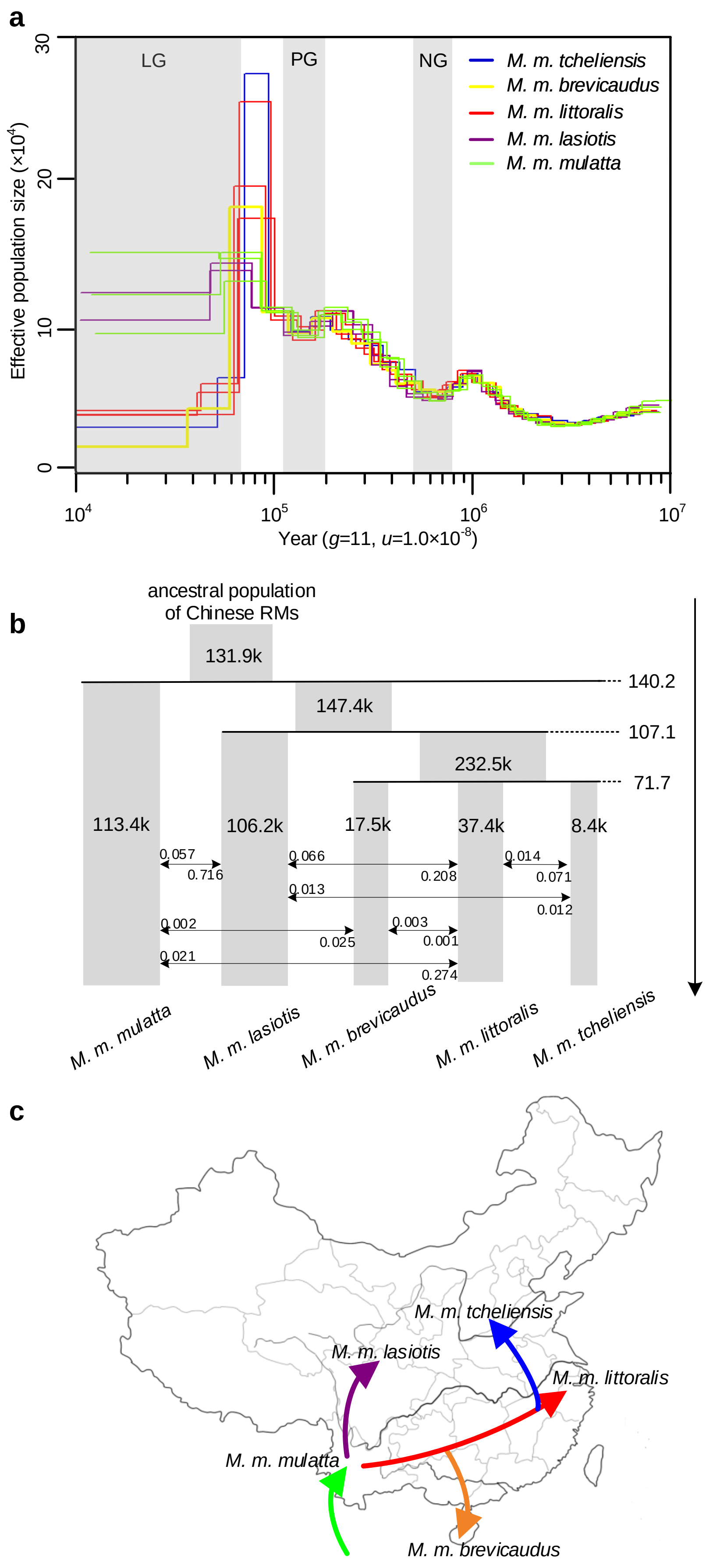

Figure

Click here to download Figure Figure 3.pdf

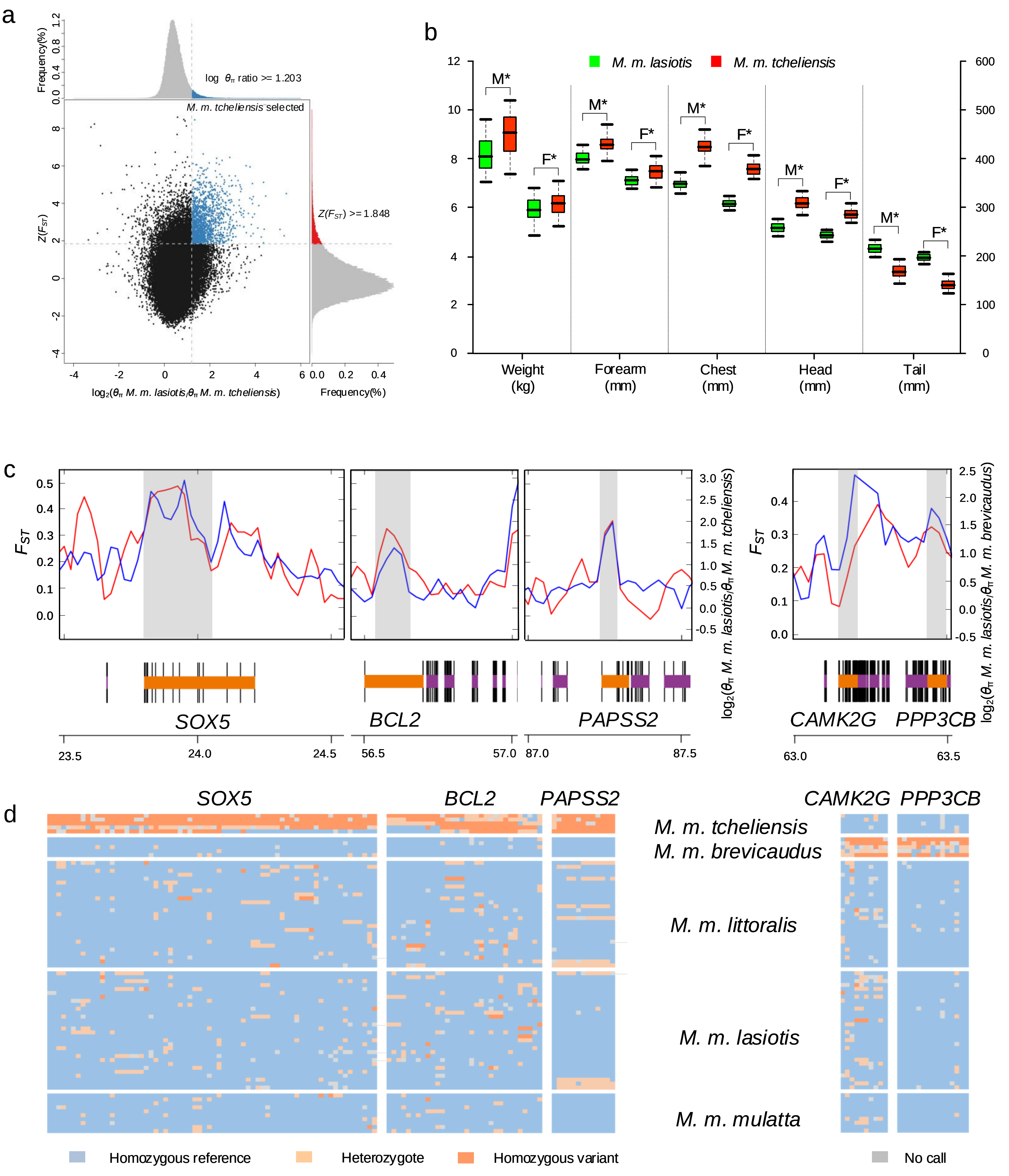

Figure

Click here to download Figure Figure 4.pdf

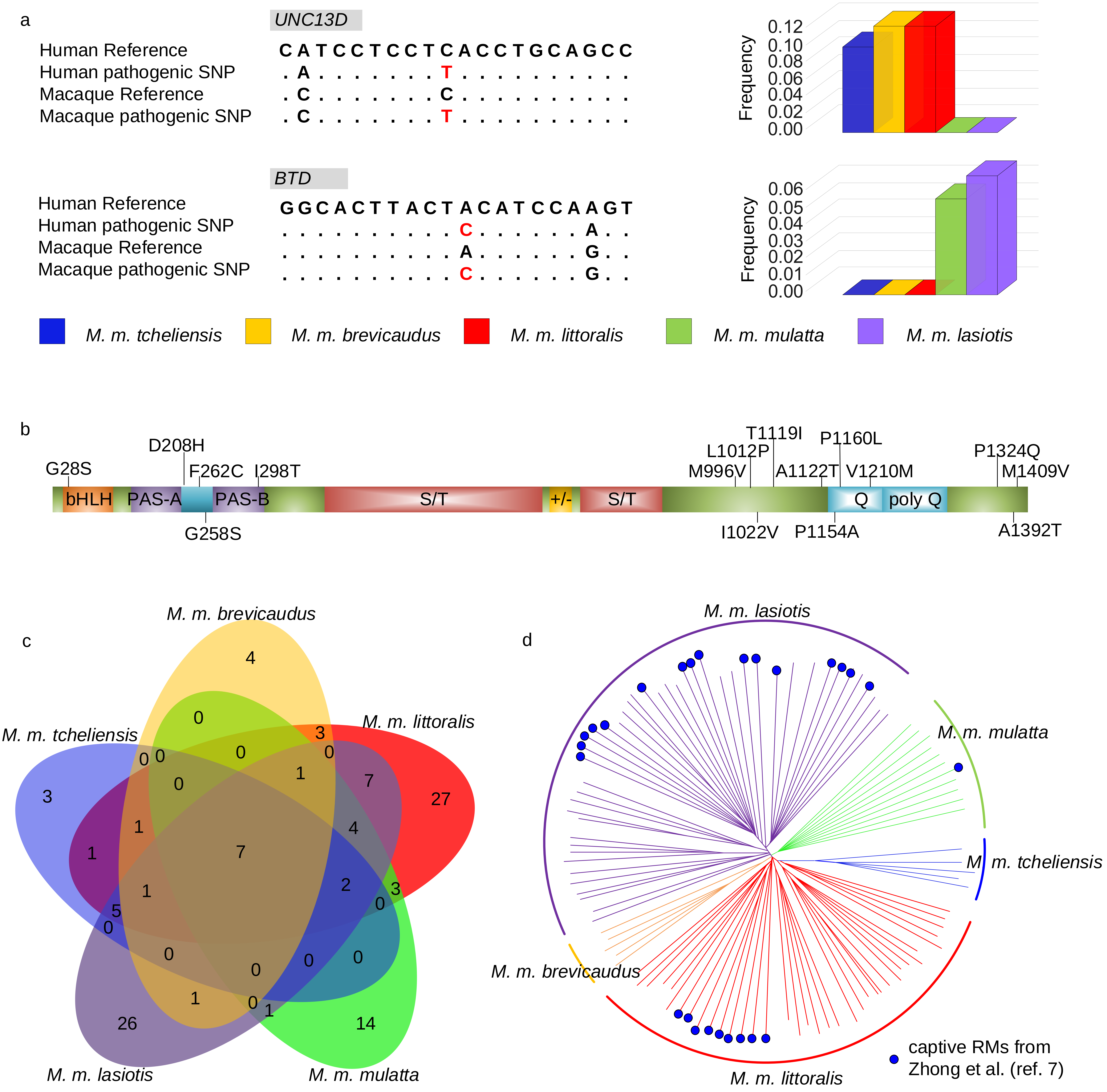

Click here to access/download
Supplementary Material
renamed_86b67.docx

Supplementary data

Click here to access/download
Supplementary Material
Supplemental Data 1.xlsx

Supplementary data

Click here to access/download
Supplementary Material
Supplemental Data 2.xlsx
