# Supplementary material for: Population genomics of wild Chinese rhesus macaques reveals a dynamic demographic history and local adaptation, with implications for biomedical research
Source: Gigascience. 2018 Aug 27;7(9):giy106. doi: 10.1093/gigascience/giy106 (PMC6143732; doi:10.1093/gigascience/giy106)
Supplement: GIGA-D-17-00291_Revision_1.pdf [file giy106_giga-d-17-00291_revision_1.pdf]

GigaScience

Population genomics of wild Chinese rhesus macaques reveals a dynamic demographic history and local adaptation, with implications for biomedical research

--Manuscript Draft--

Manuscript Number:	GIGA-D-17-00291R1
Full Title:	Population genomics of wild Chinese rhesus macaques reveals a dynamic demographic history and local adaptation, with implications for biomedical research
Article Type:	Research
Funding Information:	Strategic Priority Research Program of the Chinese Academy of Sciences (XDPB0202) — Dr Ming Li<br>NSFC (31530068) — Dr Ming Li<br>NSFC (31471989) — Dr Zhijin Liu<br>National Key R&D Program of China (2016YFC0503200) — Dr Ming Li
Abstract:	The rhesus macaque (RM, Macaca mulatta) is the most important nonhuman primate model in evolutionary biology and biomedical research. We present the first population genomics survey of wild RMs, comprising 81 geo-referenced individuals representing five subspecies from 17 locations in China, covering a large fraction of the species' natural distribution. A total of 58.7 million of autosomal single nucleotide polymorphisms (SNPs) were detected. We find a hierarchical population structure with four distinct genetic lineages on the mainland and one on Hainan Island recapitulating current subspecies designations. The five subspecies are estimated to have diverged between 116 and 45 thousand years ago, but with recent gene flow among some groups. Consistent with the expectation of a larger body size in colder climates and a smaller body size in warmer climates (Bergman's rule), both the northernmost RM lineage (subspecies, M. m. tcheliensis), which exhibits the largest body size of all Chinese RMs, and the southernmost RM lineage (subspecies, M. m. brevicaudus), which exhibits the smallest body size of all Chinese RMs, are featured with positively selected genes responsible for skeletal development. In addition, two candidate selected genes (Fbp1, Fbp2) found in M. m. tcheliensis are involved in gluconeogenesis, which might play a key role to maintain a stable blood glucose levels during starvation when food resources are scarce in winter. The tropical subspecies M. m. brevicaudus is also characterized by positively selected genes related to cardiovascular function and response to temperature stimuli, which are potentially involved in adaptation to tropical climates. We further delineated 118 RM SNPs matching human disease-causing variants with 82 being subspecies-specific. The data presented herein provides a reference resource for the choice of RMs when carrying out biomedical experiments. The unexpected demographic history of Chinese RMs, coupled with their history of local adaption offers new insights into the evolution of RMs and provides valuable baseline information for biomedical research.
Corresponding Author:	Ming Li<br><br>CHINA
Corresponding Author Secondary Information:	
Corresponding Author's Institution:	
Corresponding Author's Secondary Institution:	
First Author:	Zhijin Liu
First Author Secondary Information:	
Order of Authors:	Zhijin Liu

Powered by Editorial Manager® and ProduXion Manager® from Aries Systems Corporation

	Xinxin Tan
	Pablo Orozco-terWengel
	Xuming Zhou
	Liye Zhang
	Shilin Tian
	Zhongze Yan
	Huailiang Xu
	Baoping Ren
	Peng Zhang
	Zuofu Xiang
	Binghua Sun
	Christian Roos
	Michael W. Bruford
	Ming Li
**Order of Authors Secondary Information:**	
**Response to Reviewers:**	Dear Dr. Hans Zauner,<br><br>Thank you for your consideration and encouragement of our submission to GigaScience. In light of the reviewers' constructive comments, we have revised the manuscript and would like to re-submit it to GigaScience. Generally, we have re-done the analyses based on the updated genome reference of rhesus macaque (Mmul_8.0.1) and re-designed the models for demographic model testing. The point-by point response to the comments are below:<br><br>Reviewer #1:<br><br>Comment 1-1:<br>The rhemac2 reference genome assembly is an old genome reference sequence. There are now more recent, higher quality reference assemblies for this species. However, I do not think that the use of rhemac2 is necessarily a major problem for the population phylogenetics and demographic analyses. SNPs identified using rhemac2 should be very similar (though not identical) to the SNP calls that would be obtained using the more recent assemblies. And it should not be a problem that the rhemac2 assembly is built from an Indian-origin animal. It would have been better (more comprehensive and less susceptible to errors) for the authors to use a more recent reference genome, but using rhemac2 for the evolutionary and demographic analyses does not seem to me to be a major concern.<br>Response 1-1:<br>We really appreciated this suggestion. We have performed SNP calling using the new genome reference of rhesus macaque Mmul_8.0.1 (line 101 and 378). The population structure, phylogenetic and demographic analyses were also carried out with the new dataset. As predicted by the reviewer, the new results are very similar to the former results using rheMac2 (Figure 1, Supplementary Fig. 4 and 5, Supplementary Table 4).<br><br>Comment 1-2:<br>The second aspect of the paper is an analysis of functional genetic variation. The authors used FST and other population statistics to identify regions of the macaque genome that show significant differentiation among populations, focusing particularly on the most northern and most southern populations. These analyses suggest that there has been selection for differences in skeletal development and cardiovascular physiology that distinguish Chinese rhesus subspecies (selective sweeps). I do have some concerns about these analyses.<br>a) First and most importantly, this is where the use of rhemac2 as the reference assembly seems to me to be somewhat problematic. The rhemac2 assembly contains

Powered by Editorial Manager® and ProduXion Manager® from Aries Systems Corporation

some assembly errors. But more relevant to this manuscript, it was annotated by NCBI and Ensembl before there was substantial RNA sequence data to assist in gene prediction. Investigators who have used rhemac2 for functional studies of protein-coding genes have found errors in some of the gene models, likely due to the lack of access to good RNA sequence data at the time of the annotation. The newer reference genomes for rhesus macaque (e.g. Mmul_8.0.1) have also been annotated by NCBI and Ensembl. These newer annotations are more complete and more accurate because there is now more RNA sequence data available to support gene models and to identify true exon-intron boundaries. I would be concerned that some of the conclusions Liu et al. have generated regarding selection on specific genes may be problematic due to potential problems with rhemac2 gene annotations. Even though the analyses depend on FST and related statistics 9 (and not dN/dS ratios), I assume that the authors did examine the coding sequence differences among Chinese rhesus populations for the genes that they infer were under selection. I recommend that the authors (at a minimum) re-check their analyses and conclusions regarding positive selection on specific genes, using the more accurate, better annotated reference assemblies that were produced more recently than rhemac2.

Response 1-2:

In this revised manuscript, all analyses were carried out based on the genome reference and the annotation of Mmul_8.0.1. Most of the previously observed selection signals were confirmed, but also new findings were obtained.

Among the 176 genes found to be under positive selection in M. m. tcheliensis, two (Fbp1, Fbp2, modified Fisher Exact P=1.90E-02; Fig. 3c, d; Supplementary Table 7) are enriched in the gene ontology (GO) term “fructose 1, 6-bisphosphate 1-phosphatase activity”. These two genes encode for fructose-1, 6-bisphosphatase 1 and fructose-1, 6-bisphosphatase isozyme 2 which catalyze the hydrolysis of fructose 1, 6-bisphosphate and play a rate-limiting role in gluconeogenesis. Furthermore, in starved zebrafish it was shown that the expression of Fbp1 was significantly unregulated in brain and liver tissues. Our findings suggest that the regulation of gluconeogenesis might be a mechanism of M. m. tcheliensis to adapt to food shortage in winter. (line 222-236)

Additionally, we have found 127 putatively selected genes in M. m. brevicaudus, four of which were enriched in GO term “Bone morphogenetic protein (BMP) signaling pathway” (modified Fisher Exact P=4.65E-02) and two genes were enriched in GO term “I-SMAD binding (P=4.65E-02)”. These genes under selection might have contributed to smaller body size of M. m. brevicaudus and adaptation to hot climate. (line 257-265)

Comment 1-3:

b) It is not clear from this version of the manuscript (lines 207-219) whether Liu et al. observed any non-synonymous variants in the genes they identified as showing evidence of selective sweeps. Were there non-synonymous differences in the alleles found in the different Chinese rhesus populations, or were all the FST values based on intronic and/or intervening SNPs between genes? The case for positive selection on PAPSS2, SOX5 and other genes would be stronger if the authors identified non-synonymous or other coding variants that are predicted to influence protein function. If there are no non-synonymous differences observed between populations, then Liu et al. would (I suppose) have to argue that the selection was on non-coding regulatory variants. No specific statement about how the proposed selection is suggested to have influenced these genes is presented in the manuscript. Readers should be informed as to what particular variants distinguish the alleles in M. m. tcheliensis from M. m. brevicaudus, etc., and why the authors believe the observed sequence differences constitute true functional differences.

Response 1-3:

This is a very good point. Both coding and non-coding changes could contribute to local adaptations of organisms. To further investigate the adaptive mechanism of M. m. tcheliensis and M. m. brevicaudus to the opposite climates (cold versus hot), we focused on SNPs in the gene regions of above described candidate genes. A total of 5817 SNPs were found with significant differences at the 5% level in the distributions of genotypes between these two subspecies, and 10 SNPs were non-synonymous variants (Supplementary table 10 and 11). In M. m. tcheliensis, non-synonymous mutations were found in the coding regions of Atp6v0a4 (R667Q), Ext2 (I363M), Fto (N10S) and Rpgrip1l (R1281Q) (Supplementary table 11 and Supplementary Fig. 13), implying that selection might has acted on protein sequence changes. No non-

synonymous changes were detected in Fbp1, Fbp2, Sox5 and Sox6. However, SNPs are located in the 1kb up/downstream, 5' and 3' UTR, and intronic regions of these genes (Supplementary table 10), indicating selection on non-coding regulatory variants. Correspondingly, non-synonymous mutations in Aggf1 (H343Y), Axin1 (A674G, T656I), Hspa4 (I782V) and Ctnna3 (V551I, T577M) were revealed for M. m. brevicaudus (Supplementary table 11 and Supplementary Fig. 13) (line 278-291).

Comment 1-4:
c) It is not stated (lines 220-232) whether the GO terms related to heart development, heart rate or temperature response are statistically significantly enriched in this analysis. The authors should provide the same type of statistical evidence for these GO term results that they do for the limb morphogenesis results above.
Response 1-4:
We have found three putatively selected genes related to GO terms of "blood vessel morphogenesis", "regulation of heart rate by cardiac conduction" and "response to temperature stimulus". However, these GO terms are not significantly enriched (line 266-271).

Comment 1-5:
The new results presented in this paper regarding phylogenetic relationships among populations, and the history of population differentiation and effective size change, are important findings and make a valuable contribution to the literature.
Response 1-5:
Thank you for such an evaluation.

Other minor issues:
Comment 1-6:
Line 79: I think there may be a typo here. I do not think the authors intend to state that the effective population size of Indian rhesus macaques is only 17,000. This should be checked again.
Response 1-6:
It is not a typo here. The study on demographic history of Chinese and Indian RMs by Hernandez et al. (2007) revealed effective population sizes of ~ 17,014 and 239,704 for Indian and Chinese populations, respectively.

Comment 1-7:
Lines 137-148: It might be useful to compare the results for population size change over time that Liu et al. obtain here to those of previous population genetic analyses of rhesus macaques (e.g. Xue et al. 2016 and Hernandez et al. 2007).
Response 1-7:
We really appreciated this suggestion. Interestingly, the demographic inference by Xue et al. 2016 of the genomic data for one Chinese RM (CH_37945) from AH (M. m. littoralis) qualitatively resembled the demographic trajectory of M. m. littoralis herein presented (line 156-158).
Hernandez et al. reported that Chinese RM population has experienced 3.3-fold growth. We have checked the sample information of nine Chinese RMs included in Hernandez et al. 2007. Seven of the Chinese animals were sampled from Suzhou (eastern China), one from Kunming (western China), and one from Guandong (eastern China), which means eight individuals of M. m. littoralis and one of M. m. mulatta. Coincidently, a population expansion of M. m. littoralis (from NA1 =2.0k to Nli = 24.6k; Supplementary Table 5) since 44.8 kya was also detected in our results (line 175-178). However, since the RMs studied in Hernandez et al. 2007 were captive-born, although with wild-caught parents, different populations have been mixed. Wild-caught RMs are often transferred from one breeding center to another. Thus we think a comparison between captive- and wild-born RMs perhaps is inappropriate. So we do not address this point in the manuscript.

Comment 1-8:
Lines 145-148: How do the authors reconcile the different estimates for effective population size at about 60-80,000 years ago for M. m. tcheliensis, M. m. littoralis and M. m. brevicaudus that were obtained by the PSMC analysis versus the fastsimcoal2 analysis? Do the authors favor one of these over the other? Is there possibly a way to reconcile these different results?
Response 1-8:

Powered by Editorial Manager® and ProduXion Manager® from Aries Systems Corporation

The fastsimcoal2 analysis revealed a bottleneck in population size (NA1 =2.0k and NA2 =1.5k) of pan-eastern RMs (M. m. tcheliensis, M. m. littoralis and M. m. brevicaudus) during the period from 111.9 kya to 45.0 kya (Fig. 2b), which coincided with the population decline of pan-eastern RMs since approximately 100 kya as revealed by PSMC analyses. However, the population growth that occurred in pan-eastern RMs after the bottleneck has not been detected by PSMC analyses, given that PSMC is less accurate when reconstructing recent histories within ~100 kya (line 175-180). We prefer the PSMC analysis to reconstruct the historical demography older than 100 kya, and prefer fastsimcoal2 to model more recent demographic fluctuations.

Reviewer #2:

Comment 2-1:

In the first part, the authors reconstruct the phylogeny of Chinese rhesus macaques based on a whole-genome neighbor-joining tree. This is a rather crude type of phylogenomic analysis and doesn't allow to draw conclusions about the evolutionary history as done on lines 109-114. Here, the paper would benefit a lot from applying proper species tree methods that take incomplete lineage sorting into account. This will provide a reliable picture about the phylogenetic relationships of the five subspecies that can then act as a useful starting point to design a set of demographic models to test in the next step.

Response 2-1:

Many thanks for this helpful comment. To reveal the phylogenetic relationships among the five Chinese RM subspecies, we now employed the SVDquartets approach that takes incomplete lineage sorting into account (line 159-160, 412-414). The obtained phylogenetic tree suggests a “step-by-step” divergence for five Chinese RM subspecies. Accordingly, the M. m. mulatta lineage diverged form that of the remaining Chinese RMs firstly and then the M. m. lasiotis diverged from the ancestral lineage of pan-eastern RMs (M. m. tcheliensis, M. m. littoralis and M. m. brevicaudus). Subsequently, M. m. brevicaudus diverged from the ancestor of M. m. tcheliensis and M. m. littoralis, the divergence of which occurred lastly (Supplementary Fig. 6) (line 161-166). We used this pattern as starting point to design testable demographic models (see below).

Comment 2-2:

My main concern deals with the design of the models for demographic model testing. Here, the paper lacks critical details to understand the reasoning behind the selection of the 8 compared models. It's completely unclear how these models have been chosen from the total number of possible (sub)species tree configurations and how they were parameterized. Supplementary Table 5 shows that the number of parameters in these 8 models range from 6 to 12, but they seem to do so in a very unintuitive way. For example, in Supplementary Figure 6 it seems that model 2 is a simplified version of model 8 with one less divergence time parameter. But Supplementary Table 5 shows that model 2 has actually 3 parameters more than model 8. Moreover, for parameter estimation, the authors expanded the selected model 2 by additional parameters without specifying which of the parameters listed in Supplementary Table 6 have already been part of the model selection. Comparing oversimplified models might lead to the selection of a suboptimal model in the first step. It's therefore absolutely crucial that the authors provide a detailed table showing the parameterization of all tested models (including parameter bounds) and explain in detail the reasoning behind the selection and design of these models. The type and parameterization of models has a strong impact on the outcome of such model testing approaches and without this critical information, it's impossible to assess how robust the findings of this analysis actually are. Additionally, the authors should provide a measure of the goodness of fit of the selected scenario to show that this model can reasonably well explain the observed data.

Response 2-2:

The SVDquartets approach (see above) revealed only one divergence scenario. Under this “step-by-step” divergence scenario, we performed the joint site frequency spectrum (SFS) approach implemented in fastsimcoal2 to model demographic fluctuations, respective divergence times and gene flow events among the five RM subspecies. In Supplementary Table 5 the full results are provided. (line 159-181)

Powered by Editorial Manager® and ProduXion Manager® from Aries Systems Corporation

Comment 2-3:
In the positive selection analysis, the authors calculate genetic diversity (theta pi) based on their set of variable sites only. This approach is flawed, as it doesn't allow to distinguish between non-variable sites and sites that are not sufficiently covered for reliable genotyping in the sequenced individuals. It is therefore important that the authors take coverage information for every site in the genome into account in order to obtain reliable estimates of window-wise genetic diversity.
Response 2-3:
In the revised manuscript, we performed SNP calling again following GATK's best practice based on the single-sample calling plus joint genotyping workflow. For a variant which is not callable because of low coverage when processed separately, Joint calling allows evidence to be accumulated over all samples and renders the variant callable. In our re-analysis, we detected 58.7 million autosomal SNPs, while before only 55.4 million SNPs were found. The additionally called SNPs are due to the refined protocol and better genome reference. (line 378-387)

Minor issues:
Comment 2-4:
Lines 31-33: Genetic diversity is measured over all sites, not just the SNPs (see above).
Response 2-4:
It has been amended accordingly. We just say "A total of 58.7 million autosomal single nucleotide polymorphisms (SNPs) were detected". (line 32-33)

Comment 2-5:
Line 51: Not clear what 'successful' is supposed to mean here.
Response 2-5:
Judged by population size and geographic distribution, RMs are, after humans, the world's most successful primate, occupying a vast geographic distribution. Here we replaced "'successful" with "most widely distributed" (line 55-56).

Comment 2-6:
Line 82: "including phylogenetic and demographic analyses, as well as genome-wide selection scans, …"
Response 2-6:
It has been amended accordingly. (line 87-90)

Comment 2-7:
Lines 97-98: The number of SNPs is not informative here, since it depends on the number of individuals. Use suitable measures of genetic diversity, such as Watterson's theta or pi.
Response 2-7:
It has been amended accordingly. The value of Watterson's θ (S) and genetic diversity (π) is 0.00342 and 0.00228, respectively (Table 1). (line 102-104)

Comment 2-8:
Lines 98-99: Not clear if the number of SNPs per individual refers to all positions with differences to the reference or only the heterozygous positions within individuals.
Response 2-8:
It refers to all positions with differences to the genome reference. (line 104-106)

Comment 2-9:
Lines 99-103: Use consistent style for point estimates and CI in the brackets, i.e. proportions instead of percentages.
Response 2-9:
It has been amended accordingly.

Comment 2-10:
Lines 103-105: Are these numbers only referring to shared segregating variation or also including fixed differences to the reference?
Response 2-10:
Including fixed differences to the genome reference. (line 106-108)

Comment 2-11:

Powered by Editorial Manager® and ProduXion Manager® from Aries Systems Corporation

Line 116: "admixture proportions"
Response 2-11:
It has been amended accordingly (line 120).

Comment 2-12:
Lines 149-150: "we further employed a joint site frequency spectrum (SFS) based approach to model"
Response 2-12:
It has been amended accordingly (line 167).

Comment 2-13:
Lines 152-153: Unclear what is meant by "produced a significantly better fit of a step by step divergence scenario than alternative ones, …"
Response 2-13:
This part has been re-written and this sentence has been removed.

Comment 2-14:
Line 167: Start a new sentence after "an eastern clade"
Response 2-14:
It has been amended accordingly (line 184).

Comment 2-15:
Lines 233-234: "we also found signatures of positive selection in genes related to …"
Response 2-15:
It has been amended accordingly. (line 292-293)

Comment 2-16:
Lines 234-235: The 104 candidate genes are enriched for a certain GO term, rather than the three genes being enriched in a certain GO term.
Response 2-16:
It has been amended accordingly (line 293-296).

Comment 2-17:
Lines 323-324: Provide more details about the variant calling here. Just providing the reference is not sufficient for the reader to get a quick overview of the applied methods.
Response 2-17:
It has been amended accordingly. We performed SNP calling following GATK's best practice and the more details about the variant calling protocol was described in line 378-388.

Comment 2-18:
Line 334: "branch support" instead of "branch reliability"
Response 2-18:
It has been amended accordingly (line 393).

Comment 2-19:
Line 343: "Decay of linkage disequilibrium against physical distance"
Response 2-19:
It has been amended accordingly (line 402-404).

Comment 2-20:
Line 349: Provide more details about the reasoning behind choosing the stated values for generation time and mutation rate.
Response 2-20:
For all demographic estimations, we have chosen a mutation rate of 1×10^{-8} per site per generation and a generation time of 11 yr, which were used in the previous population genomic analyses of 133 rhesus macaques (Xue et al. 2017). To compare our results and Xue's results, we toke the same values of mutation rate and generation time. Xue et al. explained the rationale behind these values: "The most appropriate mutation rate to use for this type of analysis remains somewhat controversial, in which a variety of methods have been used to determine the "best" estimate (Ségurel et al. 2014). For rhesus macaques, there is far less empirical evidence. We chose a mutation rate of 1.0×10^{-8} per site per generation for macaques, because a review of

	the data for humans suggests a rate of 1.0–1.5×10−8 per site per generation (Ségurel et al. 2014). Assuming the generation time for rhesus macaques is 11 yr and humans is 25 yr, the per year mutation rates are then 0.9×10−9 for macaques and 0.4–0.6×10−9 for humans, an appropriate ratio given the demonstrated slowdown in humans and other hominoids. Generation time is set at 11 yr based on the field data that indicate rhesus macaques begin reproduction ~6 yr of age and can breed until their late teens, resulting in age at median birth of ~11 yr." However, perhaps it is not appropriate to paste this explanation into our manuscript. Thus I said "for the rationale to use these values see [6, 73] (Ségurel et al. 2014; Xue et al. 2017)". (line 409-410)<br><br>Comment 2-21:<br>Line 353: "to model" rather than "to simulate"<br>Response 2-21:<br>It has been amended accordingly (line 415).<br><br>Comment 2-22:<br>Line 356: "to identify the one that is best supported by the observed data"<br>Response 2-22:<br>This part has been re-written and this sentence has been removed.<br><br>Comment 2-23:<br>Line 359: How many replicates?<br>Response 2-23:<br>Each model was tested for 200 replicates (line 420).<br><br>Comment 2-24:<br>Line 364: Which additional parameters? See comment above.<br>Response 2-24:<br>This part has been re-written and this sentence has been removed.<br><br>Comment 2-25:<br>Line 371-373: Rewrite this sentence.<br>Response 2-25:<br>It has been amended accordingly. (429-430)<br><br>Comment 2-26:<br>Line 379: "candidate regions under positive selection"<br>Response 2-26:<br>It has been amended accordingly (line 436).
**Additional Information:**	
**Question**	**Response**
Are you submitting this manuscript to a special series or article collection?	No
**Experimental design and statistics**<br><br>Full details of the experimental design and statistical methods used should be given in the Methods section, as detailed in our Minimum Standards Reporting Checklist. Information essential to interpreting the data presented should be made available in the figure legends.<br><br>Have you included all the information requested in your manuscript?	Yes
**Resources**	Yes

A description of all resources used, including antibodies, cell lines, animals and software tools, with enough information to allow them to be uniquely identified, should be included in the Methods section. Authors are strongly encouraged to cite Research Resource Identifiers (RRIDs) for antibodies, model organisms and tools, where possible.<br><br>Have you included the information requested as detailed in our Minimum Standards Reporting Checklist?	
**Availability of data and materials**<br><br>All datasets and code on which the conclusions of the paper rely must be either included in your submission or deposited in publicly available repositories (where available and ethically appropriate), referencing such data using a unique identifier in the references and in the "Availability of Data and Materials" section of your manuscript.<br><br>Have you have met the above requirement as detailed in our Minimum Standards Reporting Checklist?	Yes

Powered by Editorial Manager® and ProduXion Manager® from Aries Systems Corporation

Manuscript Click here to download Manuscript renamed_77586.docx

Title: Population genomics of wild Chinese rhesus macaques reveals a dynamic demographic history and local adaptation, with implications for biomedical research

Running Title: Population genomics of wild rhesus macaques

Zhijin Liu[1, †], Xinxin Tan[1, 2, †], Pablo Orozco-terWengel[3, †], Xuming Zhou[4], Liye Zhang[1, 2], Shilin Tian[5], Zhongze Yan[1, 6], Huailiang Xu[7], Baoping Ren[1], Peng Zhang[8], Zuofu Xiang[9], Binghua Sun[10], Christian Roos[11], Michael W. Bruford[3, *], Ming Li[1, 12 *]

[1] Key Laboratory of Animal Ecology and Conservation Biology, Institute of Zoology, Chinese Academy of Sciences, Beijing, China.

[2] University of Chinese Academy of Sciences, Beijing 100039, China.

[3] School of Biosciences, Cardiff University, Sir Martin Evans Building, Museum Avenue, Cardiff CF10 3AX, United Kingdom.

[4] Division of Genetics, Department of Medicine, Brigham and Women's Hospital, Harvard Medical School, Boston, MA 02115, USA.

[5] Novogene Bioinformatics Institute, Beijing 100083, China.

[6] Institute of Health Sciences, Anhui University, Hefei, 230601, China.

[7] College of Life Science, Sichuan Agricultural University, Ya'an 625014, China.

[8] School of Sociology and Anthropology, Sun Yat-sen University, Guang Zhou, China.

[9] College of Life Science and Technology, Central South University of Forestry and Technology, Changsha 410004, Hunan, China.

[10] School of Life Sciences, Anhui University, Hefei, 230601, China.

[11] Gene Bank of Primates and Primate Genetics Laboratory, German Primate Center, Leibniz Institute for Primate Research, Kellnerweg 4, 37077 Göttingen, Germany.

[12] Center for Excellence in Animal Evolution and Genetics, Chinese Academy of Sciences, Kunming, 650223, China.

[†] Contributed equally

***** Correspondence: Ming Li, lim@ioz.ac.cn; Michael W. Bruford, BrufordMW@cardiff.ac.uk

Abstract

The rhesus macaque (RM, *Macaca mulatta*) is the most important nonhuman primate model in evolutionary biology and biomedical research. We present the first population genomics survey of wild RMs, comprising 81 geo-referenced individuals representing five subspecies from 17 locations in China, covering a large fraction of the species' natural distribution. A total of 58.7 million of autosomal single nucleotide polymorphisms (SNPs) were detected. We find a hierarchical population structure with four distinct genetic lineages on the mainland and one on Hainan Island recapitulating current subspecies designations. The five subspecies are estimated to have diverged between 116 and 45 thousand years ago, but with recent gene flow among some groups. Consistent with the expectation of a larger body size in colder climates and a smaller body size in warmer climates (Bergman's rule), both the northernmost RM lineage (subspecies, *M. m. tcheliensis*), which exhibits the largest body size of all Chinese RMs, and the southernmost RM lineage (subspecies, *M. m. brevicaudus*), which exhibits the smallest body size of all Chinese RMs, are featured with positively selected genes responsible for skeletal development. In addition, two candidate selected genes (*Fbp1*, *Fbp2*) found in *M. m. tcheliensis* are involved in gluconeogenesis, which might play a key role to maintain a stable blood glucose levels during starvation when food resources are scarce in winter. The tropical subspecies *M. m. brevicaudus* is also characterized by positively selected genes related to cardiovascular function and response to temperature stimuli, which are potentially involved in adaptation to tropical climates. We further delineated 118 RM SNPs matching human disease-causing variants with 82 being subspecies-specific. The data presented herein provides a reference resource for the choice of RMs when carrying out biomedical experiments. The unexpected demographic history of Chinese RMs, coupled with their history of local adaption offers new insights into the evolution of RMs and provides valuable baseline information for biomedical research.

Keywords: *Macaca mulatta*, population genomics, adaptive selection, biomedical model

Introduction

Understanding how species evolve and adapt to their environments is an essential question in evolutionary biology. Rhesus macaques (RMs, *Macaca mulatta*) are, after humans, the world's most widely distributed primates [1-5], occupying a vast geographic distribution spanning from Afghanistan to the Chinese shore of the Pacific Ocean and south into Myanmar, Thailand, Laos and Vietnam [5]. As the most widely distributed nonhuman primate species, RMs occupy diverse ecological landscapes and habitats, making them an interesting model to address questions about how species evolve and adapt to local environmental variation, including characterizing the genomic architecture of adaptation to habitat, climate and other biotic and abiotic factors. Yet, despite much work on primate comparative genomics, very few population genomic studies have been carried out on wild RMs [6, 7]. Importantly, as RMs are widely used as a primate model in physiological, psychological and cognitive studies [8-10], knowledge about their genomic architecture could improve and refine biomedical research [10] as the genomic composition of experimental animals can have a considerable influence on the outcome of experiments [11, 12]. Therefore, information on the genomic diversity not only of captive, but also of wild RMs, that could become a genomic resource for future utilization in medical research, is essential.

In biomedical research, two main RM populations (Indian and Chinese) are recognized [6, 13]. They diverged from each other ~162 thousand years ago (kya) and are characterized by extensive differences in morphology, behavior, ecology, physiology, reproduction, and disease progression [6, 13-19]. In 1978 India banned all RM exports to breeding centers across the world, thus curtailing the availability of wild Indian RMs and subsequently increasing the demand for Chinese RMs in biomedical research, thereby making a detailed characterization of genetic variants from Chinese RMs crucial for biomedical usage of this species.

To date, the genomes of 133 captive RMs from eight colonies have been sequenced, however, 124 of them are of Indian-origin and only nine individuals were presumed to be of Chinese origin [6]. Recently, Zhong *et al.* [7] reported genomic variation in 26 Chinese captive RMs identifying ~46 million (M) single nucleotide polymorphisms (SNPs). Nevertheless, most of the RM genetic variation known to date is limited to captive populations which may contain composite genotypes due of admixture among animals of different and unclear origin [20]. Here

we present the first attempt to survey the geo-referenced genomic diversity in wild Chinese RM populations, which is the largest extant population of the species. The current effective population size of Chinese and Indian RM was estimated to be approximate 240,000 and 17,000 individuals, respectively, indicating that the Chinese RMs are likely to harbor substantially more genomic diversity compared to their Indian conspecifics [13]. Therefore, this population genomic survey of 81 RMs originating from 17 wild locations across China including phylogenetic and demographic analyses, as well as genome-wide selection scans, corresponds to the most comprehensive characterization of RM genetic diversity to date and aimed at characterizing the processes leading to the extant patterns of variability, as well as identifying the potential implications for the use of these populations in biomedical research.

Results and Discussion

Genetic diversity, phylogeny and population structure

Blood and tissue samples from 79 wild-born RMs, representing five subspecies [21, 22], were collected at 17 sites in China (*M. m. tcheliensis*: TH; *M. m. littoralis*: AH, FJ, HB, GX, GZ; *M. m. brevicaudus*: HN; *M. m. lasiotis*: SX, SC1, SC2, SC3, SC4; *M. m. mulatta*: YN1, YN2, YN3, YN4, YN5; Fig. 1a). Genome sequences of two additional Chinese RMs (CR1 and CR2) were retrieved from NCBI [9, 23, 24]. Re-sequencing was at a high average depth of 27.79±5.31× for ten individuals and a moderate average depth of 9.99±1.05× for the remainder (n=71), with an overall average genome coverage of 93.77% of the RM reference (Mmul_8.0.1, Supplementary Table 1). A total of 58,682,158 autosome SNPs were identified in these 81 wild Chinese RMs （Supplementary Table 2), and the genetic diversity measured by segregating sites (Watterson's θ, S) and observed genetic diversity (π) is 0.00342 and 0.00228, respectively（Table 1). The number of SNPs (all positions with differences to the genome reference) per individual ranged from 6.4 to 9.8 M (mean of 8.54 M; Supplementary Fig. 1 and Supplementary Table 3). Among all detected SNPs, 7,270,577 were shared among all subspecies and 25,951,399 were shared by at least two subspecies, with the remaining SNPs confined to a single subspecies (Supplementary Fig. 2a). For each subspecies, the subspecies-specific SNPs (ssSNPs) ranged from 870,488 to 9,230,057 and the non-synonymous ssSNPs varied from 4,788 to 34,174 (Supplementary Fig. 2a, b). Among

Chinese RM subspecies, *M. m. mulatta* had the highest heterozygosity ($2.08\times10^{-3}\pm4.42\times10^{-5}$), followed by *M. m. lasiotis* ($1.82\times10^{-3}\pm1.58\times10^{-4}$) and *M. m. littoralis* ($1.78\times10^{-3}\pm1.37\times10^{-4}$). The lowest heterozygosity rates were found in *M. m. brevicaudus* ($1.60\times10^{-3}\pm1.40\times10^{-4}$) and *M. m. tcheliensis* ($1.32\times10^{-3}\pm3.14\times10^{-4}$) (Supplementary Fig. 3).

We reconstructed a neighbor-joining (NJ) tree for Chinese RMs based on autosomal SNPs, using Indian RMs and *M. sylvanus* as outgroups (Fig. 1b and Supplementary Fig. 4). Individuals from *M. m. lasiotis*, *M. m. brevicaudus* and *M. m. tcheliensis* form monophyletic lineages respectively, while *M. m. mulatta* and *M. m. littoralis* are paraphyletic. Next, we performed a population structure analysis using STRUCTURE (version 2.3.4) [25], which estimates individual ancestry and admixture proportions assuming K ancestral populations. Plots of ΔK generated from STRUCTURE results indicated five genetic clusters present in the full data set (Fig. 1b and Supplementary Fig. 5). A principal component analysis (PCA) corroborated the division of Chinese RMs into five groups. The first eigenvector separated *M. m. mulatta* and *M. m. lasiotis* from *M. m. tcheliensis*, *M. m. littoralis* and *M. m. brevicaudus* (variance explained = 7.24%, Tracy-Widom P = 4.78×10^{-44}), and the second eigenvector further separated *M. m. tcheliensis*, *M. m. littoralis* and *M. m. brevicaudus* (variance explained = 5.69%, Tracy-Widom P = 4.21×10^{-27}) (Fig. 1c, Supplementary Table 4). The division of Chinese RMs into five geographic lineages supports the former taxonomic division of Chinese RMs into five subspecies [21, 22]. *M. m. mulatta* (YN1-5) and *M. m. lasiotis* (SC1-4, SX) form the pan-western populations of Chinese RMs, with both subspecies inhabiting the montane Tibetan Plateau regions with an altitude ≥1500 meters above sea level in western China and separated from each other by the Yangtze River. *M. m. littoralis* (AH, FJ, HB, GX, GZ), *M. m. tcheliensis* (TH) and *M. m. brevicaudus* (HN) occur in the eastern coastal lowland of China and form the pan-eastern population. *M. m. tcheliensis* from the Taihang Mountains area (TH) is the northernmost (34°54'–35°16' N; 112°02'–112°52' E), while *M. m. brevicaudus*, restricted to Hainan Island, is the most southern Chinese RM subspecies.

Demographic and phylogeographic history

The estimated effective population sizes, based on the number of segregating sites (S) and observed genetic diversity (π) is approximately 85,250 and 57,000 for Chinese RMs (Table 1). In

order to infer the ancient demographic history of Chinese RMs, we applied a pairwise sequential Markovian coalescent (PSMC) [26] analysis using ten RM individuals with an average sequencing coverage depth higher than 20× (one individual of *M. m. tcheliensis* and one of *M. m. brevicaudus*, two of *M. m. lasiotis*, three of *M. m. littoralis* as well as three individuals of *M. m. mulatta*). The inferred PSMC trajectories were very similar for all analyzed individuals throughout most of the species' history until ~110 kya reflecting the species' cohesiveness (Fig. 2a). The ancient demographic history of RMs is marked by population fluctuations following the glacial periods during the Pleistocene [27]. Approximately 1200-800 kya all Chinese RMs experienced a population reduction at the time of the Xixiabangma Glaciation (XG), followed by an expansion during the Mid-Pleistocene inter-glaciation (800-200 kya). This expansion was then interrupted by the Penultimate Glaciation (PG, 200-130 kya) when suitable habitat might have been lost leading to a population decline [27]. PSMC analyses also suggested that while *M. m. mulatta* and *M. m. lasiotis* stabilized with effective population sizes somewhere around 100 kya, *M. m. tcheliensis*, *M. m. littoralis* and *M. m. brevicaudus* went through a dramatic population increase and a subsequent bottleneck reaching a stable effective population size somewhere around 60-50 kya (Fig. 2a). Interestingly, the demographic inference by Xue et al. [6] derived from genomic data of a single Chinese RM (CH_37945) from AH (*M. m. littoralis*) qualitatively resembled the demographic trajectory of *M. m. littoralis* presented herein.

To further describe the divergence process among the five Chinese RM subspecies, we also employed the SVDquartes approach [28-31] that takes incomplete lineage sorting into account. The obtained phylogenetic tree suggests a "step-by-step" divergence of the five subspecies. Accordingly, the *M. m. mulatta* lineage diverged form that of the remaining Chinese RMs firstly and then the *M. m. lasiotis* diverged from the ancestral lineage of pan-eastern RMs (*M. m. tcheliensis*, *M. m. littoralis* and *M. m. brevicaudus*). Subsequently, *M. m. brevicaudus* diverged from the ancestor of *M. m. tcheliensis* and *M. m. littoralis*, the divergence of which occurred lastly (Supplementary Fig. 6). Under this "step-by-step" divergence scenario, we performed the joint site frequency spectrum (SFS) based approach implemented in *fastsimcoal2* [32] to model demographic fluctuations, respective divergence times and gene flow events among the five RM subspecies. Following the divergence between the ancestral lineages of Indian and Chinese RMs (~162 kya), the ancestor of *M. m. mulatta* diverged from the remaining Chinese RMs ~115.8 kya

near the end of the last interglacial (Fig. 2b) [6, 13]. Subsequently, *M. m. lasiotis* diverged from the ancestral lineage of pan-eastern RMs ~111.9 kya. The divergence time between *M. m. brevicaudus* and the ancestor of *M. m. tcheliensis* and *M. m. littoralis* was estimated at ~70.4 kya, while the divergence between the latter two occurred ~ 44.8 kya at the beginning of the period leading to the last glacial maximum [33,34]. Interestingly, the coalescence analysis revealed a bottleneck in population size (N_{A1} =2.0k and N_{A2} =1.5k) of pan-eastern RMs during the period from 111.9 kya to 44.8 kya (Fig. 2b), which coincided with the population decline of pan-eastern RMs since approximate 100 kya revealed by PSMC analyses. However, the population growth that occurred in pan-eastern RMs after the bottleneck has not been detected by PSMC analyses, probably because PSMC is less accurate when reconstructing recent histories within ~100 kya [6]. Gene flow after the divergence of subspecies occurred among almost all five lineages (Fig. 2b and Supplementary Table 5).

A previous study of mitochondrial DNA identified two major haplogroups dividing Chinese RMs into a western and an eastern clade. Modern Chinese RMs were thought to have undergone a northward expansion while entering China via two possible routes: the first into the western mountains and the second following the eastern coast [35]. Our evolutionary model, however, suggests a "step-by-step" colonization process of RMs in China (Fig 2c). After the divergence from the Indian population (~162 kya) [6, 13], the ancestor of Chinese RMs colonized the Tibetan Plateau from southwestern China, and then experienced a range expansion north and eastwards. The pan-western population (*M. m. mulatta* and *M. m. lasiotis*) inhabited the western montane region in China, while the pan-eastern population (*M. m. tcheliensis*, *M. m. littoralis* and *M. m. brevicaudus*) entered the eastern coastal region. Barriers such as the Yellow, Yangtze and Pearl rivers and open sea (Fig. 1a) led to further differentiation, limiting gene flow among them. Water bodies and mountains could therefore be described as driving the formation of a habitat 'lattice' with the different subspecies of RMs occupying different grids in the lattice.

Signatures of selection and local adaptation

The wide distribution of Chinese RMs and their respective contrasting habitat types, as well as their wide use in biomedical studies, makes them an important case study for the analysis of signatures of local adaptation to divergent selective pressures [36-38]. We identified putative

targets of selection by carrying out pair-wise comparisons between RM subspecies inhabiting the most different environments to increase the chance of finding selection signatures, i.e., *M. m. tcheliensis* that occurs in the northernmost range of the species under cold conditions, and *M. m. brevicaudus* that inhabits the southernmost range of the species, a tropical island. For each analysis, we compared the five subspecies using the fixation index (F_{ST}) and genetic diversity (θ_π), calculated on 50kb long sliding windows (Fig. 3 and Supplementary Figs. 7-12). The top 5% of the windows with the largest F_{ST} and θ_π ratios ($\theta_\pi 2$ /$\theta_\pi 1$) in each pair-wise comparison were considered to be potentially under positive selection. For each subspecies, we identified the intersection of potential selective-sweep regions generated by all the pair-wise comparisons between a subspecies and each of the other subspecies (four pairwise comparisons in each case) (Supplementary Fig. 7). We used these consistent selective-sweep regions for further analyses, as they represent robust putative positively selected regions. The sizes of candidate selective-sweep regions ranged from 0.100 Mb to 11.075 Mb and the number of genes located in these regions, which are expected to represent targets of selection for each subspecies, varied from 6 to 176 in different subspecies (Supplementary Table 6).

M. m. tcheliensis from the Taihang (TH) Mountains area is the northernmost population of the species. The TH Mountains are characterized by a continental monsoon climate, and conditions for RMs are harsh during winter and early spring with extreme cold temperatures of –14°C [39]. Food resources are limited and consist mainly of barks, twigs, roots of crops and withered grass, thus, all sources are high in fiber, but low in energy and nutritional value [40, 41]. Therefore, *M. m. tcheliensis* suffers from starvation due to food shortage during winter and early spring. In starvation, blood glucose levels are maintained by gluconeogenesis through which glucose are converted from other molecules, such as amino acids and lactic acid [42]. Among the 176 genes found to be under positive selection in *M. m. tcheliensis*, two (*Fbp1*, *Fbp2*, modified Fisher Exact *P*=1.90E-02; Fig. 3c, d; Supplementary Table 7) are enriched in the gene ontology (GO) term “fructose 1, 6-bisphosphate 1-phosphatase activity”. These two genes encode for fructose-1, 6-bisphosphatase 1 and fructose-1, 6-bisphosphatase isozyme 2 which catalyze the hydrolysis of fructose 1, 6-bisphosphate and play a rate-limiting role in gluconeogenesis. Furthermore, in starved zebrafish it was shown that the expression of *Fbp1* was significantly unregulated in brain and liver tissues [43]. The positive selection genes are also enriched in other

terms and pathway related to gluconeogenesis, including KEGG pathway "Fructose and mannose metabolism" (modified Fisher Exact *P*=4.35E-02) and GO terms "hexose biosynthetic process", "monosaccharide biosynthetic process" and "cellular carbohydrate biosynthetic process" (modified Fisher Exact *P*=3.36E-02, *P*=4.64E-02 and *P*=2.65E-02; Supplementary Table 7). Our findings suggest that the regulation of gluconeogenesis might be a mechanism of *M. m. tcheliensis* to adapt to food shortage in winter.

According to Bergman's rule, animals living in cold climates tend to have larger body sizes compared to their relatives in warm climates (i.e. they have a lower surface area to volume ratio), so they radiate less body heat per unit of mass [44]. Consistent with this expectation, among all RM subspecies, *M. m. tcheliensis* exhibits the largest body size and mass, the longest forearm length and the largest head and chest circumference (Fig. 3b and Supplementary Table 8) [40, 45]. Among the consistent signatures of positive selection identified in *M. m. tcheliensis* (176 genes), we found signatures of selective sweeps in eight genes linked to limb morphogenesis or skeletal system development (Supplementary Table 6). Among these genes, *Fto* and *Rpgripl1* play an essential role in postnatal growth of mammals [46]. Mice lacking *Fto* completely display immediate postnatal growth retardation with shorter body length, lower body weight, and lower bone mineral density than control animals [47]. Furthermore, *Sox5* and *Sox6* (Fig. 3c, d) play an essential role in synovial joint morphogenesis via promoting both growth plate and articular chondrocyte differentiation [48]. Mutations in *Atp6v0a4* could cause developmental delay and delayed closure of the anterior fontanelle in human [49], while expression of *Ext2* enhances the bone formation in mice [50] These genes involved in the growth and development of the skeletal system and appendages are likely contributors to the larger body size of *M. m. tcheliensis*, and represent an undescribed adaptive pathway for primates living in colder climates.

In contrast, *M. m. brevicaudus* inhabits the tropical island of Hainan (HN) where it copes with a mean annual temperature of 24°C. *M. m. brevicaudus* has the smallest body size, the smallest body mass, and the shortest tail among RM subspecies [45]. As described above, they radiate more body heat per unit of mass (Bergman's rule) [44]. We found 127 putatively selected genes in *M. m. brevicaudus* (Supplementary Table 6), four of which were found to be enriched in GO term "Bone morphogenetic protein (BMP) signaling pathway" (modified Fisher Exact *P*=4.65E-02; Supplementary Table 9) and two genes were found to be enriched in GO term

"I-SMAD binding (modified Fisher Exact *P*=4.65E-02; Supplementary Table 9)". BMP and I-SMAD signaling pathways are involved in the development of bones and the skeleton [51, 52]. Mutations in *Axin1*, a gene of the I-SMAD pathway, cause kinked tails in mice [53]. In *M. m. brevicaudus*, we found two non-synonymous mutations in this gene (A674G, T656I) (Supplementary Fig. 13 and Supplementary Table 10, 11).

Additionally, putatively selected genes in *M. m. brevicaudus* (Fig. 3c, d, Supplementary Table 6) were also involved in GO terms related to cardiovascular system and blood circulation. For example, *Aggf1* related to GO term "blood vessel morphogenesis" and *Ctnna3* related to GO term "regulation of heart rate by cardiac conduction". The up-regulated *Aggf1* expression is capable of increasing blood flow in mouse hindlimb [54]. In addition, *Hspa4*, heat shock 70kDa protein 4, is directly involved in GO term "response to temperature stimulus". We thus hypothesize that the cardiovascular system of *M. m. brevicaudus* might play an important role in stabilizing body temperature, assisted by blood flow through different body parts requiring good fluidity and vascular permeability to transfer heat out of the body [55]. Testing these hypotheses needs further functional assays, however, these genes, together with the positively selected genes identified in *M. m. tcheliensis*, are known to be relevant to human physical function, and thus are likely of importance in the adaptation of Chinese RMs to different climate conditions.

Both coding and non-coding changes could contribute to local adaptations of organisms [56]. To further investigate the adaptive mechanism of *M. m. tcheliensis* and *M. m. brevicaudus* to the opposite climates (cold versus hot), we focused on SNPs in the gene regions of above described candidate genes. A total of 5817 SNPs were found with significant differences at the 5% level in the distributions of genotypes between these two subspecies, and 10 SNPs were non-synonymous variants (Supplementary table 10 and 11). In *M. m. tcheliensis*, non-synonymous mutations were found in the coding regions of *Atp6v0a4* (R667Q), *Ext2* (I363M), *Fto* (N10S) and *Rpgrip1l* (R1281Q) (Supplementary table 11 and Supplementary Fig. 13), implying that selection might has acted on protein sequence changes. No non-synonymous changes were detected in *Fbp1*, *Fbp2*, *Sox5* and *Sox6*. However, SNPs are located in the 1kb up/downstream, 5' and 3' UTR, and intronic regions of these genes (Supplementary table 10), indicating selection on non-coding regulatory variants. Correspondingly, non-synonymous mutations in *Aggf1* (H343Y), *Axin1* (A674G, T656I), *Hspa4* (I782V) and *Ctnna3* (V551I, T577M) were revealed for *M. m.*

brevicaudus (Supplementary table 11 and Supplementary Fig. 13).

Besides the genes related to the adaptation to various climate conditions, we also found signatures of positive selection in genes related to the nervous system. In *M. m. tcheliensis* the 176 identified candidate genes are enriched in GO term “synaptic” (modified Fisher Exact *P*=1.38E-02; Supplementary Table 10) with eight genes, and two of these gene, *Gabra2* and *Chrm2* are associated with alcohol dependence [57]. For *M. m. brevicaudus*, 18 putatively selected genes related to nervous system development were found. For example, *Dcc* is reported to be required for long-term potentiation and memory [58]. *Auts2*, one of the eight putatively selected genes in *M. m. lasiotis*, has been shown to regulate neuronal migration, and mutations in this gene cause mental dysfunction in human [59] (Supplementary Table 7). Our findings suggest that RM subspecies have experienced different adaptative processes in the nervous system and respective genomic differences should be taken into account when animals are selected for neurobiological research.

Disease–causing variants and implication for biomedical research

Given the large evolutionary similarity between macaques and humans, human diseases are better modeled in RMs than in many other animals. Thus, variants in RMs that match to orthologous human variants annotated as ‘pathogenic’ are of particular interest. We examined presumed homologous Chinese RM SNPs in the human genome and a total of 34,850,330 RM SNPs analyzed in this study were successfully identified in the human genome (hg19). Among these SNPs, 118 variants matched human variants with the accordant reference alleles and alternative alleles were annotated as ‘disease causing’ in HGMD or pathogenic in ClinVar. These 118 RM SNPs affect genes that cause specific human diseases including acromesomelic dysplasia maroteaux type, anonychia, atransferrinemia, blau syndrome, Carcinoma of colon, Charcot-Marie-Tooth disease, deafness, early infantile epileptic encephalopathy 7, glycogen storage disease and others (Supplementary Table 12). Among these 118 SNPs，only seven pathogenic SNPs are shared by all five subspecies, while 82 are subspecies-specific (Fig. 4c, Supplementary Table 12). For example, the SNP rs116229331 in the gene *Unc13d* (human Chr17: 73836585C>T), known to cause juvenile idiopathic arthritis in humans [60], has a RM homologue (RM Chr16: 69559126 C>T, Fig. 4a) that is present in *M. m. tcheliensis*, *M. m.*

brevicaudus and *M. m. littoralis*, but absent in *M. m. lasiotis* and *M. m. mulatta*. Another pathogenic variant (rs397514345, human Chr3: 15686724 A>C) in the *Btd* gene is involved in biotinidase deficiency [61]. Its homologous RM variant (RM Chr2: 172277927 A>C, Fig. 4a) is found only in *M. m. lasiotis* and *M. m. mulatta.* In addition, we also identified 16 non-synonymous SNPs in the *Noca3* gene, which encodes a protein that modulates the replication and transcriptional reactivation of HIV-1 during virus latency [62] (Fig. 4b). Ten of these 16 non-synonymous SNPs are private to one subspecies (Supplementary Table 13). The effects of these variants on HIV-1 replication and reactivation are unknown and need further investigation, but the high number of mutations suggests a complex response of the host to the virus.

Overall, these findings suggest that the genomic architecture of Chinese RMs used in biomedical research and their geographic origin could strongly influence the outcome of biomedical experiments and should be taken into account when using Chinese RMs in clinical and neurobiological research. Unfortunately, genome wide screening of RMs used in biomedical research is so far only rarely conducted and uncharacterized animals are most often used. Importantly, individuals from all five Chinese RM subspecies are used in biomedical research [63, 64]. Combined with our data, nine of the 26 captive Chinese RMs reported by Zhong *et al.* [7] were found to cluster with *M. m. littoralis*, 16 with *M. m. lasiotis* and one with *M. m. mulatta* (Fig. 4d). Thus, the data and results presented here provide the basis to trace the origin of captive RMs and to allow for the selection of appropriate animal models when testing for particular diseases, and are thus a significant contribution to the “3Rs” principle, which aims to reduce, refine, and replace experimental animals [65].

Conclusion

We present the first description of the evolutionary history and genomic variation of geo-referenced wild RMs throughout China, including scenarios on potential functions of this variation in adaptation to local environments. This genomic resource represents a valuable contribution to the understanding of the biology and evolution of a highly successful and important biomedical research species. In particular, it is important to note that due to the difference in evolutionary history of the subspecies identified here, it can be expected that animals originating from different regions may react differently to experimental tests, and thus their background needs to be assessed beforehand [10]. Our results highlight the importance that genome typing can play in biomedical research where animal origins are uncertain, and the resources generated here provide a baseline for genomic assessment of biomedical research populations, genetic resource conservation and for refined usage of RMs in future research.

Materials and Methods

Ethics statement

The methods were carried out in accordance with the approved guidelines of the Good Experimental Practices adopted by the Institute of Zoology, Chinese Academy of Sciences (CAS). All experimental procedures and animal collection were conducted under the supervision of the Committee for Animal Experiments of the Institute of Zoology, Chinese Academy of Sciences.

Sample Collection and Sequencing

Samples from 79 individuals with information about geographic origin were collected from 17 local wildlife rescue center, which covered most of the species' range in China. Muscle samples were collected from deceased individuals and the blood samples were taken during routine physical examinations. Total genomic DNA was extracted from blood or tissue samples using standard phenol/chloroform methods. For each individual, ~3 μg DNA was sheared into fragments of 500 bp with the Covaris system. DNA fragments were then processed and sequenced using the Illumina HiSeq 2000 and 2500 platform. Furthermore, published genomic data for two individuals were download form NCBI [9,23] and filtered using the same conditions. Raw reads were first filtered with the following criteria: (1) reads with unidentified nucleotides (N) exceeded 10% were discarded, (2) reads with the proportion of low quality base (phred quality <=5) larger than 50% were discarded. After the quality control, a total of 2,736.91 Gb of high quality sequences with 22.53 billion pair-end reads (100 or 125 bp) were generated.

Sequence Data Pre–processing and Variant Calling

High-quality sequence reads were mapped to the macaque reference genome, Mmul_8.0.1 [66], using the Burrows–Wheeler Aligner (BWA) (0.7.10-r789) [67]. Sequence Alignment/Map (SAM) format files were imported to SAMtools (v0.1.19) [68] for sorting and then imported to Picard (http://broadinstitute.github.io/picard/, version 1.118) for removing duplicated reads. To improve the quality of sites reported, we performed SNP calling following GATK's best practice, version 3.3–0 (GATK, RRID: SCR_001876) on autosomal sites only [69]. We get the GVCF file for each individual using the HaplotypeCaller method in GATK and then using the GATK with the

GenotypeGVCFs-based method to get the population GVCF for all samples. After SNP calling, we applied the command "SelectVariants" and "VariantFiltration" to exclude Indel and potential false-positive variant calls. All the SNPs were annotated by ANNOVAR (v2013-06-21) [70] (Supplementary Table 2). For each individual the heterozygosity was calculated as heterozygous SNP rate across the whole genome (Supplementary Table 3).

Genetic Diversity and Structure Analysis

A neighbor-jointing (NJ) tree was constructed for the 81 individuals based on the autosomal genome data using the software TreeBeST. The bootstrap was set to 1,000 times to assess branch support, with the genome information of Indian RMs and *M. sylvanus* as outgroups. FigTree (http://tree.bio.ed.ac.uk/software/figtree/, v1.4.0) was used to visualize the phylogenetic tree (Fig. 1b and Supplementary Fig. 4). Population structure analysis was performed using the software STRUCTURE 2.3.4 [25], which estimates individual ancestry and admixture proportions assuming K ancestral populations. We ran STRUCTURE five times to assess convergence and tested the number of genetic clusters (K) from 2-9 (Supplementary Fig. 5). We also carried out a principle component analysis (PCA) using the smartPCA program from the Eigensoft package (v5.0) [71]. To determine the significance level of principal components, a Tracy-Widom test was done after the PCA (Supplementary Table 4). Decay of linkage disequilibrium against physical distance for the different populations was calculated using the Haploview software [72] with the maxdistance set as 500kb (Supplementary Fig. 14).

Demographic and Divergence Inference Using PSMC and Fastsimcoal2

The PSMC model [26] was used to estimate the population histories from the individual genomes (sex chromosomes excluded) with the following parameters: –N25 –t15 –r5 –p '4+25×2+4+6'. We chose a generation length of 11 years and a mutation rate per generation (μ) of 1.0×10^{-8} (for the rationale to use these values see [6, 73]). To ensure the quality of consensus sequences, we used data of ten individuals with an average coverage >20× (22.20-34.32×).

We used PAUP* 4.0a142 [30] for Linux to run SVDquartets to estimate the branching pattern among the five subspecies with the following command: SVDQuartets SpeciesTree=yes bootstrap evalQuartets=all seed=0 nthreads=40. The joint site frequency spectrum (SFS) approach

implemented in *fastsimcoal2* [32] was performed to model more recent demographic fluctuations and respective divergence times based on the species tree estimation by SVDquartets. To mitigate the effect of linkage disequilibrium, we took one SNP every 10kb, and then SNPs located 10 kb away from genes, were used to convert SFS. The parameters used in *fastsimcoal2* were: -N 100000 (max. number of simulations), -L 40 (max. number of EM cycles), - M 0.001 (min. relative difference in parameter values for the stopping criterion). Two hundred replicates from different initial conditions were run to ensure convergence. Migration rates were ignored between subspecies which have no direct connection. The outputs of this scenario were processed with arlsumstat to obtain distributions of various summary statistics (Supplementary Table 5).

Positive Selection

To identify genomic regions that may have been subject to selection for each subspecies inhabited in different habitats, we scanned the genome using one-to-one pair-wise comparisons between all five subspecies. We calculated the genome-wide distribution of F_{ST} values [74] and θ_π ratios for each pairwise comparison among five RM subspecies. We calculated θ_π for each population and the F_{ST} between the two populations in each comparison using VCFtools [75] with a genome-wide sliding window strategy (50-kb in length with 25-kb step). The F_{ST} values were Z-transformed and the log value of θ_π ratio ($\theta_\pi 2$ /$\theta_\pi 1$) was estimated. Candidate regions under positive selection were extracted based on the top 5% of log-odds ratios for both Z (F_{ST}) and log (θ_π–ratio). Finally, for each subspecies we used the intersection of putatively selected regions generated by all the pair-wise comparisons with other subspecies as the candidate regions under positive selection (i.e. consistent signatures of selective sweeps). Genes located in these regions are expected to represent targets of selection. Functional classification and enrichment analysis of GO categories and KEGG pathways for these candidate genes were performed using DAVID (v6.8) [76]. The modified Fisher Exact *P*-value cut off was 0.05. Chi-square and *P*-values for the allele frequencies in *M. m. tcheliensis* vs. *M. m. brevicaudus* for the re-sequenced SNPs from the candidate genes were assessed with the Haploview program [72].

Genomic divergence and implication for biomedical research

A total of 118 out of 58,682,158 RM SNPs analyzed in this study were successfully mapped to human reference sequence version hg19 (GRCh37) using liftOver (https://genome.ucsc.edu/cgi-bin/hgLiftOver) and were annotated as 'disease causing' in HGMD (version 2015.1) or pathogenic in ClinVar (downloaded 25/02/2018) (Supplementary Table 12).

For more details of methods please see supplementary notes in Supplementary Material.

Data Access

All data generated from this study have been submitted to the NCBI Sequence Read Archive (SRA) under BioProject PRJNA345528.

Competing interests

The authors declare that they have no competing interests.

Acknowledgments

This project was sponsored by the following grants: Ming Li (Key Project of National Natural Science Foundation of China, 31530068; Strategic Priority Research Program of the Chinese Academy of Sciences, XDPB0202 and XDA19050202; and National Key R&D Program of China, 2016YFC0503200); Zhijin Liu (Natural Science Foundation of China, 31471989). The authors thank Baoguo Li, Meng Yao, Songtao Guo, Jiqi Lu, Zhenlong Wang, Xuelong Jiang, Tao Meng and Qihai Zhou for their help in sampling; Daniel Pitt, Quan Kang, Qi Wu and Qi Pan for their assistance in data analysis.

Author contributions

M. L., Z. L. and M. B conceived the study and designed the project. Z. L., X. T., P. O., X. Z., L. Z. and S. T. managed the project, performed the analyses and wrote the manuscript. Z. L., B. S. and H. X. prepared samples. Z. L., X. T. and P. O. performed genetic analyses. Z. L., X. T., P. O., B. R., L. Z., G. L., Z. Y., Z. P., Z. X., C. R., M. B. and M. L. discussed the data. Z. L. and X. T. wrote the manuscript with contributions from P. O., B. W., H. X., W. Z., C. R., M. B. and M. L.;

all authors contributed to data interpretation.

Supplementary Material

Supplementary information, figures S1-S14, tables S1-S13, and notes are available on line.

References

1. Moreno-Estrada A, Gignoux CR, Fernández-López JC et al. Human genetics. The genetics of Mexico recapitulates Native American substructure and affects biomedical traits. Science 2013; **344**:1280–1285.
2. Allentoft ME, Sikora M, Sjögren KG et al. Population genomics of Bronze Age Eurasia. Nature 2015; **522**:167–172.
3. Sudmant PH, Rausch T, Gardner EJ et al. An integrated map of structural variation in 2,504 human genomes. Nature 2015; **526**:75–81.
4. Maestripieri D. Macachiavellian intelligence: How rhesus macaques and humans have conquered the world. 2007. The University of Chicago Press, Chicago.
5. Zinner D, Fickenscher GH, Roos C. Family Cercopithecidae (Old World Monkeys). Handbook of the Mammals of the World. 2013; Pp. 550-753 in: Mittermeier RA, Rylands AB, Wilson DE. eds. Vol. 3. Primates. Lynx Edicions, Barcelona.
6. Xue C, Raveendran M, Harris RA et al. The population genomics of rhesus macaques (*Macaca mulatta*) based on whole genome sequences. Genome Res 2016; **26**:1651–1662.
7. Zhong X, Peng J, Shen QS et al. RhesusBase PopGateway: Genome-Wide Population Genetics Atlas in Rhesus Macaque. Mol Biol Evol 2016; **33**:1370–1375.
8. Fawcett GL, Raveendran M, Deiros DR et al. Characterization of single-nucleotide variation in Indian-origin rhesus macaques (*Macaca mulatta*). BMC Genomics 2011; **12**:311.
9. Yan G, Zhang G, Fang X et al. Genome sequencing and comparison of two nonhuman primate animal models, the cynomolgus and Chinese rhesus macaques. Nature Biot 2011; **29**:1019-1023.
10. Haus T, Ferguson B, Rogers J et al. Genome typing of nonhuman primate models: implications for biomedical research. Trends Genet 2014; **30**:482–487.
11. Flynn S, Satkoski J, Lerche N et al. Genetic variation at the TNF-alpha promotor and malaria susceptibility in rhesus (*Macaca mulatta*) and long-tailed (*Macaca fascicularis*) macaques. Infect Genet Evol 2009; **9**:769–777.
12. de Groot NG, Heijmans CMC, Koopman G et al. TRIM5 allelic polymorphism in macaque species/populations of different geographic origins: its impact on SIV vaccine studies. Tissue

Antigens. 2011; **78**:256–62.

13. Hernandez RD, Hubisz MJ, Wheeler DA et al. Demographic histories and patterns of linkage disequilibrium in Chinese and Indian rhesus macaques. Science 2007; **316**:240–243.
14. Champoux M, Higley JD, Suomi SJ. Behavioral and physiological characteristics of Indian and Chinese-Indian hybrid rhesus macaque infants. Dev Psychobiol 1997; **31**:49–63.
15. Trichel AM, Rajakumar PA, Murphey-Corb M. Species-specific variation in SIV disease progression between Chinese and Indian subspecies of rhesus macaque. J Med Primatol 2002; **31**:171–178.
16. Tosi AJ, Morales JC, Melnick DJ. Paternal, maternal, and biparental molecular markers provide unique windows onto the evolutionary history of macaque monkeys. Evolution 2003; **57**:1419–1435.
17. Smith DG. Genetic characterization of Indian-origin and Chinese-origin rhesus macaques (*Macaca mulatta*). Comp Med 2005; **55**:227–230.
18. Ferguson B, Street SL, Wright H et al. Single nucleotide polymorphisms (SNPs) distinguish Indian-origin and Chinese-origin rhesus macaques (Macaca mulatta). BMC Genomics 2007; **8**:43.
19. Kubisch HM, Falkenstein KP, Deroche CB et al. Reproductive efficiency of captive Chinese- and Indian-origin rhesus macaque (*Macaca mulatta*) females. Am J Primatol 2012; **74**:174–184.
20. Kanthaswamy S, Johnson Z, Trask JS et al. Development and validation of a SNP-based assay for inferring the genetic ancestry of rhesus macaques (*Macaca mulatta*). Am J Primatol 2014; **76**:1105–1113.
21. Fooden J. Systematic review of the rhesus macaque, *Macaca mulatta* (Zimmermann, 1780). Field Zool 2000; **96**:1–180.
22. Jiang X, Wang Y, Ma S. Taxonomic revision and distribution of subspecies of rhesus monkey (*Macaca mulatta*) in China. Zool Res 1991; **12**:241–247.
23. Fang X, Zhang Y, Zhang R et al. Genome sequence and global sequence variation map with 5.5 million SNPs in Chinese rhesus macaque. Genome Biol 2011; **12**:R63.
24. Prado-Martinez J, Sudmant PH, Kidd JM et al. Great ape genetic diversity and population history. Nature 2013; **499**:471–475.

25. Evanno G, Regnaut S, Goudet J. Detecting the number of clusters of individuals using the software STRUCTURE: a simulation study. Mol Ecol 2005; **14**:2611–2620.

26. Li H, Durbin R. Inference of human population history from individual whole-genome sequences. Nature 2011; **475**:493–496.

27. Zheng B, Xu Q, Shen Y. The relationship between climate change and Quaternary glacial cycles on the Qinghai–Tibetan Plateau: review and speculation. Quatern Int 2002; **97**:93–101.

28. Chifman J, Kubatko L. Identifiability of the unrooted species tree topology under the coalescent model with time-reversible substitution processes, site-specific rate variation, and invariable sites. J Theor Biol 2014; **374**:35-47.

29. Chifman J, Kubatko L. Quartet inference from SNP data under the coalescent model. Bioinformatics 2014; **30**:3317-3324.

30. Swofford, D, et al; PAUP*. Phylogenetic Analysis Using Parsimony (*and other methods). Version 4. Sinauer Associates, Sunderland, Massachussets. 2003.

31. Song S, Liu L, Edwards SV, Wu S. Resolving conflict in eutherian mammal phylogeny using phylogenomics and the multispecies coalescent model. Proc Natl Acad Sci USA 2012; **109**: 14942-14947.

32. Excoffier, L. Dupanloup I, Huerta-Sánchez E et al Robust demographic inference from genomic and SNP data. PLoS Genet 2013; **9**:e1003905.

33. Owen LA, Finkel RC, Caffee MW. A note on the extent of glaciation throughout the Himalaya during the global Last Glacial Maximum. Quaternary Sci Rev 2002; **21**:147–157.

34. Owen LA. Latest Pleistocene and Holoene glacier fluctuations in the Himalaya and Tibet. Quaternary Sci Rev 2009; **28**:2150–2164.

35. Wu S, Luo J, Li Q et al. Ecological genetics of Chinese rhesus macaque in response to mountain building: all things are not equal. PLoS ONE 2013; **8**:e55315.

36. Yi X, Liang Y, Huerta-Sanchez E et al. Sequencing of 50 human exomes reveals adaptation to high altitude. Science 2010; **329**:75–78.

37. Bhatia G, Patterson N, Pasaniuc B et al. Genome-wide comparison of African-ancestry populations from CARe and other cohorts reveals signals of natural selection. Am J Hum Genet 2011; **89**:368–381.

38. Zhao SC, Zheng PP, Dong SS et al. Whole-genome sequencing of giant pandas provides insights into demographic history and local adaptation. Nat Genet 2013; **45**:67–71.

39. Tian JD, Wang ZL, Lu JQ, Wang BS, Chen JR. Reproductive Parameters of Female Macaca mulatta tcheliensis in the Temperate Forest of Mount Taihangshan, Jiyuan, China. Am J Primatol 2013; **75**:605–612.

40. Zhao X, Zhang H, Lv X et al. Survey and research of morphological characters of monkeys (*Macaca mulatta*) in the Taihang Mountains. J Henan Nor Uni 1989; **62**:120–125.

41. Lu JQ, Hou JH, Wang HF, Qu WY. Current status of *Macaca mulatta* in Taihangshan Mountains Area, Jiyuan, Henan, China. Int J Primatol 2007; **28**:1085–1091.

42. Sadava DE, Heller HC, Orians GH, Purves WK, Hillis DM. Life: The Science of Biology, 8th edn. Macmillan, New York; 2008.

43. Drew RE, Rodnick KJ, Settles M et al. Effect of starvation on transcriptomes of brain and liver in adult female zebrafish (Danio rerio). Physiol Genomics 2008; **35**:283–295.

44. Bergmann C. Über die Verhältnisse der Wärmeökonomie der Thiere zu ihrer Grösse. Göttinger Studien 1847; **3**:595–708.

45. Zhang P, Lyu MY, Wu CF et al. Variation in body mass and morphological characters in Macaca mulatta brevicaudus from Hainan, China. Am J Primatol 2016; **78**:679–698.

46. Jevsinek Skok D, Kunej T, Kovac M et al. FTO gene variants are associated with growth and carcass traits in cattle. Animal Genetics 2016; **47**: 219–222.

47. Gao X, Shin YH, Li M, Wang F, Tong Q, Zhang PM. The Fat Mass and Obesity Associated Gene FTO Functions in the Brain to Regulate Postnatal Growth in Mice. PLoS ONE 2010; **5**: e14005.

48. Dy P, Smits P, Silvester A et al. Synovial joint morphogenesis requires the chondrogenic action of Sox5 and Sox6 in growth plate and articular cartilage. Dev Biol 2010; **341**:346–359.

49. Greally MT, Kalis NN, Agab W et al. Autosomal recessive cutis laxa type 2A (ARCL2A) mimicking Ehlers‐Danlos syndrome by its dermatological manifestations: Report of three affected patients. Am J Med Genet A 2014; **164A**:1245–1253.

50. Morimoto K, Shimizu T, Furukawa K, Morio H, Kurosawa H, Shirasawa T. Transgenic expression of the EXT2 gene in developing chondrocytes enhances the synthesis of heparan

sulfate and bone formation in mice. Biochem Biophys Res Commun 2002; **292**: 999–1009.

51. Salazar VS, Gamer LW, Rosen V. BMP signaling in skeletal development, disease and repair. Nat Rev Endocrinology 2016; **12**:203–221.
52. Bragdon B, Moseychuk O, Saldanha S et al. Bone Morphogenetic Proteins: A critical review. Cell Signal 2011; **23**: 609–620.
53. Ruvinsky A, Flood WD, Costantini F. Developmental mosaicism may explain spontaneous reappearance of the AxinFu mutation in mice. Genesis 2001; **29**: 49-57.
54. Lu QL, Yao YH, Yao YF et al. Angiogenic Factor AGGF1 Promotes Therapeutic Angiogenesis in a Mouse Limb Ischemia Model. PLoS ONE 2012; 7: e46998.
55. González-Alonso J. Human thermoregulation and the cardiovascular system. Exp Physiol 2012; **97**:340–346.
56. Meadows JRS, Lindblad-TohK. Dissecting evolution and disease using comparative vertebrate genomics. Nat Rev Genet 2017; 18, 624–636.
57. Dick DM, Bierut LJ. Curr Psychiatry Rep 2006; **8**: 151.
58. Horn KE, Glasgow SD, Gobert D et al. DCC expression by neurons regulates synaptic plasticity in the adult brain. Cell Rep 2010; **31**:173-185.
59. Hori K, Hoshino M. Neuronal Migration and AUTS2 Syndrome. Brain Sci 2017; **7**:e54.
60. Hazen MM, Woodward AL, Hofmann I et al. Mutations of the hemophagocytic lymphohistiocytosis-associated gene UNC13D in a patient with systemic juvenile idiopathic arthritis. Arthritis Rheum 2008; **58**:567–570.
61. Procter M, Wolf B and Mao R. Forty-eight novel mutations causing biotinidase deficiency. Mol Genet Metab 2016; **117**:369–372.
62. Munier S, Delcroix-Genete D, Carthagena L et al. Characterization of two candidate genes, NCoA3 and IRF8, potentially involved in the control of HIV-1 latency. Retrovirology 2005; **2**:73.
63. Fan ZY, Song YL. Chinese Primate Status and Primate Captive Breeding for Biomedical Research in China. In: Institute for Laboratory Animal Research, National Research Council. International Perspectives: The Future of Nonhuman Primate Resources. Washington DC: National Academy Press. 2003.
64. Hao Xin. Monkey Research in China: Developing a Natural Resource. Cell 2007; **129**: 1033–

1036.

65. Zhou Q. Balancing the welfare: the use of non-human primates in research. Trends Genet 2014; **30**: 476–478.

66. Gradnigo JS, Majumdar A, Norgren RB Jr, Moriyama EN. Advantages of an Improved Rhesus Macaque Genome for Evolutionary Analyses. PLoS ONE 2016; **11**: e0167376.

67. Li H, Durbin R. Fast and accurate short read alignment with Burrows-Wheeler transform. Bioinformatics 2009; **25**:1754–1760.

68. Li H, Handsaker B, Wysoker A et al. The sequence alignment/map format and SAMtools. Bioinformatics 2009; **25**:2078–2079.

69. Mckenna A, Hanna M, Banks E, et al. The Genome Analysis Toolkit: a MapReduce framework for analyzing next-generation DNA sequencing data. Genome Res 2010; **20(9)**:1297–303.

70. Wang K, Li M, Hakonarson H. ANNOVAR: functional annotation of genetic variants from high-throughput sequencing data. Nucleic Acids Res 2010; **38**: e164.

71. Patterson N, Price AL, Reich D. Population structure and eigenanalysis. PLoS Genet 2006; **2**:e190.

72. Barrett JC, Fry B, Maller J et al. Haploview: analysis and visualization of LD and haplotype maps. Bioinformatics 2005; **21**:263–265.

73. Ségurel L, Wyman M J, Przeworski M. Determinants of mutation rate variation in the human germline. Annu Rev Genomics Hum Genet 2014; 15: 47–70.

74. Weir BS, Cockerham CC. Estimating *F*-statistics for the analysis of population structure. Evolution 1984; **38**:1358–1370.

75. Danecek P, Auton A, Abecasis G et al. The variant call format and VCFtools. Bioinformatics 2011; **27**:2156–2158.

76. Huang da W, Sherman BT, Lempicki RA. Systematic and integrative analysis of large gene lists using DAVID Bioinformatics Resources. Nat Protoc 2009; **4**:44–57.

Tables

Table1. Genetic diversity (θ) and effective population size (N_e) in Chinese rhesus macaques based on segregating sites (S) and nucleotide diversity (π).

		Sample size (n)	S		π	
			θ_W	N_e	θ	N_e
Chinese rhesus macaques (all samples)		81	0.00341	85,250	0.00228	57,000
Subspecies	*M. m. littoralis*	29	0.00292	73,000	0.00221	55,250
	M. m. tcheliensis	5	0.00188	47,000	0.00204	51,000
	M. m. brevicaudus	5	0.00179	44,750	0.00185	46,250
	M. m. lasiotis	32	0.00287	71,750	0.00224	56,000
	M. m. mulatta	10	0.00275	68,750	0.00220	55,000

Figure Legends

Figure 1. Phylogeny and population genetic structure of 81 wild Chinese RMs. (a) Geographic distribution of RMs in China (gray shadow) and the 17 sampling sites along with their subspecies assignment. (b) Neighbor-joining (NJ) tree and clustering solution inferred using STRUCTURE and displaying five populations (inferred with Evanno's ΔK method; Supplementary Fig. 5). (c) Principal component analysis plots depicting the first two components (variance explained by PC1 = 7.24% and PC2 = 5.69%).

Figure 2. Demographic history and differentiation scenarios of Chinese RMs. (a) Historical changes in effective population size reconstructed using the pairwise sequential Markovian coalescent (PSMC) applied on individual whole genomes for each of the five subspecies. The generation length (g) and the neutral mutation rate per generation (μ) were assumed to be 11 years and 1.08×10^{-8}, respectively. The Xixiabangma Glaciation (XG, 1,200-800 kya), Penultimate Glaciation (PG, 200-130 kya) and Last Glaciation (LG, 70-10 kya) are shaded in gray. (b) Demographic history inferred by *fastsimcoal2*. The width of the gray bars and numbers on them indicate the estimated effective population size. The arrows indicate migration patterns with the numbers above arrows indicating the average number of migrants per generation between different subspecies. Numbers at the right show the divergence times between subspecies. (c) Biogeographic scenario for RMs. Chinese RMs separates from Indian RMs ~ 162 kya [13], followed by further migration into China by the different RM subspecies indicated with arrows colored following the color key in Fig. 1a.

Figure 3. Genomic regions with selection sweep signals in RM. (a) Distribution of $\log_2$ (θ_π *M. m. lasiotis*/θ_π *M. m. tcheliensis*) and Z (F_{ST}) of 50-kb windows with 25-kb steps. Blue dots located in the selected regions requirement (corresponding to Z test P<0.05, where Z (F_{ST}) >= 1.848 and θ_π log-ratio >= 1.203) represent selected windows for *M. m. tcheliensis*. (b) Morphological comparison between *M. m. tcheliensis* and *M. m. lasiotis*. M and F represent males and females. (c) Example of genes with selection sweep signals. *Ext2, Rpgrip1l, Fbp2* and *Fbp1*in *M. m. tcheliensis* and *Axin1*, *Aggf1* and *Hspa4* in *M. m. brevicaudus*. F_{ST} and θ_π log-ratio between the two subspecies are represented in red and blue, respectively. All values in figure 3c are plotted using 50-kb windows with half steps. Genome annotations are show at the bottom (black bar, coding sequences (CDS); purple bar, genes). (d) SNP genotypes in putative selective sweeps

containing *Ext2, Rpgrip1l, Fbp2, Fbp1*, *Axin1*, *Aggf1* and *Hspa4*. (e) Non-synonymous variants in gene Ext2, *Rpgrip1l* and *Hspa4.*

Figure 4. Population study of putative pathogenic SNPs in Chinese RM subspecies. (a) The site and frequency of pathogenic SNPs located in *Unc13d* and *Btd* genes. (b) Scheme of the *Ncoa3* gene in RM. The positions of nonsynonymous polymorphisms (black) and three amino-acid deletions (in red) are marked. (c) Private and shared pathogenic SNPs in Chinese RM subspecies (blue: *M. m. tcheliensis;* orange: *M. m. brevicaudus;* red: *M. m. littoralis;* green: *M. m. mulatta;* purple: *M. m. lasiotis*). The sizes of the areas are not proportional to the magnitude of the numbers. (d) NJ tree including the 81 Chinese RMs derived from this study, the 26 captive Chinese RMs from Zhong et al. [7] are indicated by blue dot.

Figure

Click here to download Figure Figure_1.pdf

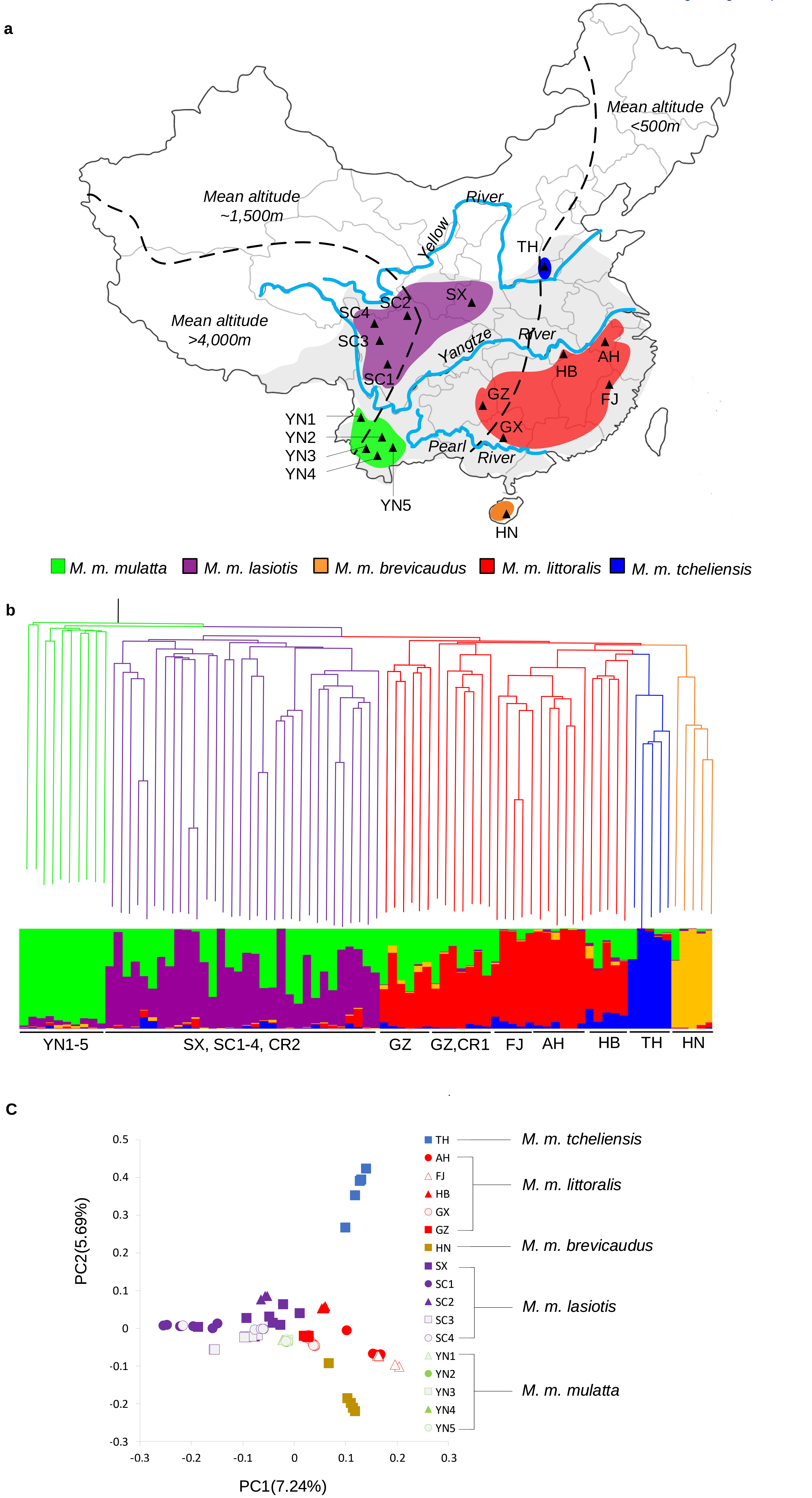

Figure

Click here to download Figure Figure_2.pdf

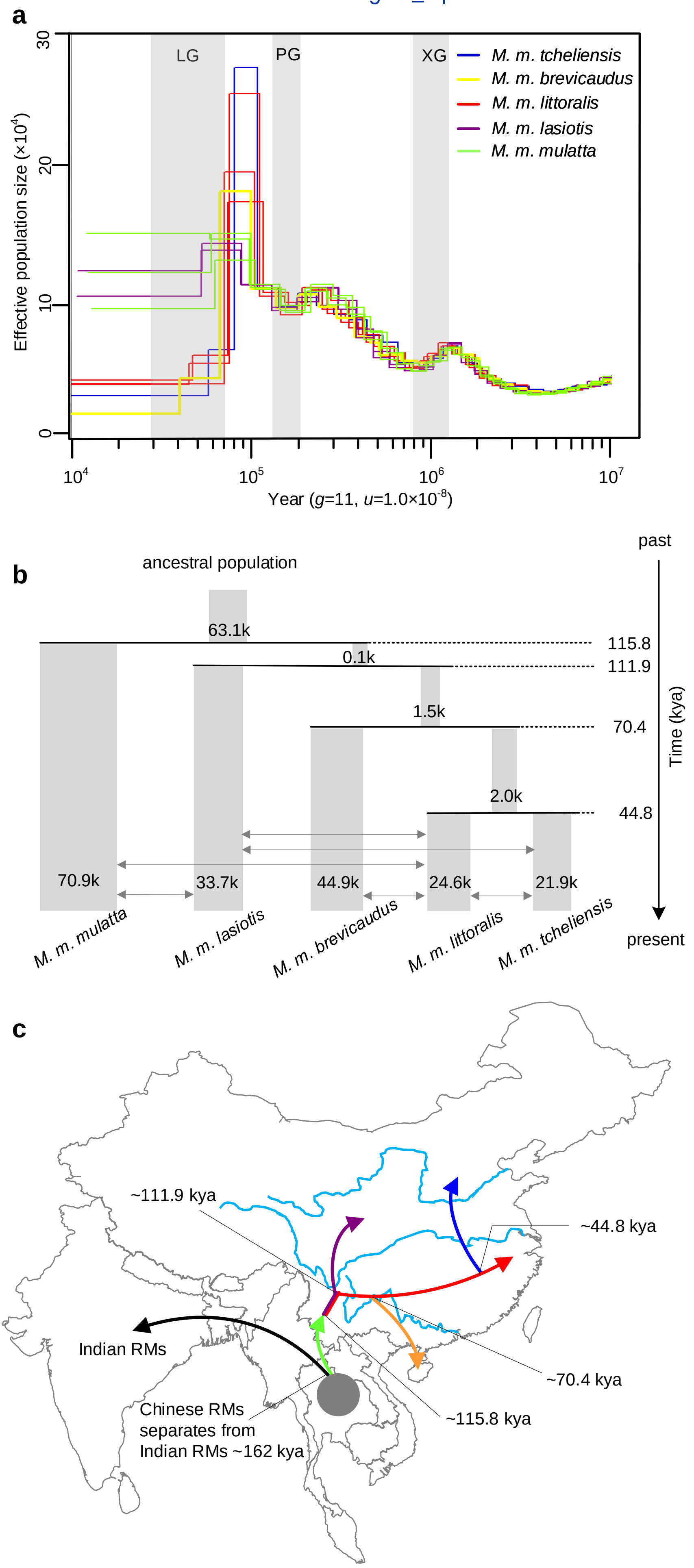

Click here to download Figure Figure_3.pdf

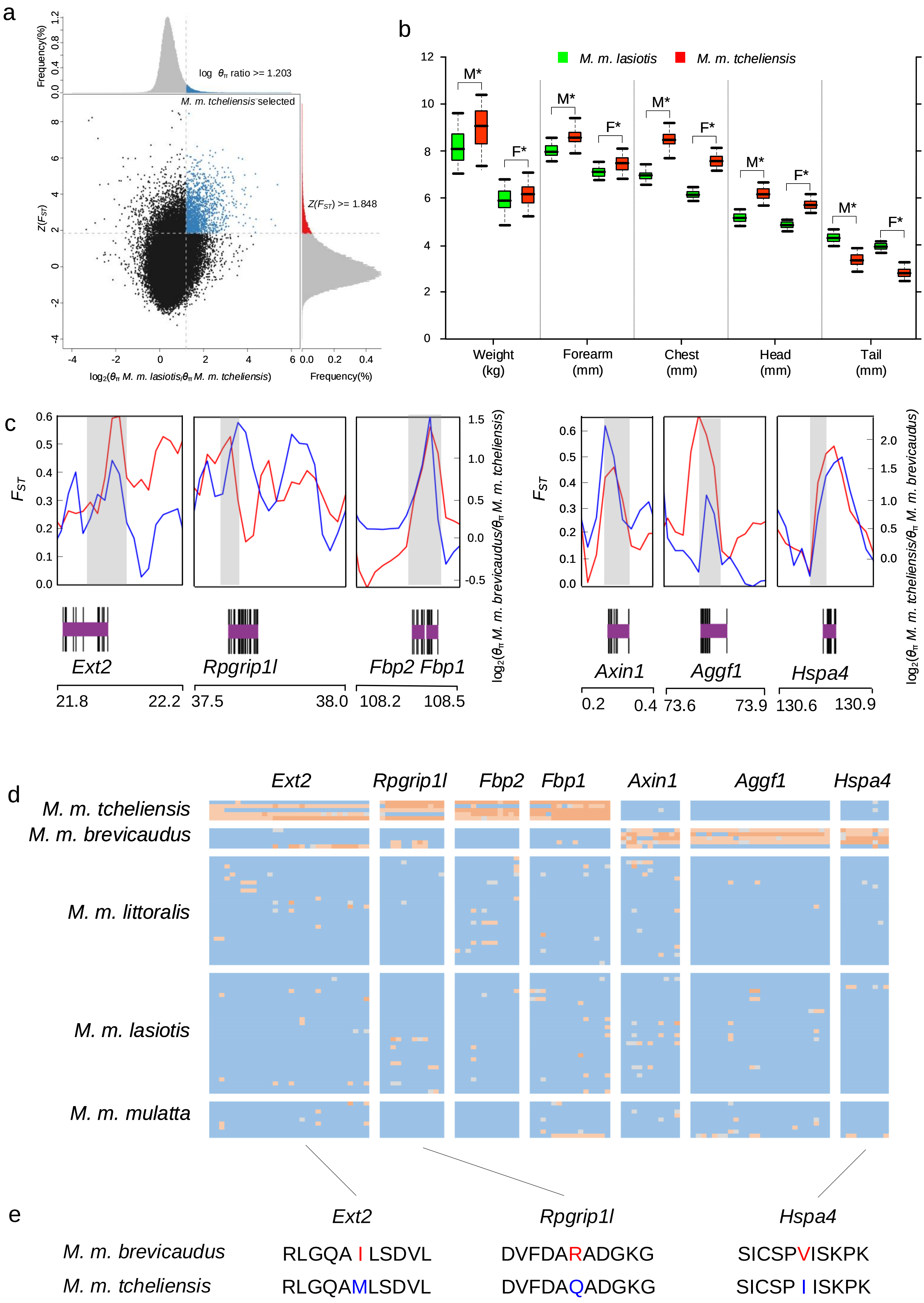

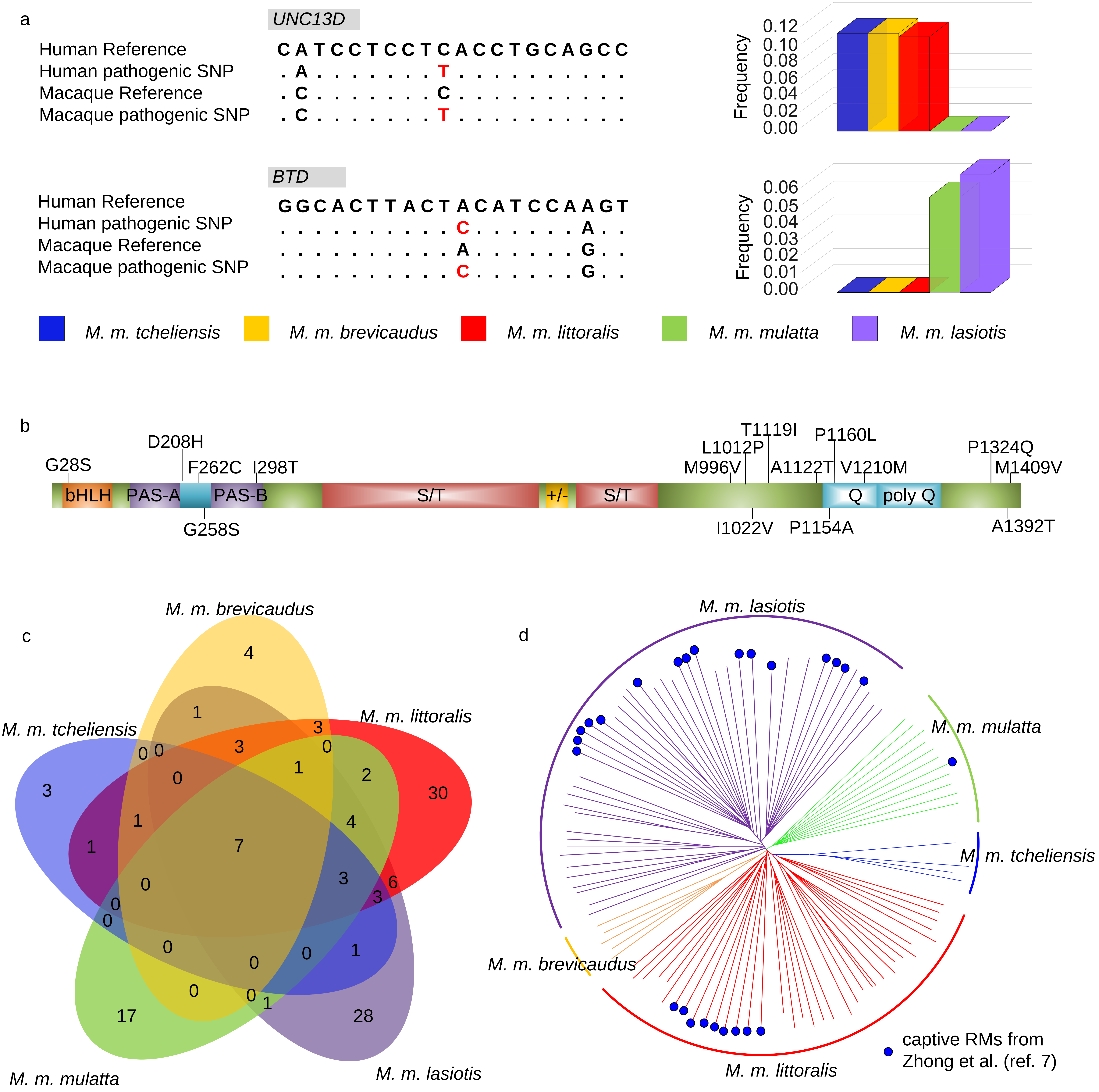
a
UNC13D
Human Reference
Human pathogenic SNP
Macaque Reference
Macaque pathogenic SNP
C A T C C T C C T C A C C T G C A G C C
. A . . . . T . . . .
. C . . . . C . . . .
. C . . . . T . . . .
Frequency
0.12 0.10 0.08 0.06 0.04 0.02 0.00
BTD
Human Reference
Human pathogenic SNP
Macaque Reference
Macaque pathogenic SNP
G G C A C T T A C T A C A T C C A A G T
. . . . C . . . . . . A . .
. . . . A . . . . . . G . .
. . . . C . . . . . . G . .
Frequency
0.06 0.05 0.04 0.03 0.02 0.01 0.00
M. m. tcheliensis
M. m. brevicaudus
M. m. littoralis
M. m. mulatta
M. m. lasiotis
b
G28S D208H F262C I298T M996V L1012P T1119I A1122T P1160L V1210M P1324Q M1409V
bHLH PAS-A PAS-B S/T +/- S/T Q poly Q
G258S I1022V P1154A A1392T
c
M. m. brevicaudus
M. m. littoralis
M. m. tcheliensis
M. m. mulatta
M. m. lasiotis
4 1 3 0 3 0 0 0 1 2 3 30 1 1 7 4 0 3 6 3 0 0 0 0 0 1 0 0 1 17 28
d
M. m. lasiotis
M. m. mulatta
M. m. tcheliensis
M. m. brevicaudus
M. m. littoralis
captive RMs from Zhong et al. (ref. 7)

Click here to access/download
Supplementary Material
renamed_d4adc.docx

Click here to access/download
Supplementary Material
Supplemental Data 1.xlsx

Click here to access/download
Supplementary Material
Supplemental Data 2.xlsx
