# Supplementary material for: Population genomics of wild Chinese rhesus macaques reveals a dynamic demographic history and local adaptation, with implications for biomedical research
Source: Gigascience. 2018 Aug 27;7(9):giy106. doi: 10.1093/gigascience/giy106 (PMC6143732; doi:10.1093/gigascience/giy106)
Supplement: GIGA-D-17-00291_Revision_2.pdf [file giy106_giga-d-17-00291_revision_2.pdf]

GigaScience

Population genomics of wild Chinese rhesus macaques reveals a dynamic demographic history and local adaptation, with implications for biomedical research

--Manuscript Draft--

**Manuscript Number:**	GIGA-D-17-00291R2
**Full Title:**	Population genomics of wild Chinese rhesus macaques reveals a dynamic demographic history and local adaptation, with implications for biomedical research
**Article Type:**	Research
**Funding Information:**	Key Project of National Natural Science Foundation of China (31530068) — Dr Ming Li<br>Strategic Priority Research Program of the Chinese Academy of Sciences (XDA19050202) — Dr Ming Li<br>Strategic Priority Research Program of the Chinese Academy of Sciences (XDB31000000) — Dr Ming Li<br>National Key R&D Program of China (2016YFC0503200) — Dr Ming Li<br>Creative Research Group Project of NSFC; and Science & Technology Department of Sichuan Province (2018JZ0008) — Dr Ming Li<br>Natural Science Foundation of China (31471989) — Dr Zhijin Liu
**Abstract:**	Background: The rhesus macaque (RM, Macaca mulatta) is the most important nonhuman primate model in biomedical research. We present the first genomic survey of wild RMs, sequencing 81 geo-referenced individuals of five subspecies from 17 locations in China, a large fraction of the species' natural distribution.<br><br>Results: Populations were structured into five genetic lineages on the mainland and Hainan Island, recapitulating current subspecies designations. These subspecies are estimated to have diverged 125.8 to 51.3 thousand years ago, but feature recent gene flow. Consistent with the expectation of a larger body size in colder climates and smaller body size in warmer climates (Bergman's rule), the northernmost RM lineage (M. m. tcheliensis), possessing the largest body size of all Chinese RMs, and the southernmost lineage (M. m. brevicaudus), with the smallest body size of all Chinese RMs, feature positively selected genes responsible for skeletal development. Further, two candidate selected genes (Fbp1, Fbp2) found in M. m. tcheliensis are involved in gluconeogenesis, potentially maintaining stable blood glucose levels during starvation when food resources are scarce in winter. The tropical subspecies M. m. brevicaudus showed positively selected genes related to cardiovascular function and response to temperature stimuli, potentially involved in tropical adaptation. We found 118 SNPs matching human disease-causing variants with 82 being subspecies-specific.<br><br>Conclusions: These data provide a resource for selection of RMs in biomedical experiments. The demographic history of Chinese RMs, and their history of local adaption offers new insights into their evolution and provides valuable baseline information for biomedical investigation.
**Corresponding Author:**	Ming Li<br><br>CHINA
**Corresponding Author Secondary Information:**	
**Corresponding Author's Institution:**	
**Corresponding Author's Secondary Institution:**	

Powered by Editorial Manager® and ProduXion Manager® from Aries Systems Corporation

**First Author:**	Zhijin Liu
**First Author Secondary Information:**	
**Order of Authors:**	Zhijin Liu
	Xinxin Tan
	Pablo Orozco-terWengel
	Xuming Zhou
	Liye Zhang
	Shilin Tian
	Zhongze Yan
	Huailiang Xu
	Baoping Ren
	Peng Zhang
	Zuofu Xiang
	Binghua Sun
	Christian Roos
	Michael W. Bruford
	Ming Li
**Order of Authors Secondary Information:**	
**Response to Reviewers:**	Dear editor,<br><br>We thank you for your encouragement and help to our manuscript "Population genomics of wild Chinese rhesus macaques reveals a dynamic demographic history and local adaptation, with implications for biomedical research".<br><br>We have shorted the abstract and structured it in sections ("Background – Results - Conclusions").<br><br>We have added a citation to our upcoming GigaDB dataset without the DOI at the end of the reference list and cited this in the 'Data Access'.<br><br>The point-to-point responses to the reviewers are listed below.<br><br><br>Reviewer #1:<br>All my major concerns have been satisfactorily addressed. However, I see several typos or other minor problems with the text.<br><br>Comment_1-1:<br>line 162: should be "from" instead of "form"<br>line 230: I think the authors mean "upregulated" not "unregulated"<br>line 285: should be "have" not "has"<br>I think there are other similar issues.<br>Response_1-1:<br>These issues have been amended accordingly. (line 158, line 226, line 283)<br><br><br>Reviewer #2:<br><br>Comment_2-1:<br>The authors have revised their manuscript based on the detailed reviewer reports and have substantially improved it in the process. Most of my previous methodological concerns have been addressed. However, there still seems to be some confusion regarding my comments about proper calculation of genetic diversity (comment 2-3

and 2-4). The authors write in their rebuttal that they have used the GATK Best Practices workflow with multi-sample genotyping, thereby solving the problem of uncallable sites by accumulating evidence across samples. This is only partly mitigating the problem here. If a site is not callable across all samples due to poor mappability or a 'N' in the reference sequence, a genetic variant cannot be detected at this position. Thus, genetic diversity will be underestimated when assuming that variant sites can occur along the entire genome. To obtain an accurate estimate of genetic diversity, it is therefore crucial to consider the exact number of sites that are callable, i.e. where it is possible to detect a potential variant. This can be achieved for example with GATK's 'CallableLoci' module. Alternatively, the '--includeNonVariantSites' flag of GATK's 'GenotypeGVCFs' can be used to emit both confident variant and non-variant sites. To be considered in the calculation of genetic diversity, a site should be callable (i.e. have a valid variant or non-variant genotype) in a certain proportion (e.g. 80%) of individuals. This initial site filter has to be independent of the variant state of a site, i.e. also variant sites in the SNP data set have to be filtered out if they don't fulfill the callability criteria.

Response_2-1:

We followed this helpful suggestion and redone the 'GenotypeGVCFs' in GATK with the '--includeNonVariantSites' flag to get both the variant and non-variant sites. Besides the basic hard filter by 'VariantFiltration' in GATK, we also filtered out the variants with a 'N' in the reference sequence or the sites including more than 20% missing genotypes. For the non-variant sites, we did the same filter and retain only the callable non-variant sites. The genetic diversity and heterozygosity have been re-estimated based on all the callable sites. (lines 101-102, lines 109-112, lines 379-390 and Table 1)

Comment_2-2:

Lines 76-79: These sentences are unclear. If to date only 9 captive Chinese RMs have been sequenced, how could Zhong et al. assess genetic diversity in 26 Chinese individuals? I guess the authors mean "Until recently, …" rather than "To date, …" at the beginning of the first sentence.

Response_2-2:

This issue has been amended accordingly. (lines 74-77)

Comment_2-3:

Line 102: This sentence is confusing. The authors write that they identified ~58 mio SNPs in the 81 Chinese RMs. From their explanations, I understood that this is the total number of variant sites, i.e. including fixed differences to the reference genome? The reference genome is of Indian origin, so it's incorrect to write that these are SNPs in Chinese RMs.

Response_2-3:

This issue has been amended accordingly. We have filtered out the fixed differences to the reference genome. (lines 386-388)

Comment_2-4:

Line 103: Was Watterson's theta correctly estimated considering only sites actually segregating within the Chinese RMs?

Response_2-4:

The θW and $\theta \pi$ have been re-estimated only based on segregating variations within Chinese RMs. (lines 101-102 and Table 1)

Comment_2-5:

Line 104: "and the nucleotide diversity measured by segregating sites (Watterson's θ, θW) and mean pairwise differences ($\theta \pi$) is …"

Response_2-5:

This issue has been amended accordingly. (lines 101-102)

Comment_2-6:

Lines 106-110: It doesn't make sense to use variant sites relative to a reference genome for the analysis of shared and private SNPs. These numbers reflect a mixture of segregating variation and fixed differences to the reference genome. Please redo these analyses by only considering actual segregating variation within the compared entities.

Response_2-6:

	We have filtered out the fixed difference to the reference genome. All these analyses have redone based on actual segregating variations within Chinese RMs. (lines 104-108, lines 387-388)<br><br>Comment_2-7:<br>Line 139: "based on θW and θπ are …"<br>Response_2-7:<br>This issue has been amended accordingly. (line 137)<br><br>Comment_2-8:<br>Lines 170-177: Round estimates and provide confidence intervals.<br>Response_2-8:<br>This issue has been amended accordingly. (lines 166-173)<br><br>Comment_2-9:<br>Lines 180-181: Tone down this statement, since you haven't explicitly compared models with and without gene flow. Something along the lines of: "Our results indicate that low levels of gene flow occurred between all five extant lineages of Chinese RMs."<br>Response_2-9:<br>This issue has been amended accordingly. Substantial gene flows have been detected between different subspecies. Please see the response to the comment_2-20 and Supplementary Table 5. (lines 177-178)<br><br>Comment_2-10:<br>Line 193: "led to further differentiation by limiting gene flow among them."<br>Response_2-10:<br>This issue has been amended accordingly. (line 190)<br><br>Comment_2-11:<br>Lines 208-211: This sentence seems to conflict with the sentence on lines 200-204.<br>Response_2-11:<br>For M. m. tcheliensis, which occurs in the northernmost range of the RMs under cold conditions, we first estimated FST and θπ between it and each of the other four subspecies. Then we got four lists of candidate genes in M. m. tcheliensis. The final positive selection genes are the intersection of these four lists. Similar, in the case of M. m. brevicaudus, we used the same method. The details of this process are shown in Supplementary Fig. 7. We chose this method to get the final positive selection genes, instead of directly comparing M. m. tcheliensis and M. m. brevicaudus, for the purpose of reducing the false positives of the results and obtaining more accurate selective gene lists. (lines 197-201, lines 205-208 and Supplementary Fig. 8)<br><br>Comment_2-12:<br>Lines 223-226: See previous comment 2-16.<br>Response_2-12:<br>This issue has been amended accordingly. (lines 220-223)<br><br>Comment_2-13:<br>Line 230: "upregulated" instead of "unregulated"?<br>Response_2-13:<br>This issue has been amended accordingly. (line 226)<br><br>Comment_2-14:<br>Lines 240-241: Having long forearms doesn't really fit the expectation, as long extremities would increase the surface to volume ration. I realize that forearm length is probably strongly correlated with body size, but this is confusing for the reader. Maybe just omit the forearm length.<br>Response_2-14:<br>This issue has been amended accordingly. We have omitted the forearm length in the revised manuscript. Many thanks for this helpful suggestion. (lines 237-238)<br><br>Comment_2-15:<br>Line 385-387: Provide details about the filter settings. The current description of the variant hard filtering approach doesn't allow to reproduce the data set used for the downstream analyses.

	Response_2-15:<br>This issue has been amended accordingly. After variant calling, we first applied the "SelectVariants" to exclude the Indel and split the variant and non-variant sites. Then we applied the hard filter command 'VariantFiltration' to exclude potential false-positive variant calls with the following criteria: "–filterExpression 'QD < 5.0 || FS > 60.0 || MQ < 40.0 || ReadPosRankSum< −8.0 || MQRankSum < -12.5'" and "--genotypeFilterExpression 'DP < 4.0'". Additionally, the sites are filtered if there is a 'N' is in the reference sequence; if the site is fixed difference to the reference genome or if the site including more than 20% missing genotypes. (lines 383-388)<br><br>Comment_2-16:<br>Line 410-411: Provide details of how the consensus sequences have been generated.<br>Response_2-16:<br>This issue has been amended accordingly. We called the consensus sequences using Samtools mpileup [68] by applying: "samtools mpileup -q 1 -C 50 -S -D -m 2 -F 0.002 -u -f *.fa(genome) *.bam | bcftools view -c - | vcfutils.pl vcf2fq -d 10 -D 100 -Q 20 - > *.psmc.fq" and "fq2psmcfa -q10 -s 100 *.psmc.fq >*.psmc.fa". To ensure the quality of consensus sequences, we used data of ten individuals with an average coverage >20× (22.20-34.32×). (lines 411-415)<br><br>Comment_2-17:<br>Line 418: Provide more details of how the SNP data has been converted to joint site frequency spectra. How was the number of non-variant sites assessed accurately (see comment above)?<br>Response_2-17:<br>VCF file containing callable variant sites was used converted to fastsimcoal style folded SFS. To mitigate the effect of linkage disequilibrium, we filtered out the SNPs located within 10 kb from genes and then we took one SNPs every 10kb randomly. The multidimensional folded SFS for all the five subspecies is generated by easySFS (https://github.com/isaacovercast/easySFS#easysfs). The non-variant sites were not used to convert the SFS. (lines 426-427)<br><br>Comment_2-18:<br>Lines 422-423: Not sure what this is supposed to mean. Have you simulated data sets under the inferred model and compared distributions of simulated summary statistics to the observed values? However, Supplementary Table 5 doesn't show distributions of summary statistics, rather estimates of model parameters. Provide details of how confidence intervals have been calculated and show how good the model fits the observed data.<br>Response_2-18:<br>Lines 422-423 is a typo-error and has been removed. We got confidence intervals after parameter estimation using parametric bootstraps. We chose the replicate with the highest estimated maximum likelihood to generate parametric bootstraps. One hundred multidimensional SFS files were generated for this set of parameters and then estimated parameters from these pseudo-observed data sets using the same tpl and est files as those used to get the parameters with highest likelihood. We used the option '-initvalues file.pv' to reduce the number of runs necessary to estimate parameters when estimating confidence intervals by bootstrap. The 'file.pv' containing initial parameter values for parameter estimation is automatically generated after parameter estimation by fsc26. The observed data and the confidence intervals from 100 parametric bootstraps were showed in Supplementary Fig. 7 and Supplementary Table 5. (lines 427-437)<br><br>Comment_2-19:<br>Line 475: I haven't been able to find any notes in the Supplementary Information.<br>Response_2-19:<br>This issue has been amended accordingly. According to the format of GigaScience, the supplementary files does not include any notes. This confusion on line 475 is a typo and we have modified it (line 491).<br><br>Comment_2-20:<br>Supplementary Table 5: Are the gene flow estimates really representing the number of migrants (i.e. Nem)? This would be completely negligible gene flow. Or are these

	numbers rather migration rates (i.e. m), which would imply quite substantial gene flow.<br>Response_2-20:<br>It is a typo-error. The gene flow estimated really represent the migration rate between subspecies, which imply quite substantial gene flow (Supplementary Table 5).
**Additional Information:**	
**Question**	**Response**
Are you submitting this manuscript to a special series or article collection?	No
**Experimental design and statistics**<br><br>Full details of the experimental design and statistical methods used should be given in the Methods section, as detailed in our Minimum Standards Reporting Checklist. Information essential to interpreting the data presented should be made available in the figure legends.<br><br>Have you included all the information requested in your manuscript?	Yes
**Resources**<br><br>A description of all resources used, including antibodies, cell lines, animals and software tools, with enough information to allow them to be uniquely identified, should be included in the Methods section. Authors are strongly encouraged to cite Research Resource Identifiers (RRIDs) for antibodies, model organisms and tools, where possible.<br><br>Have you included the information requested as detailed in our Minimum Standards Reporting Checklist?	Yes
**Availability of data and materials**<br><br>All datasets and code on which the conclusions of the paper rely must be either included in your submission or deposited in publicly available repositories (where available and ethically appropriate), referencing such data using	Yes

a unique identifier in the references and in the “Availability of Data and Materials” section of your manuscript.<br><br>Have you have met the above requirement as detailed in our Minimum Standards Reporting Checklist?	

Powered by Editorial Manager® and ProduXion Manager® from Aries Systems Corporation

Manuscript

Click here to access/download;Manuscript;rhesus_macaques_11_8_18.doc

Click here to view linked References

Title: Population genomics of wild Chinese rhesus macaques reveals a dynamic demographic history and local adaptation, with implications for biomedical research

Running Title: Population genomics of wild rhesus macaques

Zhijin Liu[1, †], Xinxin Tan[1, 2, †], Pablo Orozco-terWengel[3, †], Xuming Zhou[1, 4], Liye Zhang[1, 2], Shilin Tian[5], Zhongze Yan[1, 6], Huailiang Xu[7], Baoping Ren[1], Peng Zhang[8], Zuofu Xiang[9], Binghua Sun[10], Christian Roos[11], Michael W. Bruford[3, *], Ming Li[1, 12 *]

[1] CAS Key Laboratory of Animal Ecology and Conservation Biology, Institute of Zoology, Beijing, China.

[2] University of Chinese Academy of Sciences, Beijing 100039, China.

[3] School of Biosciences, Cardiff University, Sir Martin Evans Building, Museum Avenue, Cardiff CF10 3AX, United Kingdom.

[4] Division of Genetics, Department of Medicine, Brigham and Women's Hospital, Harvard Medical School, Boston, MA 02115, USA.

[5] Novogene Bioinformatics Institute, Beijing 100083, China.

[6] Institute of Physical Science and Information Technology, Anhui University, Hefei, 230601, China.

[7] College of Life Science, Sichuan Agricultural University, Ya'an 625014, China.

[8] School of Sociology and Anthropology, Sun Yat-sen University, Guang Zhou, China.

[9] College of Life Science and Technology, Central South University of Forestry and Technology, Changsha 410004, Hunan, China.

[10] School of Life Sciences, Anhui University, Hefei, 230601, China.

[11] Gene Bank of Primates and Primate Genetics Laboratory, German Primate Center, Leibniz Institute for Primate Research, Kellnerweg 4, 37077 Göttingen, Germany.

[12] Center for Excellence in Animal Evolution and Genetics, Chinese Academy of Sciences, Kunming, 650223, China.

[†] Contributed equally

***** Correspondence: Ming Li, lim@ioz.ac.cn; Michael W. Bruford, BrufordMW@cardiff.ac.uk

Abstract

Background: The rhesus macaque (RM, *Macaca mulatta*) is the most important nonhuman primate model in biomedical research. We present the first genomic survey of wild RMs, sequencing 81 geo-referenced individuals of five subspecies from 17 locations in China, a large fraction of the species' natural distribution.

Results: Populations were structured into five genetic lineages on the mainland and Hainan Island, recapitulating current subspecies designations. These subspecies are estimated to have diverged 125.8 to 51.3 thousand years ago, but feature recent gene flow. Consistent with the expectation of a larger body size in colder climates and smaller body size in warmer climates (Bergman's rule), the northernmost RM lineage (*M. m. tcheliensis*), possessing the largest body size of all Chinese RMs, and the southernmost lineage (*M. m. brevicaudus*), with the smallest body size of all Chinese RMs, feature positively selected genes responsible for skeletal development. Further, two candidate selected genes (*Fbp1*, *Fbp2*) found in *M. m. tcheliensis* are involved in gluconeogenesis, potentially maintaining stable blood glucose levels during starvation when food resources are scarce in winter. The tropical subspecies *M. m. brevicaudus* showed positively selected genes related to cardiovascular function and response to temperature stimuli, potentially involved in tropical adaptation. We found 118 SNPs matching human disease-causing variants with 82 being subspecies-specific.

Conclusions: These data provide a resource for selection of RMs in biomedical experiments. The demographic history of Chinese RMs, and their history of local adaption offers new insights into their evolution and provides valuable baseline information for biomedical investigation.

Keywords: *Macaca mulatta*, population genomics, adaptive selection, biomedical model

Introduction

Understanding how species evolve and adapt to their environments is an essential question in evolutionary biology. Rhesus macaques (RMs, *Macaca mulatta*) are, after humans, the world's most widely distributed primates [1-5], occupying a vast geographic distribution spanning from Afghanistan to the Chinese shore of the Pacific Ocean and south into Myanmar, Thailand, Laos and Vietnam [5]. As the most widely distributed nonhuman primate species, RMs occupy diverse ecological landscapes and habitats, making them an interesting model to address questions about how species evolve and adapt to local environmental variation, including characterizing the genomic architecture of adaptation to habitat, climate and other biotic and abiotic factors. Yet, despite much work on primate comparative genomics, very few population genomic studies have been carried out on wild RMs [6, 7]. Importantly, as RMs are widely used as a primate model in physiological, psychological and cognitive studies [8-10], knowledge about their genomic architecture could improve and refine biomedical research [10] as the genomic composition of experimental animals can have a considerable influence on the outcome of experiments [11, 12]. Therefore, information on the genomic diversity not only of captive, but also of wild RMs, that could become a genomic resource for future utilization in medical research, is essential.

In biomedical research, two main RM populations (Indian and Chinese) are recognized [6, 13]. They diverged from each other ~162 thousand years ago (kya) and are characterized by extensive differences in morphology, behavior, ecology, physiology, reproduction, and disease progression [6, 13-19]. In 1978 India banned all RM exports to breeding centers across the world, thus curtailing the availability of wild Indian RMs and subsequently increasing the demand for Chinese RMs in biomedical research, thereby making a detailed characterization of genetic variants from Chinese RMs crucial for biomedical usage of this species.

Until recently, the genomes of 133 captive RMs from eight colonies have been sequenced, however, 124 of them are of Indian-origin and only nine individuals were presumed to be of Chinese origin [6]. Besides, Zhong *et al.* [7] reported genomic variation in 26 Chinese captive RMs identifying ~46 million (M) single nucleotide polymorphisms (SNPs). Nevertheless, most of the RM genetic variation known to date is limited to captive populations which may contain composite genotypes due of admixture among animals of different and unclear origin [20]. Here

we present the first attempt to survey the geo-referenced genomic diversity in wild Chinese RM populations, which is the largest extant population of the species. The current effective population size of Chinese and Indian RM was estimated to be approximate 240,000 and 17,000 individuals, respectively, indicating that the Chinese RMs are likely to harbor substantially more genomic diversity compared to their Indian conspecifics [13]. Therefore, this population genomic survey of 81 RMs originating from 17 wild locations across China including phylogenetic and demographic analyses, as well as genome-wide selection scans, corresponds to the most comprehensive characterization of RM genetic diversity to date and aimed at characterizing the processes leading to the extant patterns of variability, as well as identifying the potential implications for the use of these populations in biomedical research.

Results and Discussion

Genetic diversity, phylogeny and population structure

Blood and tissue samples from 79 wild-born RMs, representing five subspecies [21, 22], were collected at 17 sites in China (*M. m. tcheliensis*: TH; *M. m. littoralis*: AH, FJ, HB, GX, GZ; *M. m. brevicaudus*: HN; *M. m. lasiotis*: SX, SC1, SC2, SC3, SC4; *M. m. mulatta*: YN1, YN2, YN3, YN4, YN5; Fig. 1a). Genome sequences of two additional Chinese RMs (CR1 and CR2) were retrieved from NCBI [9, 23, 24]. Re-sequencing was at a high average depth of 28.06±5.08× for ten individuals and a moderate average depth of 9.98±1.05× for the remainder (n=71), with an overall average genome coverage of 93.77% of the RM reference (Mmul_8.0.1, Supplementary Table 1). A total of 52,534,348 autosome SNPs were identified in these 81 wild Chinese RMs (Supplementary Table 2), and the nucleotide diversity measured by segregating sites (Watterson's θ, θ_W) and mean pairwise differences ($\theta\pi$) is 0.00375 and 0.00247, respectively (Table 1). The number of SNPs (all positions with differences to the genome reference) per individual ranged from 7.0 to 9.2 M (mean of 8.50 M; Supplementary Fig. 1 and Supplementary Table 3). Among all detected SNPs, 8,171,139 were shared among all subspecies and 22,768,395 were shared by at least two subspecies, with the remaining SNPs confined to a single subspecies (Supplementary Fig. 2a). For each subspecies, the subspecies-specific SNPs (ssSNPs) ranged from 702,099 to 7,736,924 and the non-synonymous ssSNPs varied from 3,056 to 25,960 (Supplementary Fig. 2a,

b). Among Chinese RM subspecies, *M. m. mulatta* had the highest heterozygosity ($2.29\times10^{-3}\pm3.24\times10^{-5}$), followed by *M. m. lasiotis* ($2.04\times10^{-3}\pm1.40\times10^{-4}$) and *M. m. littoralis* ($2.00\times10^{-3}\pm1.18\times10^{-4}$). The lowest heterozygosity rates were found in *M. m. brevicaudus* ($1.82\times10^{-3}\pm1.28\times10^{-4}$) and *M. m. tcheliensis* ($1.46\times10^{-3}\pm2.65\times10^{-4}$) (Supplementary Fig. 3).

We reconstructed a neighbor-joining (NJ) tree for Chinese RMs based on autosomal SNPs, using Indian RMs and *M. sylvanus* as outgroups (Fig. 1b and Supplementary Fig. 4). Individuals from *M. m. lasiotis*, *M. m. brevicaudus* and *M. m. tcheliensis* form monophyletic lineages respectively, while *M. m. mulatta* and *M. m. littoralis* are paraphyletic. Next, we performed a population structure analysis using STRUCTURE (version 2.3.4) [25], which estimates individual ancestry and admixture proportions assuming K ancestral populations. Plots of ΔK generated from STRUCTURE results indicated five genetic clusters present in the full data set (Fig. 1b and Supplementary Fig. 5). A principal component analysis (PCA) corroborated the division of Chinese RMs into five groups. The first eigenvector separated *M. m. mulatta* and *M. m. lasiotis* from *M. m. tcheliensis*, *M. m. littoralis* and *M. m. brevicaudus* (variance explained = 7.24%, Tracy-Widom $P = 4.78\times10^{-44}$), and the second eigenvector further separated *M. m. tcheliensis*, *M. m. littoralis* and *M. m. brevicaudus* (variance explained = 5.69%, Tracy-Widom $P = 4.21\times10^{-27}$) (Fig. 1c, Supplementary Table 4). The division of Chinese RMs into five geographic lineages supports the former taxonomic division of Chinese RMs into five subspecies [21, 22]. *M. m. mulatta* (YN1-5) and *M. m. lasiotis* (SC1-4, SX) form the pan-western populations of Chinese RMs, with both subspecies inhabiting the montane Tibetan Plateau regions with an altitude ≥1500 meters above sea level in western China and separated from each other by the Yangtze River. *M. m. littoralis* (AH, FJ, HB, GX, GZ), *M. m. tcheliensis* (TH) and *M. m. brevicaudus* (HN) occur in the eastern coastal lowland of China and form the pan-eastern population. *M. m. tcheliensis* from the Taihang Mountains area (TH) is the northernmost (34°54’–35°16’ N; 112°02’–112°52’ E), while *M. m. brevicaudus*, restricted to Hainan Island, is the most southern Chinese RM subspecies.

Demographic and phylogeographic history

The estimated effective population sizes, based on θ_W and $\theta\pi$ are approximately 93,750 and 61,750 for Chinese RMs (Table 1). In order to infer the ancient demographic history of Chinese

RMs, we applied a pairwise sequential Markovian coalescent (PSMC) [26] analysis using ten RM individuals with an average sequencing coverage depth higher than 20× (one individual of *M. m. tcheliensis* and one of *M. m. brevicaudus*, two of *M. m. lasiotis*, three of *M. m. littoralis* as well as three individuals of *M. m. mulatta*). The inferred PSMC trajectories were very similar for all analyzed individuals throughout most of the species' history reflecting the species' cohesiveness (Fig. 2a). The ancient demographic history of RMs is marked by population fluctuations following the glacial periods during the Pleistocene [27]. Approximately 1,200-800 kya all Chinese RMs experienced a population reduction at the time of the Xixiabangma Glaciation (XG), followed by an expansion during the Mid-Pleistocene inter-glaciation (800-200 kya). This expansion was then interrupted by the Penultimate Glaciation (PG, 200-130 kya) when suitable habitat might have been lost leading to a population decline [27]. PSMC analyses also suggested that, all the Chinese RMs had a population expansion during the last interglacial (around 100 kya) and a subsequent bottleneck during the Last Glaciation (LG, 70-10 kya) (Fig. 2a). Interestingly, the demographic inference by Xue et al. [6] derived from genomic data of a single Chinese RM (CH_37945) from AH (*M. m. littoralis*) qualitatively resembled the demographic trajectory of *M. m. littoralis* presented herein.

To further describe the divergence process among the five Chinese RM subspecies, we also employed the SVDquartes approach [28-31] that takes incomplete lineage sorting into account. The obtained phylogenetic tree suggests a "step-by-step" divergence of the five subspecies. Accordingly, the *M. m. mulatta* lineage diverged from that of the remaining Chinese RMs firstly and then the *M. m. lasiotis* diverged from the ancestral lineage of pan-eastern RMs (*M. m. tcheliensis*, *M. m. littoralis* and *M. m. brevicaudus*). Subsequently, *M. m. brevicaudus* diverged from the ancestor of *M. m. tcheliensis* and *M. m. littoralis*, the divergence of which occurred lastly (Supplementary Fig. 6). Under this "step-by-step" divergence scenario, we performed the joint site frequency spectrum (SFS) based approach implemented in *fastsimcoal2* [32] to model demographic fluctuations, respective divergence times and gene flow events among the five RM subspecies. Following the divergence between the ancestral lineages of Indian and Chinese RMs (~162 kya), the ancestor of *M. m. mulatta* diverged from the remaining Chinese RMs ~125.8 kya (95 % CI: 92.0-162.1 kya) (Fig. 2b) [6, 13]. Subsequently, *M. m. lasiotis* diverged from the ancestral lineage of pan-eastern RMs ~104.1 kya (95 % CI: 50.2-154.5 kya) near the end of the

last interglacial. The divergence time between *M. m. brevicaudus* and the ancestor of *M. m. tcheliensis* and *M. m. littoralis* was estimated at ~61.7 kya (95 % CI: 43.6-115.1 kya), while the divergence between the latter two occurred ~ 51.3 kya (95 % CI: 7.2-55.4 kya) during the last glacial maximum [33,34]. Interestingly, the coalescence analysis revealed a large ancestral population size of the Chinese RMs 125.8 kya (95 % CI: 92.0-162.1 kya) before and a subsequent population decline and the divergence among the five subspecies (Fig. 2b), which coincided with the population expansion during the last interglacial (around 100 kya) and the subsequent bottleneck of Chinese RMs during the Last Glaciation (LG, 70-10 kya) revealed by PSMC analyses. Our results indicate substantial gene flow occurred between all five extant lineages of Chinese RMs (Fig. 2b, Supplementary Table 5 and Supplementary Fig. 7).

A previous study of mitochondrial DNA identified two major haplogroups dividing Chinese RMs into a western and an eastern clade. Modern Chinese RMs were thought to have undergone a northward expansion while entering China via two possible routes: the first into the western mountains and the second following the eastern coast [35]. Our evolutionary model, however, suggests a “step-by-step” colonization process of RMs in China (Fig 2c). After the divergence from the Indian population (~162 kya) [6, 13], the ancestor of Chinese RMs colonized the Tibetan Plateau from southwestern China, and then experienced a range expansion north and eastwards. The pan-western population (*M. m. mulatta* and *M. m. lasiotis*) inhabited the western montane region in China, while the pan-eastern population (*M. m. tcheliensis, M. m. littoralis* and *M. m. brevicaudus*) entered the eastern coastal region. These five subspecies further diverged from each other during the bottleneck caused by the Last Glaciation. Additionally, barriers such as the Yellow, Yangtze and Pearl rivers and open sea (Fig. 1a) led to further differentiation by limiting gene flow among them. Water bodies and mountains could therefore be described as driving the formation of a habitat ‘lattice’ with the different subspecies of RMs occupying different grids in the lattice.

Signatures of selection and local adaptation

The wide distribution of Chinese RMs and their respective contrasting habitat types, as well as their wide use in biomedical studies, makes them an important case study for the analysis of signatures of local adaptation to divergent selective pressures [36-38]. We identified putative targets of selection by carrying out pair-wise comparisons between RM subspecies inhabiting the

most different environments to increase the chance of finding selection signatures, i.e., *M. m. tcheliensis* that occurs in the northernmost range of the species under cold conditions, and *M. m. brevicaudus* that inhabits the southernmost range of the species, a tropical island. For each analysis, we compared the five subspecies using the fixation index (F_{ST}) and genetic diversity (θ_{π}), calculated on 50kb long sliding windows (Fig. 3 and Supplementary Figs. 8-13). The top 5% of the windows with the largest F_{ST} and θ_{π} ratios ($\theta_{\pi}2$ /$\theta_{\pi}1$) in each pair-wise comparison were considered to be potentially under positive selection. For each subspecies, we identified the intersection of potential selective-sweep regions generated by all the pair-wise comparisons between a subspecies and each of the other subspecies (four pairwise comparisons in each case) (Supplementary Fig. 8). We used these consistent selective-sweep regions for further analyses, as they represent robust putative positively selected regions. The sizes of candidate selective-sweep regions ranged from 0.100 Mb to 11.075 Mb and the number of genes located in these regions, which are expected to represent targets of selection for each subspecies, varied from 6 to 176 in different subspecies (Supplementary Table 6).

M. m. tcheliensis from the Taihang (TH) Mountains area is the northernmost population of the species. The TH Mountains are characterized by a continental monsoon climate, and conditions for RMs are harsh during winter and early spring with extreme cold temperatures of –14°C [39]. Food resources are limited and consist mainly of barks, twigs, roots of crops and withered grass, thus, all sources are high in fiber, but low in energy and nutritional value [40, 41]. Therefore, *M. m. tcheliensis* suffers from starvation due to food shortage during winter and early spring. In starvation, blood glucose levels are maintained by gluconeogenesis through which glucose are converted from other molecules, such as amino acids and lactic acid [42]. For *M. m. tcheliensis*, the positive selection genes are enriched in the gene ontology (GO) term "fructose 1, 6-bisphosphate 1-phosphatase activity" with two genes (*Fbp1*, *Fbp2*, modified Fisher Exact *P*=1.90E-02; Fig. 3c, d; Supplementary Table 7). These two genes encode for fructose-1, 6-bisphosphatase 1 and fructose-1, 6-bisphosphatase isozyme 2 which catalyze the hydrolysis of fructose 1, 6-bisphosphate and play a rate-limiting role in gluconeogenesis. Furthermore, in starved zebrafish it was shown that the expression of *Fbp1* was significantly upregulated in brain and liver tissues [43]. The positive selection genes are also enriched in other terms and pathway related to gluconeogenesis, including KEGG pathway "Fructose and mannose metabolism"

(modified Fisher Exact *P*=4.35E-02) and GO terms "hexose biosynthetic process", "monosaccharide biosynthetic process" and "cellular carbohydrate biosynthetic process" (modified Fisher Exact *P*=3.36E-02, *P*=4.64E-02 and *P*=2.65E-02; Supplementary Table 7). Our findings suggest that the regulation of gluconeogenesis might be a mechanism of *M. m. tcheliensis* to adapt to food shortage in winter.

According to Bergman's rule, animals living in cold climates tend to have larger body sizes compared to their relatives in warm climates (i.e. they have a lower surface area to volume ratio), so they radiate less body heat per unit of mass [44]. Consistent with this expectation, among all RM subspecies, *M. m. tcheliensis* exhibits the largest body size and mass, and the largest head and chest circumference (Fig. 3b and Supplementary Table 8) [40, 45]. Among the consistent signatures of positive selection identified in *M. m. tcheliensis* (176 genes), we found signatures of selective sweeps in eight genes linked to limb morphogenesis or skeletal system development (Supplementary Table 6). Among these genes, *Fto* and *Rpgripl1* play an essential role in postnatal growth of mammals [46]. Mice lacking *Fto* completely display immediate postnatal growth retardation with shorter body length, lower body weight, and lower bone mineral density than control animals [47]. Furthermore, *Sox5* and *Sox6* (Fig. 3c, d) play an essential role in synovial joint morphogenesis via promoting both growth plate and articular chondrocyte differentiation [48]. Mutations in *Atp6v0a4* could cause developmental delay and delayed closure of the anterior fontanelle in human [49], while expression of *Ext2* enhances the bone formation in mice [50] These genes involved in the growth and development of the skeletal system and appendages are likely contributors to the larger body size of *M. m. tcheliensis*, and represent an undescribed adaptive pathway for primates living in colder climates.

In contrast, *M. m. brevicaudus* inhabits the tropical island of Hainan (HN) where it copes with a mean annual temperature of 24°C. *M. m. brevicaudus* has the smallest body size, the smallest body mass, and the shortest tail among RM subspecies [45]. As described above, they radiate more body heat per unit of mass (Bergman's rule) [44]. We found 127 putatively selected genes in *M. m. brevicaudus* (Supplementary Table 6), four of which were found to be enriched in GO term "Bone morphogenetic protein (BMP) signaling pathway" (modified Fisher Exact *P*=4.65E-02; Supplementary Table 9) and two genes were found to be enriched in GO term "I-SMAD binding (modified Fisher Exact *P*=4.65E-02; Supplementary Table 9)". BMP and

I-SMAD signaling pathways are involved in the development of bones and the skeleton [51, 52]. Mutations in *Axin1*, a gene of the I-SMAD pathway, cause kinked tails in mice [53]. In *M. m. brevicaudus*, we found two non-synonymous mutations in this gene (A674G, T656I) (Supplementary Fig. 14 and Supplementary Table 10, 11).

Additionally, putatively selected genes in *M. m. brevicaudus* (Fig. 3c, d, Supplementary Table 6) were also involved in GO terms related to cardiovascular system and blood circulation. For example, *Aggf1* related to GO term "blood vessel morphogenesis" and *Ctnna3* related to GO term "regulation of heart rate by cardiac conduction". The up-regulated *Aggf1* expression is capable of increasing blood flow in mouse hindlimb [54]. In addition, *Hspa4*, heat shock 70kDa protein 4, is directly involved in GO term "response to temperature stimulus". We thus hypothesize that the cardiovascular system of *M. m. brevicaudus* might play an important role in stabilizing body temperature, assisted by blood flow through different body parts requiring good fluidity and vascular permeability to transfer heat out of the body [55]. Testing these hypotheses needs further functional assays, however, these genes, together with the positively selected genes identified in *M. m. tcheliensis*, are known to be relevant to human physical function, and thus are likely of importance in the adaptation of Chinese RMs to different climate conditions.

Both coding and non-coding changes could contribute to local adaptations of organisms [56]. To further investigate the adaptive mechanism of *M. m. tcheliensis* and *M. m. brevicaudus* to the opposite climates (cold versus hot), we focused on SNPs in the gene regions of above described candidate genes. A total of 5817 SNPs were found with significant differences at the 5% level in the distributions of genotypes between these two subspecies, and 10 SNPs were non-synonymous variants (Supplementary table 10 and 11). In *M. m. tcheliensis*, non-synonymous mutations were found in the coding regions of *Atp6v0a4* (R667Q), *Ext2* (I363M), *Fto* (N10S) and *Rpgrip1l* (R1281Q) (Supplementary table 11 and Supplementary Fig. 14), implying that selection might have acted on protein sequence changes. No non-synonymous changes were detected in *Fbp1*, *Fbp2*, *Sox5* and *Sox6*. However, SNPs are located in the 1kb up/downstream, 5' and 3' UTR, and intronic regions of these genes (Supplementary table 10), indicating selection on non-coding regulatory variants. Correspondingly, non-synonymous mutations in *Aggf1* (H343Y), *Axin1* (A674G, T656I), *Hspa4* (I782V) and *Ctnna3* (V551I, T577M) were revealed for *M. m. brevicaudus* (Supplementary table 11 and Supplementary Fig. 14).

Besides the genes related to the adaptation to various climate conditions, we also found signatures of positive selection in genes related to the nervous system. In *M. m. tcheliensis* the 176 identified candidate genes are enriched in GO term “synapse” (modified Fisher Exact *P*=4.28E-02; Supplementary Table 7) with eight genes, and two of these gene, *Gabra2* and *Chrm2* are associated with alcohol dependence [57]. For *M. m. brevicaudus,* 18 putatively selected genes related to nervous system development were found. For example, *Dcc* is reported to be required for long-term potentiation and memory [58]. *Auts2*, one of the eight putatively selected genes in *M. m. lasiotis,* has been shown to regulate neuronal migration, and mutations in this gene cause mental dysfunction in human [59] (Supplementary Table 6). Our findings suggest that RM subspecies have experienced different adaptative processes in the nervous system and respective genomic differences should be taken into account when animals are selected for neurobiological research.

Disease–causing variants and implication for biomedical research

Given the large evolutionary similarity between macaques and humans, human diseases are better modeled in RMs than in many other animals. Thus, variants in RMs that match to orthologous human variants annotated as ‘pathogenic’ are of particular interest. We examined presumed homologous Chinese RM SNPs in the human genome and a total of 34,850,330 RM SNPs analyzed in this study were successfully identified in the human genome (hg19). Among these SNPs, 118 variants matched human variants with the accordant reference alleles and alternative alleles were annotated as ‘disease causing’ in HGMD or pathogenic in ClinVar. These 118 RM SNPs affect genes that cause specific human diseases including acromesomelic dysplasia maroteaux type, anonychia, atransferrinemia, blau syndrome, Carcinoma of colon, Charcot-Marie-Tooth disease, deafness, early infantile epileptic encephalopathy 7, glycogen storage disease and others (Supplementary Table 12). Among these 118 SNPs，only seven pathogenic SNPs are shared by all five subspecies, while 82 are subspecies-specific (Fig. 4c, Supplementary Table 12). For example, the SNP rs116229331 in the gene *Unc13d* (human Chr17: 73836585C>T), known to cause juvenile idiopathic arthritis in humans [60], has a RM homologue (RM Chr16: 69559126 C>T, Fig. 4a) that is present in *M. m. tcheliensis*, *M. m. brevicaudus* and *M. m. littoralis*, but absent in *M. m. lasiotis* and *M. m. mulatta*. Another

pathogenic variant (rs397514345, human Chr3: 15686724 A>C) in the *Btd* gene is involved in biotinidase deficiency [61]. Its homologous RM variant (RM Chr2: 172277927 A>C, Fig. 4a) is found only in *M. m. lasiotis* and *M. m. mulatta.* In addition, we also identified 16 non-synonymous SNPs in the *Noca3* gene, which encodes a protein that modulates the replication and transcriptional reactivation of HIV-1 during virus latency [62] (Fig. 4b). Ten of these 16 non-synonymous SNPs are private to one subspecies (Supplementary Table 13). The effects of these variants on HIV-1 replication and reactivation are unknown and need further investigation, but the high number of mutations suggests a complex response of the host to the virus.

Overall, these findings suggest that the genomic architecture of Chinese RMs used in biomedical research and their geographic origin could strongly influence the outcome of biomedical experiments and should be taken into account when using Chinese RMs in clinical and neurobiological research. Unfortunately, genome wide screening of RMs used in biomedical research is so far only rarely conducted and uncharacterized animals are most often used. Importantly, individuals from all five Chinese RM subspecies are used in biomedical research [63, 64]. Combined with our data, nine of the 26 captive Chinese RMs reported by Zhong *et al.* [7] were found to cluster with *M. m. littoralis*, 16 with *M. m. lasiotis* and one with *M. m. mulatta* (Fig. 4d). Thus, the data and results presented here provide the basis to trace the origin of captive RMs and to allow for the selection of appropriate animal models when testing for particular diseases, and are thus a significant contribution to the "3Rs" principle, which aims to reduce, refine, and replace experimental animals [65].

Conclusion

We present the first description of the evolutionary history and genomic variation of geo-referenced wild RMs throughout China, including scenarios on potential functions of this variation in adaptation to local environments. This genomic resource represents a valuable contribution to the understanding of the biology and evolution of a highly successful and important biomedical research species. In particular, it is important to note that due to the difference in evolutionary history of the subspecies identified here, it can be expected that animals originating from different regions may react differently to experimental tests, and thus their background needs to be assessed beforehand [10]. Our results highlight the importance that genome typing can play in biomedical research where animal origins are uncertain, and the resources generated here provide a baseline for genomic assessment of biomedical research populations, genetic resource conservation and for refined usage of RMs in future research.

Materials and Methods

Ethics statement

The methods were carried out in accordance with the approved guidelines of the Good Experimental Practices adopted by the Institute of Zoology, Chinese Academy of Sciences (CAS). All experimental procedures and animal collection were conducted under the supervision of the Committee for Animal Experiments of the Institute of Zoology, Chinese Academy of Sciences.

Sample Collection and Sequencing

Samples from 79 individuals with information about geographic origin were collected from 17 local wildlife rescue center, which covered most of the species' range in China. Muscle samples were collected from deceased individuals and the blood samples were taken during routine physical examinations. Total genomic DNA was extracted from blood or tissue samples using standard phenol/chloroform methods. For each individual, ~3 μg DNA was sheared into fragments of 500 bp with the Covaris system. DNA fragments were then processed and sequenced using the Illumina HiSeq 2000 and 2500 platform. Furthermore, published genomic data for two individuals were download form NCBI [9,23] and filtered using the same conditions. Raw reads were first filtered with the following criteria: (1) reads with unidentified nucleotides (N) exceeded 10% were discarded, (2) reads with the proportion of low quality base (phred quality <=5) larger than 50% were discarded. After the quality control, a total of 3,095.6 Gb of high quality sequences with 22.53 billion pair-end reads (100 or 125 bp) were generated.

Sequence Data Pre–processing and Variant Calling

High-quality sequence reads were mapped to the macaque reference genome, Mmul_8.0.1 [66], using the Burrows–Wheeler Aligner 0.7.10-r789 (BWA, RRID:SCR_010910) [67]. Sequence Alignment/Map (SAM) format files were imported to SAMtools v0.1.19 (SAMtools, RRID:SCR_002105) [68] for sorting and then imported to Picard version 1.118 (Picard, RRID:SCR_006525) (http://broadinstitute.github.io/picard/) for removing duplicated reads. To improve the quality of sites reported, we performed SNP calling following GATK's best practice, version 3.3–0 (GATK, RRID: SCR_001876) on autosomal sites only [69]. We get the GVCF file

for each individual using the “HaplotypeCaller” method in GATK and then using GenotypeGVCFs-based method with the “-includeNonVariantSites” flag to get the population VCF file including all the confident sites. After that, we first applied the “SelectVariants” to exclude the Indel and split the variant and non-variant sites. Then we applied the hard filter command ‘VariantFiltration’ to exclude potential false-positive variant calls with the following criteria: “-filterExpression ‘QD < 5.0 || FS > 60.0 || MQ < 40.0 || ReadPosRankSum< -8.0 || MQRankSum < -12.5’” and “--genotypeFilterExpression ‘DP < 4.0’”. Additionally, the sites are filtered out if there is a 'N' is in the reference sequence; if the site is fixed difference to the reference genome or if the site including more than 20% missing genotypes. For non-variant sites, we filtered the sites if there is a 'N' is in the reference sequence or if the site including more than 20% missing genotypes. All the SNPs were annotated by ANNOVAR v2013-06-21 (ANNOVAR, RRID:SCR_012821) [70] (Supplementary Table 2). For each individuals, the heterozygosity was calculated as heterozygous SNP rate across the whole genome based on the whole number of sites that are callable (Supplementary Table 3).

Genetic Diversity and Structure Analysis

A neighbor-jointing (NJ) tree was constructed for the 81 individuals based on the autosomal genome data using the software TreeBeST. The bootstrap was set to 1,000 times to assess branch support, with the genome information of Indian RMs and *M. sylvanus* as outgroups. FigTree (http://tree.bio.ed.ac.uk/software/figtree/, v1.4.0) was used to visualize the phylogenetic tree (Fig. 1b and Supplementary Fig. 4). Population structure analysis was performed using the software STRUCTURE 2.3.4 [25], which estimates individual ancestry and admixture proportions assuming *K* ancestral populations. We ran STRUCTURE five times to assess convergence and tested the number of genetic clusters (*K*) from 2-9 (Supplementary Fig. 5). We also carried out a principle component analysis (PCA) using the smartPCA program from the Eigensoft package, v5.0 (Eigensoft, RRID:SCR_004965) [71]. To determine the significance level of principal components, a Tracy-Widom test was done after the PCA (Supplementary Table 4). Decay of linkage disequilibrium against physical distance for the different populations was calculated using the Haploview software [72] with the maxdistance set as 500kb (Supplementary Fig. 15).

Demographic and Divergence Inference Using PSMC and Fastsimcoal2

We called the consensus sequences using Samtools mpileup [68] by applying: "samtools mpileup -q 1 -C 50 -S -D -m 2 -F 0.002 -u -f *.fa(genome) *.bam | bcftools view -c - | vcfutils.pl vcf2fq -d 10 -D 100 -Q 20 - > *.psmc.fq" and "fq2psmcfa -q10 -s 100 *.psmc.fq >*.psmc.fa" . To ensure the quality of consensus sequences, we used data of ten individuals with an average coverage >20× (22.20-34.32×). The PSMC model [26] was used to estimate the population histories from the individual genomes (sex chromosomes excluded) with the following parameters: –N30 –t15 –r5 –p '4+25×2+4+6'. We chose a generation length of 11 years and a mutation rate per generation (μ) of 1.0×10^{-8} (for the rationale to use these values see [6, 73]).

We used PAUP* 4.0a142 (PAUP, RRID:SCR_014931) [30] to run SVDquartets to estimate the branching pattern among the five subspecies with the following command: SVDQuartets SpeciesTree=yes bootstrap evalQuartets=all seed=0 nthreads=40. The joint site frequency spectrum (SFS) approach implemented in *fastsimcoal2* [32] was performed to model more recent demographic fluctuations and respective divergence times based on the species tree estimation by SVDquartets. VCF file containing callable variant sites was used converted to fastsimcoal style folded SFS. To mitigate the effect of linkage disequilibrium, we filtered out the SNPs located within 10 kb from genes and then we took one SNPs every 10kb randomly. The multidimensional folded SFS for all the five subspecies is generated by easySFS (https://github.com/isaacovercast/easySFS#easysfs). During likelihood calculation, a conditional maximization algorithm (ECM) is used to maximize the likelihood of each parameter while keeping the others stabilized. This ECM procedure runs through 40 cycles where each composite-likelihood was calculated using 100,000 coalescent simulations. Additionally, in order to avoid likelihood estimates that oversample parameter values at local maxima across the composite likelihood surface, we ran 50 replicates with each starting from different initial conditions. We chose the replicate with the highest estimated maximum likelihood score to estimate confidence intervals using parametric bootstrapping. The SFS used in bootstrap was simulated with the parameter values from the highest likelihood model and then new parameter values re-estimated from the simulated SFS. We ran 100 parametric bootstraps (Supplementary Fig. 7).

Positive Selection

To identify genomic regions that may have been subject to selection for each subspecies inhabited in different habitats, we scanned the genome using one-to-one pair-wise comparisons between all five subspecies. We calculated the genome-wide distribution of F_{ST} values [74] and θ_π ratios for each pairwise comparison among five RM subspecies. We calculated θ_π for each population and the F_{ST} between the two populations in each comparison using VCFtools (VCFtools, RRID:SCR_001235) [75] with a genome-wide sliding window strategy (50-kb in length with 25-kb step). The F_{ST} values were Z-transformed and the log value of θ_π ratio ($\theta_\pi 2$ / $\theta_\pi 1$) was estimated. Candidate regions under positive selection were extracted based on the top 5% of log-odds ratios for both Z (F_{ST}) and log (θ_π–ratio). Finally, for each subspecies we used the intersection of putatively selected regions generated by all the pair-wise comparisons with other subspecies as the candidate regions under positive selection (i.e. consistent signatures of selective sweeps). Genes located in these regions are expected to represent targets of selection. Functional classification and enrichment analysis of GO categories and KEGG pathways for these candidate genes were performed using DAVID v6.8 (DAVID, RRID:SCR_001881) [76]. The modified Fisher Exact *P*-value cut off was 0.05. Chi-square and *P*-values for the allele frequencies in *M. m. tcheliensis* vs. *M. m. brevicaudus* for the re-sequenced SNPs from the candidate genes were assessed with the Haploview program [72].

Genomic divergence and implication for biomedical research

A total of 118 out of 52,534,348 RM SNPs analyzed in this study were successfully mapped to human reference sequence version hg19 (GRCh37) using liftOver (https://genome.ucsc.edu/cgi-bin/hgLiftOver) and were annotated as 'disease causing' in HGMD (version 2015.1) or pathogenic in ClinVar (downloaded 25/02/2018) (Supplementary Table 12).

Data Availability

All data generated from this study have been submitted to the NCBI Sequence Read Archive (SRA) under BioProject PRJNA345528. The datasets supporting the results of this article are available in the *GigaScience* GigaDB repository [77].

Competing interests

The authors declare that they have no competing interests.

Acknowledgments

This project was sponsored by the following grants: Ming Li (Key Project of National Natural Science Foundation of China, 31530068; Strategic Priority Research Program of the Chinese Academy of Sciences, XDA19050202 and XDB31000000; National Key R&D Program of China, 2016YFC0503200; Creative Research Group Project of NSFC; and Science & Technology Department of Sichuan Province, 2018JZ0008); Zhijin Liu (Natural Science Foundation of China, 31471989). The authors thank Baoguo Li, Meng Yao, Songtao Guo, Jiqi Lu, Zhenlong Wang, Xuelong Jiang, Tao Meng and Qihai Zhou for their help in sampling; Daniel Pitt, Quan Kang, Qi Wu, Chuanyun Li and Qi Pan for their assistance in data analysis.

Author contributions

M. L., Z. L. and M. B conceived the study and designed the project. Z. L., X. T., P. O., X. Z., L. Z. and S. T. managed the project, performed the analyses and wrote the manuscript. Z. L., B. S. and H. X. prepared samples. Z. L., X. T. and P. O. performed genetic analyses. Z. L., X. T., P. O., B. R., L. Z., G. L., Z. Y., Z. P., Z. X., C. R., M. B. and M. L. discussed the data. Z. L. and X. T. wrote the manuscript with contributions from P. O., B. W., H. X., W. Z., C. R., M. B. and M. L.; all authors contributed to data interpretation.

Supplementary Material

Supplementary information, figures S1-S15 and tables S1-S13 are available on line.

References

1. Moreno-Estrada A, Gignoux CR, Fernández-López JC et al. Human genetics. The genetics of Mexico recapitulates Native American substructure and affects biomedical traits. Science 2013; **344**:1280–1285.
2. Allentoft ME, Sikora M, Sjögren KG et al. Population genomics of Bronze Age Eurasia. Nature 2015; **522**:167–172.
3. Sudmant PH, Rausch T, Gardner EJ et al. An integrated map of structural variation in 2,504 human genomes. Nature 2015; **526**:75–81.
4. Maestripieri D. Macachiavellian intelligence: How rhesus macaques and humans have conquered the world. 2007. The University of Chicago Press, Chicago.
5. Zinner D, Fickenscher GH, Roos C. Family Cercopithecidae (Old World Monkeys). Handbook of the Mammals of the World. 2013; Pp. 550-753 in: Mittermeier RA, Rylands AB, Wilson DE. eds. Vol. 3. Primates. Lynx Edicions, Barcelona.
6. Xue C, Raveendran M, Harris RA et al. The population genomics of rhesus macaques (*Macaca mulatta*) based on whole genome sequences. Genome Res 2016; **26**:1651–1662.
7. Zhong X, Peng J, Shen QS et al. RhesusBase PopGateway: Genome-Wide Population Genetics Atlas in Rhesus Macaque. Mol Biol Evol 2016; **33**:1370–1375.
8. Fawcett GL, Raveendran M, Deiros DR et al. Characterization of single-nucleotide variation in Indian-origin rhesus macaques (*Macaca mulatta*). BMC Genomics 2011; **12**:311.
9. Yan G, Zhang G, Fang X et al. Genome sequencing and comparison of two nonhuman primate animal models, the cynomolgus and Chinese rhesus macaques. Nature Biot 2011; **29**:1019-1023.
10. Haus T, Ferguson B, Rogers J et al. Genome typing of nonhuman primate models: implications for biomedical research. Trends Genet 2014; **30**:482–487.
11. Flynn S, Satkoski J, Lerche N et al. Genetic variation at the TNF-alpha promotor and malaria susceptibility in rhesus (*Macaca mulatta*) and long-tailed (*Macaca fascicularis*) macaques. Infect Genet Evol 2009; **9**:769–777.
12. de Groot NG, Heijmans CMC, Koopman G et al. TRIM5 allelic polymorphism in macaque species/populations of different geographic origins: its impact on SIV vaccine studies. Tissue

Antigens. 2011; **78**:256–62.

13. Hernandez RD, Hubisz MJ, Wheeler DA et al. Demographic histories and patterns of linkage disequilibrium in Chinese and Indian rhesus macaques. Science 2007; **316**:240–243.
14. Champoux M, Higley JD, Suomi SJ. Behavioral and physiological characteristics of Indian and Chinese-Indian hybrid rhesus macaque infants. Dev Psychobiol 1997; **31**:49–63.
15. Trichel AM, Rajakumar PA, Murphey-Corb M. Species-specific variation in SIV disease progression between Chinese and Indian subspecies of rhesus macaque. J Med Primatol 2002; **31**:171–178.
16. Tosi AJ, Morales JC, Melnick DJ. Paternal, maternal, and biparental molecular markers provide unique windows onto the evolutionary history of macaque monkeys. Evolution 2003; **57**:1419–1435.
17. Smith DG. Genetic characterization of Indian-origin and Chinese-origin rhesus macaques (*Macaca mulatta*). Comp Med 2005; **55**:227–230.
18. Ferguson B, Street SL, Wright H et al. Single nucleotide polymorphisms (SNPs) distinguish Indian-origin and Chinese-origin rhesus macaques (Macaca mulatta). BMC Genomics 2007; **8**:43.
19. Kubisch HM, Falkenstein KP, Deroche CB et al. Reproductive efficiency of captive Chinese- and Indian-origin rhesus macaque (*Macaca mulatta*) females. Am J Primatol 2012; **74**:174–184.
20. Kanthaswamy S, Johnson Z, Trask JS et al. Development and validation of a SNP-based assay for inferring the genetic ancestry of rhesus macaques (*Macaca mulatta*). Am J Primatol 2014; **76**:1105–1113.
21. Fooden J. Systematic review of the rhesus macaque, *Macaca mulatta* (Zimmermann, 1780). Field Zool 2000; **96**:1–180.
22. Jiang X, Wang Y, Ma S. Taxonomic revision and distribution of subspecies of rhesus monkey (*Macaca mulatta*) in China. Zool Res 1991; **12**:241–247.
23. Fang X, Zhang Y, Zhang R et al. Genome sequence and global sequence variation map with 5.5 million SNPs in Chinese rhesus macaque. Genome Biol 2011; **12**:R63.
24. Prado-Martinez J, Sudmant PH, Kidd JM et al. Great ape genetic diversity and population history. Nature 2013; **499**:471–475.

25. Evanno G, Regnaut S, Goudet J. Detecting the number of clusters of individuals using the software STRUCTURE: a simulation study. Mol Ecol 2005; **14**:2611–2620.
26. Li H, Durbin R. Inference of human population history from individual whole-genome sequences. Nature 2011; **475**:493–496.
27. Zheng B, Xu Q, Shen Y. The relationship between climate change and Quaternary glacial cycles on the Qinghai–Tibetan Plateau: review and speculation. Quatern Int 2002; **97**:93–101.
28. Chifman J, Kubatko L. Identifiability of the unrooted species tree topology under the coalescent model with time-reversible substitution processes, site-specific rate variation, and invariable sites. J Theor Biol 2014; **374**:35-47.
29. Chifman J, Kubatko L. Quartet inference from SNP data under the coalescent model. Bioinformatics 2014; **30**:3317-3324.
30. Swofford, D, et al; PAUP*. Phylogenetic Analysis Using Parsimony (*and other methods). Version 4. Sinauer Associates, Sunderland, Massachussets. 2003.
31. Song S, Liu L, Edwards SV, Wu S. Resolving conflict in eutherian mammal phylogeny using phylogenomics and the multispecies coalescent model. Proc Natl Acad Sci USA 2012; **109**: 14942-14947.
32. Excoffier, L. Dupanloup I, Huerta-Sánchez E et al Robust demographic inference from genomic and SNP data. PLoS Genet 2013; **9**:e1003905.
33. Owen LA, Finkel RC, Caffee MW. A note on the extent of glaciation throughout the Himalaya during the global Last Glacial Maximum. Quaternary Sci Rev 2002; **21**:147–157.
34. Owen LA. Latest Pleistocene and Holoene glacier fluctuations in the Himalaya and Tibet. Quaternary Sci Rev 2009; **28**:2150–2164.
35. Wu S, Luo J, Li Q et al. Ecological genetics of Chinese rhesus macaque in response to mountain building: all things are not equal. PLoS ONE 2013; **8**:e55315.
36. Yi X, Liang Y, Huerta-Sanchez E et al. Sequencing of 50 human exomes reveals adaptation to high altitude. Science 2010; **329**:75–78.
37. Bhatia G, Patterson N, Pasaniuc B et al. Genome-wide comparison of African-ancestry populations from CARe and other cohorts reveals signals of natural selection. Am J Hum Genet 2011; **89**:368–381.

38. Zhao SC, Zheng PP, Dong SS et al. Whole-genome sequencing of giant pandas provides insights into demographic history and local adaptation. Nat Genet 2013; **45**:67–71.
39. Tian JD, Wang ZL, Lu JQ, Wang BS, Chen JR. Reproductive Parameters of Female Macaca mulatta tcheliensis in the Temperate Forest of Mount Taihangshan, Jiyuan, China. Am J Primatol 2013; **75**:605–612.
40. Zhao X, Zhang H, Lv X et al. Survey and research of morphological characters of monkeys (*Macaca mulatta*) in the Taihang Mountains. J Henan Nor Uni 1989; **62**:120–125.
41. Lu JQ, Hou JH, Wang HF, Qu WY. Current status of *Macaca mulatta* in Taihangshan Mountains Area, Jiyuan, Henan, China. Int J Primatol 2007; **28**:1085–1091.
42. Sadava DE, Heller HC, Orians GH, Purves WK, Hillis DM. Life: The Science of Biology, 8th edn. Macmillan, New York; 2008.
43. Drew RE, Rodnick KJ, Settles M et al. Effect of starvation on transcriptomes of brain and liver in adult female zebrafish (Danio rerio). Physiol Genomics 2008; **35**:283–295.
44. Bergmann C. Über die Verhältnisse der Wärmeökonomie der Thiere zu ihrer Grösse. Göttinger Studien 1847; **3**:595–708.
45. Zhang P, Lyu MY, Wu CF et al. Variation in body mass and morphological characters in Macaca mulatta brevicaudus from Hainan, China. Am J Primatol 2016; **78**:679–698.
46. Jevsinek Skok D, Kunej T, Kovac M et al. FTO gene variants are associated with growth and carcass traits in cattle. Animal Genetics 2016; **47**: 219–222.
47. Gao X, Shin YH, Li M, Wang F, Tong Q, Zhang PM. The Fat Mass and Obesity Associated Gene FTO Functions in the Brain to Regulate Postnatal Growth in Mice. PLoS ONE 2010; **5**: e14005.
48. Dy P, Smits P, Silvester A et al. Synovial joint morphogenesis requires the chondrogenic action of Sox5 and Sox6 in growth plate and articular cartilage. Dev Biol 2010; **341**:346–359.
49. Greally MT, Kalis NN, Agab W et al. Autosomal recessive cutis laxa type 2A (ARCL2A) mimicking Ehlers‐Danlos syndrome by its dermatological manifestations: Report of three affected patients. Am J Med Genet A 2014; **164A**:1245–1253.
50. Morimoto K, Shimizu T, Furukawa K, Morio H, Kurosawa H, Shirasawa T. Transgenic expression of the EXT2 gene in developing chondrocytes enhances the synthesis of heparan

sulfate and bone formation in mice. Biochem Biophys Res Commun 2002; **292**: 999–1009.

51. Salazar VS, Gamer LW, Rosen V. BMP signaling in skeletal development, disease and repair. Nat Rev Endocrinology 2016; **12**:203–221.
52. Bragdon B, Moseychuk O, Saldanha S et al. Bone Morphogenetic Proteins: A critical review. Cell Signal 2011; **23**: 609–620.
53. Ruvinsky A, Flood WD, Costantini F. Developmental mosaicism may explain spontaneous reappearance of the AxinFu mutation in mice. Genesis 2001; **29**: 49-57.
54. Lu QL, Yao YH, Yao YF et al. Angiogenic Factor AGGF1 Promotes Therapeutic Angiogenesis in a Mouse Limb Ischemia Model. PLoS ONE 2012; 7: e46998.
55. González-Alonso J. Human thermoregulation and the cardiovascular system. Exp Physiol 2012; **97**:340–346.
56. Meadows JRS, Lindblad-TohK. Dissecting evolution and disease using comparative vertebrate genomics. Nat Rev Genet 2017; 18, 624–636.
57. Dick DM, Bierut LJ. Curr Psychiatry Rep 2006; **8**: 151.
58. Horn KE, Glasgow SD, Gobert D et al. DCC expression by neurons regulates synaptic plasticity in the adult brain. Cell Rep 2010; **31**:173-185.
59. Hori K, Hoshino M. Neuronal Migration and AUTS2 Syndrome. Brain Sci 2017; **7**:e54.
60. Hazen MM, Woodward AL, Hofmann I et al. Mutations of the hemophagocytic lymphohistiocytosis-associated gene UNC13D in a patient with systemic juvenile idiopathic arthritis. Arthritis Rheum 2008; **58**:567–570.
61. Procter M, Wolf B and Mao R. Forty-eight novel mutations causing biotinidase deficiency. Mol Genet Metab 2016; **117**:369–372.
62. Munier S, Delcroix-Genete D, Carthagena L et al. Characterization of two candidate genes, NCoA3 and IRF8, potentially involved in the control of HIV-1 latency. Retrovirology 2005; **2**:73.
63. Fan ZY, Song YL. Chinese Primate Status and Primate Captive Breeding for Biomedical Research in China. In: Institute for Laboratory Animal Research, National Research Council. International Perspectives: The Future of Nonhuman Primate Resources. Washington DC: National Academy Press. 2003.
64. Hao Xin. Monkey Research in China: Developing a Natural Resource. Cell 2007; **129**: 1033–

1036.
65. Zhou Q. Balancing the welfare: the use of non-human primates in research. Trends Genet 2014; **30**: 476–478.
66. Gradnigo JS, Majumdar A, Norgren RB Jr, Moriyama EN. Advantages of an Improved Rhesus Macaque Genome for Evolutionary Analyses. PLoS ONE 2016; **11**: e0167376.
67. Li H, Durbin R. Fast and accurate short read alignment with Burrows-Wheeler transform. Bioinformatics 2009; **25**:1754–1760.
68. Li H, Handsaker B, Wysoker A et al. The sequence alignment/map format and SAMtools. Bioinformatics 2009; **25**:2078–2079.
69. Mckenna A, Hanna M, Banks E, et al. The Genome Analysis Toolkit: a MapReduce framework for analyzing next-generation DNA sequencing data. Genome Res 2010; **20(9)**:1297–303.
70. Wang K, Li M, Hakonarson H. ANNOVAR: functional annotation of genetic variants from high-throughput sequencing data. Nucleic Acids Res 2010; **38**: e164.
71. Patterson N, Price AL, Reich D. Population structure and eigenanalysis. PLoS Genet 2006; **2**:e190.
72. Barrett JC, Fry B, Maller J et al. Haploview: analysis and visualization of LD and haplotype maps. Bioinformatics 2005; **21**:263–265.
73. Ségurel L, Wyman M J, Przeworski M. Determinants of mutation rate variation in the human germline. Annu Rev Genomics Hum Genet 2014; 15: 47–70.
74. Weir BS, Cockerham CC. Estimating *F*-statistics for the analysis of population structure. Evolution 1984; **38**:1358–1370.
75. Danecek P, Auton A, Abecasis G et al. The variant call format and VCFtools. Bioinformatics 2011; **27**:2156–2158.
76. Huang da W, Sherman BT, Lempicki RA. Systematic and integrative analysis of large gene lists using DAVID Bioinformatics Resources. Nat Protoc 2009; **4**:44–57.
77. Liu Z, Tan X, Orozco-terWengel P, Zhou X, Zhang L, Tian S, et al. Supporting data for “Population genomics of wild Chinese rhesus macaques reveals a dynamic demographic history and local adaptation, with implications for biomedical research” GigaScience

Database 2018. http://dx.doi.org/10.5524/100484.

Tables

Table1. Genetic diversity (θ) and effective population size (N_e) in Chinese rhesus macaques based on segregating sites (θ_W) and nucleotide diversity (θπ).

		Sample size (n)	θ_W		θπ	
			θ	N_e	θ	N_e
Chinese rhesus macaques (all samples)		81	0.00375	93,750	0.00247	61,750
subspecies	*M. m. littoralis*	29	0.00313	78,250	0.00240	60,000
	M. m. tcheliensis	5	0.00215	53,750	0.00230	57,500
	M. m. brevicaudus	5	0.00203	50,750	0.00207	51,750
	M. m. lasiotis	32	0.00298	74,500	0.00239	59,750
	M. m. mulatta	10	0.00303	75,750	0.00245	61,250

Figure Legends

Figure 1. Phylogeny and population genetic structure of 81 wild Chinese RMs. (a) Geographic distribution of RMs in China (gray shadow) and the 17 sampling sites along with their subspecies assignment. (b) Neighbor-joining (NJ) tree and clustering solution inferred using STRUCTURE and displaying five populations (inferred with Evanno's ΔK method; Supplementary Fig. 5). (c) Principal component analysis plots depicting the first two components (variance explained by PC1 = 7.24% and PC2 = 5.69%).

Figure 2. Demographic history and differentiation scenarios of Chinese RMs. (a) Historical changes in effective population size reconstructed using the pairwise sequential Markovian coalescent (PSMC) applied on individual whole genomes for each of the five subspecies. The generation length (g) and the neutral mutation rate per generation (μ) were assumed to be 11 years and 1.08×10^{-8}, respectively. The Xixiabangma Glaciation (XG, 1,200-800 kya), Penultimate Glaciation (PG, 200-130 kya) and Last Glaciation (LG, 70-10 kya) are shaded in gray. (b) Demographic history inferred by *fastsimcoal2*. The width of the gray bars and numbers on them indicate the estimated effective population size (all effective population sizes were converted to individuals). The arrows indicate migration rate between different subspecies. The detailed migration rates are listed in Supplementary Table 5. Numbers at the right show the divergence times between subspecies (all times were converted to years assuming a generation time of 11 years). (c) Biogeographic scenario for RMs. Chinese RMs separates from Indian RMs ~ 162 kya [13], followed by further migration into China by the different RM subspecies indicated with arrows colored following the color key in Fig. 1a.

Figure 3. Genomic regions with selection sweep signals in RM. (a) Distribution of $\log_2$ (θ_π *M. m. lasiotis*/θ_π *M. m. tcheliensis*) and Z (F_{ST}) of 50-kb windows with 25-kb steps. Blue dots located in the selected regions requirement (corresponding to Z test P<0.05, where Z (F_{ST}) >= 1.848 and θ_π log-ratio >= 1.203) represent selected windows for *M. m. tcheliensis*. (b) Morphological comparison between *M. m. tcheliensis* and *M. m. lasiotis*. M and F represent males and females. (c) Example of genes with selection sweep signals. *Ext2, Rpgrip1l, Fbp2* and *Fbp1*in *M. m. tcheliensis* and *Axin1*, *Aggf1* and *Hspa4* in *M. m. brevicaudus*. F_{ST} and θ_π log-ratio between the two subspecies are represented in red and blue, respectively. All values in figure 3c are plotted using 50-kb windows with half steps. Genome annotations are show at the bottom (black bar,

coding sequences (CDS); purple bar, genes). (d) SNP genotypes in putative selective sweeps containing *Ext2*, *Rpgrip1l*, *Fbp2*, *Fbp1*, *Axin1*, *Aggf1* and *Hspa4*. (e) Non-synonymous variants in gene Ext2, *Rpgrip1l* and *Hspa4.*

Figure 4. Population study of putative pathogenic SNPs in Chinese RM subspecies. (a) The site and frequency of pathogenic SNPs located in *Unc13d* and *Btd* genes. (b) Scheme of the *Ncoa3* gene in RM. The positions of nonsynonymous polymorphisms (black) and three amino-acid deletions (in red) are marked. (c) Private and shared pathogenic SNPs in Chinese RM subspecies (blue: *M. m. tcheliensis;* orange: *M. m. brevicaudus;* red: *M. m. littoralis;* green: *M. m. mulatta;* purple: *M. m. lasiotis*). The sizes of the areas are not proportional to the magnitude of the numbers. (d) NJ tree including the 81 Chinese RMs derived from this study, the 26 captive Chinese RMs from Zhong et al. [7] are indicated by blue dot.

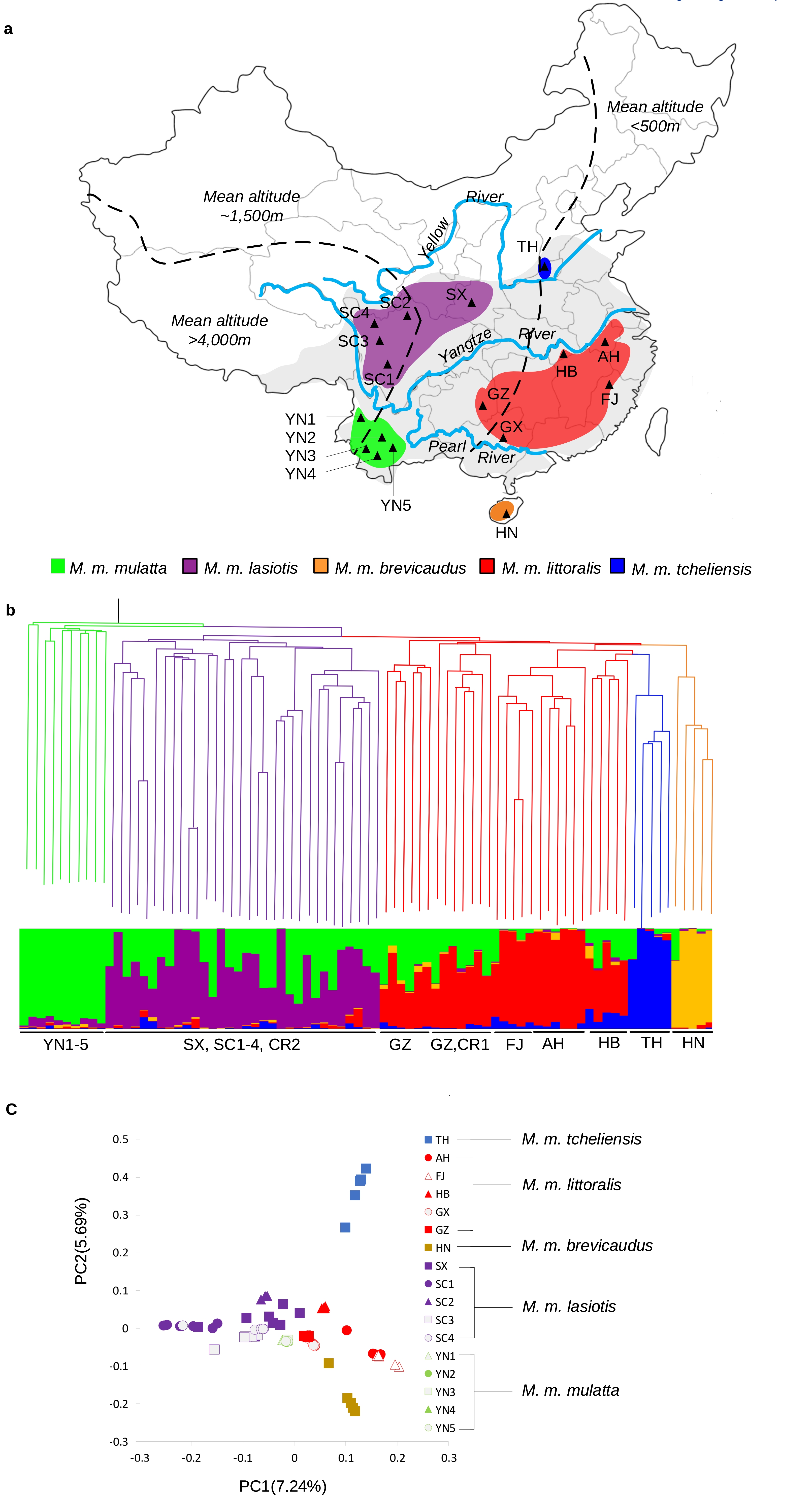
a
Mean altitude <500m
Mean altitude ~1,500m
Mean altitude >4,000m
Yellow River
Yangtze River
Pearl River
TH
SX
SC1
SC2
SC3
SC4
HB
AH
FJ
GZ
GX
YN1
YN2
YN3
YN4
YN5
HN
M. m. mulatta
M. m. lasiotis
M. m. brevicaudus
M. m. littoralis
M. m. tcheliensis
b
YN1-5
SX, SC1-4, CR2
GZ
GZ,CR1
FJ
AH
HB
TH
HN
C
PC2(5.69%)
PC1(7.24%)
0.5
0.4
0.3
0.2
0.1
0
-0.1
-0.2
-0.3
-0.3
-0.2
-0.1
0
0.1
0.2
0.3
TH
AH
FJ
HB
GX
GZ
HN
SX
SC1
SC2
SC3
SC4
YN1
YN2
YN3
YN4
YN5
M. m. tcheliensis
M. m. littoralis
M. m. brevicaudus
M. m. lasiotis
M. m. mulatta

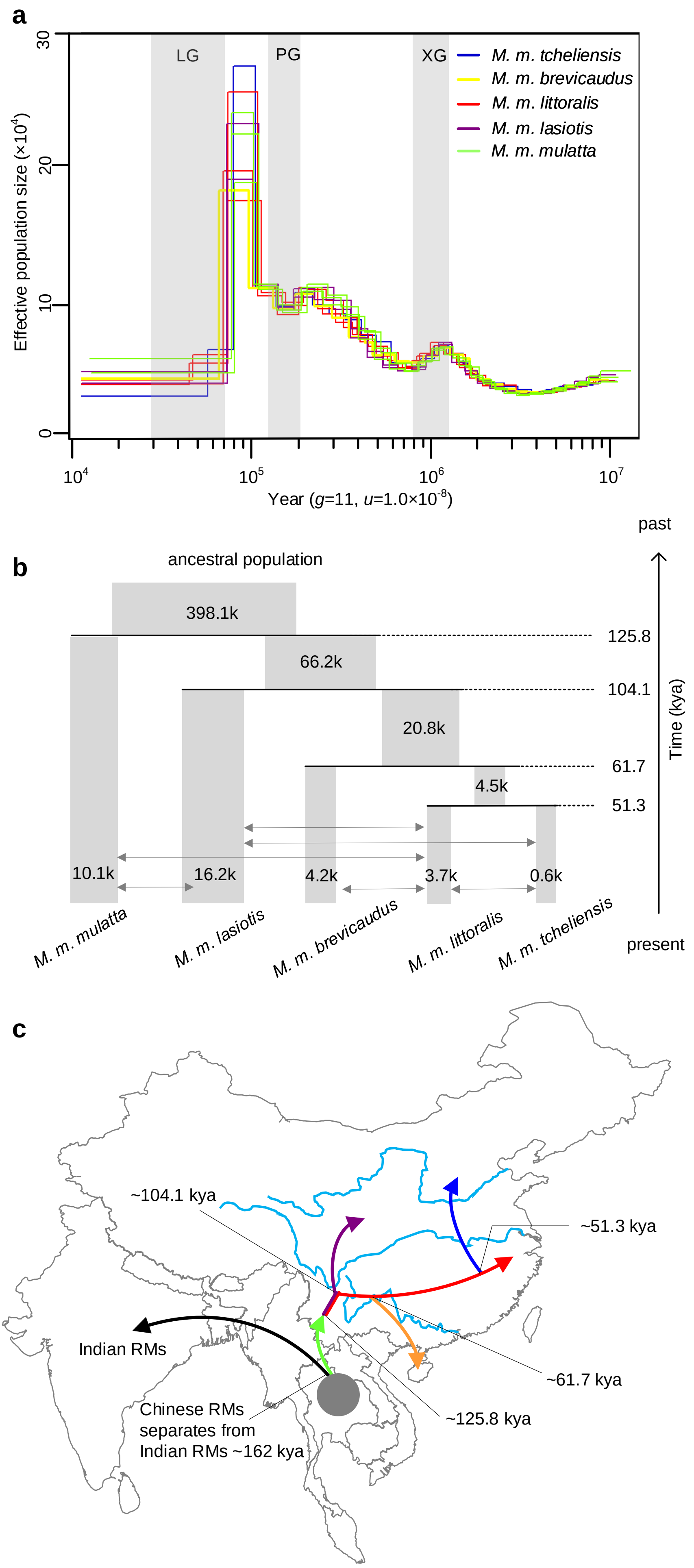
a
LG
PG
XG
M. m. tcheliensis
M. m. brevicaudus
M. m. littoralis
M. m. lasiotis
M. m. mulatta
Effective population size (×10^4)
Year (g=11, u=1.0×10^-8)
b
ancestral population
398.1k
66.2k
20.8k
4.5k
10.1k
16.2k
4.2k
3.7k
0.6k
125.8
104.1
61.7
51.3
past
present
Time (kya)
M. m. mulatta
M. m. lasiotis
M. m. brevicaudus
M. m. littoralis
M. m. tcheliensis
c
~104.1 kya
~51.3 kya
~61.7 kya
~125.8 kya
Indian RMs
Chinese RMs
separates from
Indian RMs ~162 kya

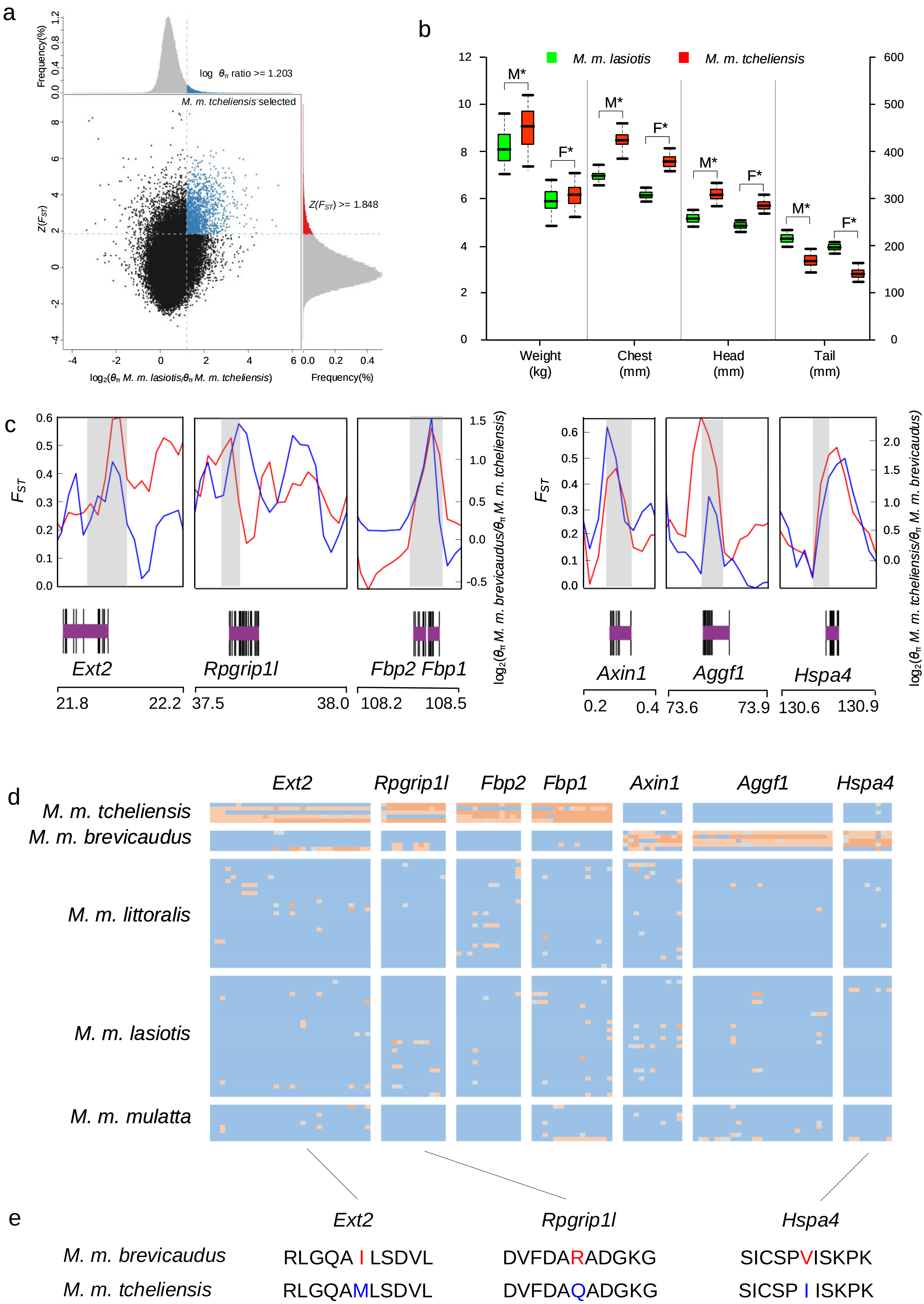

a
log θπ ratio >= 1.203
M. m. tcheliensis selected
Z(FST) >= 1.848
Frequency(%)
Z(FST)
log2(θπ M. m. lasiotis/θπ M. m. tcheliensis)
b
M. m. lasiotis
M. m. tcheliensis
M*
F*
Weight (kg)
Chest (mm)
Head (mm)
Tail (mm)
c
FST
log2(θπ M. m. brevicaudus/θπ M. m. tcheliensis)
log2(θπ M. m. tcheliensis/θπ M. m. brevicaudus)
Ext2
Rpgrip1l
Fbp2 Fbp1
Axin1
Aggf1
Hspa4
21.8
22.2
37.5
38.0
108.2
108.5
0.2
0.4
73.6
73.9
130.6
130.9
d
Ext2
Rpgrip1l
Fbp2
Fbp1
Axin1
Aggf1
Hspa4
M. m. tcheliensis
M. m. brevicaudus
M. m. littoralis
M. m. lasiotis
M. m. mulatta
e
Ext2
Rpgrip1l
Hspa4
M. m. brevicaudus
M. m. tcheliensis
RLGQA I LSDVL
RLGQAMLSDVL
DVFDARADGKG
DVFDAQADGKG
SICSPVISKPK
SICSP I ISKPK

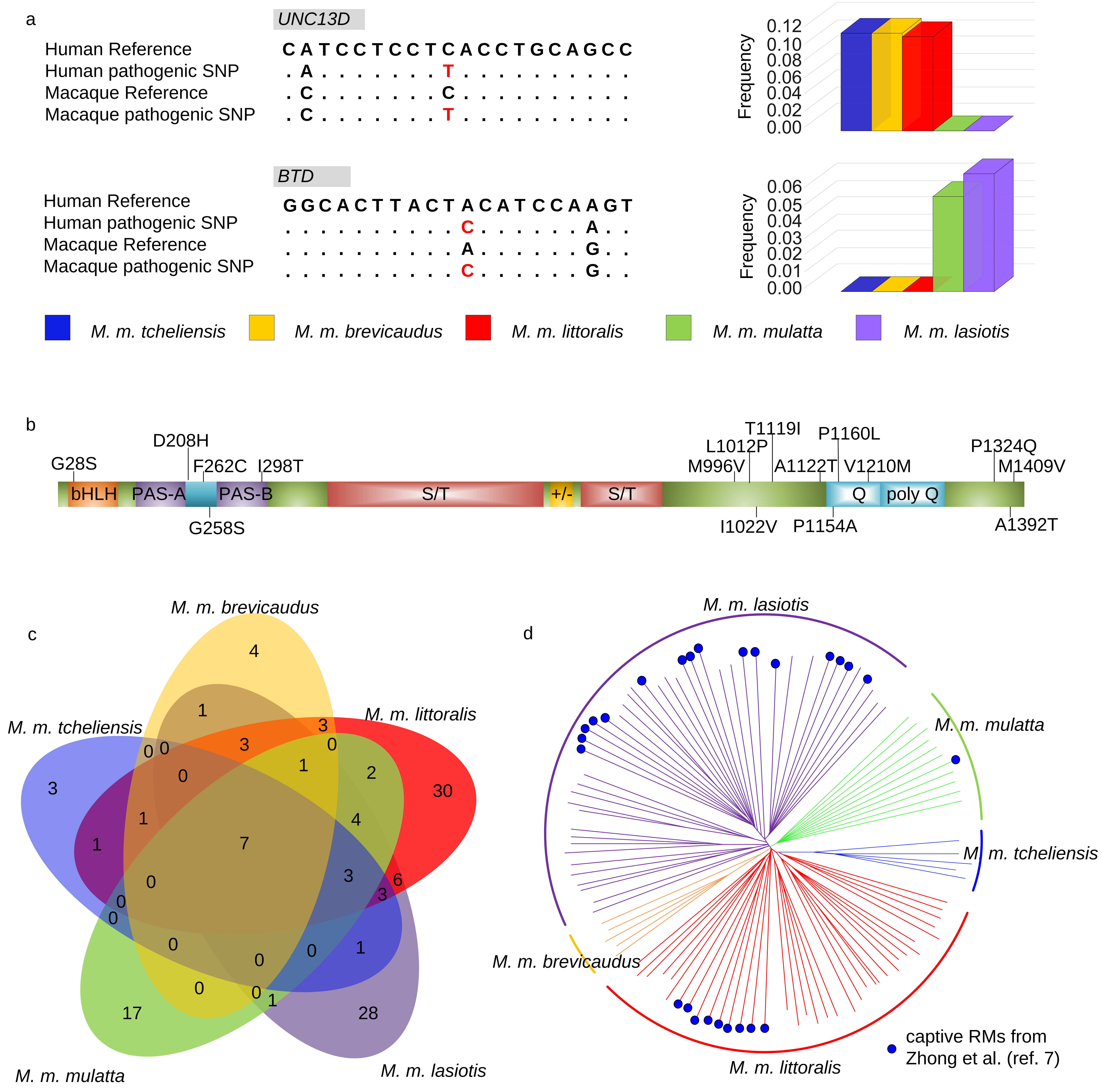
a
UNC13D
Human Reference
Human pathogenic SNP
Macaque Reference
Macaque pathogenic SNP
C A T C C T C C T C A C C T G C A G C C
. A . . . . T . . . .
. C . . . . C . . . .
. C . . . . T . . . .
Frequency
0.00 0.02 0.04 0.06 0.08 0.10 0.12
BTD
Human Reference
Human pathogenic SNP
Macaque Reference
Macaque pathogenic SNP
G G C A C T T A C T A C A T C C A A G T
. . . . C . . . . . . A . .
. . . . A . . . . . . G . .
. . . . C . . . . . . G . .
Frequency
0.00 0.01 0.02 0.03 0.04 0.05 0.06
M. m. tcheliensis
M. m. brevicaudus
M. m. littoralis
M. m. mulatta
M. m. lasiotis
b
G28S
D208H
F262C
I298T
G258S
bHLH
PAS-A
PAS-B
S/T
+/-
S/T
M996V
L1012P
T1119I
A1122T
P1160L
V1210M
I1022V
P1154A
Q
poly Q
P1324Q
M1409V
A1392T
c
M. m. brevicaudus
M. m. tcheliensis
M. m. littoralis
M. m. mulatta
M. m. lasiotis
4
1
3
0
0
3
0
0
1
2
3
30
1
1
4
7
0
3
6
0
3
0
0
1
0
0
0
0
0
1
17
28
d
M. m. lasiotis
M. m. mulatta
M. m. tcheliensis
M. m. brevicaudus
M. m. littoralis
captive RMs from
Zhong et al. (ref. 7)

Click here to access/download
Supplementary Material
renamed_cae38.docx

Click here to access/download
Supplementary Material
Supplemental Data 1.xlsx

Click here to access/download
Supplementary Material
Supplemental Data 2.xlsx
