# Supplementary material for: Population genomics of wild Chinese rhesus macaques reveals a dynamic demographic history and local adaptation, with implications for biomedical research
Source: Gigascience. 2018 Aug 27;7(9):giy106. doi: 10.1093/gigascience/giy106 (PMC6143732; doi:10.1093/gigascience/giy106)
Supplement: Supplement Files [file giy106_supplement_files.zip › Supplementary_Material.pdf]

Supplementary Information

Title: Population genomics of wild Chinese rhesus macaques reveals a dynamic demographic history and local adaptation, with implications for biomedical research

Zhijin Liu[1, †], Xinxin Tan[1, 2, †], Pablo Orozco-terWengel[3, †], Xuming Zhou[1, 4], Liye Zhang[1, 2], Shilin Tian[5], Zhongze Yan[1, 6], Huailiang Xu[7], Baoping Ren[1], Peng Zhang[8], Zuofu Xiang[9], Binghua Sun[10], Christian Roos[11], Michael W. Bruford[3, *], Ming Li[1, 12 *]

[1] CAS Key Laboratory of Animal Ecology and Conservation Biology, Institute of Zoology, Beijing, China.

[2] University of Chinese Academy of Sciences, Beijing 100039, China.

[3] School of Biosciences, Cardiff University, Sir Martin Evans Building, Museum Avenue, Cardiff CF10 3AX, United Kingdom.

[4] Division of Genetics, Department of Medicine, Brigham and Women's Hospital, Harvard Medical School, Boston, MA 02115, USA.

[5] Novogene Bioinformatics Institute, Beijing 100083, China.

[6] Institute of Physical Science and Information Technology, Anhui University, Hefei, 230601, China.

[7] College of Life Science, Sichuan Agricultural University, Ya'an 625014, China.

[8] School of Sociology and Anthropology, Sun Yat-sen University, Guang Zhou, China.

[9] College of Life Science and Technology, Central South University of Forestry and Technology, Changsha 410004, Hunan, China.

[10] School of Life Sciences, Anhui University, Hefei, 230601, China.

[11] Gene Bank of Primates and Primate Genetics Laboratory, German Primate Center, Leibniz Institute for Primate Research, Kellnerweg 4, 37077 Göttingen, Germany.

[12] Center for Excellence in Animal Evolution and Genetics, Chinese Academy of Sciences, Kunming, 650223, China.

† Contributed equally

***** Correspondence: Ming Li, lim@ioz.ac.cn; Michael W. Bruford, BrufordMW@cardiff.ac.uk

Table of content

Supplementary Figures 1

Supplementary Fig. 1. Variant number for the 81 individuals in this study 1

Supplementary Fig. 2. Private and shared SNPs per Chinese RM subspecies 2

Supplementary Fig. 3. Heterozygosity per base-pair for five subspecies of Chinese RMs 3

Supplementary Fig. 4. Neighbor-joining tree derived from 1,000 bootstrap replicates 4

Supplementary Fig. 5. Plots of *ΔK* generated from STRUCTURE results 5

Supplementary Fig. 6. The species tree based on SVDquartets+PAUP* 6

Supplementary Fig. 7. Confidence Intervals from 100 parametric bootstraps for inferred demographic parameters 7

Supplementary Fig. 8. Positive selection flow chart for each subspecies 8

Supplementary Fig. 9. Distribution of Z(F_{ST}) and θ_π log ratio of 50kb windows with 25kb sliding window for *M. m. tcheliensis* 9

Supplementary Fig. 10. Distribution of Z(F_{ST}) and θ_π log ratio of 50kb windows with 25kb sliding window for *M. m. brevicaudus* 10

Supplementary Fig. 11. Distribution of Z(F_{ST}) and θ_π log ratio of 50kb windows with 25kb sliding window for *M. m. littoralis* 11

Supplementary Fig. 12. Distribution of Z(F_{ST}) and θ_π log ratio of 50kb windows with 25kb sliding window for *M. m. lasiotis* 12

Supplementary Fig. 13. Distribution of Z(F_{ST}) and θ_π log ratio of 50kb windows with 25kb sliding window for *M. m. mulatta* 13

Supplementary Fig. 14. Non-synonymous variants in putatively selected genes 14

Supplementary Fig. 15. Linkage disequilibrium pattern of the five Chinese RM subspecies 15

Supplementary Tables 16

Supplementary Table 1. Overview of sample information and sequencing statistics 16

Supplementary Table 2. Distribution of autosomal SNPs within various genomic regions of RM 20

Supplementary Table 3. Identified SNPs and heterozygosity for 81 individuals from 17 sampling locations 21

Supplementary Table 4. Tracy-Widom (*TW*) statistics and *P* values for the ten first eigenvalues in PCA 24

Supplementary Table 5. Inferred demographic parameters with 95% confidence intervals for fastsimcoal2 model 25

Supplementary Table 6. List of positively selected genes in the five Chinese RM subspecies 26

Supplementary Table 7. Enrichment of genes under selective sweep in *M. m. tcheliensis* 28

Supplementary Table 8. Morphological differences between the five investigated Chinese RM subspecies 29

Supplementary Table 9. Enrichment of the genes under selective sweep in *M. m. brevicaudus* 30

Supplementary Table 10. Distribution of SNPs in the selected genes described in the part of "Signatures of selection and local adaptation" 33

Supplementary Table 11. Non-synonymous SNPs with significant differences at the 5% level in the distributions of genotypes between *M. m. tcheliensis* and *M. m. brevicaudus* 34

Supplementary Table 12. List of RM variants scored by HGMD and ClinVar as "disease causing" or "pathogenic" 35

Supplementary Table 13. Population study for *Ncoa3* reveals multiple genotypes 35

Supplementary Figures

Supplementary Fig. 1. Variant number for the 81 individuals in this study. Blue bars are the number of total SNPs for each individual, orange bars are the heterozygous SNPs and the grey bars are the homozygous SNPs.

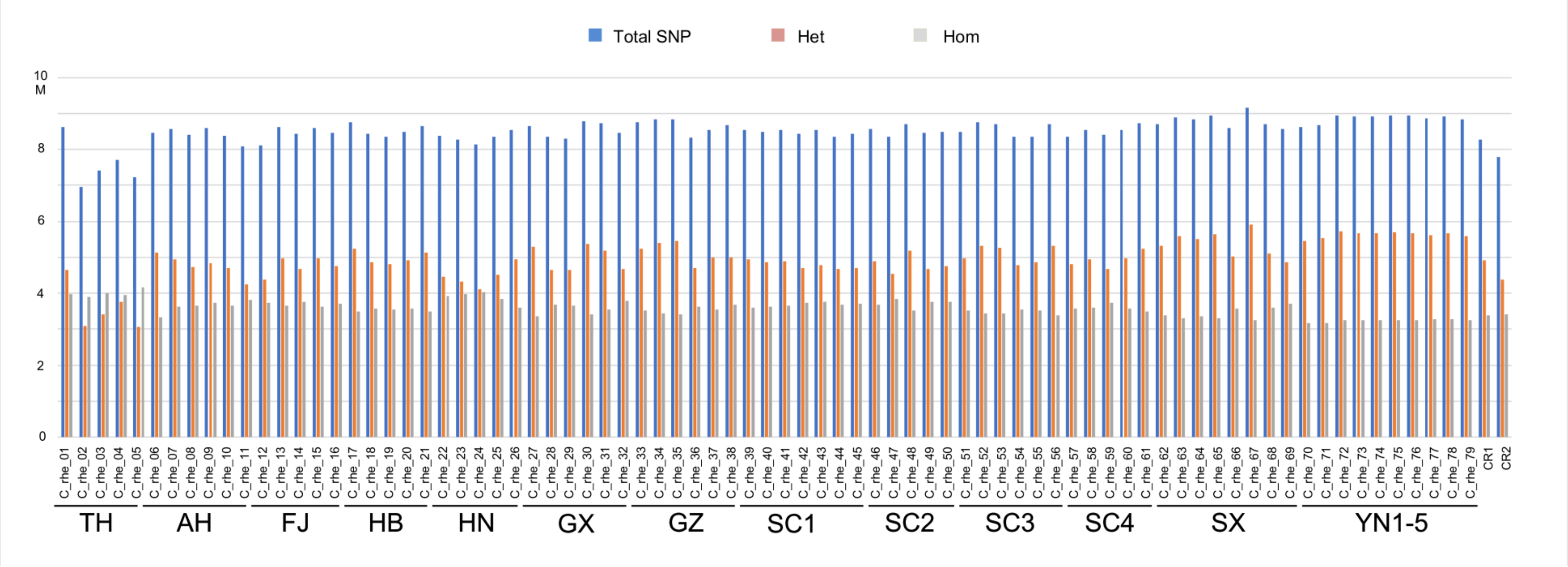

Supplementary Fig. 2. Private and shared SNPs per Chinese RM subspecies (blue: *M. m. tcheliensis;* yellow: *M. m. brevicaudus;* red: *M. m. littoralis;* purple: *M. m. lasiotis;* green: *M. m. mulatta*). The sizes of the areas are not proportional to the magnitude of the numbers. (a) Private and shared total SNPs per subspecies. (b) Genome-wide private and shared non-synonymous SNPs per subspecies.

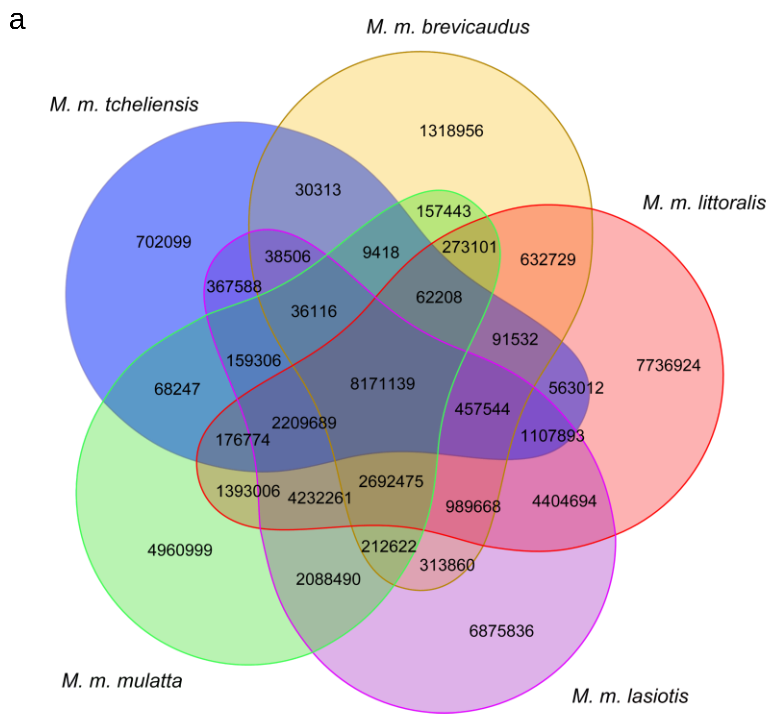

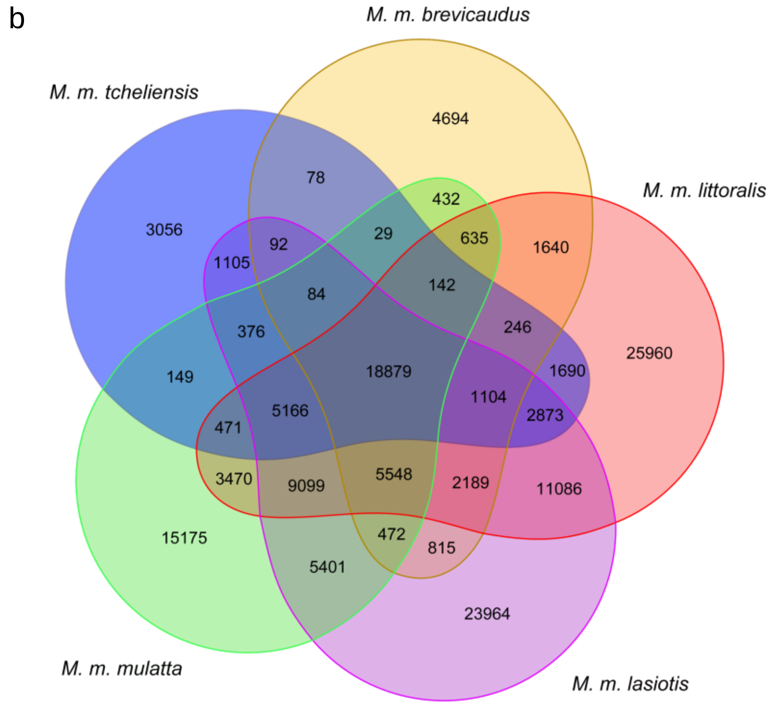

Supplementary Fig. 3. Heterozygosity per base-pair for five subspecies of Chinese RMs.

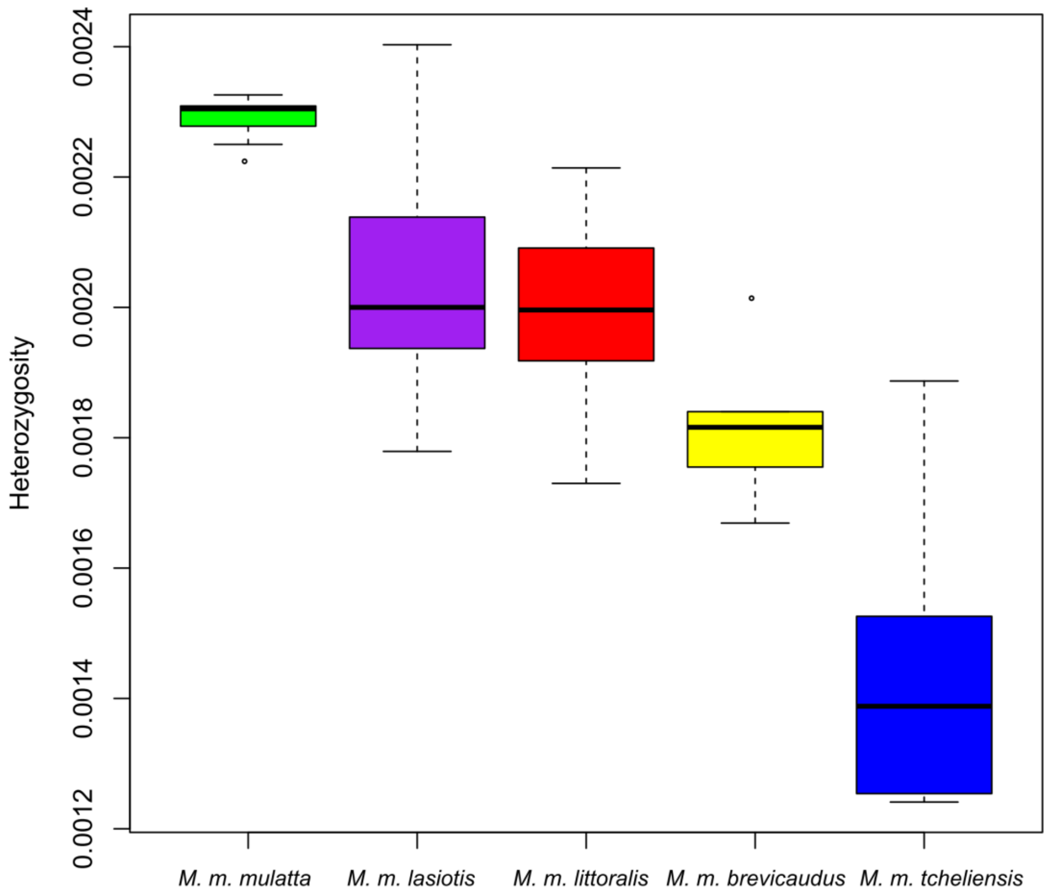

Supplementary Fig. 4. Neighbor-joining tree derived from 1,000 bootstrap replicates based on autosomal SNPs. The Indian RMs (I-977286, I-977280, I-977282, I-977285 and I-977315) and *M. sylvanus* were used as outgroups. 1 - 79 are wild RMs from China. Blue, red, yellow, purple and green represent the different subspecies (*M. m. tcheliensis, M. m. littoralis, M. m. brevicaudus, M. m. lasiotis* and *M. m. mulatta*), respectively (Supplemental Table 1). The data of CR1 and CR2 are from previous publication (Supplemental Table 1).

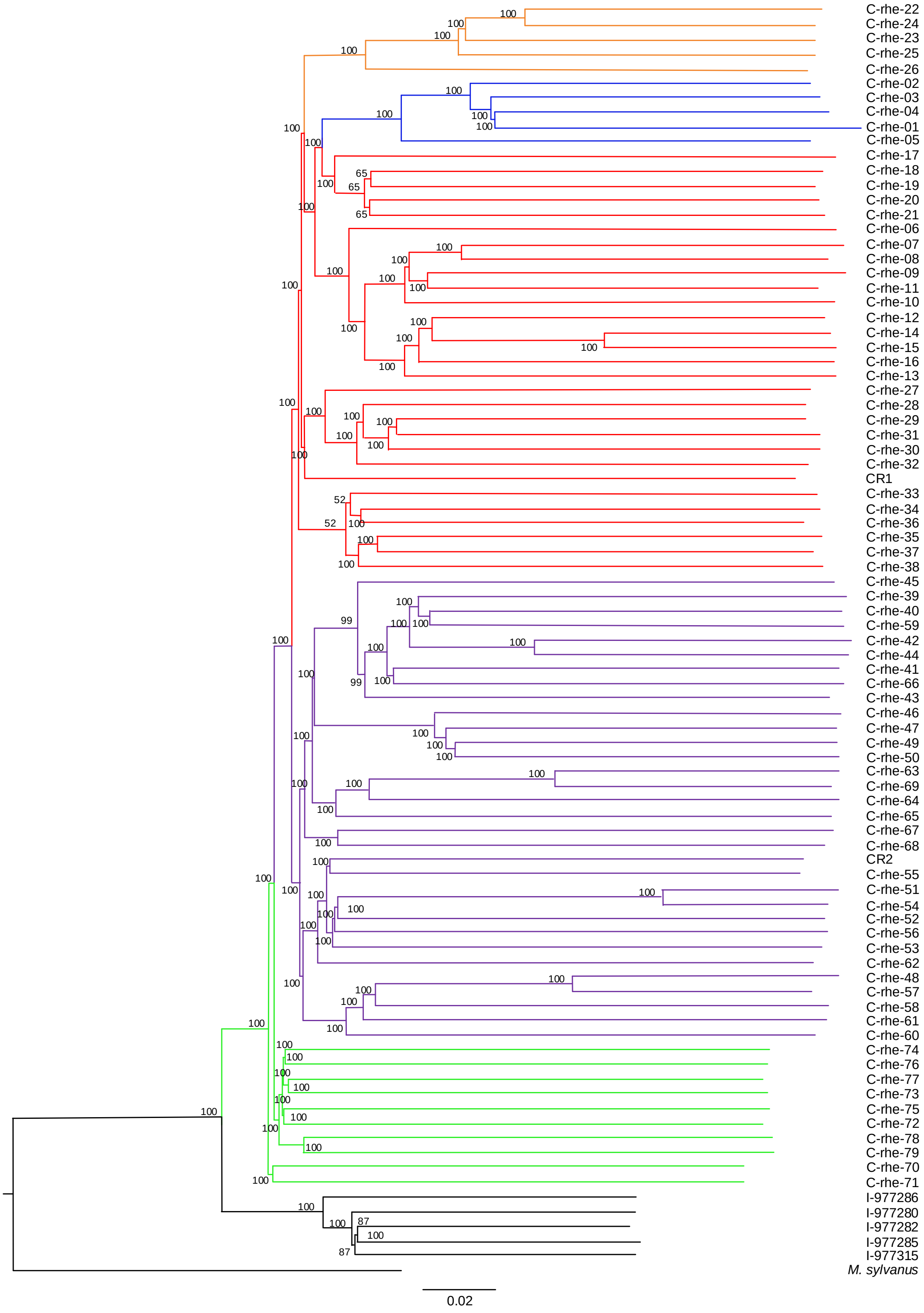

Supplementary Fig. 5. Plots of *ΔK* generated from STRUCTURE results. K represents the number of assumptive ancestral populations.

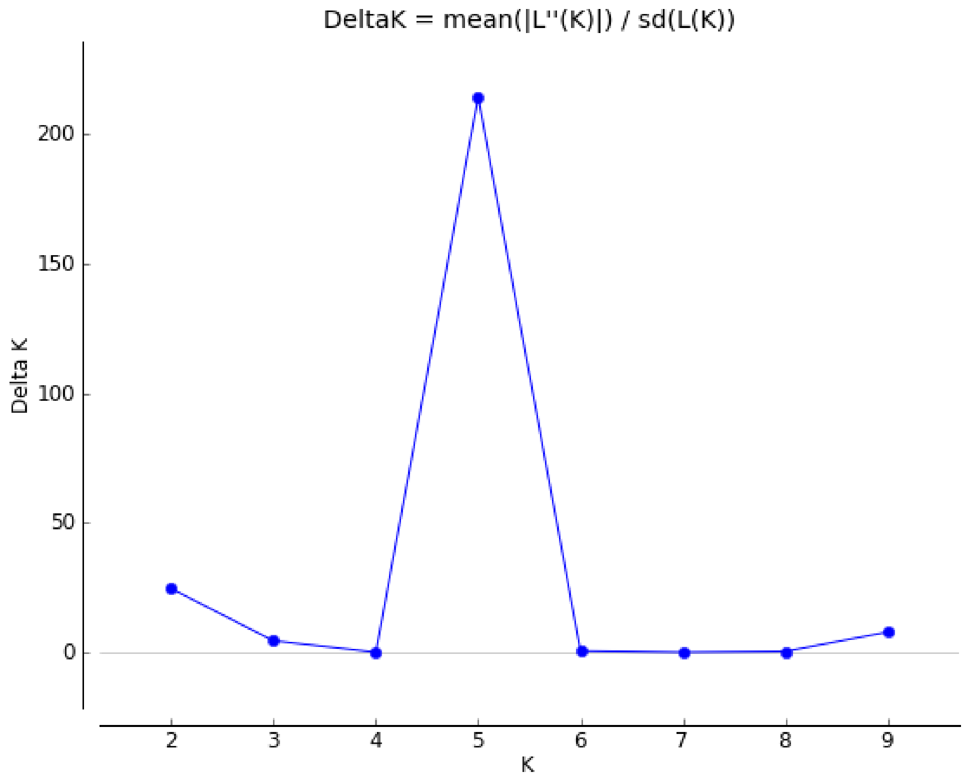

Supplementary Fig. 6. The species tree based on SVDquartets+PAUP*. Labels on branches indicate bootstrap support.

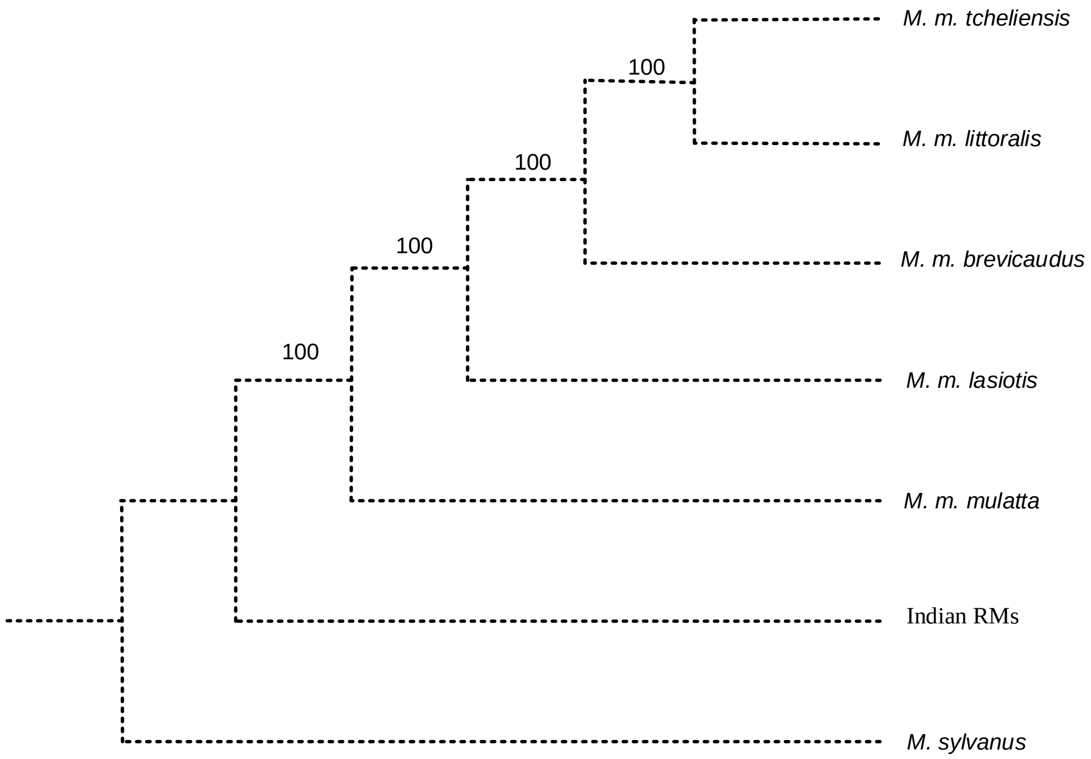

Supplementary Fig. 7. Confidence Intervals from 100 parametric bootstraps for inferred demographic parameters. Red dots represent point estimates from replicates with the highest likelihood. (a) Ne: the effective population sizes of each subspecies and the ancestral populations (all effective population sizes were converted to individuals). (b) Divergence: the divergence time of each subspecies (all times were converted to years assuming a generation time of 11 years). (c) Migration rate: proportion of individuals that move from one population to another per generation.

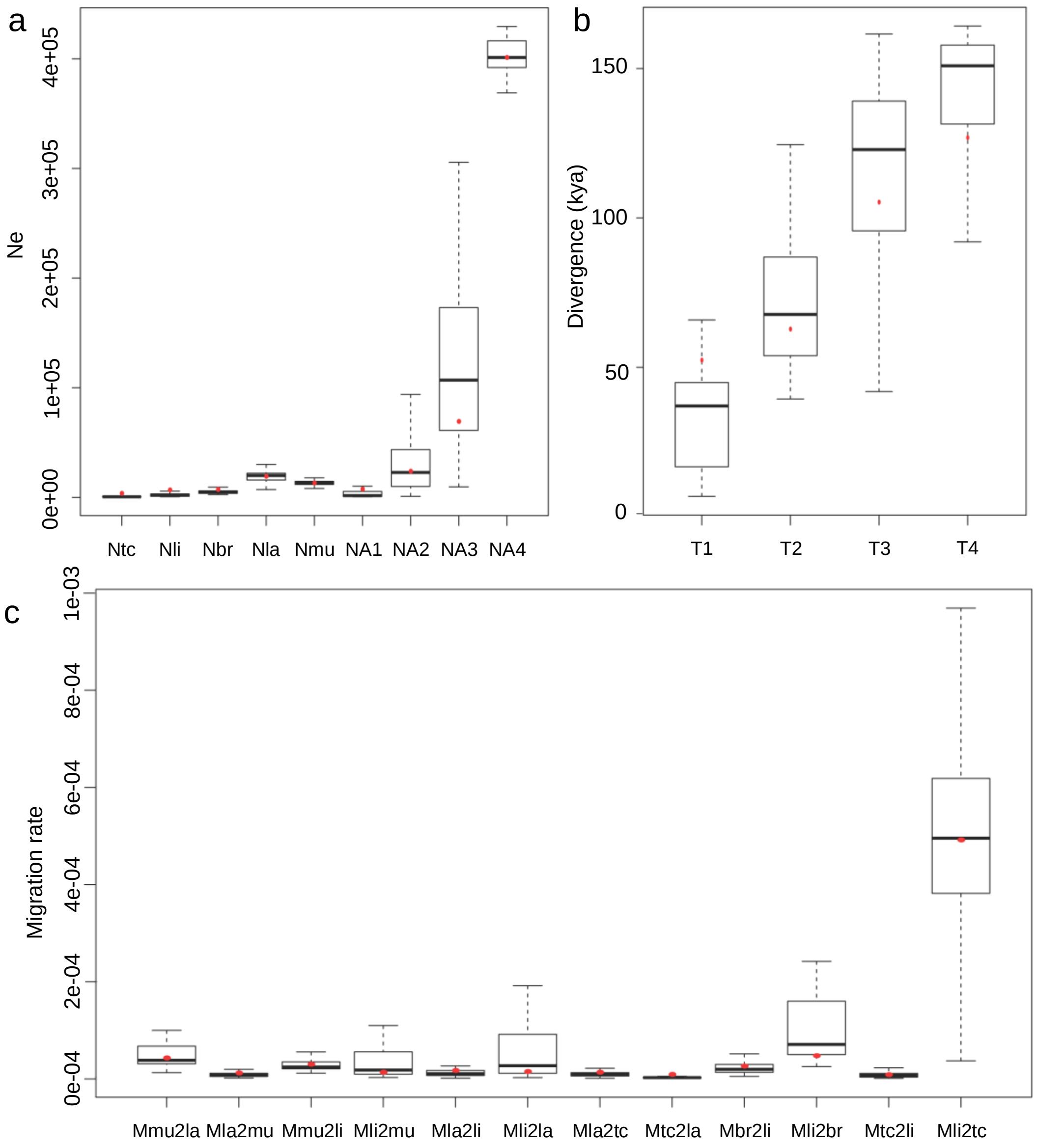

Supplementary Fig. 8. Positive selection flow chart for each subspecies.

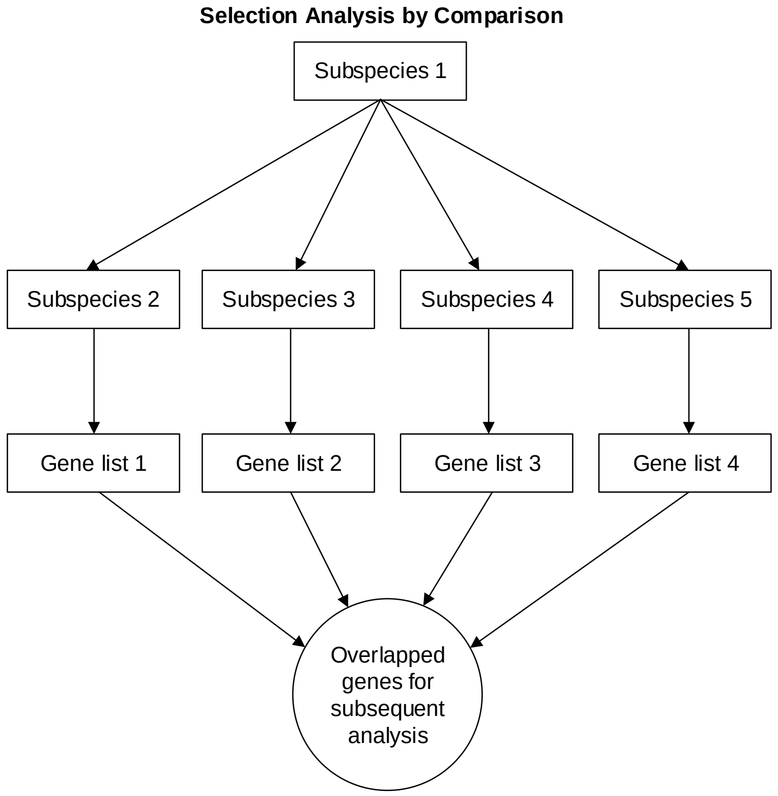

Supplementary Fig. 9. Distribution of Z (F_{ST}) and θ_π log ratio of 50kb windows with 25kb sliding window for *M. m. tcheliensis* (a) comparison against *M. m. brevicaudus*, (b) comparison against *M. m. littoralis*, (c) comparison against *M. m. lasiotis*, (d) comparison against *M. m. mulatta*.

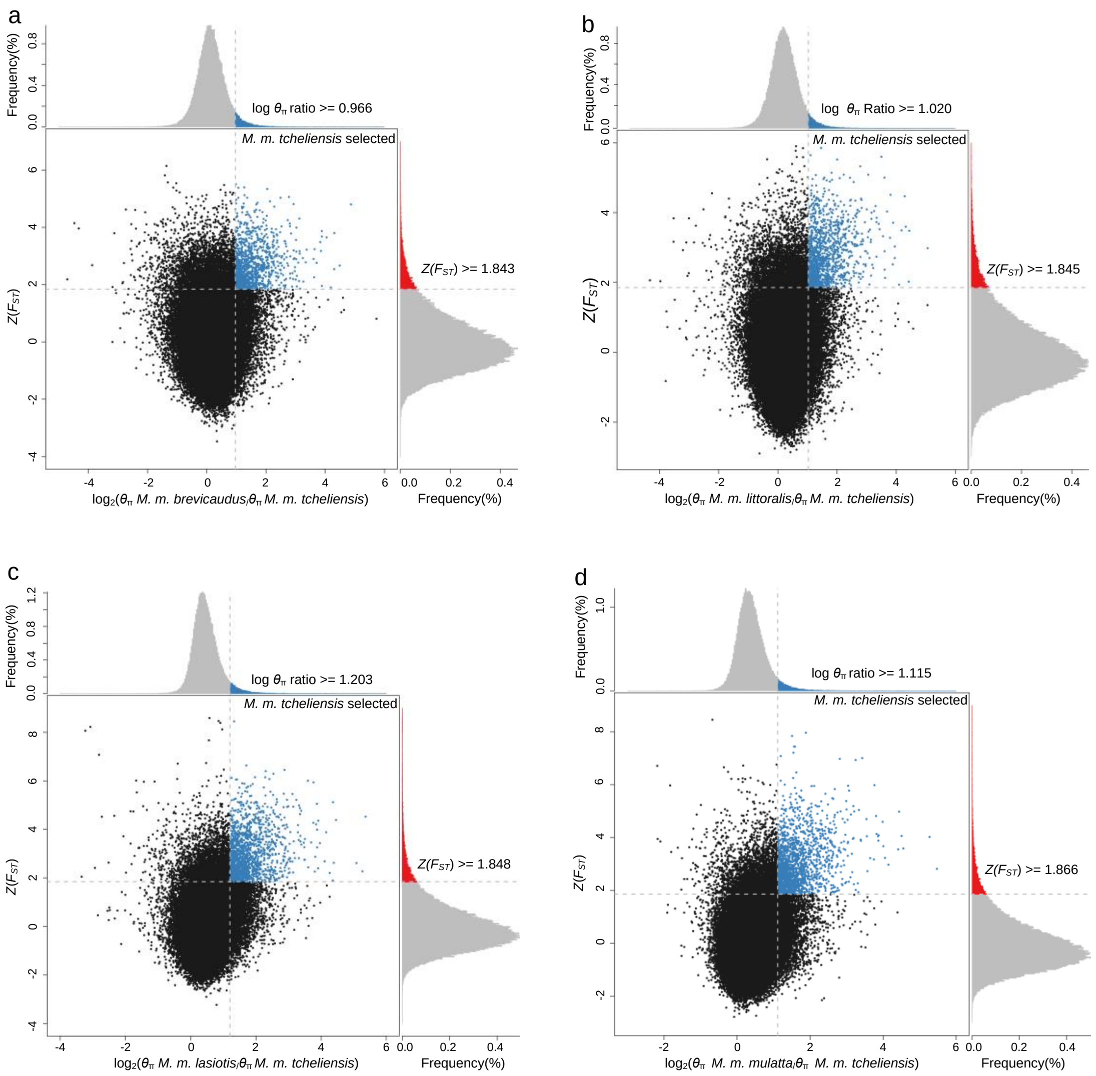

Supplementary Fig. 10. Distribution of Z (F_{ST}) and θ_π log ratio of 50kb windows with 25kb sliding window for *M. m. brevicaudus*. (a) comparison against *M. m. tcheliensis*, (b) comparison against *M. m. littoralis*, (c) comparison against *M. m. lasiotis*, (d) comparison against *M. m. mulatta*.

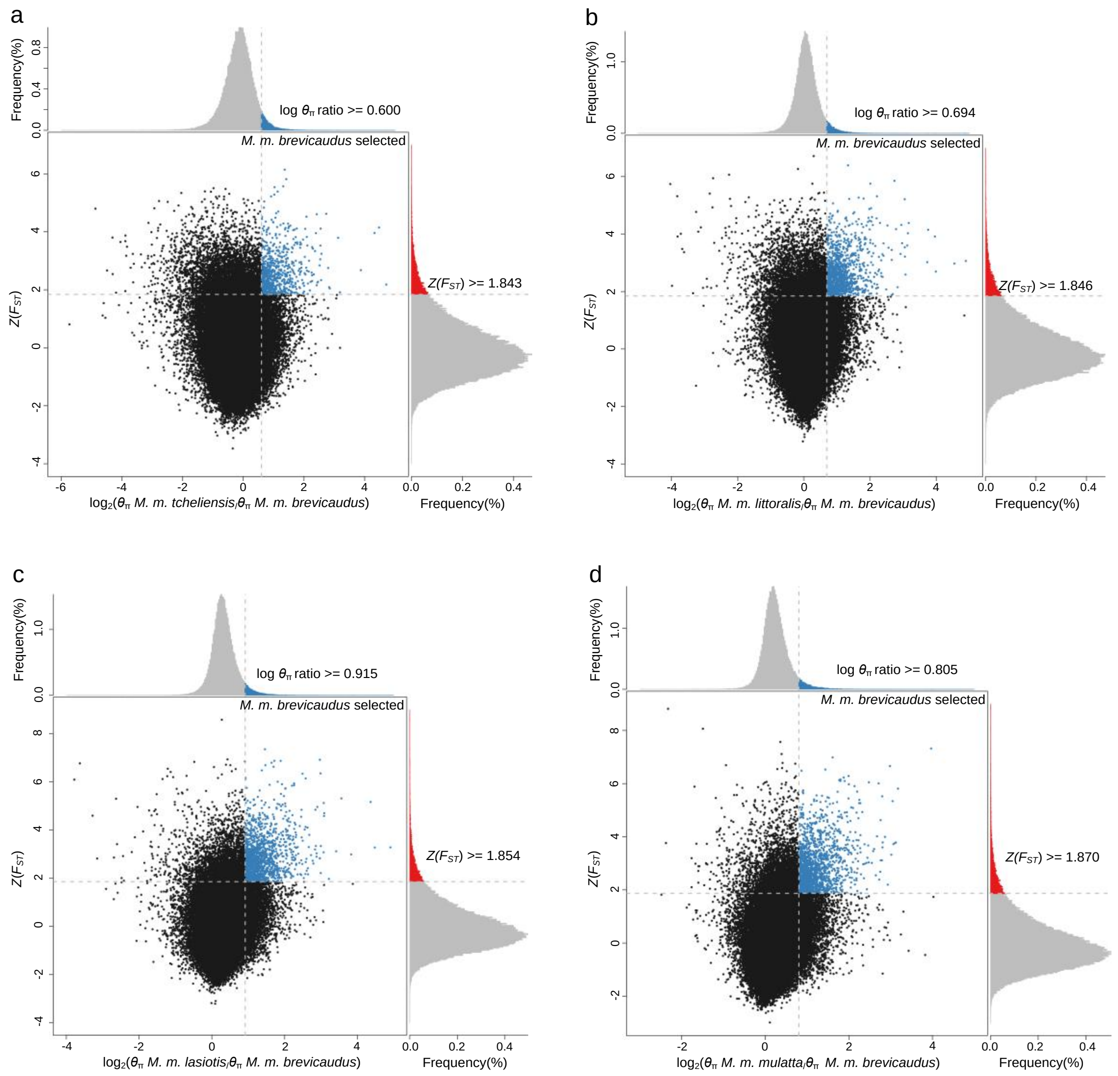

Supplementary Fig. 11. Distribution of Z (F_{ST}) and θ_π log ratio of 50kb windows with 25kb sliding window for *M. m. littoralis*. (a) comparison against *M. m. tcheliensis*, (b) comparison against *M. m. brevicaudus*, (c) comparison against *M. m. lasiotis*, (d) comparison against *M. m. mulatta*.

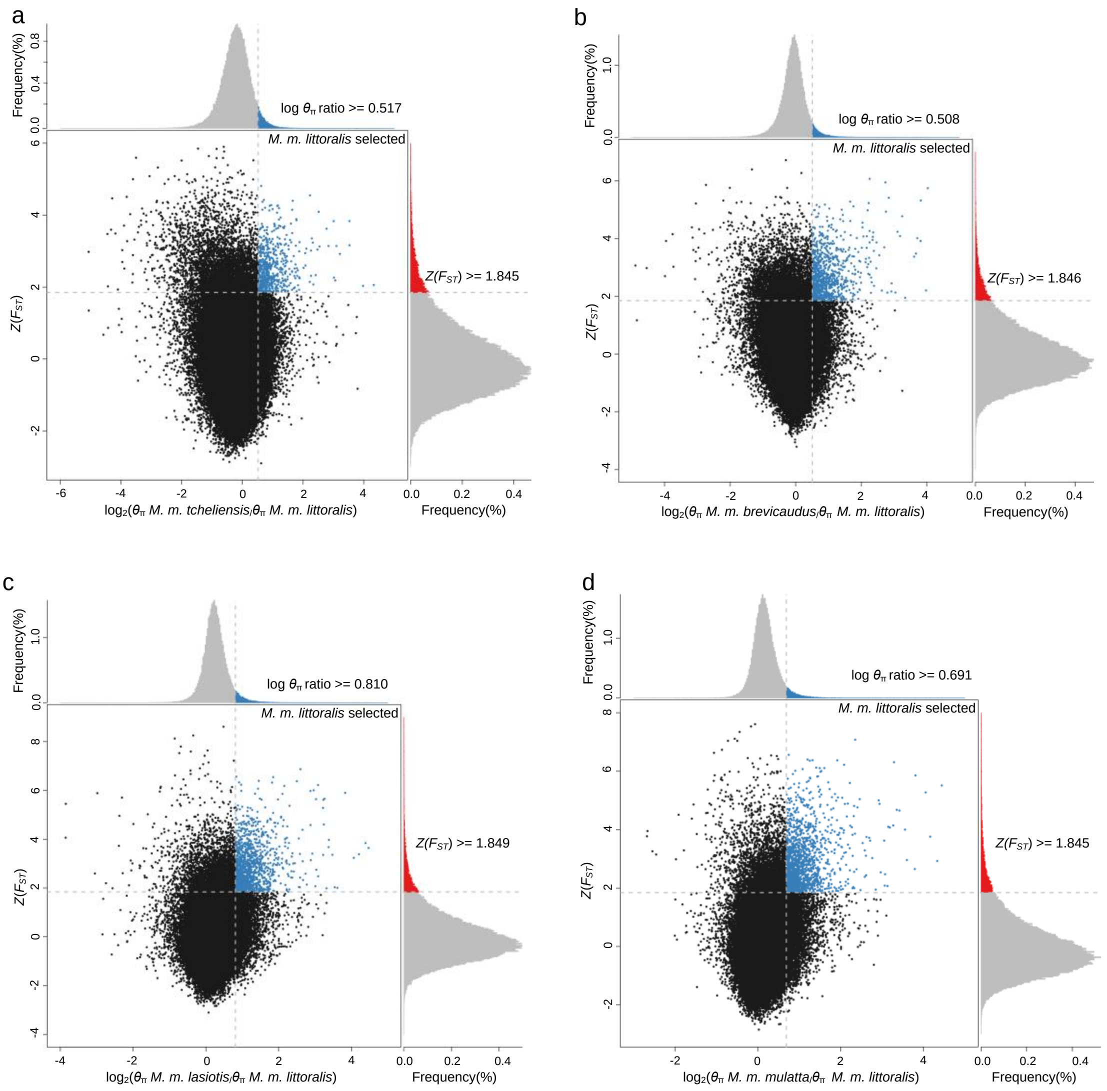

Supplementary Fig. 12. Distribution of Z (F_{ST}) and θ_π log ratio of 50kb windows with 25kb sliding window for *M. m. lasiotis*. (a) comparison against *M. m. tcheliensis*, (b) comparison against *M. m. brevicaudus*, (c) comparison against *M. m. littoralis*, (d) comparison against *M. m. mulatta*.

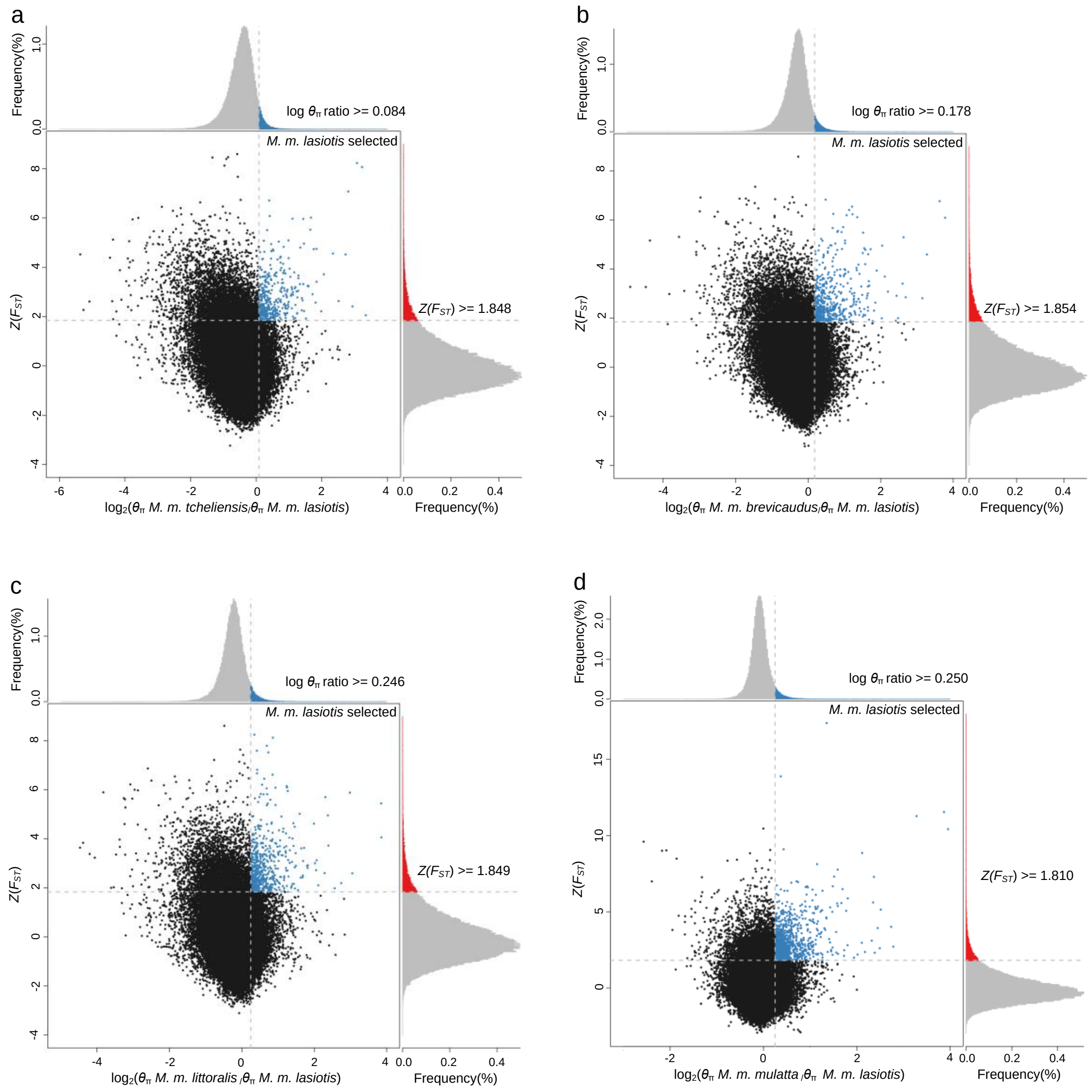

Supplementary Fig. 13. Distribution of Z (F_{ST}) and θ_π log ratio of 50kb windows with 25kb sliding window for *M. m. mulatta*. (a) comparison against *M. m. tcheliensis*, (b) comparison against *M. m. brevicaudus*, (c) comparison against *M. m. littoralis*, (d) comparison against *M. m. lasiotis*.

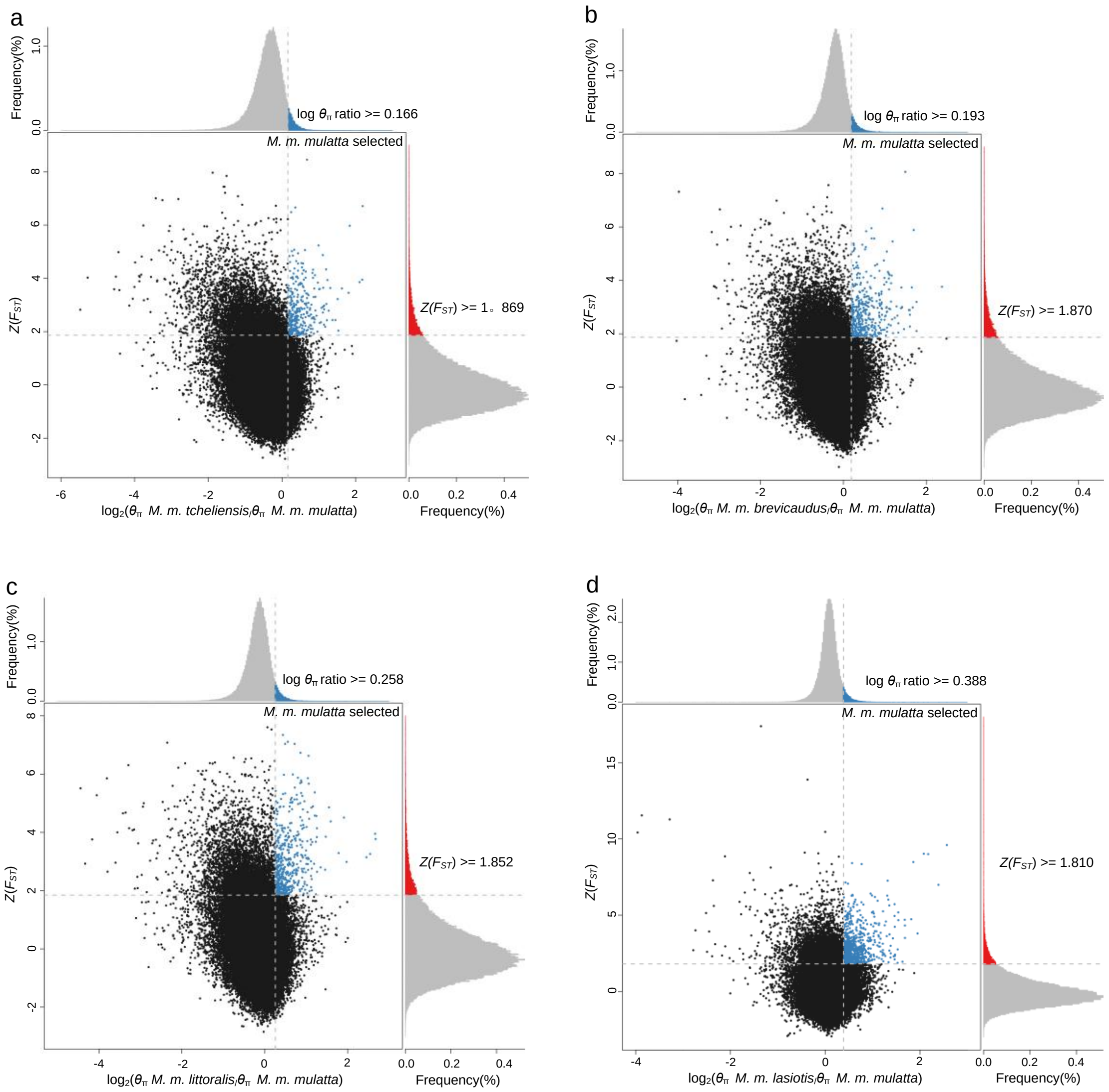

Supplementary Fig. 14. Non-synonymous variants in putatively selected genes. *Rpgripl1*, *Ext2*, *Fto* and *Atp6v0a4* are selected genes in *M. m. tcheliensis*. *Aggf1, Hspa4, Axin1* and *Ctnna3* are selected genes in *M. m. brevicaudus.*

RPGRIP1L

M. m. brevicaudus	D V F D A R A D G K G
M. m. brevicaudus	D V F D A R A D G K G
M. m. brevicaudus	D V F D A R A D G K G
M. m. brevicaudus	D V F D A R A D G K G
M. m. brevicaudus	D V F D A R A D G K G
M. m. tcheliensis	D V F D A Q A D G K G
M. m. tcheliensis	D V F D A Q A D G K G
M. m. tcheliensis	D V F D A Q A D G K G
M. m. tcheliensis	D V F D A Q A D G K G
M. m. tcheliensis	D V F D A Q A D G K G

EXT2

M. m. brevicaudus	R L G Q A I L S D V L
M. m. brevicaudus	R L G Q A I L S D V L
M. m. brevicaudus	R L G Q A I L S D V L
M. m. brevicaudus	R L G Q A I L S D V L
M. m. brevicaudus	R L G Q A I L S D V L
M. m. tcheliensis	R L G Q A M L S D V L
M. m. tcheliensis	R L G Q A M L S D V L
M. m. tcheliensis	R L G Q A M L S D V L
M. m. tcheliensis	R L G Q A M L S D V L
M. m. tcheliensis	R L G Q A M L S D V L

FTO

M. m. brevicaudus	K V S E C N S V E P Y
M. m. brevicaudus	K V S E C N S V E P Y
M. m. brevicaudus	K V S E C N S V E P Y
M. m. brevicaudus	K V S E C N S V E P Y
M. m. brevicaudus	K V S E C N S V E P Y
M. m. tcheliensis	K V S E C S S V E P Y
M. m. tcheliensis	K V S E C S S V E P Y
M. m. tcheliensis	K V S E C S S V E P Y
M. m. tcheliensis	K V S E C N S V E P Y
M. m. tcheliensis	K V S E C N S V E P Y

ATP6V0A4

M. m. brevicaudus	L R A S H R K S Q A S
M. m. brevicaudus	L R A S H R K S Q A S
M. m. brevicaudus	L R A S H R K S Q A S
M. m. brevicaudus	L R A S H R K S Q A S
M. m. brevicaudus	L R A S H R K S Q A S
M. m. tcheliensis	L R A S H Q K S Q A S
M. m. tcheliensis	L R A S H Q K S Q A S
M. m. tcheliensis	L R A S H Q K S Q A S
M. m. tcheliensis	L R A S H Q K S Q A S
M. m. tcheliensis	L R A S H R K S Q A S

AGGF1

M. m. brevicaudus	I E S P L Y E D I S N
M. m. brevicaudus	I E S P L Y E D I S N
M. m. brevicaudus	I E S P L Y E D I S N
M. m. brevicaudus	I E S P L Y E D I S N
M. m. brevicaudus	I E S P L H E D I S N
M. m. tcheliensis	I E S P L H E D I S N
M. m. tcheliensis	I E S P L H E D I S N
M. m. tcheliensis	I E S P L H E D I S N
M. m. tcheliensis	I E S P L H E D I S N
M. m. tcheliensis	I E S P L H E D I S N

HSPA4

M. m. brevicaudus	S I C S P V I S K P K
M. m. brevicaudus	S I C S P V I S K P K
M. m. brevicaudus	S I C S P V I S K P K
M. m. brevicaudus	S I C S P V I S K P K
M. m. brevicaudus	S I C S P I I S K P K
M. m. tcheliensis	S I C S P I I S K P K
M. m. tcheliensis	S I C S P I I S K P K
M. m. tcheliensis	S I C S P I I S K P K
M. m. tcheliensis	S I C S P I I S K P K
M. m. tcheliensis	S I C S P I I S K P K

CTNNA3

M. m. brevicaudus	H I V T G ... T S T V I
M. m. brevicaudus	H I V T G ... T S T V I
M. m. brevicaudus	H I V T G ... T S T V I
M. m. brevicaudus	H I V T G ... T S T V I
M. m. brevicaudus	H I V T G ... T S T V I
M. m. tcheliensis	H I I T G ... T S M V I
M. m. tcheliensis	H I I T G ... T S M V I
M. m. tcheliensis	H I I T G ... T S M V I
M. m. tcheliensis	H I V T G ... T S M V I
M. m. tcheliensis	H I V T G ... T S T V I

AXIN1

M. m. brevicaudus	S G T R K ... R P G A V
M. m. brevicaudus	S G T R K ... R P G A V
M. m. brevicaudus	S G T R K ... R P G A V
M. m. brevicaudus	S G T R K ... R P G A V
M. m. brevicaudus	S G T R K ... R P G A V
M. m. tcheliensis	S G I R K ... R P A A V
M. m. tcheliensis	S G I R K ... R P A A V
M. m. tcheliensis	S G I R K ... R P A A V
M. m. tcheliensis	S G T R K ... R P A A V
M. m. tcheliensis	S G T R K ... R P G A V

Supplementary Fig. 15. Linkage disequilibrium pattern of the five Chinese RM subspecies.

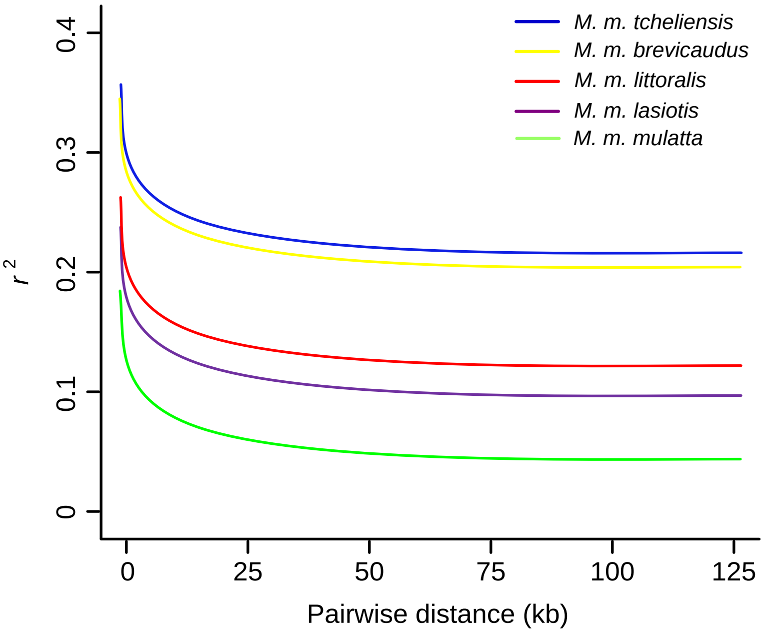

Supplementary Tables

Supplementary Table 1. Overview of sample information and sequencing statistics.

Location	Longitude and Latitude	Symbol	Sample ID	clean bases(Gb)	mapping rate	Effective Depth	Genome Coverage
Taihang Mountain, Henan Province	112°14'E, 35°04'N	TH	C_rhe_1	70.6	99.66%	22.49	97.00%
Taihang Mountain, Henan Province	112°14'E, 35°04'N	TH	C_rhe_2	22.7	99.64%	7.6	93.19%
Taihang Mountain, Henan Province	112°14'E, 35°04'N	TH	C_rhe_3	22.9	99.59%	7.63	94.56%
Taihang Mountain, Henan Province	112°14'E, 35°04'N	TH	C_rhe_4	29.8	99.66%	9.75	95.21%
Taihang Mountain, Henan Province	112°14'E, 35°04'N	TH	C_rhe_5	25.8	99.64%	8.52	94.29%
Huangshan Mountain, Anhui Province	118°01'E, 30°01'N	AH	C_rhe_6	40.4	99.63%	13.12	95.58%
Huangshan Mountain, Anhui Province	118°01'E, 30°01'N	AH	C_rhe_7	34.3	99.54%	10.78	95.84%
Huangshan Mountain, Anhui Province	118°01'E, 30°01'N	AH	C_rhe_8	28.3	99.49%	9.65	94.64%
Huangshan Mountain, Anhui Province	118°01'E, 30°01'N	AH	C_rhe_9	39.0	99.63%	11.92	95.02%
Huangshan Mountain, Anhui Province	118°01'E, 30°01'N	AH	C_rhe_10	35.4	99.63%	11.11	94.50%
Huangshan Mountain, Anhui Province	118°01'E, 30°01'N	AH	C_rhe_11	26.4	99.67%	8.3	94.05%
Nanping County, Fujian Province	117°00'E, 26°15'N	FJ	C_rhe_12	33.4	99.63%	10.5	95.03%
Nanping County, Fujian Province	117°00'E, 26°15'N	FJ	C_rhe_13	35.0	99.67%	10.63	95.88%
Nanping County, Fujian Province	117°00'E, 26°15'N	FJ	C_rhe_14	28.9	99.57%	9.41	95.07%
Nanping County, Fujian Province	117°00'E, 26°15'N	FJ	C_rhe_15	29.8	99.61%	9.64	95.01%
Nanping County, Fujian Province	117°00'E, 26°15'N	FJ	C_rhe_16	29.5	99.62%	9.43	94.92%
Daba Mountain, Hubei Province	110°03'E, 31°21'N	HB	C_rhe_17	33.8	99.65%	10.23	95.98%
Daba Mountain, Hubei Province	110°03'E, 31°21'N	HB	C_rhe_18	29.3	99.66%	8.64	95.34%
Daba Mountain, Hubei Province	110°03'E, 31°21'N	HB	C_rhe_19	31.7	99.62%	9.48	95.14%
Daba Mountain, Hubei Province	110°03'E, 31°21'N	HB	C_rhe_20	92.9	99.62%	28.93	94.75%
Daba Mountain, Hubei Province	110°03'E, 31°21'N	HB	C_rhe_21	111	99.63%	34.29	95.11%

Nanwan, Hainan Province	108°00'E, 27°00'N	HN	C_rhe_22	100.7	99.66%	30.55	95.17%
Nanwan, Hainan Province	108°00'E, 27°00'N	HN	C_rhe_23	29.4	99.65%	9.38	94.96%
Nanwan, Hainan Province	108°00'E, 27°00'N	HN	C_rhe_24	29.3	99.63%	9.3	94.34%
Nanwan, Hainan Province	108°00'E, 27°00'N	HN	C_rhe_25	29.8	99.61%	9.48	94.41%
Nanwan, Hainan Province	108°00'E, 27°00'N	HN	C_rhe_26	29.8	99.57%	9.6	94.82%
Longhu Mountain, Guangxi Province	107°21'E, 22°51'N	GX	C_rhe_27	31.2	99.65%	9.96	96.22%
Longhu Mountain, Guangxi Province	107°21'E, 22°51'N	GX	C_rhe_28	31.0	99.65%	10.2	94.52%
Longhu Mountain, Guangxi Province	107°21'E, 22°51'N	GX	C_rhe_29	31.5	99.64%	10.26	94.67%
Longhu Mountain, Guangxi Province	107°21'E, 22°51'N	GX	C_rhe_30	59.6	99.69%	20.2	95.21%
Longhu Mountain, Guangxi Province	107°21'E, 22°51'N	GX	C_rhe_31	37.1	99.67%	12.03	95.39%
Longhu Mountain, Guangxi Province	107°21'E, 22°51'N	GX	C_rhe_32	32.5	99.70%	10.61	95.07%
Qianling Mountain, Guizhou Province	106°29'E, 26°33'N	GZ	C_rhe_33	28.5	99.67%	9.52	92.35%
Qianling Mountain, Guizhou Province	106°29'E, 26°33'N	GZ	C_rhe_34	28.9	99.67%	9.56	92.86%
Qianling Mountain, Guizhou Province	106°29'E, 26°33'N	GZ	C_rhe_35	30.3	99.63%	9.89	92.91%
Qianling Mountain, Guizhou Province	106°29'E, 26°33'N	GZ	C_rhe_36	29.4	99.63%	9.17	92.59%
Qianling Mountain, Guizhou Province	106°29'E, 26°33'N	GZ	C_rhe_37	28.8	99.64%	9.17	92.31%
Qianling Mountain, Guizhou Province	106°29'E, 26°33'N	GZ	C_rhe_38	34.4	99.65%	11.1	92.13%
Aba County, Sichuan Province	101°18'E, 32°18'N	SC1	C_rhe_39	31.9	99.64%	9.85	95.52%
Aba County, Sichuan Province	101°18'E, 32°18'N	SC1	C_rhe_40	30.0	99.67%	9.91	90.97%
Aba County, Sichuan Province	101°18'E, 32°18'N	SC1	C_rhe_41	29.4	99.67%	9.39	91.52%
Aba County, Sichuan Province	101°18'E, 32°18'N	SC1	C_rhe_42	31.4	99.67%	10.48	91.54%
Aba County, Sichuan Province	101°18'E, 32°18'N	SC1	C_rhe_43	27.3	99.67%	8.36	93.15%
Aba County, Sichuan Province	101°18'E, 32°18'N	SC1	C_rhe_44	29.9	99.67%	10.13	90.29%
Aba County, Sichuan Province	101°18'E, 32°18'N	SC1	C_rhe_45	29.7	99.60%	9.67	94.67%
Beichuan County, Sichuan Province	103°44'E, 31°14'N	SC2	C_rhe_46	29.5	99.67%	9.83	93.03%

Beichuan County, Sichuan Province	103°44'E, 31°14'N	SC2	C_rhe_47	30.5	99.60%	10.18	92.72%
Beichuan County, Sichuan Province	103°44'E, 31°14'N	SC2	C_rhe_48	31.0	99.61%	10.45	92.30%
Beichuan County, Sichuan Province	103°44'E, 31°14'N	SC2	C_rhe_49	30.1	99.67%	9.99	92.86%
Beichuan County, Sichuan Province	103°44'E, 31°14'N	SC2	C_rhe_50	30.1	99.65%	9.94	93.02%
Ganzi County, Sichuan Province	97°22'E, 27°58'N	SC3	C_rhe_51	33.6	99.64%	9.45	95.94%
Ganzi County, Sichuan Province	97°22'E, 27°58'N	SC3	C_rhe_52	29.8	99.67%	9.82	92.40%
Ganzi County, Sichuan Province	97°22'E, 27°58'N	SC3	C_rhe_53	30.8	99.59%	9.89	91.53%
Ganzi County, Sichuan Province	97°22'E, 27°58'N	SC3	C_rhe_54	29.4	99.65%	9.07	92.45%
Ganzi County, Sichuan Province	97°22'E, 27°58'N	SC3	C_rhe_55	28.4	99.55%	8.91	91.83%
Ganzi County, Sichuan Province	97°22'E, 27°58'N	SC3	C_rhe_56	34.4	99.63%	11.62	91.90%
Hanyuan County, Sichuan Province	102°16'E, 29°05'N	SC4	C_rhe_57	28.2	99.61%	8.1	95.38%
Hanyuan County, Sichuan Province	102°16'E, 29°05'N	SC4	C_rhe_58	101.1	99.62%	30.75	90.59%
Hanyuan County, Sichuan Province	102°16'E, 29°05'N	SC4	C_rhe_59	28.2	99.64%	8.72	91.31%
Hanyuan County, Sichuan Province	102°16'E, 29°05'N	SC4	C_rhe_60	29.1	99.67%	9.53	90.97%
Hanyuan County, Sichuan Province	102°16'E, 29°05'N	SC4	C_rhe_61	29.9	99.63%	10.08	91.36%
Qinling Mountain, Shanxi Province	107°37'E, 33°57'N	SX	C_rhe_62	33.7	99.66%	10.99	94.98%
Qinling Mountain, Shanxi Province	107°37'E, 33°57'N	SX	C_rhe_63	34.1	99.65%	11.16	94.64%
Qinling Mountain, Shanxi Province	107°37'E, 33°57'N	SX	C_rhe_64	33.5	99.66%	10.91	94.98%
Qinling Mountain, Shanxi Province	107°37'E, 33°57'N	SX	C_rhe_65	38.8	99.66%	12.48	95.27%
Qinling Mountain, Shanxi Province	107°37'E, 33°57'N	SX	C_rhe_66	32.1	99.67%	10.26	94.75%
Qinling Mountain, Shanxi Province	107°37'E, 33°57'N	SX	C_rhe_67	38.7	99.67%	12.48	94.92%
Qinling Mountain, Shanxi Province	107°37'E, 33°57'N	SX	C_rhe_68	34.6	99.68%	11.09	94.79%
Qinling Mountain, Shanxi Province	107°37'E, 33°57'N	SX	C_rhe_69	30.5	99.63%	9.81	93.70%
Baoshan County, Yunnan Province	98°25'E, 24°08'N	YN1	C_rhe_70	30.6	99.67%	9.31	95.65%
Baoshan County, Yunnan Province	98°25'E, 24°08'N	YN1	C_rhe_71	30.2	99.67%	9.85	93.86%

Zhenyuan County, Yunnan Province	100°21'E, 23°24'N	YN2	C_rhe_72	101.7	99.67%	30.44	93.51%
Zhenyuan County, Yunnan Province	100°21'E, 23°24'N	YN2	C_rhe_73	30.6	99.68%	10.44	90.31%
Jinggu County, Yunnan Province	100°02'E, 22°49'N	YN3	C_rhe_74	70.2	99.65%	21.74	91.68%
Jinggu County, Yunnan Province	100°02'E, 22°49'N	YN3	C_rhe_75	31.3	99.56%	10.58	91.36%
Simao County, Yunnan Province	100°19'E, 22°27'N	YN4	C_rhe_76	29.7	99.66%	10.1	91.15%
Simao County, Yunnan Province	100°19'E, 22°27'N	YN4	C_rhe_77	29.2	99.68%	9.77	91.63%
Mojiang County, Yunnan Province	101°08'E, 22°51'N	YN5	C_rhe_78	30.1	99.67%	10.23	91.31%
Mojiang County, Yunnan Province	101°08'E, 22°51'N	YN5	C_rhe_79	85.8	99.58%	26.86	90.58%
Southwestern China	-	-	CR1	101	99.55%	34.32	96.25%
Sichuan Province	97°21'E, 26°03'N	SC	CR2	32.4	99.21%	10.04	97.03%

Supplementary Table 2. Distribution of autosomal SNPs within various genomic regions of RM.

**Variant type**	**SNP Count**	***M. m. littoralis***	***M. m. tcheliensis***	***M. m. brevicaudus***	***M. m. lasiotis***	***M. m. mulatta***
Total	52534348	35190639	14247374	15483720	34353777	26899284
Intergenic	33471626	22550975	9177392	9975853	22015530	17271353
Intronic	17938133	11902803	4769257	5189228	11619850	9070631
Upstream(1kb)	204882	136804	57309	60528	133743	104820
Downstream(1kb)	258562	172287	70822	75397	168095	131688
Upstream; Downstream	4247	2752	1109	1161	2700	2072
Splicing	1659	1072	438	453	1043	784
Non-synonymous	146135	90198	35540	37079	88253	65528
Synonymous	161466	106026	44023	46238	103635	80868
UTR3	285641	187547	75288	80823	182019	141466
UTR5	57798	37532	15143	15894	36313	28171
UTR5; UTR3	1104	707	297	334	713	534
Stop-gain	2783	1733	660	646	1675	1203
Stop-loss	311	203	95	86	207	166
Unknown	1	0	1	0	1	0

Supplementary Table 3. Identified SNPs and heterozygosity for 81 individuals from 17 sampling locations.

Location	Sample ID	Total SNP	Heterozygous SNP	Homozygous SNP	Heterozygosity
TH	C_rhe_1	8604308	4634141	3970167	0.001887
TH	C_rhe_2	6965015	3079508	3885507	0.001254
TH	C_rhe_3	7399391	3409275	3990116	0.001388
TH	C_rhe_4	7703607	3747481	3956126	0.001526
TH	C_rhe_5	7220064	3049001	4171063	0.001241
AH	C_rhe_6	8454885	5126823	3328062	0.002087
AH	C_rhe_7	8569933	4945610	3624323	0.002014
AH	C_rhe_8	8396058	4732478	3663580	0.001927
AH	C_rhe_9	8582394	4844177	3738217	0.001972
AH	C_rhe_10	8370707	4710144	3660563	0.001918
AH	C_rhe_11	8072982	4250293	3822689	0.001730
FJ	C_rhe_12	8114060	4382411	3731649	0.001784
FJ	C_rhe_13	8621659	4968245	3653414	0.002023
FJ	C_rhe_14	8429062	4683640	3745422	0.001907
FJ	C_rhe_15	8587793	4964925	3622868	0.002021
FJ	C_rhe_16	8452224	4758721	3693503	0.001938
HB	C_rhe_17	8740443	5240727	3499716	0.002134
HB	C_rhe_18	8422389	4856673	3565716	0.001977
HB	C_rhe_19	8353170	4807084	3546086	0.001957
HB	C_rhe_20	8479088	4902901	3576187	0.001996
HB	C_rhe_21	8632167	5134978	3497189	0.002091
HN	C_rhe_22	8376438	4460868	3915570	0.001816
HN	C_rhe_23	8280797	4309324	3971473	0.001755
HN	C_rhe_24	8136441	4098593	4037848	0.001669
HN	C_rhe_25	8350591	4518035	3832556	0.001840
HN	C_rhe_26	8536420	4947295	3589125	0.002014
GX	C_rhe_27	8635755	5283232	3352523	0.002151

GX	C_rhe_28	8339715	4649299	3690416	0.001893
GX	C_rhe_29	8289381	4635321	3654060	0.001887
GX	C_rhe_30	8768847	5358429	3410418	0.002182
GX	C_rhe_31	8722271	5183280	3538991	0.002110
GX	C_rhe_32	8455982	4682475	3773507	0.001906
GZ	C_rhe_33	8747929	5233094	3514835	0.002131
GZ	C_rhe_34	8824742	5390359	3434383	0.002195
GZ	C_rhe_35	8834164	5436772	3397392	0.002214
GZ	C_rhe_36	8323413	4700884	3622529	0.001914
GZ	C_rhe_37	8542092	4986712	3555380	0.002030
GZ	C_rhe_38	8671481	4996053	3675428	0.002034
SC1	C_rhe_39	8534202	4929967	3604235	0.002007
SC1	C_rhe_40	8480620	4853306	3627314	0.001976
SC1	C_rhe_41	8545434	4886168	3659266	0.001989
SC1	C_rhe_42	8421340	4697533	3723807	0.001913
SC1	C_rhe_43	8534725	4772641	3762084	0.001943
SC1	C_rhe_44	8349914	4681723	3668191	0.001906
SC1	C_rhe_45	8423688	4706730	3716958	0.001916
SC2	C_rhe_46	8561441	4895177	3666264	0.001993
SC2	C_rhe_47	8356498	4523554	3832944	0.001842
SC2	C_rhe_48	8701169	5189475	3511694	0.002113
SC2	C_rhe_49	8447782	4681171	3766611	0.001906
SC2	C_rhe_50	8495016	4743612	3751404	0.001931
SC3	C_rhe_51	8495663	4977298	3518365	0.002026
SC3	C_rhe_52	8743129	5319147	3423982	0.002166
SC3	C_rhe_53	8696887	5265132	3431755	0.002144
SC3	C_rhe_54	8335720	4786778	3548942	0.001949
SC3	C_rhe_55	8352688	4848790	3503898	0.001974
SC3	C_rhe_56	8698473	5308761	3389712	0.002161

SC4	C_rhe_57	8361063	4798087	3562976	0.001954
SC4	C_rhe_58	8533859	4941682	3592177	0.002012
SC4	C_rhe_59	8401200	4679782	3721418	0.001905
SC4	C_rhe_60	8524780	4964734	3560046	0.002021
SC4	C_rhe_61	8728250	5239720	3488530	0.002133
SX	C_rhe_62	8692788	5315071	3377717	0.002164
SX	C_rhe_63	8873239	5572226	3301013	0.002269
SX	C_rhe_64	8843283	5496303	3346980	0.002238
SX	C_rhe_65	8945908	5642782	3303126	0.002297
SX	C_rhe_66	8587353	5026905	3560448	0.002047
SX	C_rhe_67	9155134	5903093	3252041	0.002403
SX	C_rhe_68	8694125	5108777	3585348	0.002080
SX	C_rhe_69	8553814	4852649	3701165	0.001976
YN1	C_rhe_70	8630430	5462857	3167573	0.002224
YN1	C_rhe_71	8682636	5526581	3156055	0.002250
YN2	C_rhe_72	8951194	5713251	3237943	0.002326
YN2	C_rhe_73	8905896	5663643	3242253	0.002306
YN3	C_rhe_74	8922599	5670181	3252418	0.002309
YN3	C_rhe_75	8941855	5694887	3246968	0.002319
YN4	C_rhe_76	8928107	5671955	3256152	0.002309
YN4	C_rhe_77	8868221	5603921	3264300	0.002282
YN5	C_rhe_78	8925368	5657591	3267777	0.002303
YN5	C_rhe_79	8838445	5594057	3244388	0.002278
Southwestern China	CR1	8278458	4901797	3376661	0.001996
SC	CR2	7790708	4368908	3421800	0.001779

Supplementary Table 4. Tracy-Widom (*TW*) statistics and *P* values for the ten first eigenvalues in PCA. The significant *P* values are in bold.

**Number**	**Eigenvalues**	***TW***	***P***
1	7.240145	27.656	**4.78E-44**
2	5.689154	19.641	**4.21E-27**
3	5.293970	20.531	**7.72E-29**
4	4.758652	18.278	**1.62E-24**
5	4.497497	19.131	**4.00E-26**
6	4.194127	18.666	**3.04E-25**
7	3.893094	17.169	**1.75E-22**
8	3.514994	12.097	**4.98E-14**
9	3.424976	13.088	**1.45E-15**
10	3.337483	14.237	**2.03E-17**

Supplementary Table 5. Inferred demographic parameters with 95% confidence intervals for fastsimcoal2 model.

**Parameters**	**Prior distribution**	**Point estimate (95% CI)**
N_{A1}	logunif (1e3, 1e6)	9012 (1144, 248269)
N_{A2}	logunif (1e3, 1e6)	41536 (3420, 176483)
N_{A3}	logunif (1e3, 1e6)	132420 (41162, 602860)
N_{A4}	logunif (1e3, 1e6)	796292 (750910, 855957)
N_{mu}	logunif (1e3, 1e6)	20179 (7112, 33771)
N_{la}	logunif (1e3, 1e6)	32377 (16509, 53650)
N_{li}	logunif (1e3, 1e6)	7460 (1320, 10011)
N_{br}	logunif (1e3, 1e6)	8339 (5405, 18452)
N_{tc}	logunif (1e3, 1e6)	1223 (1078, 3556)
**T_1**	Uniform (500, 15000) *	4660 (651, 5034)
**T_2**	Uniform (500, 15000)	5605 (3962, 10468)
**T_3**	Uniform (1000, 15000)	9468 (4563, 14047)
**T_4**	Uniform (1000, 15000)	11432 (8363, 14737)
**m_** *mulatta_to_lasiotis*	logunif (1e-6, 1e-2)	3.63e-05 (2.39e-05, 2.50e-04)
**m_** *lasiotis__to_ mulatta*	logunif (1e-6, 1e-2)	5.33e-06 (2.38e-06, 2.51e-05)
**m_** *littoralis _to_ lasiotis*	logunif (1e-6, 1e-2)	8.36e-06 (4.59e-06, 1.38e-04)
**m_** *lasiotis _to_ littoralis*	logunif (1e-6, 1e-2)	1.05e-05 (2.19e-06, 3.93e-05)
**m_** *littoralis _to_ tcheliensis*	logunif (1e-6, 1e-2)	4.85e-04 (1.52e-04, 9.69e-04)
**m_** *tcheliensis _to_ littoralis*	logunif (1e-6, 1e-2)	1.80e-06 (1.57e-06, 2.24e-05)
**m_** *mulatta__to_ littoralis*	logunif (1e-6, 1e-2)	2.34e-05 (1.55e-05, 7.76e-05)
**m_** *littoralis _to_ mulatta*	logunif (1e-6, 1e-2)	6.91e-06 (4.75e-06, 9.34e-05)
**m_** *lasiotis _to_ tcheliensis*	logunif (1e-6, 1e-2)	6.88e-06 (2.28e-06, 2.95e-05)
**m_** *tcheliensis _to_ lasiotis*	logunif (1e-6, 1e-2)	2.43e-06 (1.29e-06, 5.12e-06)
**m_** *littoralis _to_ brevicaudus*	logunif (1e-6, 1e-2)	4.09e-05 (3.26e-05, 2.28e-04)
**m_** *brevicaudus__to_ littoralis*	logunif (1e-6, 1e-2)	1.96e-05 (9.00e-06, 4.87e-05)

Note: **N**, population size of haploids. The actual effective population sizes in Figure 2 and Supplementary Fig. 7 should be half of this value. (**A1** = ancestral population of *M. m. littoralis* and *M. m. tcheliensis,* **A2** = ancestral population of A1 and *M. m. brevicaudus,* **A3** = ancestral population of A2 and *M. m. lasiotis,* **A4** = ancestral population of all Chinese RMs, ***mu*** = population of *M. m. mulatta,* ***la*** = population of *M. m. lasiotis,* ***li*** = population of *M. m. littoralis,* ***br*** = population of *M. m. brevicaudus,* ***tc*** = population of *M. m. tcheliensis*) **T**, time since the population split in generations. These values multiplied by the generations time (g=11 years) are the actual divergence time in Figure 2 and Supplementary Fig. 7. (**1** = split among *M. m. littoralis* and *M. m. tcheliensis,* **2** = split between A1 and *M. m. brevicaudus,* **3**= split between A2 and *M. m. lasiotis,* **4**= split between A3 and *M. m. mulatta*); **m**, migration rates per generation between different subspecies).

*: We set the upper limit of divergence time between different subspecies to be 15000 generations due to the divergence between the ancestral lineages of Indian and Chinese RMs is ~ 162 kya, which is approximate 15,000 generations.

Supplementary Table 6. List of positively selected genes in the five Chinese RM subspecies.

**Subspecies**	**Gene number**	**Genes**
M. m. tcheliensis	176	ABCC9, ACCS, ACCSL, ACER3, ACTG2, ADAMTS2, ADAMTS7, ADARB1, AGMO, AKT3, AKTIP, ANKRD34B, AP1B1, AP4E1, ASH1L, ATP6V0A4, AZIN1, BEND7, BRAF, C10orf118, C14orf2, C18orf1, C1D, C1orf123, CAB39, CABLES1, CACNA2D3, CADM2, CAMKMT, CAPRIN1, CBY1, CCSER2, CD200, CDADC1, CDH13, CELF4, CHRM2, CHRNA9, CNTN3, CORIN, CPT2, CSMD3, CTBP2, CTNNA3, CTSH, DAB1, DCSTAMP, DLG2, DNAH6, DPYS, EBAG9, ELP2, EPDR1,EPHA6, ERICH1, ERP44, EXT2, EYS, FAM151B, FAM198A, FAM227A, FAR1, FBP1, FBP2, FBXO36, FER, FHOD3, FILIP1, FNDC7, FTO, GABRA2, GCKR, GMDS, GPM6A, GTF2F2, GTPBP4, HDGFRP3, HYOU1, IDI2, IL12RB2, INVS, JARID2, JOSD1, KIF18A, KLHL2, KTN1, LARP4B, LIMCH1, LRFN5, LRP12, MACROD2, MAGI1, MAGOH, MDGA2, METTL15, MORF4L1, MSH3, MYLIP, MYO6, NAT10, NEO1, NEURL1B, NKAIN2, NKTR, NME8, NOVA1, NPFFR2,NRXN1, OLIG3, OR4D1, OXCT1, PAPSS2, PCTP, PDE10A, PHF21A, PKIB, PLAT, PRKG1, PRPF38B, PRPSAP2, PXDNL, RAB28, RASA3, RBFOX1, RD3L, RFX7, RPGRIP1L, RPRD1A, RSRC1, SCN10A, SENP6, SEPSECS, SERBP1, SFRP4, SHOX2, SIM1, SLC26A9, SLC30A7, SLC37A4, SLC39A6, SLC5A10, SNTG1, SOCS5, SOD2, SOX5, SOX6, SP100, SPRR2E, SPZ1, ST6GALNAC3, STAMBP, SYBU, SYNDIG1, TCF20, TDRD9, TEX9, TM6SF1, TMEM100, TMEM213, TRAPPC4, USP47, VAV3, VDAC1, VKORC1L1, WDR36, WDR37, WDR7, WNT9B, XKR4, ZBTB47, ZFYVE16, ZNF354C, ZNF423, ZNF701, ZNF804A, ZNF83
M. m. littoralis	103	ADHFE1, ADIPOR1, ALKBH4, ANXA9, AP1S1, ARPC1A, ASTN2, ATP8A1, AUTS2, B3GALT6, BHMG1, C17orf85, C1QTNF12, C5, C7orf31, C8orf46, CAMKK1, CDKAL1, CERS2, CHN2, CLCC1, CNTNAP5, CPNE4, CUX1, CYTH3, DDC,DEDD2, DIRC1, DKK2, DYNLL2, EPO, ERF, ESYT2, ETNK2, FARS2, FBXO11, FN3K, FN3KRP, FOCAD, GARS, GNAZ, GPSM2, HSPB1, IMMP2L, LDLRAD3, LIPE, LPO, LRWD1, LYRM4, MINDY1, MNAT1, MPO, MYO9A, NAB1, NAT16, NEBL, NMNAT3, NPEPPS, NPR3, NUMB, ORAI2, OSBPL3, PDIA4, PHF21A, POLR2J, POP7, PPP1R9A, RAD51C, REC114, REN, RPL7A, RUNX1T1, RYK, SDF4, SERPINE1, SETDB1, SNX9, SOX13, SPTBN1, SRRM3, STK31, STKLD1, SURF1, SURF2, SURF4, TBCD, TBX5, THSD4, TMEM39B, TNFRSF18, TRAF1, TRIM5, TRIM56, TTLL10, UBE2J2, USP15, VGF, YWHAG, ZAN, ZBBX, ZNF526, ZNF559, ZZEF1
M. m. brevicaudus	148	AASDHPPT, ABCA10, ACVR2A, ADGRV1, AEBP2, AGAP2, AGGF1, ANGEL2, ARFGAP1, ARHGAP42, ARHGDIG, ASTN1, ASXL1, ATP8A2, ATRNL1, AXIN1, BIRC2, BIRC7, BNIPL, BSN, BTRC, C16orf5, C1orf56, CACNA2D1, CACNG7, CAMKMT, CAMKV, CAPS, CASC1, CASP4, CBLB, CD36, CDC42SE1, CEP170B, CHCHD3, CLEC4A, CLTC, CNTN5, CNTNAP5, COPG2, COX4I1, COX4NB, CRISPLD2, CTNNA3, DCC, DLG2, EIF2A, EMP1, EPHX1, ERO1A, FAM234A, FANCL, FERMT2, FGF14, FNDC11, GABPB2, GMEB2, GNPNAT1, GOLGA4, GPR137C, GRIA4, GRM7, GSE1, HELZ2, HMOX2, HSPA4, ID1,

		IGF1R, IGSF11, IQCM, KIF3B, MEI1, MMP13, MOK, MON1A, MSANTD4, MST1R, MYOZ1, NECAP1, NKAIN4, NPEPPS, NUP58, ORC4L, OS9, PDIA2, PDZRN3, PKD2L1, PLCL1, PPP3CB, PRKCG, PRPF6, PRUNE1, PTPN5, PTRH2, PXYLP1, PYROXD1, RAB11FIP4, RAB39A, RALGPS2, RANBP3, RAPGEF5, RGS11, RPS6KC1, SCAPER, SCD, SCMH1, SEC24C, SERP1, SIK3, SKA1, SLC18B1, SLC2A13, SLC35F2, SLC38A9, SLC4A4, SLCO1B1, SMURF1, SORD, SRP9, STK17A, STYX, SVEP1, SYNPO2, SYNPO2L, TCF4, TENM2, TMEM176A, TMEM176B, TMEM86A, TSGA13, TUBD1, TXNDC16, UBE2D4, UBE2L3, UCKL1, URGCP, VNN2, VWA8, YAP1, ZBED3, ZCCHC10, ZMYND8, ZNF248, ZNF274, ZNF346, ZNF512B, ZNF544, ZNF8
M. m. lasiotis	19	ASCC3, AUTS2, C11orf87, C5orf44, CCDC73, CENPK, DGKB, DLG2, ERICH3, PATJ, PPWD1, PSMB1, RABGAP1L, RALGPS2, SLC25A12, STXBP5L, TBP, TRIM23, TTC28,
M. m. mulatta	6	CFL2, DMAC2, ERICH4, FAF1, SEC24A, VPS13B,

Supplementary Table 7. Enrichment of genes under selective sweep in *M. m. tcheliensis*. Gray shade-coded enriched terms are pathways associated with gluconeogenesis.

Category	Term	Description	Modified Fisher Exact *P*-value	Count	Genes
GO_MF	GO:0045296	cadherin binding	5.24E-04	4	CDH13, NEO1, FER, CTNNA3
GO_MF	GO:0050839	cell adhesion molecule binding	2.86E-03	4	CDH13, NEO1, FER, CTNNA3
GO_MF	GO:0042132	fructose 1,6-bisphosphate 1-phosphatase activity	1.90E-02	2	FBP1, FBP2
GO_CC	GO:0044459	plasma membrane part	2.13E-02	30	CORIN, AP1B1, NEO1, IL12RB2, CHRNA9, RAB28, NPFFR2, RASA3, DLG2, EBAG9, SCN10A, GABRA2, MYO6, CTBP2, MAGI1, KIF18A, KTN1, NRXN1, CAPRIN1, CTNNA3, CDH13, ABCC9, EPHA6, CHRM2, LRP12, ASH1L, SLC26A9, SNTG1, ATP6V0A4, CD200
GO_BP	GO:0034637	cellular carbohydrate biosynthetic process	2.65E-02	4	GMDS, FBP1, FBP2, EXT2
GO_CC	GO:0044451	nucleoplasm part	2.93E-02	11	MORF4L1, ELP2, CTBP2, SP100, MYO6, RSRC1, MAGOH, GTF2F2, PHF21A, CBY1, C1D
GO_BP	GO:0019319	hexose biosynthetic process	3.36E-02	3	GMDS, FBP1, FBP2
GO_MF	GO:0046873	metal ion transmembrane transporter activity	3.78E-02	8	ABCC9, GPM6A, CHRNA9, SLC39A6, RASA3, CACNA2D3, SLC30A7, SCN10A
GO_MF	GO:0005262	calcium channel activity	3.98E-02	4	GPM6A, CHRNA9, RASA3, CACNA2D3
GO_BP	GO:0016358	dendrite development	4.19E-02	3	MYO6, TRAPPC4, PRKG1
GO_CC	GO:0045202	synapse	4.28E-02	8	PLAT, GABRA2, CTBP2, CHRNA9, CHRM2, TRAPPC4, NRXN1, DLG2
GO_BP	GO:0046364	monosaccharide biosynthetic process	4.64E-02	3	GMDS, FBP1, FBP2
GO_CC	GO:0070013	intracellular organelle lumen	4.82E-02	24	MORF4L1, GTPBP4, ELP2, CTBP2, SP100, ADARB1, MYO6, MAGOH, CBY1, SOD2, VDAC1, FAR1, ERP44, HYOU1, GCKR, OXCT1, RSRC1, LRP12, GTF2F2, PHF21A, NAT10, ZNF701, NOVA1, C1D
GO_BP	GO:0045934	negative regulation of nucleobase, nucleoside, nucleotide and nucleic acid metabolic process	4.97E-02	10	GTPBP4, CTBP2, SP100, MSH3, JARID2, PHF21A, CBY1, SOX6, C1D, ZNF423
KEGG	hsa00051	Fructose and mannose metabolism	4.35E-02	3	GMDS, FBP1, FBP2

Supplementary Table 8. Morphological differences between the five investigated Chinese RM subspecies.

Subspecies	*M. m. tcheliensis*	*M. m. littoralis*	*M. m. brevicaudus*	*M. m. mulatta*	*M. m. lasiotis*	references
Body size	big	big	small	small	middle	Jiang et al. 1991
Trunk length (mm)	403.8±13.9 (♂)<br>366.4±6.8 (♀)	388.0±19.6 (♂)<br>380.7±25.0 (♀)	336.0±2.5 (♂)<br>292.0±3.4 (♀)	-	-	Zhang et al. 2008<br>Zhang et al. 2016
Tail length (mm)	163.4±4.9 (♂)<br>141.4±3.5 (♀)	197.4±17.2 (♂)<br>176.4±18.1 (♀)	152.0±2.0 (♂)<br>147.0±2.7 (♀)	-	214.4±4.0 (♂)<br>198.9±2.1 (♀)	Zhang et al. 2008<br>Zhang et al. 2016
Body mass (kg)	8.8±3.0 (♂)<br>6.5±1.0 (♀)	8.64 ± 1.32 (♂)<br>7.16 ± 1.04 (♀)	6.3 ± 1.3 (♂)<br>5.5 ± 1.1 (♀)	6.7 ± 1.0 (♂)<br>5.3 ± 0.7 (♀)	8.0±1.1 (♂)<br>6.3±1.0 (♀)	Zhang et al. 2008<br>Zhang et al. 2016
chest circumference (mm)	424.5±16.8 (♂)<br>379.5±7.4 (♀)	-	-	-	347.0±5.0 (♂)<br>311.5±2.7 (♀)	Zhang et al. 2008<br>Zhang et al. 2016
head circumference (mm)	312.3±8.8 (♂)<br>286.2±2.8 (♀)	-	-	-	258.0±2.1 (♂)<br>246.6±1.2 (♀)	Zhang et al. 2008<br>Zhang et al. 2016
Shoulder back hair length (mm)	70-151	-	40-60	45-70	100-130	Zhao et al. 1997
Male total cranial length (mm)	-	133.6	113.0	119.6	127.1	Jiang et al. 1991
Coat color (shoulder and back)	gray	yellow and brown	rusty red	olive	olive	Jiang et al. 1991

Supplementary Table 9. Enrichment of the genes under selective sweep in *M. m. brevicaudus.* Gray shade-coded enriched terms are pathways associated with bone development.

Category	Term	Description	Modified Fisher Exact *P*-value	Count	Genes
GO_BP	GO:0008104	protein localization	3.79E-04	18	ARFGAP1, PDIA2, CLTC, OS9, RAB11FIP4, CBLB, COPG2, CD36, ID1, PPP3CB, RANBP3, NECAP1, SMURF1, SEC24C, AGAP2, SRP9, SERP1, MON1A
GO_BP	GO:0015031	protein transport	2.04E-03	15	ARFGAP1, CLTC, RAB11FIP4, CBLB, COPG2, CD36, RANBP3, PPP3CB, NECAP1, SMURF1, SEC24C, AGAP2, SRP9, SERP1, MON1A
GO_BP	GO:0045184	establishment of protein localization	2.22E-03	15	ARFGAP1, CLTC, RAB11FIP4, CBLB, COPG2, CD36, RANBP3, PPP3CB, NECAP1, SMURF1, SEC24C, AGAP2, SRP9, SERP1, MON1A
GO_CC	GO:0030120	vesicle coat	2.46E-03	4	COPG2, NECAP1, CLTC, SEC24C
GO_CC	GO:0030659	cytoplasmic vesicle membrane	3.11E-03	6	COPG2, CD36, NECAP1, GRIA4, CLTC, SEC24C
GO_CC	GO:0012506	vesicle membrane	4.43E-03	6	COPG2, CD36, NECAP1, GRIA4, CLTC, SEC24C
GO_BP	GO:0030509	BMP signaling pathway	4.65E-03	4	ACVR2A, ID1, ZNF8, SMURF1
GO_BP	GO:0008219	cell death	9.05E-03	13	DCC, BNIPL, CASP4, FGF14, PDIA2, BIRC7, PRKCG, STK17A, ZNF346, PTRH2, C16ORF5, BIRC2, AXIN1
GO_BP	GO:0016265	death	9.53E-03	13	DCC, BNIPL, CASP4, FGF14, PDIA2, BIRC7, PRKCG, STK17A, ZNF346, PTRH2, C16ORF5, BIRC2, AXIN1
GO_CC	GO:0048475	coated membrane	1.02E-02	4	COPG2, NECAP1, CLTC, SEC24C
GO_CC	GO:0030117	membrane coat	1.02E-02	4	COPG2, NECAP1, CLTC, SEC24C
GO_CC	GO:0044433	cytoplasmic vesicle part	1.07E-02	6	COPG2, CD36, NECAP1, GRIA4, CLTC, SEC24C
GO_CC	GO:0030662	coated vesicle membrane	1.52E-02	4	COPG2, NECAP1, CLTC, SEC24C
GO_BP	GO:0006915	apoptosis	1.73E-02	11	DCC, BNIPL, CASP4, PDIA2, BIRC7, STK17A, ZNF346, PTRH2, C16ORF5, BIRC2, AXIN1
GO_BP	GO:0012501	programmed cell death	1.90E-02	11	DCC, BNIPL, CASP4, PDIA2, BIRC7, STK17A, ZNF346, PTRH2, C16ORF5, BIRC2, AXIN1
GO_MF	GO:0005245	voltage-gated calcium channel activity	2.02E-02	3	CACNA2D1, CACNG7, GRM7
GO_BP	GO:0016044	membrane organization	2.75E-02	8	ARFGAP1, CD36, CDC42SE1, NECAP1, HSPA4, CLTC, SEC24C, SERP1

GO_MF	GO:0046872	metal ion binding	2.96E-02	43	CAPS, ARFGAP1, ZNF274, SORD, SLC38A9, SCAPER, ZNF346, ZMYND8, FANCL, HMOX2, PLCL1, AASDHPPT, ZNF512B, ZNF248, PPP3CB, SLC4A4, SEC24C, AGAP2, SIK3, AEBP2, ZCCHC10, CACNA2D1, SVEP1, GMEB2, CACNG7, SCD, ZNF544, ASXL1, BIRC7, ZNF8, BSN, PRKCG, PDZRN3, NPEPPS, MMP13, BIRC2, PKD2L1, RAB11FIP4, ACVR2A, CBLB, GRM7, ZBED3, ATP8A2
GO_MF	GO:0008270	zinc ion binding	3.22E-02	27	ARFGAP1, ZNF274, SORD, SCAPER, ZNF346, ZMYND8, FANCL, ZNF512B, ZNF248, PPP3CB, SEC24C, AGAP2, AEBP2, ZCCHC10, GMEB2, ZNF544, ASXL1, BIRC7, ZNF8, BSN, PRKCG, PDZRN3, NPEPPS, BIRC2, MMP13, CBLB, ZBED3
GO_MF	GO:0043169	cation binding	3.44E-02	43	CAPS, ARFGAP1, ZNF274, SORD, SLC38A9, SCAPER, ZNF346, ZMYND8, FANCL, HMOX2, PLCL1, AASDHPPT, ZNF512B, ZNF248, PPP3CB, SLC4A4, SEC24C, AGAP2, SIK3, AEBP2, ZCCHC10, CACNA2D1, SVEP1, GMEB2, CACNG7, SCD, ZNF544, ASXL1, BIRC7, ZNF8, BSN, PRKCG, PDZRN3, NPEPPS, MMP13, BIRC2, PKD2L1, RAB11FIP4, ACVR2A, CBLB, GRM7, ZBED3, ATP8A2
GO_CC	GO:0031410	cytoplasmic vesicle	3.88E-02	10	CAMKV, ARHGDIG, COPG2, CD36, CRISPLD2, NECAP1, GRIA4, CLTC, SEC24C, AXIN1
GO_BP	GO:0034613	cellular protein localization	3.90E-02	8	COPG2, CBLB, ID1, PPP3CB, SMURF1, CLTC, SEC24C, SRP9
GO_BP	GO:0070727	cellular macromolecule localization	4.03E-02	8	COPG2, CBLB, ID1, PPP3CB, SMURF1, CLTC, SEC24C, SRP9
GO_BP	GO:0031396	regulation of protein ubiquitination	4.18E-02	4	BTRC, PRKCG, SMURF1, AXIN1
GO_CC	GO:0016023	cytoplasmic membrane-bounded vesicle	4.19E-02	9	ARHGDIG, COPG2, CD36, CRISPLD2, NECAP1, GRIA4, CLTC, SEC24C, AXIN1
GO_MF	GO:0043167	ion binding	4.33E-02	43	CAPS, ARFGAP1, ZNF274, SORD, SLC38A9, SCAPER, ZNF346, ZMYND8, FANCL, HMOX2, PLCL1, AASDHPPT, ZNF512B, ZNF248, PPP3CB, SLC4A4,

					SEC24C, AGAP2, SIK3, AEBP2, ZCCHC10, CACNA2D1, SVEP1, GMEB2, CACNG7, SCD, ZNF544, ASXL1, BIRC7, ZNF8, BSN, PRKCG, PDZRN3, NPEPPS, MMP13, BIRC2, PKD2L1, RAB11FIP4, ACVR2A, CBLB, GRM7, ZBED3, ATP8A2
GO_BP	GO:0007178	transmembrane receptor protein serine/threonine kinase signaling pathway	4.49E-02	4	ACVR2A, ID1, ZNF8, SMURF1
GO_MF	GO:0070411	I-SMAD binding	4.62E-02	2	SMURF1, AXIN1
GO_CC	GO:0031982	vesicle	4.86E-02	10	CAMKV, ARHGDIG, COPG2, CD36, CRISPLD2, NECAP1, GRIA4, CLTC, SEC24C, AXIN1
GO_CC	GO:0031988	membrane-bounded vesicle	4.91E-02	9	ARHGDIG, COPG2, CD36, CRISPLD2, NECAP1, GRIA4, CLTC, SEC24C, AXIN1
KEGG	hsa04120	Ubiquitin mediated proteolysis	5.38E-04	7	FANCL, UBE2D4, CBLB, BTRC, SMURF1, UBE2L3, BIRC2
KEGG	hsa04144	Endocytosis	2.49E-03	7	ARFGAP1, RAB11FIP4, IGF1R, CBLB, SMURF1, CLTC, AGAP2
KEGG	hsa05200	Pathways in cancer	1.12E-02	8	DCC, IGF1R, CBLB, FGF14, PRKCG, BIRC2, CTNNA3, AXIN1

Supplementary Table 10. Distribution of SNPs in the selected genes described in the part of "Signatures of selection and local adaptation".

Subspecies	Gene	Total	Intronic	Down	Up	UTR3	UTR5	S	N
M. m. tcheliensis	Fbp1	120	112	7	0	1	0	0	0
	Fbp2	50	50	0	0	0	0	0	0
	Ext2	276	266	2	5	1	0	0	1
	Sox5	683	677	1	2	3	0	0	0
	Sox6	445	434	0	0	1	8	0	1
	Atp6v0a4	202	187	2	1	2	0	4	1
	Fto	623	620	0	1	0	0	0	1
	Rpgrip1l	61	56	2	1	0	0	0	1
M. m. brevicaudus	Aggf1	225	214	0	3	2	0	2	1
	Axin1	181	153	0	3	4	0	8	2
	Hspa4	54	48	1	2	1	0	0	1
	Ctnna3	2898	2884	3	2	1	0	2	2

Note: Down = the downstream 1kb region of gene;
Up = the upstream 1kb region of the gene;
UTR3 = 3'-untranslated region;
URT5 = 5'-untranslated region;
S = Synonymous;
N = Nonsynonymous.

Supplementary Table 11. Non-synonymous SNPs with significant differences at the 5% level in the distributions of genotypes between *M. m. tcheliensis* and *M. m. brevicaudus*. The SNP names were combined with chromosome and physical position. The annotation information is based on the gff3 form Ensembl.

Subspecies	Gene	SNP name	Case, Control frequencies	Chi-square	*P* value	Annotation
M. m. tcheliensis	Atp6v0a4	3:165371351	0.600, 0.000	8.571	0.0034	R667Q
	Ext2	14:21923519	0.900, 0.000	16.364	0.0001	I363M
	Fto	20:37946505	0.400, 0.000	5	0.0253	N10S
	Rpgrip1l	20:37634331	0.900, 0.000	16.364	0.0001	R1281Q
M. m. brevicaudus	Aggf1	6:73720067	0.500, 0.000	6.667	0.0098	H343Y
	Hspa4	6:130796077	0.600, 0.000	8.571	0.0034	I782V
	Ctnna3	9:66267701	1.000, 0.600	5	0.0253	V551I
	Ctnna3	9:66267780	1.000, 0.500	6.667	0.0098	T577M
	Axin1	20:279484	1.000, 0.500	6.667	0.0098	A674G
	Axin1	20:279538	1.000, 0.600	5	0.0253	T656I

Supplementary Table 12. List of RM variants scored by HGMD and ClinVar as “disease causing” or “pathogenic”.

Please refer to Excel file Supplemental Data 1.xlsx.

Supplementary Table 13. Population study for *Ncoa3* reveals multiple genotypes.

Please refer to Excel file Supplemental Data 2.xlsx.
