# Supplementary material for: Population genomics of wild Chinese rhesus macaques reveals a dynamic demographic history and local adaptation, with implications for biomedical research
Source: Gigascience. 2018 Aug 27;7(9):giy106. doi: 10.1093/gigascience/giy106 (PMC6143732; doi:10.1093/gigascience/giy106)
Supplement: Response_to_Reviewer_Comments_Original_Submission.pdf [file giy106_response_to_reviewer_comments_original_submission.pdf]

Author's Response To Reviewer Comments

Close

Dear Dr. Hans Zauner,

Thank you for your consideration and encouragement of our submission to GigaScience. In light of the reviewers' constructive comments, we have revised the manuscript and would like to re-submit it to GigaScience. Generally, we have re-done the analyses based on the updated genome reference of rhesus macaque (Mmul_8.0.1) and re-designed the models for demographic model testing. The point-by point response to the comments are below:

Reviewer #1:

Comment 1-1:
The rhemac2 reference genome assembly is an old genome reference sequence. There are now more recent, higher quality reference assemblies for this species. However, I do not think that the use of rhemac2 is necessarily a major problem for the population phylogenetics and demographic analyses. SNPs identified using rhemac2 should be very similar (though not identical) to the SNP calls that would be obtained using the more recent assemblies. And it should not be a problem that the rhemac2 assembly is built from an Indian-origin animal. It would have been better (more comprehensive and less susceptible to errors) for the authors to use a more recent reference genome, but using rhemac2 for the evolutionary and demographic analyses does not seem to me to be a major concern.
Response 1-1:
We really appreciated this suggestion. We have performed SNP calling using the new genome reference of rhesus macaque Mmul_8.0.1 (line 101 and 378). The population structure, phylogenetic and demographic analyses were also carried out with the new dataset. As predicted by the reviewer, the new results are very similar to the former results using rheMac2 (Figure 1, Supplementary Fig. 4 and 5, Supplementary Table 4).

Comment 1-2:
The second aspect of the paper is an analysis of functional genetic variation. The authors used FST and other population statistics to identify regions of the macaque genome that show significant differentiation among populations, focusing particularly on the most northern and most southern populations. These analyses suggest that there has been selection for differences in skeletal development and cardiovascular physiology that distinguish Chinese rhesus subspecies (selective sweeps). I do have some concerns about these analyses.
a) First and most importantly, this is where the use of rhemac2 as the reference assembly seems to me to be somewhat problematic. The rhemac2 assembly contains some assembly errors. But more relevant to this manuscript, it was annotated by NCBI and Ensembl before there was substantial RNA sequence data to assist in gene prediction. Investigators who have used rhemac2 for functional studies of protein-coding genes have found errors in some of the gene models, likely due to the lack of access to good RNA sequence data at the time of the annotation. The newer reference genomes for rhesus macaque (e.g. Mmul_8.0.1) have also been annotated by NCBI and Ensembl. These newer annotations are more complete and more accurate because there is now more RNA sequence data available to support gene models and to identify true exon-intron boundaries. I would be concerned that some of the conclusions Liu et al. have generated regarding selection on specific genes may be

problematic due to potential problems with rhemac2 gene annotations. Even though the analyses depend on FST and related statistics 9 (and not dN/dS ratios), I assume that the authors did examine the coding sequence differences among Chinese rhesus populations for the genes that they infer were under selection. I recommend that the authors (at a minimum) re-check their analyses and conclusions regarding positive selection on specific genes, using the more accurate, better annotated reference assemblies that were produced more recently than rhemac2.

Response 1-2:

In this revised manuscript, all analyses were carried out based on the genome reference and the annotation of Mmul_8.0.1. Most of the previously observed selection signals were confirmed, but also new findings were obtained.

Among the 176 genes found to be under positive selection in M. m. tcheliensis, two (Fbp1, Fbp2, modified Fisher Exact P=1.90E-02; Fig. 3c, d; Supplementary Table 7) are enriched in the gene ontology (GO) term "fructose 1, 6-bisphosphate 1-phosphatase activity". These two genes encode for fructose-1, 6-bisphosphatase 1 and fructose-1, 6-bisphosphatase isozyme 2 which catalyze the hydrolysis of fructose 1, 6-bisphosphate and play a rate-limiting role in gluconeogenesis. Furthermore, in starved zebrafish it was shown that the expression of Fbp1 was significantly unregulated in brain and liver tissues. Our findings suggest that the regulation of gluconeogenesis might be a mechanism of M. m. tcheliensis to adapt to food shortage in winter. (line 222-236)

Additionally, we have found 127 putatively selected genes in M. m. brevicaudus, four of which were enriched in GO term "Bone morphogenetic protein (BMP) signaling pathway" (modified Fisher Exact P=4.65E-02) and two genes were enriched in GO term "I-SMAD binding (P=4.65E-02)". These genes under selection might have contributed to smaller body size of M. m. brevicaudus and adaptation to hot climate. (line 257-265)

Comment 1-3:

b) It is not clear from this version of the manuscript (lines 207-219) whether Liu et al. observed any non-synonymous variants in the genes they identified as showing evidence of selective sweeps. Were there non-synonymous differences in the alleles found in the different Chinese rhesus populations, or were all the FST values based on intronic and/or intervening SNPs between genes? The case for positive selection on PAPSS2, SOX5 and other genes would be stronger if the authors identified non-synonymous or other coding variants that are predicted to influence protein function. If there are no non-synonymous differences observed between populations, then Liu et al. would (I suppose) have to argue that the selection was on non-coding regulatory variants. No specific statement about how the proposed selection is suggested to have influenced these genes is presented in the manuscript. Readers should be informed as to what particular variants distinguish the alleles in M. m. tcheliensis from M. m. brevicaudus, etc., and why the authors believe the observed sequence differences constitute true functional differences.

Response 1-3:

This is a very good point. Both coding and non-coding changes could contribute to local adaptations of organisms. To further investigate the adaptive mechanism of M. m. tcheliensis and M. m. brevicaudus to the opposite climates (cold versus hot), we focused on SNPs in the gene regions of above described candidate genes. A total of 5817 SNPs were found with significant differences at the 5% level in the distributions of genotypes between these two subspecies, and 10 SNPs were non-synonymous variants (Supplementary table 10 and 11). In M. m. tcheliensis, non-synonymous mutations were found in the coding regions of Atp6v0a4 (R667Q), Ext2 (I363M), Fto (N10S) and Rpgrip1l (R1281Q) (Supplementary table 11 and Supplementary Fig. 13), implying that selection might has acted on protein

sequence changes. No non-synonymous changes were detected in Fbp1, Fbp2, Sox5 and Sox6. However, SNPs are located in the 1kb up/downstream, 5' and 3' UTR, and intronic regions of these genes (Supplementary table 10), indicating selection on non-coding regulatory variants. Correspondingly, non-synonymous mutations in Aggf1 (H343Y), Axin1 (A674G, T656I), Hspa4 (I782V) and Ctnna3 (V551I, T577M) were revealed for M. m. brevicaudus (Supplementary table 11 and Supplementary Fig. 13) (line 278-291).

Comment 1-4:
c) It is not stated (lines 220-232) whether the GO terms related to heart development, heart rate or temperature response are statistically significantly enriched in this analysis. The authors should provide the same type of statistical evidence for these GO term results that they do for the limb morphogenesis results above.
Response 1-4:
We have found three putatively selected genes related to GO terms of "blood vessel morphogenesis", "regulation of heart rate by cardiac conduction" and "response to temperature stimulus". However, these GO terms are not significantly enriched (line 266-271).

Comment 1-5:
The new results presented in this paper regarding phylogenetic relationships among populations, and the history of population differentiation and effective size change, are important findings and make a valuable contribution to the literature.
Response 1-5:
Thank you for such an evaluation.

Other minor issues:
Comment 1-6:
Line 79: I think there may be a typo here. I do not think the authors intend to state that the effective population size of Indian rhesus macaques is only 17,000. This should be checked again.
Response 1-6:
It is not a typo here. The study on demographic history of Chinese and Indian RMs by Hernandez et al. (2007) revealed effective population sizes of ~ 17,014 and 239,704 for Indian and Chinese populations, respectively.

Comment 1-7:
Lines 137-148: It might be useful to compare the results for population size change over time that Liu et al. obtain here to those of previous population genetic analyses of rhesus macaques (e.g. Xue et al. 2016 and Hernandez et al. 2007).
Response 1-7:
We really appreciated this suggestion. Interestingly, the demographic inference by Xue et al. 2016 of the genomic data for one Chinese RM (CH_37945) from AH (M. m. littoralis) qualitatively resembled the demographic trajectory of M. m. littoralis herein presented (line 156-158).
Hernandez et al. reported that Chinese RM population has experienced 3.3-fold growth. We have checked the sample information of nine Chinese RMs included in Hernandez et al. 2007. Seven of the Chinese animals were sampled from Suzhou (eastern China), one from Kunming (western China), and one from Guandong (eastern China), which means eight individuals of M. m. littoralis and one of M. m. mulatta. Coincidently, a population expansion of M. m. littoralis (from NA1 =2.0k to Nli = 24.6k; Supplementary Table 5) since

44.8 kya was also detected in our results (line 175-178). However, since the RMs studied in Hernandez et al. 2007 were captive-born, although with wild-caught parents, different populations have been mixed. Wild-caught RMs are often transferred from one breeding center to another. Thus we think a comparison between captive- and wild-born RMs perhaps is inappropriate. So we do not address this point in the manuscript.

Comment 1-8:
Lines 145-148: How do the authors reconcile the different estimates for effective population size at about 60-80,000 years ago for M. m. tcheliensis, M. m. littoralis and M. m. brevicaudus that were obtained by the PSMC analysis versus the fastsimcoal2 analysis? Do the authors favor one of these over the other? Is there possibly a way to reconcile these different results?
Response 1-8:
The fastsimcoal2 analysis revealed a bottleneck in population size (NA1 =2.0k and NA2 =1.5k) of pan-eastern RMs (M. m. tcheliensis, M. m. littoralis and M. m. brevicaudus) during the period from 111.9 kya to 45.0 kya (Fig. 2b), which coincided with the population decline of pan-eastern RMs since approximately 100 kya as revealed by PSMC analyses. However, the population growth that occurred in pan-eastern RMs after the bottleneck has not been detected by PSMC analyses, given that PSMC is less accurate when reconstructing recent histories within ~100 kya (line 175-180). We prefer the PSMC analysis to reconstruct the historical demography older than 100 kya, and prefer fastsimcoal2 to model more recent demographic fluctuations.

Reviewer #2:

Comment 2-1:
In the first part, the authors reconstruct the phylogeny of Chinese rhesus macaques based on a whole-genome neighbor-joining tree. This is a rather crude type of phylogenomic analysis and doesn't allow to draw conclusions about the evolutionary history as done on lines 109-114. Here, the paper would benefit a lot from applying proper species tree methods that take incomplete lineage sorting into account. This will provide a reliable picture about the phylogenetic relationships of the five subspecies that can then act as a useful starting point to design a set of demographic models to test in the next step.
Response 2-1:
Many thanks for this helpful comment. To reveal the phylogenetic relationships among the five Chinese RM subspecies, we now employed the SVDquartets approach that takes incomplete lineage sorting into account (line 159-160, 412-414). The obtained phylogenetic tree suggests a “step-by-step” divergence for five Chinese RM subspecies. Accordingly, the M. m. mulatta lineage diverged form that of the remaining Chinese RMs firstly and then the M. m. lasiotis diverged from the ancestral lineage of pan-eastern RMs (M. m. tcheliensis, M. m. littoralis and M. m. brevicaudus). Subsequently, M. m. brevicaudus diverged from the ancestor of M. m. tcheliensis and M. m. littoralis, the divergence of which occurred lastly (Supplementary Fig. 6) (line 161-166). We used this pattern as starting point to design testable demographic models (see below).

Comment 2-2:
My main concern deals with the design of the models for demographic model testing. Here, the paper lacks critical details to understand the reasoning behind the selection of the 8 compared models. It's completely unclear how these models have been chosen from the total

number of possible (sub)species tree configurations and how they were parameterized. Supplementary Table 5 shows that the number of parameters in these 8 models range from 6 to 12, but they seem to do so in a very unintuitive way. For example, in Supplementary Figure 6 it seems that model 2 is a simplified version of model 8 with one less divergence time parameter. But Supplementary Table 5 shows that model 2 has actually 3 parameters more than model 8. Moreover, for parameter estimation, the authors expanded the selected model 2 by additional parameters without specifying which of the parameters listed in Supplementary Table 6 have already been part of the model selection. Comparing oversimplified models might lead to the selection of a suboptimal model in the first step. It's therefore absolutely crucial that the authors provide a detailed table showing the parameterization of all tested models (including parameter bounds) and explain in detail the reasoning behind the selection and design of these models. The type and parameterization of models has a strong impact on the outcome of such model testing approaches and without this critical information, it's impossible to assess how robust the findings of this analysis actually are. Additionally, the authors should provide a measure of the goodness of fit of the selected scenario to show that this model can reasonably well explain the observed data.
Response 2-2:
The SVDquartets approach (see above) revealed only one divergence scenario. Under this "step-by-step" divergence scenario, we performed the joint site frequency spectrum (SFS) approach implemented in fastsimcoal2 to model demographic fluctuations, respective divergence times and gene flow events among the five RM subspecies. In Supplementary Table 5 the full results are provided. (line 159-181)

Comment 2-3:
In the positive selection analysis, the authors calculate genetic diversity (theta pi) based on their set of variable sites only. This approach is flawed, as it doesn't allow to distinguish between non-variable sites and sites that are not sufficiently covered for reliable genotyping in the sequenced individuals. It is therefore important that the authors take coverage information for every site in the genome into account in order to obtain reliable estimates of window-wise genetic diversity.
Response 2-3:
In the revised manuscript, we performed SNP calling again following GATK's best practice based on the single-sample calling plus joint genotyping workflow. For a variant which is not callable because of low coverage when processed separately, Joint calling allows evidence to be accumulated over all samples and renders the variant callable. In our re-analysis, we detected 58.7 million autosomal SNPs, while before only 55.4 million SNPs were found. The additionally called SNPs are due to the refined protocol and better genome reference. (line 378-387)

Minor issues:
Comment 2-4:
Lines 31-33: Genetic diversity is measured over all sites, not just the SNPs (see above).
Response 2-4:
It has been amended accordingly. We just say "A total of 58.7 million autosomal single nucleotide polymorphisms (SNPs) were detected". (line 32-33)

Comment 2-5:
Line 51: Not clear what 'successful' is supposed to mean here.
Response 2-5:
Judged by population size and geographic distribution, RMs are, after humans, the world's

most successful primate, occupying a vast geographic distribution. Here we replaced "'successful" with "most widely distributed" (line 55-56).

Comment 2-6:
Line 82: "including phylogenetic and demographic analyses, as well as genome-wide selection scans, …"
Response 2-6:
It has been amended accordingly. (line 87-90)

Comment 2-7:
Lines 97-98: The number of SNPs is not informative here, since it depends on the number of individuals. Use suitable measures of genetic diversity, such as Watterson's theta or pi.
Response 2-7:
It has been amended accordingly. The value of Watterson's θ (S) and genetic diversity (π) is 0.00342 and 0.00228, respectively (Table 1). (line 102-104)

Comment 2-8:
Lines 98-99: Not clear if the number of SNPs per individual refers to all positions with differences to the reference or only the heterozygous positions within individuals.
Response 2-8:
It refers to all positions with differences to the genome reference. (line 104-106)

Comment 2-9:
Lines 99-103: Use consistent style for point estimates and CI in the brackets, i.e. proportions instead of percentages.
Response 2-9:
It has been amended accordingly.

Comment 2-10:
Lines 103-105: Are these numbers only referring to shared segregating variation or also including fixed differences to the reference?
Response 2-10:
Including fixed differences to the genome reference. (line 106-108)

Comment 2-11:
Line 116: "admixture proportions"
Response 2-11:
It has been amended accordingly (line 120).

Comment 2-12:
Lines 149-150: "we further employed a joint site frequency spectrum (SFS) based approach to model"
Response 2-12:
It has been amended accordingly (line 167).

Comment 2-13:
Lines 152-153: Unclear what is meant by "produced a significantly better fit of a step by step divergence scenario than alternative ones, …"
Response 2-13:
This part has been re-written and this sentence has been removed.

Comment 2-14:
Line 167: Start a new sentence after "an eastern clade"
Response 2-14:
It has been amended accordingly (line 184).

Comment 2-15:
Lines 233-234: "we also found signatures of positive selection in genes related to …"
Response 2-15:
It has been amended accordingly. (line 292-293)

Comment 2-16:
Lines 234-235: The 104 candidate genes are enriched for a certain GO term, rather than the three genes being enriched in a certain GO term.
Response 2-16:
It has been amended accordingly (line 293-296).

Comment 2-17:
Lines 323-324: Provide more details about the variant calling here. Just providing the reference is not sufficient for the reader to get a quick overview of the applied methods.
Response 2-17:
It has been amended accordingly. We performed SNP calling following GATK's best practice and the more details about the variant calling protocol was described in line 378-388.

Comment 2-18:
Line 334: "branch support" instead of "branch reliability"
Response 2-18:
It has been amended accordingly (line 393).

Comment 2-19:
Line 343: "Decay of linkage disequilibrium against physical distance"
Response 2-19:
It has been amended accordingly (line 402-404).

Comment 2-20:
Line 349: Provide more details about the reasoning behind choosing the stated values for generation time and mutation rate.
Response 2-20:
For all demographic estimations, we have chosen a mutation rate of 1×10^{-8} per site per generation and a generation time of 11 yr, which were used in the previous population genomic analyses of 133 rhesus macaques (Xue et al. 2017). To compare our results and Xue's results, we toke the same values of mutation rate and generation time. Xue et al. explained the rationale behind these values: "The most appropriate mutation rate to use for this type of analysis remains somewhat controversial, in which a variety of methods have been used to determine the "best" estimate (Ségurel et al. 2014). For rhesus macaques, there is far less empirical evidence. We chose a mutation rate of 1.0×10^{-8} per site per generation for macaques, because a review of the data for humans suggests a rate of $1.0–1.5\times10^{-8}$ per site per generation (Ségurel et al. 2014). Assuming the generation time for rhesus macaques is 11 yr and humans is 25 yr, the per year mutation rates are then 0.9×10^{-9} for macaques

and 0.4–0.6×10−9 for humans, an appropriate ratio given the demonstrated slowdown in humans and other hominoids. Generation time is set at 11 yr based on the field data that indicate rhesus macaques begin reproduction ∼6 yr of age and can breed until their late teens, resulting in age at median birth of ∼11 yr.” However, perhaps it is not appropriate to paste this explanation into our manuscript. Thus I said “for the rationale to use these values see [6, 73] (Ségurel et al. 2014; Xue et al. 2017)”. (line 409-410)

Comment 2-21:
Line 353: "to model" rather than "to simulate"
Response 2-21:
It has been amended accordingly (line 415).

Comment 2-22:
Line 356: "to identify the one that is best supported by the observed data"
Response 2-22:
This part has been re-written and this sentence has been removed.

Comment 2-23:
Line 359: How many replicates?
Response 2-23:
Each model was tested for 200 replicates (line 420).

Comment 2-24:
Line 364: Which additional parameters? See comment above.
Response 2-24:
This part has been re-written and this sentence has been removed.

Comment 2-25:
Line 371-373: Rewrite this sentence.
Response 2-25:
It has been amended accordingly. (429-430)

Comment 2-26:
Line 379: "candidate regions under positive selection"
Response 2-26:
It has been amended accordingly (line 436).

Close
