# Supplementary material for: Population genomics of wild Chinese rhesus macaques reveals a dynamic demographic history and local adaptation, with implications for biomedical research
Source: Gigascience. 2018 Aug 27;7(9):giy106. doi: 10.1093/gigascience/giy106 (PMC6143732; doi:10.1093/gigascience/giy106)
Supplement: Response_to_Reviewer_Comments_Revision_1.pdf [file giy106_response_to_reviewer_comments_revision_1.pdf]

Author's Response To Reviewer Comments

Close

Dear editor,

We thank you for your encouragement and help to our manuscript “Population genomics of wild Chinese rhesus macaques reveals a dynamic demographic history and local adaptation, with implications for biomedical research”.

We have shorted the abstract and structured it in sections (“Background – Results - Conclusions”).

We have added a citation to our upcoming GigaDB dataset without the DOI at the end of the reference list and cited this in the ‘Data Access’.

The point-to-point responses to the reviewers are listed below.

Reviewer #1:
All my major concerns have been satisfactorily addressed. However, I see several typos or other minor problems with the text.

Comment_1-1:
line 162: should be "from" instead of "form"
line 230: I think the authors mean "upregulated" not "unregulated"
line 285: should be "have" not "has"
I think there are other similar issues.
Response_1-1:
These issues have been amended accordingly. (line 158, line 226, line 283)

Reviewer #2:

Comment_2-1:
The authors have revised their manuscript based on the detailed reviewer reports and have substantially improved it in the process. Most of my previous methodological concerns have been addressed. However, there still seems to be some confusion regarding my comments about proper calculation of genetic diversity (comment 2-3 and 2-4). The authors write in their rebuttal that they have used the GATK Best Practices workflow with multi-sample genotyping, thereby solving the problem of uncallable sites by accumulating evidence across samples. This is only partly mitigating the problem here. If a site is not callable across all samples due to poor mappability or a 'N' in the reference sequence, a genetic variant cannot be detected at this position. Thus, genetic diversity will be underestimated when assuming that variant sites can occur along the entire genome. To obtain an accurate estimate of genetic diversity, it is therefore crucial to consider the exact number of sites that are callable, i.e. where it is possible to detect a potential variant. This can be achieved for example with GATK's 'CallableLoci' module. Alternatively, the '--includeNonVariantSites' flag of GATK's 'GenotypeGVCFs' can be used to emit both confident variant and non-variant sites. To be considered in the calculation of genetic diversity, a site should be

callable (i.e. have a valid variant or non-variant genotype) in a certain proportion (e.g. 80%) of individuals. This initial site filter has to be independent of the variant state of a site, i.e. also variant sites in the SNP data set have to be filtered out if they don't fulfill the callability criteria.
Response_2-1:
We followed this helpful suggestion and redone the ‘GenotypeGVCFs’ in GATK with the '--includeNonVariantSites' flag to get both the variant and non-variant sites. Besides the basic hard filter by ‘VariantFiltration’ in GATK, we also filtered out the variants with a 'N' in the reference sequence or the sites including more than 20% missing genotypes. For the non-variant sites, we did the same filter and retain only the callable non-variant sites. The genetic diversity and heterozygosity have been re-estimated based on all the callable sites. (lines 101-102, lines 109-112, lines 379-390 and Table 1)

Comment_2-2:
Lines 76-79: These sentences are unclear. If to date only 9 captive Chinese RMs have been sequenced, how could Zhong et al. assess genetic diversity in 26 Chinese individuals? I guess the authors mean "Until recently, …" rather than "To date, …" at the beginning of the first sentence.
Response_2-2:
This issue has been amended accordingly. (lines 74-77)

Comment_2-3:
Line 102: This sentence is confusing. The authors write that they identified ~58 mio SNPs in the 81 Chinese RMs. From their explanations, I understood that this is the total number of variant sites, i.e. including fixed differences to the reference genome? The reference genome is of Indian origin, so it's incorrect to write that these are SNPs in Chinese RMs.
Response_2-3:
This issue has been amended accordingly. We have filtered out the fixed differences to the reference genome. (lines 386-388)

Comment_2-4:
Line 103: Was Watterson's theta correctly estimated considering only sites actually segregating within the Chinese RMs?
Response_2-4:
The θW and $\theta\pi$ have been re-estimated only based on segregating variations within Chinese RMs. (lines 101-102 and Table 1)

Comment_2-5:
Line 104: "and the nucleotide diversity measured by segregating sites (Watterson's θ, θW) and mean pairwise differences ($\theta\pi$) is …"
Response_2-5:
This issue has been amended accordingly. (lines 101-102)

Comment_2-6:
Lines 106-110: It doesn't make sense to use variant sites relative to a reference genome for the analysis of shared and private SNPs. These numbers reflect a mixture of segregating variation and fixed differences to the reference genome. Please redo these analyses by only considering actual segregating variation within the compared entities.
Response_2-6:
We have filtered out the fixed difference to the reference genome. All these analyses have

redone based on actual segregating variations within Chinese RMs. (lines 104-108, lines 387-388)

Comment_2-7:
Line 139: "based on θW and θπ are …"
Response_2-7:
This issue has been amended accordingly. (line 137)

Comment_2-8:
Lines 170-177: Round estimates and provide confidence intervals.
Response_2-8:
This issue has been amended accordingly. (lines 166-173)

Comment_2-9:
Lines 180-181: Tone down this statement, since you haven't explicitly compared models with and without gene flow. Something along the lines of: "Our results indicate that low levels of gene flow occurred between all five extant lineages of Chinese RMs."
Response_2-9:
This issue has been amended accordingly. Substantial gene flows have been detected between different subspecies. Please see the response to the comment_2-20 and Supplementary Table 5. (lines 177-178)

Comment_2-10:
Line 193: "led to further differentiation by limiting gene flow among them."
Response_2-10:
This issue has been amended accordingly. (line 190)

Comment_2-11:
Lines 208-211: This sentence seems to conflict with the sentence on lines 200-204.
Response_2-11:
For M. m. tcheliensis, which occurs in the northernmost range of the RMs under cold conditions, we first estimated FST and θπ between it and each of the other four subspecies. Then we got four lists of candidate genes in M. m. tcheliensis. The final positive selection genes are the intersection of these four lists. Similar, in the case of M. m. brevicaudus, we used the same method. The details of this process are shown in Supplementary Fig. 7. We chose this method to get the final positive selection genes, instead of directly comparing M. m. tcheliensis and M. m. brevicaudus, for the purpose of reducing the false positives of the results and obtaining more accurate selective gene lists. (lines 197-201, lines 205-208 and Supplementary Fig. 8)

Comment_2-12:
Lines 223-226: See previous comment 2-16.
Response_2-12:
This issue has been amended accordingly. (lines 220-223)

Comment_2-13:
Line 230: "upregulated" instead of "unregulated"?
Response_2-13:
This issue has been amended accordingly. (line 226)

Comment_2-14:
Lines 240-241: Having long forearms doesn't really fit the expectation, as long extremities would increase the surface to volume ration. I realize that forearm length is probably strongly correlated with body size, but this is confusing for the reader. Maybe just omit the forearm length.
Response_2-14:
This issue has been amended accordingly. We have omitted the forearm length in the revised manuscript. Many thanks for this helpful suggestion. (lines 237-238)

Comment_2-15:
Line 385-387: Provide details about the filter settings. The current description of the variant hard filtering approach doesn't allow to reproduce the data set used for the downstream analyses.
Response_2-15:
This issue has been amended accordingly. After variant calling, we first applied the “SelectVariants” to exclude the Indel and split the variant and non-variant sites. Then we applied the hard filter command ‘VariantFiltration’ to exclude potential false-positive variant calls with the following criteria: “–filterExpression ‘QD < 5.0 ‖ FS > 60.0 ‖ MQ < 40.0 ‖ ReadPosRankSum< −8.0 || MQRankSum < -12.5’” and “--genotypeFilterExpression ‘DP < 4.0’”. Additionally, the sites are filtered if there is a 'N' is in the reference sequence; if the site is fixed difference to the reference genome or if the site including more than 20% missing genotypes. (lines 383-388)

Comment_2-16:
Line 410-411: Provide details of how the consensus sequences have been generated.
Response_2-16:
This issue has been amended accordingly. We called the consensus sequences using Samtools mpileup [68] by applying: “samtools mpileup -q 1 -C 50 -S -D -m 2 -F 0.002 -u -f *.fa(genome) *.bam | bcftools view -c - | vcfutils.pl vcf2fq -d 10 -D 100 -Q 20 - > *.psmc.fq” and “fq2psmcfa -q10 -s 100 *.psmc.fq >*.psmc.fa”. To ensure the quality of consensus sequences, we used data of ten individuals with an average coverage >20× (22.20-34.32×). (lines 411-415)

Comment_2-17:
Line 418: Provide more details of how the SNP data has been converted to joint site frequency spectra. How was the number of non-variant sites assessed accurately (see comment above)?
Response_2-17:
VCF file containing callable variant sites was used converted to fastsimcoal style folded SFS. To mitigate the effect of linkage disequilibrium, we filtered out the SNPs located within 10 kb from genes and then we took one SNPs every 10kb randomly. The multidimensional folded SFS for all the five subspecies is generated by easySFS (https://github.com/isaacovercast/easySFS#easysfs). The non-variant sites were not used to convert the SFS. (lines 426-427)

Comment_2-18:
Lines 422-423: Not sure what this is supposed to mean. Have you simulated data sets under the inferred model and compared distributions of simulated summary statistics to the observed values? However, Supplementary Table 5 doesn't show distributions of summary statistics, rather estimates of model parameters. Provide details of how confidence intervals

have been calculated and show how good the model fits the observed data.
Response_2-18:
Lines 422-423 is a typo-error and has been removed. We got confidence intervals after parameter estimation using parametric bootstraps. We chose the replicate with the highest estimated maximum likelihood to generate parametric bootstraps. One hundred multidimensional SFS files were generated for this set of parameters and then estimated parameters from these pseudo-observed data sets using the same tpl and est files as those used to get the parameters with highest likelihood. We used the option '-initvalues file.pv' to reduce the number of runs necessary to estimate parameters when estimating confidence intervals by bootstrap. The 'file.pv' containing initial parameter values for parameter estimation is automatically generated after parameter estimation by fsc26. The observed data and the confidence intervals from 100 parametric bootstraps were showed in Supplementary Fig. 7 and Supplementary Table 5. (lines 427-437)

Comment_2-19:
Line 475: I haven't been able to find any notes in the Supplementary Information.
Response_2-19:
This issue has been amended accordingly. According to the format of GigaScience, the supplementary files does not include any notes. This confusion on line 475 is a typo and we have modified it (line 491).

Comment_2-20:
Supplementary Table 5: Are the gene flow estimates really representing the number of migrants (i.e. Nem)? This would be completely negligible gene flow. Or are these numbers rather migration rates (i.e. m), which would imply quite substantial gene flow.
Response_2-20:
It is a typo-error. The gene flow estimated really represent the migration rate between subspecies, which imply quite substantial gene flow (Supplementary Table 5).

Close
