# Supplementary material for: Population genomics of wild Chinese rhesus macaques reveals a dynamic demographic history and local adaptation, with implications for biomedical research
Source: Gigascience. 2018 Aug 27;7(9):giy106. doi: 10.1093/gigascience/giy106 (PMC6143732; doi:10.1093/gigascience/giy106)
Supplement: Reviewer_1_Report_(Original_Submission) -- Jeffrey Rogers — 12/27/2017 Reviewed [file giy106_reviewer_1_report_(original_submission).pdf]

**Reviewer Report**

**Title:** **Population genomics of wild Chinese rhesus macaques reveals a dynamic demographic history and local adaptation, with implications for biomedical research**

**Version:** **Original Submission** **Date:** 12/27/2017

**Reviewer name:** **Jeffrey Rogers**

**Reviewer Comments to Author:**

This manuscript reports a novel and substantive analysis of population genetic variation among Chinese-origin rhesus macaques. The authors have generated whole genome sequence data for 81 wild rhesus monkeys from 17 distinct localities in China. These samples include all five major regional populations (subspecies) within Chinese rhesus macaques. The authors use genome-wide SNP data to investigate phylogenetic relationships among the subspecies, demographic history for each, and possible signatures of natural selection (selective sweeps) related to ecological and environmental differences among the five major populations (subspecies).

This is an important and timely study that provides a significant increase in the available information regarding genetic diversity within this widely studied nonhuman primate. Little in known about whole genome polymorphism in Chinese rhesus. As the authors correctly argue, the rhesus macaque is a widely distributed and intensively studied primate. The species is important for both fundamental studies of primate biology and for more applied biomedical analyses. Consequently, detailed information about genetic variation within and among the Chinese populations of this species, and associated analysis of the implications of that variation for population relationships and demographic history is valuable.

This manuscript investigates three different aspects of the population genetics of Chinese rhesus. First, the authors describe analyses of population relationships (phylogeny) and demographic history. For these analyses, the authors use genome-wide SNP genotypes determined by mapping Illumina short read sequencing data to the rhemac2 rhesus macaque reference genome assembly. This reference genome (rhemac2) was produced a number of years ago using Sanger sequencing methods, and DNA from an Indian-origin rhesus macaque. The phylogenetic, demographic and evolutionary analyses reported here (neighbor joining phylogeny, population structure studies using STRUCTURE2.3.4, demographic analysis using PSMC and fastsimcoal2) are well-designed and provide interesting new insights into the pattern of genetic variation among populations within Chinese rhesus. The results provide evidence for previously unrecognized relationships among the five regional populations, and also generate an interesting scenario regarding changes in effective population sizes over the last 2 million years. The more recent fastsimcoal2 analyses are especially valuable in outlining dates of divergence among lineages, and effective sizes of those lineages.

The rhemac2 reference genome assembly is an old genome reference sequende. There are now more recent, higher quality reference assemblies for this species. However, I do not think that the use of rhemac2 is necessarily a major problem for the population phylogenetics and demographic analyses. SNPs identified using rhemac2 should be very similar (though not identical) to the SNP calls that would be obtained using the more recent assemblies. And it should not be a problem that the rhemac2 assembly is built from an Indian-origin animal. It would have been better (more comprehensive and less susceptible to errors) for the authors to use a more recent reference genome, but using rhemac2 for the evolutionary and demographic analyses does not seem to me to be a major concern.

The second aspect of the paper is an analysis of functional genetic variation. The authors used FST and other population statistics to identify regions of the macaque genome that show significant differentiation

among populations, focusing particularly on the most northern and most southern populations. These analyses suggest that there has been selection for differences in skeletal development and cardiovascular physiology that distinguish Chinese rhesus subspecies (selective sweeps). I do have some concerns about these analyses.

a) First and most importantly, this is where the use of rhemac2 as the reference assembly seems to me to be somewhat problematic. The rhemac2 assembly contains some assembly errors. But more relevant to this manuscript, it was annotated by NCBI and Ensembl before there was substantial RNA sequence data to assist in gene prediction. Investigators who have used rhemac2 for functional studies of protein-coding genes have found errors in some of the gene models, likely due to the lack of access to good RNA sequence data at the time of the annotation. The newer reference genomes for rhesus macaque (e.g. Mmul_8.0.1) have also been annotated by NCBI and Ensembl. These newer annotations are more complete and more accurate because there is now more RNA sequence data available to support gene models and to identify true exon-intron boundaries. I would be concerned that some of the conclusions Liu et al. have generated regarding selection on specific genes may be problematic due to potential problems with rhemac2 gene annotations. Even though the analyses depend on FST and related statistics 9 (and not dN/dS ratios), I assume that the authors did examine the coding sequence differences among Chinese rhesus populations for the genes that they infer were under selection. I recommend that the authors (at a minimum) re-check their analyses and conclusions regarding positive selection on specific genes, using the more accurate, better annotated reference assemblies that were produced more recently than rhemac2.

b) It is not clear from this version of the manuscript (lines 207-219) whether Liu et al. observed any non-synonymous variants in the genes they identified as showing evidence of selective sweeps. Were there non-synonymous differences in the alleles found in the different Chinese rhesus populations, or were all the FST values based on intronic and/or intervening SNPs between genes? The case for positive selection on PAPSS2, SOX5 and other genes would be stronger if the authors identified non-synonymous or other coding variants that are predicted to influence protein function. If there are no non-synonymous differences observed between populations, then Liu et al. would (I suppose) have to argue that the selection was on non-coding regulatory variants. No specific statement about how the proposed selection is suggested to have influenced these genes is presented in the manuscript. Readers should be informed as to what particular variants distinguish the alleles in M. m. tcheliensis from M. m. brevicaudus, etc., and why the authors believe the observed sequence differences constitute true functional differences.

c) It is not stated (lines 220-232) whether the GO terms related to heart development, heart rate or temperature response are statistically significantly enriched in this analysis. The authors should provide the same type of statistical evidence for these GO term results that they do for the limb morphogenesis results above.

The new results presented in this paper regarding phylogenetic relationships among populations, and the history of population differentiation and effective size change, are important findings and make a valuable contribution to the literature.

Other minor issues:

Line 79: I think there may be a typo here. I do not think the authors intend to state that the effective population size of Indian rhesus macaques is only 17,000. This should be checked again.

Lines 137-148: It might be useful to compare the results for population size change over time that Liu et al. obtain here to those of previous population genetic analyses of rhesus macaques (e.g. Xue et al. 2016 and Hernandez et al. 2007).

Lines 145-148: How do the authors reconcile the different estimates for effective population size at about 60-80,000 years ago for M. m. tcheliensis, M. m. littoralis and M. m. brevicaudus that were obtained by the

PSMC analysis versus the fastsimcoal2 analysis? Do the authors favor one of these over the other? Is there possibly a way to reconcile these different results?

**Methods**

Are the methods appropriate to the aims of the study, are they well described, and are necessary controls included? No

**Conclusions**

Are the conclusions adequately supported by the data shown? No

**Reporting Standards**

Does the manuscript adhere to the journal's guidelines on minimum standards of reporting? Yes

Choose an item.

**Statistics**

Are you able to assess all statistics in the manuscript, including the appropriateness of statistical tests used? Yes, and I have assessed the statistics in my report.

**Quality of Written English**

Please indicate the quality of language in the manuscript: Acceptable

**Declaration of Competing Interests**

Please complete a declaration of competing interests, considering the following questions:

- Have you in the past five years received reimbursements, fees, funding, or salary from an organisation that may in any way gain or lose financially from the publication of this manuscript, either now or in the future?
- Do you hold any stocks or shares in an organisation that may in any way gain or lose financially from the publication of this manuscript, either now or in the future?
- Do you hold or are you currently applying for any patents relating to the content of the manuscript?
- Have you received reimbursements, fees, funding, or salary from an organization that holds or has applied for patents relating to the content of the manuscript?
- Do you have any other financial competing interests?
- Do you have any non-financial competing interests in relation to this paper?

If you can answer no to all of the above, write 'I declare that I have no competing interests' below. If your reply is yes to any, please give details below.

I declare that I have no competing interests

I agree to the open peer review policy of the journal. I understand that my name will be included on my report to the authors and, if the manuscript is accepted for publication, my named report including any attachments I upload will be posted on the website along with the authors' responses. I agree for my report to be made available under an Open Access Creative Commons CC-BY license (http://creativecommons.org/licenses/by/4.0/). I understand that any comments which I do not wish to be included in my named report can be included as confidential comments to the editors, which will not be published.

I agree to the open peer review policy of the journal

To further support our reviewers, we have joined with Publons, where you can gain additional credit to further highlight your hard work (see: https://publons.com/journal/530/gigascience). On publication of this paper, your review will be automatically added to Publons, you can then choose whether or not to claim your Publons credit. I understand this statement.

Yes Choose an item.
