# Supplementary material for: Population genomics of wild Chinese rhesus macaques reveals a dynamic demographic history and local adaptation, with implications for biomedical research
Source: Gigascience. 2018 Aug 27;7(9):giy106. doi: 10.1093/gigascience/giy106 (PMC6143732; doi:10.1093/gigascience/giy106)
Supplement: Reviewer_2_Report_(Original_Submission) -- Alexander Nater — 01/04/2018 Reviewed [file giy106_reviewer_2_report_(original_submission).pdf]

Reviewer Report

Title: Population genomics of wild Chinese rhesus macaques reveals a dynamic demographic history and local adaptation, with implications for biomedical research

Version: Original Submission **Date:** 1/4/2018

Reviewer name: Alexander Nater

Reviewer Comments to Author:

This manuscript by Liu and colleagues presents a comprehensive set of resequenced genomes of 81 geo-referenced individuals of Chinese rhesus macaques. The authors reconstructed the demographic history of the Chinese subspecies and performed genome scans for signatures of positive selection. My main criticism is that the paper lacks clear hypotheses and is purely exploratory. Moreover, the analyses of positive selection and demography remain rather superficial and the authors don't discuss their results in sufficient detail to provide a comprehensive picture of the evolutionary history of Chinese rhesus macaques. Nevertheless, the paper represents a useful genomic resource for further studies and can make a valuable contribution given that some methodological issues can be addressed in a revised version.

In the first part, the authors reconstruct the phylogeny of Chinese rhesus macaques based on a whole-genome neighbor-joining tree. This is a rather crude type of phylogenomic analysis and doesn't allow to draw conclusions about the evolutionary history as done on lines 109-114. Here, the paper would benefit a lot from applying proper species tree methods that take incomplete lineage sorting into account. This will provide a reliable picture about the phylogenetic relationships of the five subspecies that can then act as a useful starting point to design a set of demographic models to test in the next step.

My main concern deals with the design of the models for demographic model testing. Here, the paper lacks critical details to understand the reasoning behind the selection of the 8 compared models. It's completely unclear how these models have been chosen from the total number of possible (sub)species tree configurations and how they were parameterized. Supplementary Table 5 shows that the number of parameters in these 8 models range from 6 to 12, but they seem to do so in a very unintuitive way. For example, in Supplementary Figure 6 it seems that model 2 is a simplified version of model 8 with one less divergence time parameter. But Supplementary Table 5 shows that model 2 has actually 3 parameters more than model 8. Moreover, for parameter estimation, the authors expanded the selected model 2 by additional parameters without specifying which of the parameters listed in Supplementary Table 6 have already been part of the model selection. Comparing oversimplified models might lead to the selection of a suboptimal model in the first step. It's therefore absolutely crucial that the authors provide a detailed table showing the parameterization of all tested models (including parameter bounds) and explain in detail the reasoning behind the selection and design of these models. The type and parameterization of models has a strong impact on the outcome of such model testing approaches and without this critical information, it's impossible to assess how robust the findings of this analysis actually are. Additionally, the authors should provide a measure of the goodness of fit of the selected scenario to show that this model can reasonably well explain the observed data.

In the positive selection analysis, the authors calculate genetic diversity (theta pi) based on their set of variable sites only. This approach is flawed, as it doesn't allow to distinguish between non-variable sites and sites that are not sufficiently covered for reliable genotyping in the sequenced individuals. It is therefore important that the authors take coverage information for every site in the genome into account in order to obtain reliable estimates of window-wise genetic diversity.

Minor issues:

Lines 31-33: Genetic diversity is measured over all sites, not just the SNPs (see above).
Line 51: Not clear what 'successful' is supposed to mean here.
Line 82: "including phylogenetic and demographic analyses, as well as genome-wide selection scans, …"
Lines 97-98: The number of SNPs is not informative here, since it depends on the number of individuals. Use suitable measures of genetic diversity, such as Watterson's theta or pi.
Lines 98-99: Not clear if the number of SNPs per individual refers to all positions with differences to the reference or only the heterozygous positions within individuals.
Lines 99-103: Use consistent style for point estimates and CI in the brackets, i.e. proportions instead of percentages.
Lines 103-105: Are these numbers only referring to shared segregating variation or also including fixed differences to the reference?
Line 116: "admixture proportions"
Lines 149-150: "we further employed a joint site frequency spectrum (SFS) based approach to model"
Lines 152-153: Unclear what is meant by "produced a significantly better fit of a step by step divergence scenario than alternative ones, …"
Line 167: Start a new sentence after "an eastern clade"
Lines 233-234: "we also found signatures of positive selection in genes related to …"
Lines 234-235: The 104 candidate genes are enriched for a certain GO term, rather than the three genes being enriched in a certain GO term.
Lines 323-324: Provide more details about the variant calling here. Just providing the reference is not sufficient for the reader to get a quick overview of the applied methods.
Line 334: "branch support" instead of "branch reliability"
Line 343: "Decay of linkage disequilibrium against physical distance"
Line 349: Provide more details about the reasoning behind choosing the stated values for generation time and mutation rate.
Line 353: "to model" rather than "to simulate"
Line 356: "to identify the one that is best supported by the observed data"
Line 359: How many replicates?
Line 364: Which additional parameters? See comment above.
Line 371-373: Rewrite this sentence.
Line 379: "candidate regions under positive selection"

Methods

Are the methods appropriate to the aims of the study, are they well described, and are necessary controls included? No

Conclusions

Are the conclusions adequately supported by the data shown? Yes

Reporting Standards

Does the manuscript adhere to the journal's guidelines on minimum standards of reporting? Yes

Choose an item.

Statistics

Are you able to assess all statistics in the manuscript, including the appropriateness of statistical tests used? Yes, and I have assessed the statistics in my report.

Quality of Written English

Please indicate the quality of language in the manuscript: Needs some language corrections before being published

Declaration of Competing Interests

Please complete a declaration of competing interests, considering the following questions:

- Have you in the past five years received reimbursements, fees, funding, or salary from an organisation that may in any way gain or lose financially from the publication of this manuscript, either now or in the future?
- Do you hold any stocks or shares in an organisation that may in any way gain or lose financially from the publication of this manuscript, either now or in the future?
- Do you hold or are you currently applying for any patents relating to the content of the manuscript?
- Have you received reimbursements, fees, funding, or salary from an organization that holds or has applied for patents relating to the content of the manuscript?
- Do you have any other financial competing interests?
- Do you have any non-financial competing interests in relation to this paper?

If you can answer no to all of the above, write 'I declare that I have no competing interests' below. If your reply is yes to any, please give details below.

I declare that I have no competing interests.

I agree to the open peer review policy of the journal. I understand that my name will be included on my report to the authors and, if the manuscript is accepted for publication, my named report including any attachments I upload will be posted on the website along with the authors' responses. I agree for my report to be made available under an Open Access Creative Commons CC-BY license (http://creativecommons.org/licenses/by/4.0/). I understand that any comments which I do not wish to be included in my named report can be included as confidential comments to the editors, which will not be published.

I agree to the open peer review policy of the journal

To further support our reviewers, we have joined with Publons, where you can gain additional credit to further highlight your hard work (see: https://publons.com/journal/530/gigascience). On publication of this paper, your review will be automatically added to Publons, you can then choose whether or not to claim your Publons credit. I understand this statement.

Yes Choose an item.
