# Supplementary material for: Population genomics of wild Chinese rhesus macaques reveals a dynamic demographic history and local adaptation, with implications for biomedical research
Source: Gigascience. 2018 Aug 27;7(9):giy106. doi: 10.1093/gigascience/giy106 (PMC6143732; doi:10.1093/gigascience/giy106)
Supplement: Reviewer_2_Report_(Revision_1) -- Alexander Nater — 04/15/2018 Reviewed [file giy106_reviewer_2_report_(revision_1).pdf]

Reviewer Report

Title: Population genomics of wild Chinese rhesus macaques reveals a dynamic demographic history and local adaptation, with implications for biomedical research

Version: **Revision 1** **Date:** 4/15/2018

Reviewer name: **Alexander Nater**

Reviewer Comments to Author:

The authors have revised their manuscript based on the detailed reviewer reports and have substantially improved it in the process. Most of my previous methodological concerns have been addressed. However, there still seems to be some confusion regarding my comments about proper calculation of genetic diversity (comment 2-3 and 2-4). The authors write in their rebuttal that they have used the GATK Best Practices workflow with multi-sample genotyping, thereby solving the problem of uncallable sites by accumulating evidence across samples. This is only partly mitigating the problem here. If a site is not callable across all samples due to poor mappability or a 'N' in the reference sequence, a genetic variant cannot be detected at this position. Thus, genetic diversity will be underestimated when assuming that variant sites can occur along the entire genome. To obtain an accurate estimate of genetic diversity, it is therefore crucial to consider the exact number of sites that are callable, i.e. where it is possible to detect a potential variant. This can be achieved for example with GATK's 'CallableLoci' module. Alternatively, the '--includeNonVariantSites' flag of GATK's 'GenotypeGVCFs' can be used to emit both confident variant and non-variant sites. To be considered in the calculation of genetic diversity, a site should be callable (i.e. have a valid variant or non-variant genotype) in a certain proportion (e.g. 80%) of individuals. This initial site filter has to be independent of the variant state of a site, i.e. also variant sites in the SNP data set have to be filtered out if they don't fulfill the callability criteria.

Minor comments:
Lines 76-79: These sentences are unclear. If to date only 9 captive Chinese RMs have been sequenced, how could Zhong et al. assess genetic diversity in 26 Chinese individuals? I guess the authors mean "Until recently, …" rather than "To date, …" at the beginning of the first sentence.
Line 102: This sentence is confusing. The authors write that they identified ~58 mio SNPs in the 81 Chinese RMs. From their explanations, I understood that this is the total number of variant sites, i.e. including fixed differences to the reference genome? The reference genome is of Indian origin, so it's incorrect to write that these are SNPs in Chinese RMs.
Line 103: Was Watterson's theta correctly estimated considering only sites actually segregating within the Chinese RMs?
Line 104: "and the nucleotide diversity measured by segregating sites (Watterson's θ, θW) and mean pairwise differences ($\theta \pi$) is …"
Lines 106-110: It doesn't make sense to use variant sites relative to a reference genome for the analysis of shared and private SNPs. These numbers reflect a mixture of segregating variation and fixed differences to the reference genome. Please redo these analysis by only considering actual segregating variation within the compared entities.
Line 139: "based on θW and $\theta \pi$ are …"
Lines 170-177: Round estimates and provide confidence intervals.
Lines 180-181: Tone down this statement, since you haven't explicitly compared models with and without gene flow. Something along the lines of: "Our results indicate that low levels of gene flow occurred between all five extant lineages of Chinese RMs."
Line 193: "led to further differentiation by limiting gene flow among them."
Lines 208-211: This sentence seems to conflict with the sentence on lines 200-204.
Lines 223-226: See previous comment 2-16.

Line 230: "upregulated" instead of "unregulated"?
Lines 240-241: Having long forearms doesn't really fit the expectation, as long extremities would increase the surface to volume ration. I realize that forearm length is probably strongly correlated with body size, but this is confusing for the reader. Maybe just omit the forearm length.
Line 385-387: Provide details about the filter settings. The current description of the variant hard filtering approach doesn't allow to reproduce the data set used for the downstream analyses.
Line 410-411: Provide details of how the consensus sequences have been generated.
Line 418: Provide more details of how the SNP data has been converted to joint site frequency spectra. How was the number of non-variant sites assessed accurately (see comment above)?
Lines 422-423: Not sure what this is supposed to mean. Have you simulated data sets under the inferred model and compared distributions of simulated summary statistics to the observed values? However, Supplementary Table 5 doesn't show distributions of summary statistics, rather estimates of model parameters. Provide details of how confidence intervals have been calculated and show how good the model fits the observed data.
Line 475: I haven't been able to find any notes in the Supplementary Information.
Supplementary Table 5: Are the gene flow estimates really representing the number of migrants (i.e. Nem)? This would be completely negligible gene flow. Or are these numbers rather migration rates (i.e. m), which would imply quite substantial gene flow.

Methods

Are the methods appropriate to the aims of the study, are they well described, and are necessary controls included? Choose an item.

Conclusions

Are the conclusions adequately supported by the data shown? Choose an item.

Reporting Standards

Does the manuscript adhere to the journal's guidelines on minimum standards of reporting? Choose an item.

Choose an item.

Statistics

Are you able to assess all statistics in the manuscript, including the appropriateness of statistical tests used? Choose an item.

Quality of Written English

Please indicate the quality of language in the manuscript: Choose an item.

Declaration of Competing Interests

Please complete a declaration of competing interests, considering the following questions:

- Have you in the past five years received reimbursements, fees, funding, or salary from an organisation that may in any way gain or lose financially from the publication of this manuscript, either now or in the future?
- Do you hold any stocks or shares in an organisation that may in any way gain or lose financially from the publication of this manuscript, either now or in the future?
- Do you hold or are you currently applying for any patents relating to the content of the manuscript?
- Have you received reimbursements, fees, funding, or salary from an organization that holds or has applied for patents relating to the content of the manuscript?
- Do you have any other financial competing interests?
- Do you have any non-financial competing interests in relation to this paper?

If you can answer no to all of the above, write 'I declare that I have no competing interests' below. If your reply is yes to any, please give details below.

I declare that I have no competing interests.

I agree to the open peer review policy of the journal. I understand that my name will be included on my report to the authors and, if the manuscript is accepted for publication, my named report including any attachments I upload will be posted on the website along with the authors' responses. I agree for my report to be made available under an Open Access Creative Commons CC-BY license (http://creativecommons.org/licenses/by/4.0/). I understand that any comments which I do not wish to be included in my named report can be included as confidential comments to the editors, which will not be published.

I agree to the open peer review policy of the journal

To further support our reviewers, we have joined with Publons, where you can gain additional credit to further highlight your hard work (see: https://publons.com/journal/530/gigascience). On publication of this paper, your review will be automatically added to Publons, you can then choose whether or not to claim your Publons credit. I understand this statement.

Yes Choose an item.
